# Enantioselective synthesis of cyclopenta[*b*]benzofurans *via* an organocatalytic intramolecular double cyclization†
†Electronic supplementary information (ESI) available. CCDC 1545253. For ESI and crystallographic data in CIF or other electronic format see DOI: 10.1039/c7sc03006a


**DOI:** 10.1039/c7sc03006a

**Published:** 2017-10-02

**Authors:** Bruno Matos Paz, Yang Li, Mathias Kirk Thøgersen, Karl Anker Jørgensen

**Affiliations:** a Department of Chemistry , Aarhus University , DK-8000 Aarhus C , Denmark . Email: kaj@chem.au.dk

## Abstract

An enantioselective organocatalytic strategy, combining Brønsted base and N-heterocyclic carbene catalysis in a unique manner, is demonstrated for a concise construction of the privileged cyclopenta[*b*]benzofuran scaffold, present in many bioactive compounds having both academic and commercial interests. The reaction concept relies on an intramolecular one-pot double cyclization involving a cycle-specific enantioselective Michael addition followed by a benzoin condensation of *ortho*-substituted cinnamaldehydes. Cyclopenta[*b*]benzofurans were achieved in moderate to good yields, with excellent stereoselectivities. A proof of principle for a diastereodivergent variation is demonstrated through the synthesis of cyclopenta[*b*]benzofurans containing two contiguous aromatic substituents in a substitution pattern present in commercial and natural compounds. Furthermore, several transformations have been performed, demonstrating the synthetic utility of the products. Finally, insights into the activation mode and stereoindution models are presented for this new synthetic strategy.

## Abstract 2

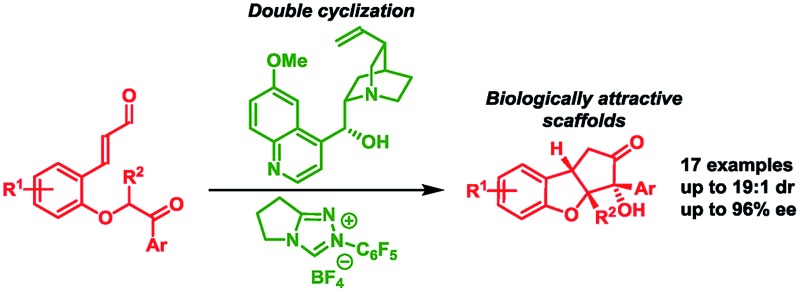
An enantioselective organocatalytic strategy, combining Brønsted base and N-heterocyclic carbene catalysis in a unique manner, is demonstrated for a concise construction of the privileged cyclopenta[*b*]benzofuran scaffold, present in many bioactive compounds having both academic and commercial interests.

## Introduction

Natural products play a key role in the drug discovery process.^1^ The structural complexity of these compounds – present in the form of several ring systems, functional groups, stereocenters and pharmacophores – has been built up through the chemical evolution in biological systems. However, these outstanding features of natural products are a double-edged knife for their applications. The chemical space around them have, so far, been more likely to give “hits” in drug screening process compared to *e.g.* high throughput screening techniques,^2^ despite the improvements obtained by diversity oriented synthesis.^3^ On the other hand, some drawbacks severely undermine the applicability of natural products. These include the low availability of secondary metabolites from natural sources, as well as the complex and costly synthetic endeavors necessary for their preparation in useful quantities.^4^

Biology oriented synthesis (BIOS)^5^ and function oriented synthesis (FOS)^6^ are complementary conceptual frameworks that aim to overcome some of the challenges for studying natural products in drug discovery.^7^ In several instances these concepts couple molecular simplification approaches with the current stage of available synthetic methodologies and strategies to generate libraries of analogs in a rational and efficient manner.

**Fig. 1:**
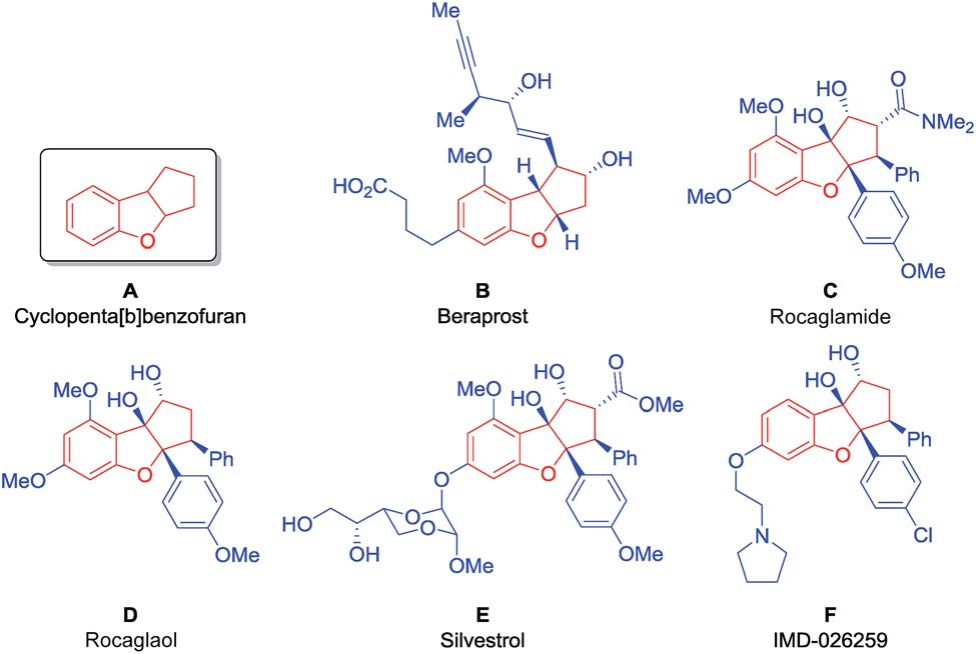
Bioactive compounds containing the cyclopenta[*b*]benzofuran scaffold.

Aiming to develop a new tool suitable for the application of BIOS and FOS concepts, we decided to investigate an organocatalytic approach for an enantioselective synthesis of the privileged scaffold cyclopenta[*b*]benzofuran (Fig. 1, **A**), present in many natural, synthetic and even commercial bioactive compounds.

Beraprost (Fig. 1, **B**) is a stable and orally active drug with antiplatelet and vasodilating properties, being applied for the treatment of patients with pulmonary arterial hypertension and peripheral artery disease.^8^ It is the first example of a drug with a cyclopenta[*b*]benzofuran scaffold to enter the market.^9^

The cyclopenta[*b*]benzofuran scaffold is also present in flavaglines, a family of biologically active natural products, first discovered in 1982, with the identification of rocaglamide (Fig. 1, **C**).10*a* More than a hundred other flavaglines have since been discovered, including rocaglaol (Fig. 1, **D**) and silvestrol (Fig. 1, **E**).10*b* Their pharmacological properties include the potential for treating inflammatory, cardiac and neurological diseases. Remarkably, they have also shown the ability to induce the death of human cancer cells while promoting the survival of non-cancer cells against many forms of stress, at nanomolar concentrations.^11^ Compound IMD-026259, which was designed based on the structure of rocaglaol, is a pre-clinical candidate for the treatment of Parkinson's disease (Fig. 1, **F**).^12^

Given the outstanding potential of the cyclopenta[*b*]benzofuran scaffold for drug development, this class of compounds has attracted the attention of the synthetic community. A large number of strategies have been developed for the synthesis of these cyclopenta[*b*]benzofuran derived molecules, such as palladium catalyzed [3 + 2] cycloadditions,^13^ Nazarov cyclizations,^14^–^16^ intramolecular epoxide openings^17^,^18^ and umpolung approaches.^19^–^21^ Furthermore, a racemic [3 + 2] photocycloaddition was performed^22^ inspired by its biosynthesis,^23^ which was later developed to be enantioselective *via* hydrogen-bonding catalysis, using TADDOL as the catalyst.^24^ This approach was then combined with flow chemistry technology to produce a series of silvestrol analogues (Fig. 1, **E**).^25^

## Synthetic design

**Scheme 1:**
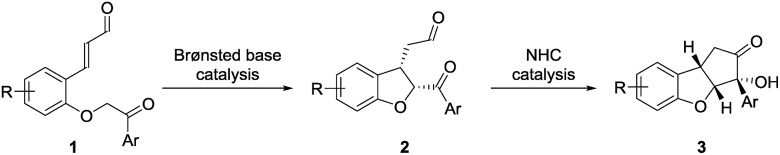
Double cyclization strategy for the synthesis of cyclopenta[*b*]benzofurans.

The above-mentioned strategies for the synthesis of the cyclopenta[*b*]benzofuran scaffold are all based on multi-step reaction approaches, which makes the assembly of the fused ring for the synthesis of analogs costly and time demanding. To overcome this, we envisioned that a straightforward strategy capable of providing all the ring systems of the cyclopenta[*b*]benzofuran scaffold in a one-pot fashion could be very attractive.^26^ An approach relying on one class of compounds, *ortho*-substituted cinnamaldehydes, easily prepared from readily available starting materials is also desirable. We anticipated that a double cyclization consisting of an intramolecular Michael addition followed by a N-heterocyclic carbene (NHC)-catalyzed benzoin condensation would afford – in a one-pot fashion – both ring systems starting from *ortho*-substituted cinnamaldehydes **1** (Scheme 1).^27^

**Scheme 2:**
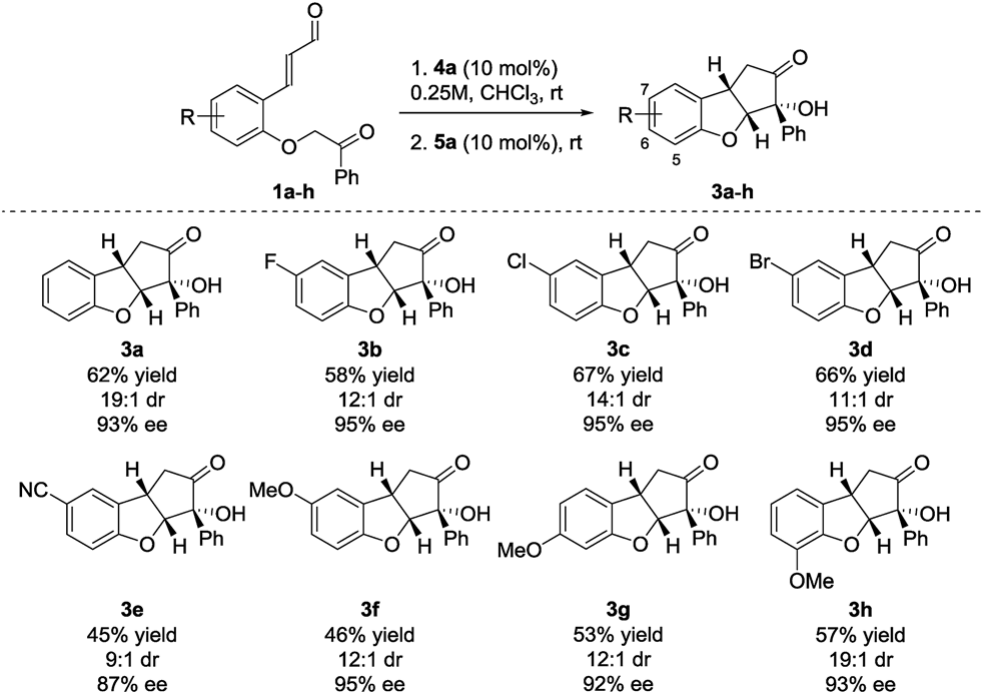
Substituents scope for the Brønsted-base/NHC-catalyzed double cyclization. Reactions were performed on a 0.25 mmol scale. Diastereomeric ratio was determined by ^1^H NMR analysis of the crude reaction mixture. Enantiomeric excess was determined by UPC^2^.

**Scheme 3:**
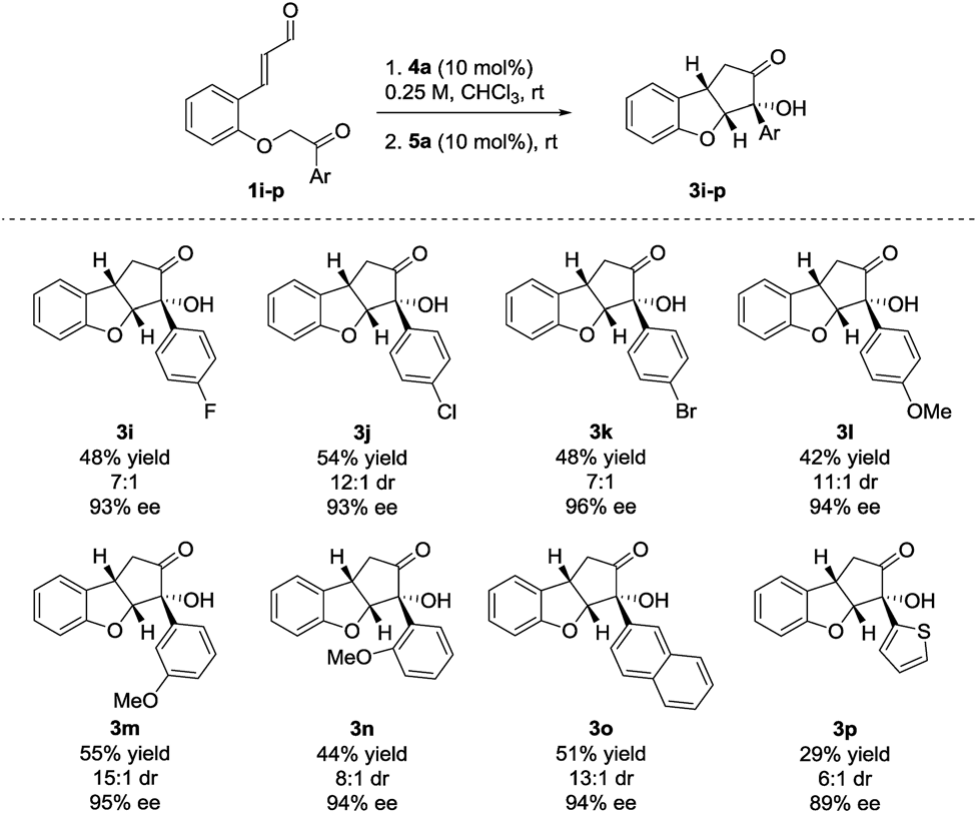
Side-chain scope for the Brønsted base/NHC-catalyzed double cyclization. Reactions were performed on a 0.25 mmol scale. Diastereomeric ratio was determined by ^1^H NMR analysis of the crude reaction mixture. Enantiomeric excess was determined by UPC^2^.

In order to resemble the known biologically active compounds, stereochemical concerns must also be taken into account. Thus, it is desirable for the cyclopenta[*b*]benzofuran product to have a *cis*-ring fusion (Scheme 1, **2**), while the aryl side-chain at the tetrasubstituted chiral carbon should preferably be at the exo-face (Scheme 1, **3**). A chiral Brønsted base, with a basicity suitable to deprotonate the substrate^28^ as well as the NHC-precatalyst, while tolerating the aldehyde functional group,^29^ would be the ideal catalyst candidate. This would render the Brønsted base a double role: to catalyze the enantioselective Michael addition and be an initiator for the NHC-catalyzed benzoin condensation. NHC-catalysis has been performed in combination with transition-metal, hydrogen-bond donor, Lewis acid, Brønsted-acid and Brønsted-base catalysis.^30^ However, to the best of our knowledge, despite its conceptual simplicity, a cycle-specific^31^ enantioselective Michael addition/benzoin condensation *via* Brønsted base/NHC-catalysis approach have not been achieved. Thus, the two catalytic systems operate in a complementary manner as the Brønsted base acts as the base for activating the NHC-catalyst.

## Results and discussion

To evaluate the strategy, a series of commercially available cinchona alkaloids **4** and NHC-catalysts **5** were tested as catalysts for the reaction of *ortho*-substituted cinnamaldehyde **1a** (Table 1). To our delight, by using quinine **4a** and **5a** as catalysts, product **3a** was obtained in 54% yield, 20 : 1 dr and 92% ee (entry 1), with the desired relative stereochemistry. A small decrease in enantioselectivity (86% ee) was observed when cinchonidine **4b** was used (entry 2), indicating a possible role for the methoxy group of quinine **4a** in the stereo-defining step. Interestingly, the pseudoenantiomers **4c** and **4d** afforded ***ent*-3a** in comparable enantioselectivities (entries 3, 4), which allowed us to obtain both enantiomers of **3** with high enantioselectivity. As an attempt to achieve diastereodivergence in the formation of **3a**,^32^ the chiral NHC-catalysts **5b** and **5c** were tested (entries 5, 6). Both catalysts formed **3a** as the major product with 5 : 1 and 20 : 1 dr, respectively, which implies that catalyst control of **5b** mismatches with the substrate control, while for **5c** we observe the match case. However, catalyst **5b** was not selective enough to override the substrate bias of intermediate **2** as the major diastereoisomer remains the same. As a result, Brønsted base **4a** and achiral NHC-catalyst **5a** were chosen to investigate the scope of the reaction.

**Table 1:** Screening of Brønsted base/NHC-catalysis for the double cyclization of **1a**

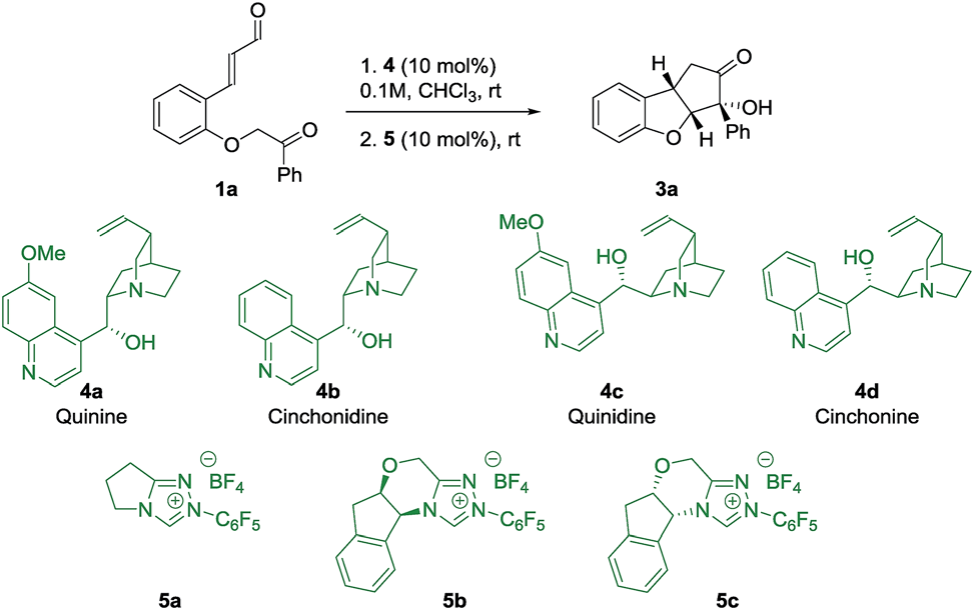							
Entry^*a*^	Base	NHC	*t*_1_ ^*b*^ (h)	*t*_2_ (h)	dr^*c*^	Yield (%)	ee^*d*^ (%)
1	**4a**	**5a**	24	24	20 : 1	54	92
2	**4b**	**5a**	24	24	12 : 1	48	86
3	**4c**	**5a**	24	24	19 : 1	54	–93
4	**4d**	**5a**	24	24	16 : 1	53	–89
5	**4a**	**5b**	20	16	5 : 1	44	95
6	**4a**	**5c**	20	12	20 : 1	60	93

^*a*^Reactions were performed on a 0.1 mmol scale.

^*b*^Determined by ^1^H NMR of the crude reaction mixture; *t*_1_ refers to the reaction time for the first step, while *t*_2_ refers to the second reaction step.

^*c*^Diastereomeric ratio was determined by ^1^H NMR analysis of the crude reaction mixture.

^*d*^Enantiomeric excess was determined by UPC^2^.

Performing the reaction on 0.25 mmol scale provided **3a** in 62% yield, 19 : 1 dr and 93% ee (Scheme 2). Substrates containing aromatic halides all reacted in a satisfactory way, giving **3b** in 58% yield, 12 : 1 dr and 95% ee (F), **3c** in 67% yield, 14 : 1 dr and 95% ee (Cl) and **3d** in 66% yield, 11 : 1 dr and 95% ee (Br). When a substrate bearing a cyano group (**1e**) was used, product **3e** was isolated in 45% yield, 9 : 1 dr and 87% ee. The lower enantioselectivity observed for **3e** possibly results from the increased acidity of the carbonyl α-proton due to the presence of the electron-withdrawing substituent. Substrates having the electron-donating methoxy substituent in positions 5–7 (**3f–h**), all react providing the desired products in moderate yields (68–75% yield for each cyclization step) and high diastereo- and enantioselectivity.

The reaction also showed tolerance for variation of the substituents in the aryl side-chain in **1** (Scheme 3). The results in Scheme 3 show a similar trend to the scope in Scheme 2 for the halogenated substrates. While showing comparable enantioselectivities, the chlorinated entry showed a higher diastereoselectivity than the fluorinated and brominated counterparts. The fluorinated product **3i** was obtained in 48% yield, 7 : 1 dr and 93% ee, the chlorinated **3j** in 54% yield, 12 : 1 dr, 93% ee and brominated **3k** in 48% yield, 7 : 1 dr, 96% ee. Electron-donating functionalities in various positions in the aryl side-chain also reacted smoothly. The *para*-, *meta*- and *ortho*-substituted products **3l–n** were formed in 42% yield, 11 : 1 dr and 94% ee (**3l**), 55% yield, 15 : 1 dr and 95% ee (**3m**) and 44% yield, 8 : 1 dr and 94% ee (**3n**). Substrate **1o**, containing a 2-naphthyl group gave **3o** in 51% yield, 13 : 1 dr and 94% ee. The presence of a 2-thienyl side-chain led to lower yield and selectivity compared to the other substrates and **3p** was formed in 29% yield, 6 : 1 dr and 89% ee. Substrates bearing ethyl, isopropyl or *tert*-butyl groups in the ketone side-chains were also tested; however, no reactivity was observed under the optimal reaction conditions. This limitation to the scope may result from the lower acidity of the α-proton from alkyl ketones, when compared to aromatic ones.^33^ In an attempt to achieve conversion for the alkyl ketones, the reaction was also carried out at 40 °C. After a very long reaction time (>2 weeks), intermediate **2** could be observed for the ethyl- and isopropyl substrates with >80% conversion, albeit in low diastereoselectivity. However, a complex reaction mixture was formed after adding the NHC-precatalyst **5a**.^27^

The reaction has also been tested under synergistic reaction conditions, where both catalysts were added at the same time. No reactivity was observed, compared to the one-pot reaction conditions. This lack of synergistic activity might be due to the Brønsted base deprotonating the NHC-precatalyst, thereby not being able to catalyze the first step.

### Synthetic elaborations

**Scheme 4:**
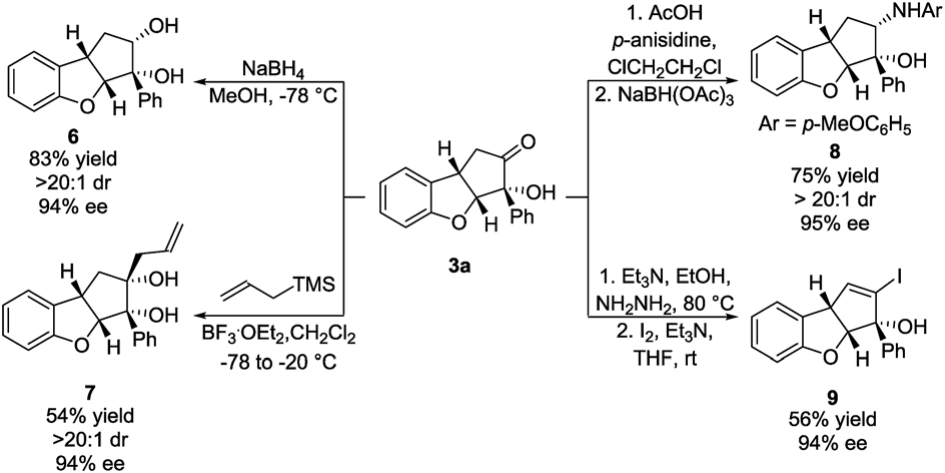
Functionalizations of **3a**: carbonyl reduction (top left), allylation (bottom left), reductive amination (top right) and Barton's synthesis of vinyl iodide (bottom right). Reactions were performed on a 0.25 mmol scale. Diastereomeric ratio was determined by ^1^H NMR analysis of the crude reaction mixture. Enantiomeric excess was determined by UPC^2^.

To demonstrate the synthetic applicability of the Brønsted base/NHC-catalysed double cyclization process, the synthesis of **3a** was scaled up and the product was subjected to various transformations. 4 mmol of **1a** (1.06 g) reacted under our optimal reaction conditions, forming **3a** in 53% yield, 11 : 1 dr and 93% ee. A sodium borohydride reduction of **3a** afforded the *cis*-diol **6** in 83% yield, >20 : 1 dr and 94% ee (Scheme 4, top left). Allylation using allyltrimethylsilane, mediated by boron trifluoride, provided the *cis*-diol **7** in 54% yield, >20 : 1 dr and 94% ee (Scheme 4, bottom left). A reductive amination using *p*-anisidine generated amine **8** in 75% yield, >20 : 1 dr and 95% ee (Scheme 4, top right). In all three cases, the nucleophilic attack happened exclusively in the exo face of the cyclopenta[*b*]benzofuran bicyclic ring system. Such a selectivity is remarkable as it was obtained despite the presence of a bulky phenyl side chain in the exo face. The formation of a hydrazone, followed by iodination and elimination (Barton's vinyl iodide synthesis)^34^ allowed us to obtain vinyl iodide **9** in 56% yield and 94% ee (Scheme 4, bottom right). These transformations demonstrate diversification of the substitution pattern of the cyclopenta[*b*]benzofuran scaffold without any loss in enantiopurity.

### Catalyst structure–activity relationship studies

In an attempt to obtain insight into the reaction mechanism, a structure–activity relationship study of the Brønsted-base catalyst was performed (Table 2). Hydrogenation of the catalyst double bond (entries 1, 2) led to the formation of **3a** in stereoselectivity comparable to using quinine **4a** and quinidine **4c** (entries 1 and 3), indicating that the selectivity of the reaction is not sensitive to small changes in the vinyl side-chain of the Brønsted-base catalyst. However, by using the quinine derivative **4g** as catalyst, bearing a phenol functional group, a dramatic change in the stereoselectivity was observed, as a reversion in enantioselectivity took place and ***ent*-3a** was formed in 22% ee. This change in selectivity might result from a scenario where multiple hydrogen-bonding donor sites at the catalyst are interacting with the deprotonated substrate in the transition state. If the phenolic and alcoholic hydroxy groups each stabilize preferably a transition state that leads to opposite enantiomers, the competition between this enantiodivergent pathways would be expected to lead to a diminished stereoselectivity. The observed reversion of enantioselectivity might originate from the stronger hydrogen bond of the phenolic hydroxy group compared to the aliphatic hydroxyl group.

**Table 2:** Structure–activity relationship studies of the Brønsted base catalyst

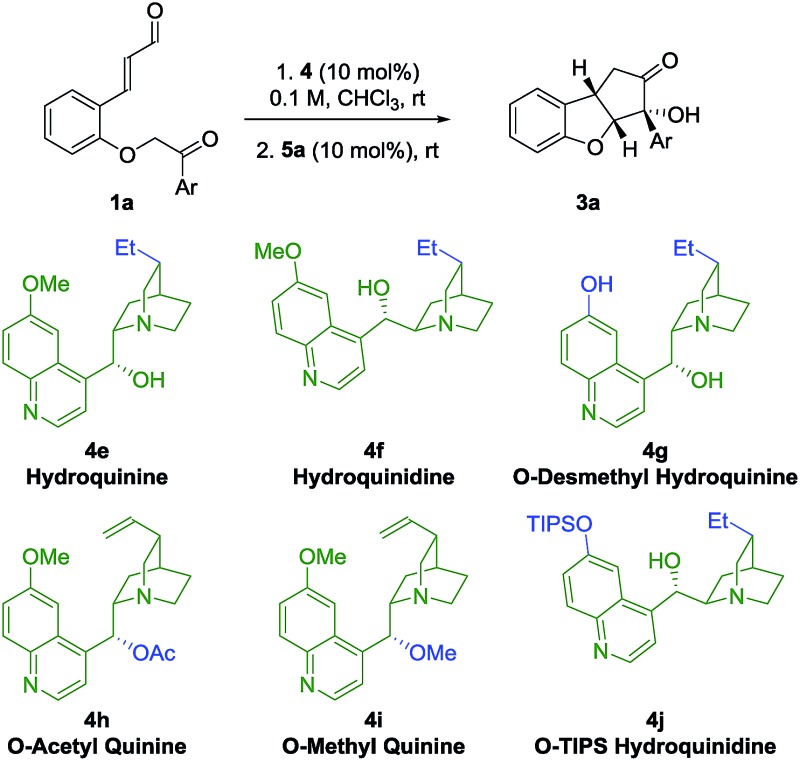						
Entry^*a*^	Base	*t*_1_ ^*b*^ (h)	*t*_2_ ^*b*^ (h)	dr^*c*^	Yield (%)	ee^*d*^ (%)
1	**4e**	18	18	20 : 1	66	92
2	**4f**	18	18	12 : 1	73	–94
3	**4g**	18	18	1 : 1	26	–22
4	**4h**	—	—	—	—	—
5	**4i**	>72	—	—	—	—
6	**4j**	20	32	8 : 1	49	–96

^*a*^Reactions were performed on a 0.1 mmol scale.

^*b*^Determined by ^1^H NMR of the crude reaction mixture; *t*_1_ refers to the reaction time for the first step, while *t*_2_ refers to the second reaction step.

^*c*^Diastereomeric ratio was determined by ^1^H NMR analysis of the crude reaction mixture.

^*d*^Enantiomeric excess was determined by UPC^2^.

Upon acetylation of the hydroxy group of quinine (**4h**), no catalytic activity is observed (Table 2, entry 4). When the hydroxy group of quinine is methylated (**4i**), only traces of intermediate **2a** are observed after 72 h (entry 5). These results showcase the importance of the hydroxy group of the quinine catalyst **4a** for the catalytic activity. When a cinchonidine derivative bearing a much more sterically demanding group (*O*-triisopropylsilyl, OTIPS, **4j**) is used (entry 6), a minor increase in enantioselectivity is observed (–96% ee). Together with the fact that the absence of the methoxy group (cinchonidine **4b**, Table 1, entry 2) reduces the enantioselectivity, these results indicate that a substituent in this position might contribute to some type of steric shielding in the transition state.

**Scheme 5:**
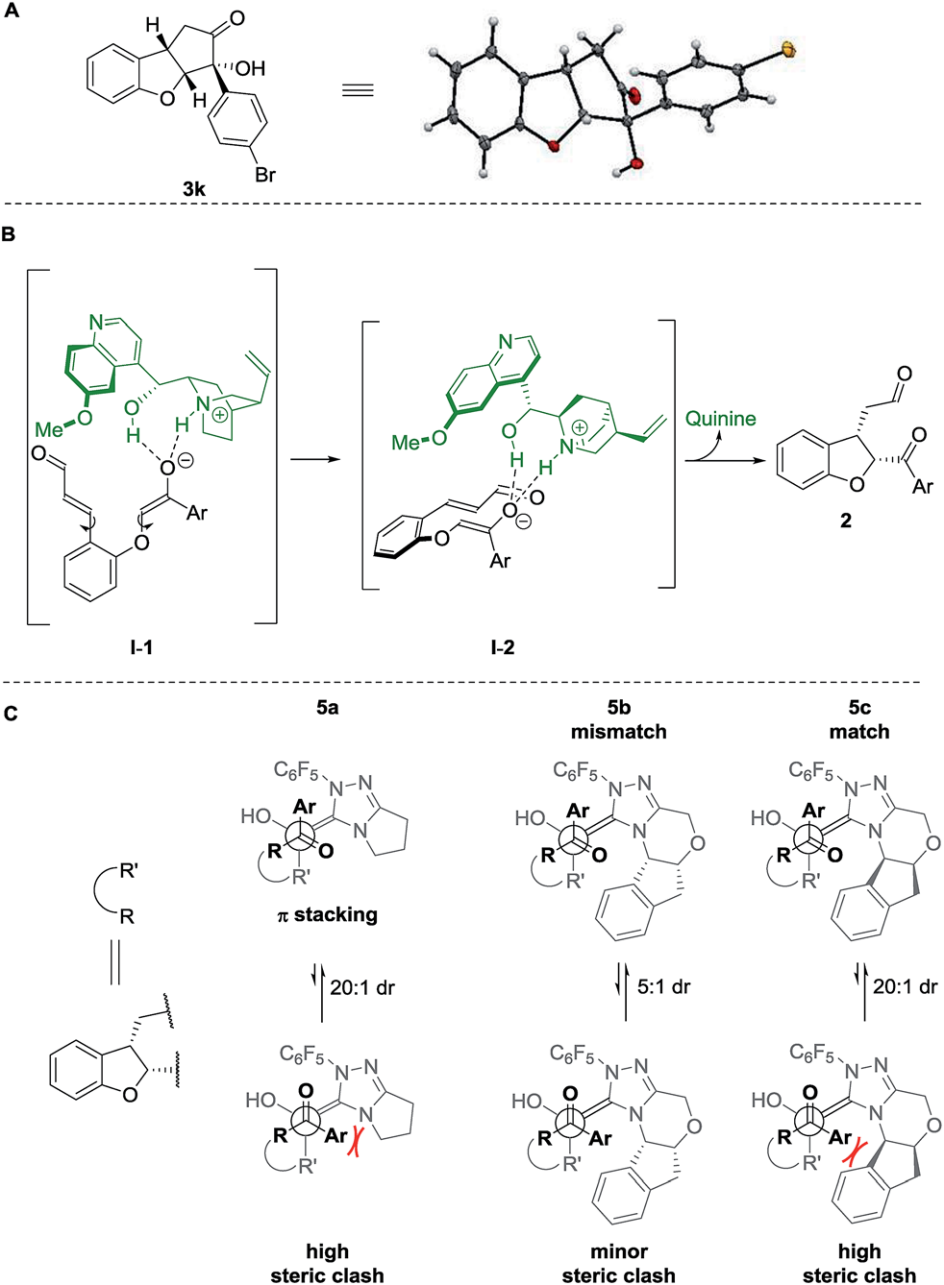
(A) X-ray structure of **3k**. Stereochemical model for (B) proposed Michael addition step, (C) benzoin condensation.

The absolute configuration of cyclopenta[*b*]benzofurans was unambiguously assigned by X-ray analysis of crystals of **3k** (Scheme 5A). This allowed us to propose a stereochemical model for the Michael addition based on the structure activity relationship studies of the Brønsted-base catalyst (Scheme 5B). In the first step, quinine **4a** acts as a base and deprotonates the α-position to the ketone forming the enolate and a chiral ammonium ion. The chiral ammonium ion and the enolate are proposed to generate intermediate **I-1** by hydrogen bonding interactions between the enolate with the hydroxy and ammonium groups of the catalyst. The proposal in **I-1** is supported by the results in Table 2. For the formation of the observed stereochemistry in **2**, the 6-methoxyquinoline shields the upper face of the substrate and forces the α,β-unsaturated aldehyde moiety to rotate and point down, leading to the arrangement in **I-2**. The stereochemical outcome of the Michael addition would arise from the cooperation between steric shielding over the β-position of the aldehyde and hydrogen bonding directing effects over the enolate.

**Scheme 6:**
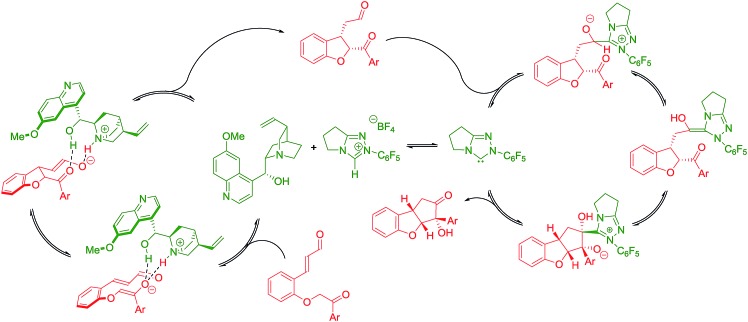
Proposed catalytic cycles.

Newman projections for the proposed stereochemical model for the benzoin condensation are depicted (Scheme 5C), taking into account the expected steric repulsions and π-stacking interactions between the aryl side-chain of the substrate and the perfluoroaryl group of the NHC-catalyst.^35^ The pathways that would minimize the steric repulsions and maximize the π-stacking interactions are expected to lead the observed products (top). In the case of the disfavored pathways (bottom), NHC-catalyst **5b** is causing lower steric repulsion compared to **5a** and **5c** which is supported by the results in Table 1. The mechanistic proposal shown in Scheme 6, outlines the cycle-specific nature of the two catalytic processes and the double role of quinine **4a**: as catalyst for the first cycle, and deprotonating the precursor of the NHC-catalyst **5a**.

### Contiguous tetrasubstituted tertiary stereocenters

Virtually all flavaglines and their biologically active analogs contain an aryl side-chain at the oxygenated carbon of the ring fusion. Under the double cyclization conditions, this would lead to a cyclopenta[*b*]benzofuran bearing two contiguous tetrasubstituted tertiary stereocenters.^36^ Unfortunately, under the optimal reaction conditions, the introduction of an additional phenyl group in the *ortho*-substituted cinnamaldehyde that would give the desired aryl side-chain pattern, provided virtually no diastereoselectivity in the second step (Table 3, entry 1). When cinchonidine **4b** was used as catalyst, a small improvement in diastereoselectivity was observed for the Michael addition step (entry 2). Surprisingly, by using the chiral NHC-catalyst **5b**, product ***epi*-3q** was obtained in 63% yield, 1 : 2 dr and 96% ee. We were pleased to find that the application of NHC-catalyst **5c** in combination with **4b** led to the formation of product **3q** in 65% yield, 6 : 1 dr and 92% ee. This demonstrates that the system tolerates the introduction of an additional aryl side-chain forming the product with the desired stereochemistry and is a proof of principle for achieving diastereodivergence.

**Table 3:** Formation of contiguous tetrasubstituted tertiary stereocenters

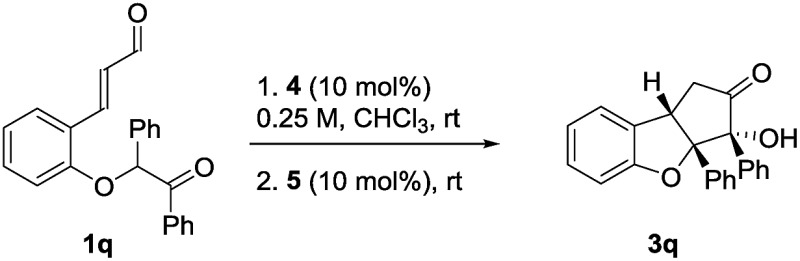								
Entry^*a*^	Base	NHC	*t*_1_ ^*b*^ (h)	*t*_2_ ^*b*^ (h)	dr_1_^*c*^	dr_2_^*c*^	Yield (%)	ee^*d*^ (%)
1	**4a**	**5a**	36	24	2.5 : 1	1 : 1	—	—
2	**4b**	**5a**	16	24	3.5 : 1	1 : 1	—	—
3	**4b**	**5b**	16	48	3.5 : 1	1 : 2	63	96
4	**4b**	**5c**	16	24	3.5 : 1	6 : 1	65	92

^*a*^Reactions were performed on a 0.1 mmol scale.

^*b*^Determined by ^1^H NMR of the crude reaction mixture; *t*_1_ refers to the reaction time for the first step, while *t*_2_ refers to the second reaction step.

^*c*^Diastereomeric ratio was determined by ^1^H NMR analysis of the crude reaction mixture; dr_1_ refers to the first step, while dr_2_ to the second step.

^*d*^Enantiomeric excess was determined by UPC^2^.

## Conclusions

A concise one-pot approach for the cyclopenta[*b*]benzofuran scaffold was developed. This was based on an intramolecular double cyclization through a cycle-specific enantioselective Michael addition followed by a benzoin condensation *via* Brønsted-base and NHC-combined catalysis. The reaction scope was demonstrated for 17 representative examples, forming products in moderate to good yields, with up to 19 : 1 dr and 96% ee. Both electron-donating and electron-withdrawing substituents were tolerated, in various substitution patterns. Several transformations were performed, demonstrating the synthetic utility of the products. Insights into the activation mode of the Brønsted-base catalyst were achieved through a structure activity relationship study, and stereochemical models were proposed based on the absolute configuration. A proof of principle for the possibility of achieving diastereodivergence by using chiral NHC-catalysts was also performed, leading to a cyclopenta[*b*]benzofuran bearing two contiguous tetrasubstituted tertiary stereocenters.

## Conflicts of interest

There are no conflicts to declare.

## Supplementary Material

Supplementary information

Crystal structure data
