# Supplementary material for: Enantioselective synthesis of cyclopenta[b]benzofurans via an organocatalytic intramolecular double cyclization
Source: Chem Sci. 2017 Oct 2;8(12):8086–93. doi: 10.1039/c7sc03006a (PMC5855134; doi:10.1039/c7sc03006a)

Electronic Supplementary Material (ESI) for Chemical Science.
This journal is © The Royal Society of Chemistry 2017

Enantioselective Synthesis of Cyclopenta[*b*]benzofurans via an Organocatalytic Intramolecular Double Cyclization

Bruno Matos Paz, Yang Li, Mathias Kirk Thøgensen and Karl Anker Jørgensen*

Department of Chemistry, Aarhus University
DK-8000 Aarhus C, Denmark
Fax (45) 8919 6199, e-mail: kaj@chem.au.dk

Contents

1. General methods S2
2. Synthesis of aldehydes **1a**-**t** S3
3. The asymmetric synthesis of cyclopenta[b]benzofurans S10
    3.1. Optimization S10
    3.2. General procedure for organocatalytic synthesis of **3a-p** S13
    3.3. Results and characterization S13
    3.4 Procedure for the synthesis of **3a** at 4.0 mmol scale S18
4. Transformations S19
    4.1 Reduction S19
    4.2 Allylation S19
    4.3 Reductive amination S20
    4.4 Barton's vinyl iodide synthesis S20
5. Diastereodivergence S22
6. Attempts to react with alkyl substituted substrates S23
7. Determination of the absolute configuration S24
8. NMR spectra S25
9. UPC^2 traces S69
10. IR spectra S90

1. General methods

NMR spectra were acquired on a Bruker AVANCE III HD spectrometer running at 400 MHz for ^{1}H, 100 MHz for ^{13}C and 162 MHz for ^{31}P. Chemical shifts (δ) are reported in ppm relative to residual solvent signals ($CHCl_3$, 7.26 ppm for ^{1}H NMR, $CDCl_3$, 77.16 ppm for ^{13}C NMR). The following abbreviations are used to indicate the multiplicity in NMR spectra: s, singlet; d, doublet; t, triplet; q, quartet; m, multiplet; bs, broad signal; dd, double doublet; ddd, double double doublet. ^{13}C spectra were acquired in broad band decoupled mode. For characterization of isomeric mixtures * denotes minor isomer, # denotes overlap of signals of both isomers, whereas no sign denotes signal of major isomer. The number of protons given in parentheses is the sum over both isomers. Mass spectra were recorded on a Bruker Maxis Impact mass spectrometer using electronspray (ES^{+}) ionization (referenced to the mass of the charged species). Dry solvents were obtained from a MBraun MB SPS-800 solvent purification system. Analytical thin layer chromatography (TLC) was performed using pre-coated aluminium-backed plates (Merck Kieselgel 60 F254) and visualized by UV radiation, $KMnO_4$ or p-anisaldehyde stains. For flash chromatography (FC) silica gel (Silica gel 60, 230- 400 mesh, Sigma-Aldrich) or Iatrobeads 6RS - 8060 were used. Optical rotations were measured on a PerkinElmer 241 polarimeter, $[\alpha]_D$ values are given in $deg \cdot cm^{3} \cdot g^{-1} \cdot dm^{-1}$; concentration c in g $(100\ mL)^{-1}$. The diastereomeric ratio (dr) of products was evaluated by ^{1}H NMR analysis of the crude mixture. The enantiomeric excess (ee) of products was determined by Ultraperformance Convergence Chromtagraphy (Waters ACQUITY UPC^{2}) using Daicel Chiralpak IA, IB, IC, ID columns as chiral stationary phases. Racemates for UPC^{2} analysis were made by mixing **3** and ***ent*-3**, formed from quinine **3a** or quinidine **3b** as catalysts. For the reactions in which the dr was poor and the diastereoisomers could not be well separated by FC, the diastereoisomeric mixture was characterized by means of chiral stationary phase UPC^{2}, in which all four peaks of both diastereoisomers were present; the correct correspondence of the peaks was confirmed by the juxtaposition of the UV spectra, recorded by the PDA detector of the UPC^{2} system. Unless otherwise noted, analytical grade solvents and commercially available reagents were used without further purification.

2. Synthesis of aldehydes 1a-t

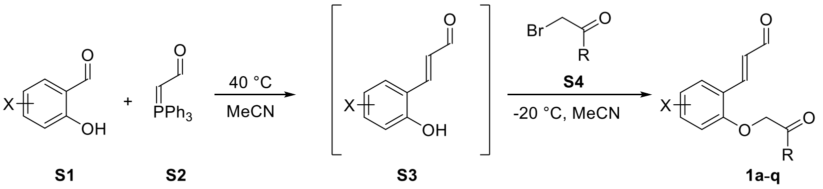

In a flame dried flask, the stabilized ylide **S2** (1.1 eq, 5.5 mmol, 1.67 g) was dissolved in dry MeCN (20 mL). Afterwards, the respective 2-hydroxybenzaldehyde **S1** (1.0 eq, 5 mmol) was added in one portion and the reaction mixture was heated to 40 °C for 16-24 h. After full conversion, the respective α-bromo-ketone **S4** (1.2 eq, 6 mmol) was added and the reaction mixture was cooled to -20 °C. After stirring for 5 min, oven dried K_2CO_3 (1.2 eq, 6 mmol, 829 mg) was added in one portion. The reaction mixture was then stirred at -20 °C until full conversion, filtered over cotton, concentrated *in vacuo* and purified by FC over silica gel.

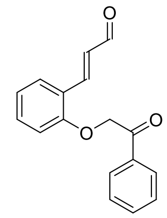

1a

Following the general procedure, the second step was over in 4 d. The aldehyde **1a** was purified by FC on silica (10:40:50 to 20:40:40 EtOAc/PhMe/pentane). The product was then recrystallized from CH_2Cl_2/pentane. The aldehyde **1a** was isolated in 51% yield (679 mg) as a white solid.

^{1}H NMR (400 MHz, CDCl$_3$): δ 9.70 (d, *J* = 7.8 Hz, 1H), 8.03 – 7.93 (m, 2H), 7.69 – 7.58 (m, 2H), 7.53 (t, *J* = 7.8 Hz, 2H), 7.37 (ddd, *J* = 8.8, 7.5, 1.7 Hz, 2H), 7.05 (t, *J* = 7.5 Hz, 1H), 6.87 – 6.78 (m, 2H), 5.42 (s, 2H).

^{13}C NMR (100 MHz, CDCl$_3$): δ 194.7, 193.7, 156.7, 147.9, 134.4, 134.3, 132.7, 129.5, 129.1, 129.0, 128.2 (2C), 123.8, 122.0, 112.6, 71.0.

HRMS (ESI+) calculated for: $[C_{17}H_{14}O_3+Na]^+$ 289.0835; found: 289.0840.

IR, ṽ (cm^{-1}): C=O 1618, 1596.

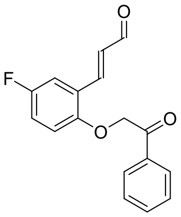

1b

Following the general procedure, the second step was over in 1 d. The aldehyde **1b** was purified by FC on silica (10:40:50 to 20:40:40 EtOAc/PhMe/pentane). The product was then recrystallized from CH_2Cl_2/pentane. The aldehyde **1b** was isolated in 57% yield (810 mg) as a white solid.

^{1}H NMR (400 MHz, CDCl$_3$): δ 9.71 (dd, *J* = 7.7, 1.2 Hz, 1H), 8.01 – 7.96 (m, 2H), 7.93 (d, *J* = 16.1, 1H), 7.66 (t, *J* = 7.4 Hz, 2H), 7.53 (t, *J* = 7.7 Hz, 2H), 7.29 (ddd, *J* = 8.9, 3.2, 1.3 Hz, 1H), 7.10 – 7.03 (m, 1H), 6.82 – 6.72 (m, 2H), 5.41 (s, 2H).

^{13}C NMR (100 MHz, CDCl$_3$): δ 194.2, 193.5, 157.5 (d, *J* = 241.2 Hz), 153.0 (d, *J* = 2.1 Hz), 146.3 (d, *J* = 2.3 Hz), 134.4, 134.3, 130.2, 129.2 (2C), 128.1 (2C), 125.1 (d, *J* = 7.5 Hz), 118.9 (d, *J* = 23.7 Hz), 114.7 (d, *J* = 23.5Hz), 114.1 (d, *J* = 8.1Hz), 71.58.

^{19}F NMR (376 MHz, CDCl$_3$): δ -121.41

HRMS (ESI+) calculated for: $[C_{17}H_{13}O_3F+Na]^+$ 307.0741; found: 307.0745.

IR, ṽ (cm^{-1}): C=O 1704, 1670.

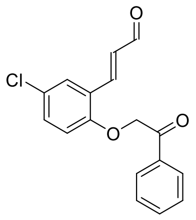

1c

Following the general procedure, the second step was over in 3 d. The aldehyde **1c** was purified by FC on silica (10:40:50 to 15:40:45 EtOAc/PhMe/pentane). The product was then recrystallized from CH_2Cl_2/pentane. The aldehyde **1c** was isolated in 52% yield (782 mg) as a white solid.

^{1}H NMR (400 MHz, $CDCl_3$): δ 9.71 (d, *J* = 7.7 Hz, 1H), 8.00 – 7.94 (m, 2H), 7.88 (d, *J* = 16.1 Hz, 1H), 7.69 – 7.62 (m, 1H), 7.58 – 7.49 (m, 3H), 7.30 (dd, *J* = 8.9, 2.6 Hz, 1H), 6.84 – 6.74 (m, 2H), 5.42 (s, 2H).

^{13}C NMR (100 MHz, $CDCl_3$): δ 194.2, 193.2, 155.2, 146.1, 134.5, 134.2, 132.0, 130.4, 129.2 (2C), 128.4, 128.1 (2C), 127.2, 125.2, 114.0, 71.1.

HRMS (ESI+) calculated for: $[C_{17}H_{13}O_3Cl+Na]^+$ 323.0445; found: 323.0444.

IR, ṽ (cm^{-1}): C=O 1705, 1670.

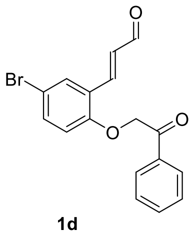

1d

Following the general procedure, the second step was over in 5 d. The aldehyde **1c** was purified by FC on silica (10:40:50 EtOAc/PhMe/pentane). The product was then recrystallized from CH_2Cl_2/pentane. The aldehyde **1d** was isolated in 58% yield (1.00 g) as a white solid.

^{1}H NMR (400 MHz, $CDCl_3$): δ 9.69 (d, *J* = 7.7 Hz, 1H), 7.97 (d, *J* = 7.4 Hz, 2H), 7.86 (d, *J* = 16.1 Hz, 1H), 7.69 (d, *J* = 2.4 Hz, 1H), 7.66 (t, *J* = 7.5 Hz, 1H), 7.53 (t, *J* = 7.7 Hz, 2H), 7.43 (dd, *J* = 8.8, 2.4 Hz, 1H), 6.79 (dd, *J* = 16.1, 7.7 Hz, 1H), 6.71 (d, *J* = 8.8 Hz, 1H), 5.42 (s, 2H).

^{13}C NMR (100 MHz, $CDCl_3$): δ 194.2, 193.2, 155.7, 146.0, 134.9, 134.5, 134.2, 131.4, 130.4, 129.2 (2C), 128.1 (2C), 125.7, 114.4, 114.3, 71.0.

HRMS (ESI+) calculated for: $[C_{17}H_{13}BrO_3+Na]^+$: 366.9940; found: 366.9940.

IR, ṽ (cm^{-1}): C=O 1703, 1668.

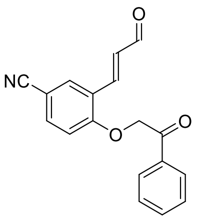

1e

Following the general procedure, the second step was over in 40 h. The aldehyde **1e** was purified by FC on silica (EtOAc/PhMe/pentane 10:40:50 to 40:30:30) and isolated in 32% yield (466 mg) as a white solid.

^{1}H NMR (400 MHz, $CDCl_3$): δ 9.73 (d, J = 7.6 Hz, 1H), 8.01 – 7.94 (m, 2H), 7.89 – 7.81 (m, 2H), 7.72 – 7.65 (m, 1H), 7.62 (dd, J = 8.7, 2.1 Hz, 1H), 7.55 (t, J = 7.8 Hz, 2H), 6.90 – 6.81 (m, 2H), 5.54 (s, 2H).

^{13}C NMR (100 MHz, $CDCl_3$): δ 193.9, 192.2, 159.4, 144.9, 135.8, 134.7, 133.9, 133.0, 131.3, 129.3, 128.0, 124.9, 118.2, 113.2, 105.8, 70.7.

HRMS (ESI+) calculated for: $[C_{18}H_{13}NO_3+Na]^+$ 314.0788; found: 314.0793.

IR, ṽ (cm^{-1}): C≡N 2229; C=O 1697, 1665.

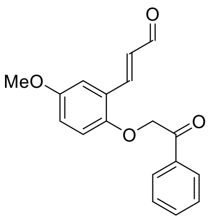

1f

Following the general procedure, the second step was over in 3 d. The aldehyde **1f** was purified by FC on silica (10:40:50 to 20:40:40 EtOAc/PhMe/pentane). The product was then recrystallized from CH_2Cl_2/pentane. The aldehyde **1f** was isolated in 73% yield (1.08 g) as a yellow solid.

^{1}H NMR (400 MHz, $CDCl_3$): δ 9.70 (d, *J* = 7.8 Hz, 1H), 8.01 – 7.93 (m, 3H), 7.67 – 7.61 (m, 1H), 7.52 (t, *J* = 7.8 Hz, 2H), 7.11 (d, *J* = 3.0 Hz, 1H), 6.93 (dd, *J* = 9.0, 3.0 Hz, 1H), 6.83 – 6.73 (m, 2H), 5.36 (s, 2H), 3.80 (s, 3H).

^{13}C NMR (100 MHz, CDCl$_3$): δ 194.5, 194.1, 154.4, 151.2, 147.5, 134.5, 134.3, 129.5, 129.1 (2C), 128.1 (2C), 124.5, 118.5, 114.3, 112.8, 71.9, 55.9.
HRMS (ESI+) calculated for: [$C_{18}H_{16}O_4$+Na]$^+$ 319.0941; found: 319.0943.
IR, ṽ (cm^{-1}): C=O 1687, 1678.

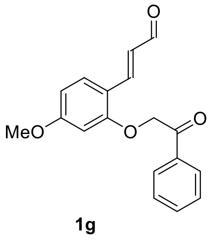

1g

Following the general procedure, the second step was over in 4 d. The aldehyde **1g** was purified by FC on silica (10:40:50 to 20:40:40 EtOAc/PhMe/pentane) and isolated in 49% yield (726 mg) as a yellow solid.
^{1}H NMR (400 MHz, CDCl$_3$): δ 9.64 (d, *J* = 7.9 Hz, 1H), 8.02 – 7.96 (m, 2H), 7.86 (d, *J* = 16.0 Hz, 1H), 7.68 – 7.61 (m, 1H), 7.58 – 7.48 (m, 3H), 6.75 (dd, *J* = 16.0, 7.9 Hz, 1H), 6.59 (dd, *J* = 8.7, 2.3Hz, 1H), 6.36 (d, *J* = 2.3 Hz, 1H), 5.38 (s, 2H), 3.82 (s, 3H).
^{13}C NMR (100 MHz, CDCl$_3$): δ 194.7, 193.5, 163.6, 158.2, 148.0, 134.4, 134.3, 130.5, 129.1 (2C), 128.2 (2C), 127.3, 116.9, 106.6, 99.9, 71.0, 55.8.
HRMS (ESI+) calculated for: [$C_{18}H_{16}O_4$+Na]$^+$ 319.0941; found: 319.0948.
IR, ṽ (cm^{-1}): C=O 1659, 1594.

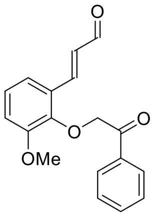

1h

Following the general procedure, the second step was over in 4 d. The aldehyde **1h** was purified by FC on silica (10:40:50 to 15:40:45 EtOAc/PhMe/pentane) and isolated in 82% yield (1.21 g) as a white solid.
^{1}H NMR (400 MHz, CDCl$_3$): δ 9.68 (d, *J* = 7.8 Hz, 1H), 8.15 (d, *J* = 16.2 Hz, 1H), 7.99 – 7.92 (m, 2H), 7.65 – 7.59 (m, 1H), 7.50 (dd, *J* = 8.4, 7.1 Hz, 2H), 7.23 (dd, *J* = 8.0, 1.4 Hz, 1H), 7.12 (t, *J* = 8.0 Hz, 1H), 7.00 (dd, *J* = 8.1, 1.5 Hz, 1H), 6.70 (dd, *J* = 16.1, 7.8 Hz, 1H), 5.42 (s, 2H), 3.85 (s, 3H).
^{13}C NMR (100 MHz, CDCl$_3$): δ 194.7, 194.6, 152.3, 147.8, 146.7, 134.8, 133.9, 129.7, 129.0 (2C), 128.3, 128.1 (2C), 124.6, 119.2, 115.1, 75.2, 56.1.
HRMS (ESI+) calculated for: [$C_{18}H_{16}O_4$+Na]$^+$ 319.0941; found: 319.0945.
IR, ṽ (cm^{-1}): C=O 1670, 1706.

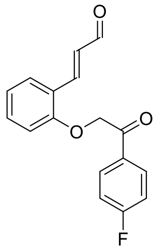

1i

Following the general procedure, the second step was over in 4 d. The aldehyde **1i** was purified by FC on silica (10:40:50 to 20:40:40 EtOAc/PhMe/pentane) and isolated in 67% yield (952 mg) as a white solid.
^{1}H NMR (400 MHz, CDCl$_3$): δ 9.71 (d, *J* = 7.8 Hz, 1H), 8.08 – 7.99 (m, 2H), 7.95 (d, *J* = 16.0 Hz, 1H), 7.61 (dd, *J* = 7.8, 1.7 Hz, 1H), 7.37 (ddd, *J* = 8.7, 7.5, 1.7 Hz, 1H), 7.23 – 7.15 (m, 2H), 7.06 (t, *J* = 7.5 Hz, 1H), 6.88 – 6.79 (m, 2H), 5.37 (s, 2H).
^{13}C NMR (100 MHz, CDCl$_3$): δ 194.6, 192.3, 166.4 (d, *J* = 256.50 Hz), 156.6, 147.7, 132.7, 131.0 (d, *J* = 9.56 Hz) (2C), 130.8 (d, *J* = 3.29 Hz), 129.6, 129.1, 123.8, 122.1, 116.4 (d, *J* = 22.02 Hz) (2C), 112.5, 70.9.
^{19}F NMR (376 MHz, CDCl$_3$): δ -102.64
HRMS (ESI+) calculated for: [$C_{17}H_{13}O_3F$+Na]$^+$ 307.0741; found: 307.0740.
IR, ṽ (cm^{-1}): C=O 1678, 1594.

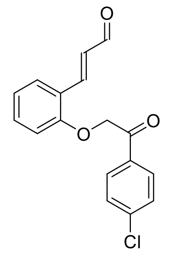

1j

Following the general procedure, the second step was over in 3 d. The aldehyde **1j** was purified by FC on silica (10:40:50 to 15:40:45 EtOAc/PhMe/pentane). The product was then recrystallized from CH_2Cl_2/pentane. The aldehyde **1f** was isolated in 66% yield (992 mg) as a white solid.

^{1}H NMR (400 MHz, $CDCl_3$): δ 9.71 (d, *J* = 7.8 Hz, 1H), 7.98 – 7.90 (m, 3H), 7.61 (dd, *J* = 7.8, 1.7 Hz, 1H), 7.53 – 7.46 (m, 2H), 7.37 (ddd, *J* = 8.7, 7.4, 1.7 Hz, 1H), 7.05 (t, *J* = 15.1 Hz, 1H) (m, 1H), 6.87 – 6.79 (m, 2H), 5.37 (s, 2H).

^{13}C NMR (100 MHz, $CDCl_3$): δ 194.6, 192.7, 156.6, 147.7, 140.9, 132.7 (2C), 129.7, 129.6 (2C), 129.5 (2C), 129.1, 123.8, 122.2, 112.5, 71.0.

HRMS (ESI+) calculated for: $[C_{17}H_{13}O_3Cl+Na]^+$ 323.0445; found: 323.0447.

IR, ṽ (cm^{-1}): C=O 1703, 1658.

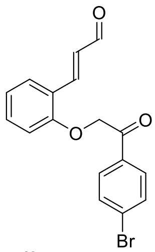

1k

Following the general procedure, the second step was over in 5 d. The aldehyde **1k** was purified by FC on silica (10:40:50 EtOAc/PhMe/pentane). The product was then recrystallized from CH_2Cl_2/pentane. The aldehyde **1k** was isolated in 50% yield (863 mg) as a white solid.

^{1}H NMR (400 MHz, $CDCl_3$): δ 9.70 (d, *J* = 7.7 Hz, 1H), 7.94 (d, *J* = 16.1 Hz, 1H), 7.86 (d, *J* = 8.0 Hz, 2H), 7.67 (d, *J* = 8.0 Hz, 2H), 7.60 (d, *J* = 7.5 Hz, 1H), 7.37 (t, *J* = 7.5 Hz, 1H), 7.05 (t, *J* = 7.4 Hz, 1H), 6.88 – 6.79 (m, 2H), 5.36 (s, 2H).

^{13}C NMR (100 MHz, $CDCl_3$): δ 194.6, 192.9, 156.6, 147.7, 133.1, 132.7, 132.5 (2C), 129.6 (2C), 129.6 (2C), 129.1, 123.8, 122.2, 112.5, 70.9.

HRMS (ESI+) calculated for: $[C_{17}H_{13}O_3Br+Na]^+$ 366.9940; found: 366.9940.

IR, ṽ (cm^{-1}): C=O 1703, 1664.

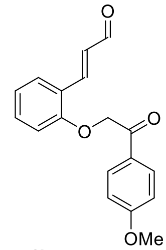

1l

Following the general procedure, the second step was over in 3 d. The aldehyde **1l** was purified by FC on silica (10:40:50 to 20:40:40 EtOAc/PhMe/pentane) and isolated in 76% (1.13 g) yield as a pale yellow solid.

^{1}H NMR (400 MHz, $CDCl_3$): δ 9.70 (d, *J* = 7.8 Hz, 1H), 8.02 – 7.94 (m, 3H), 7.60 (dd, *J* = 7.8, 1.7 Hz, 1H), 7.36 (ddd, *J* = 8.5, 7.4, 1.7 Hz, 1H), 7.04 (t, *J* = 7.6, 1H), 7.01 – 6.95 (m, 2H), 6.87 – 6.78 (m, 2H), 5.36 (s, 2H), 3.89 (s, 3H).

^{13}C NMR (100 MHz, $CDCl_3$): δ 194.7, 192.2, 164.4, 156.9, 147.9, 132.7, 130.6 (2C), 129.5, 128.9, 127.4, 123.7, 121.9, 114.3 (2C), 112.7, 70.9, 55.7.

HRMS (ESI+) calculated for: $[C_{18}H_{16}O_4+Na]^+$ 319.0941; found: 319.0945.

IR, ṽ (cm^{-1}): C=O 1670, 1595.

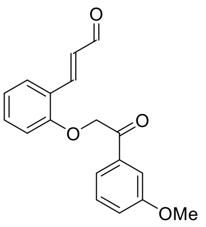

1m

Following the general procedure, the second step was over in 6 h. The aldehyde **1m** was purified by FC on silica (10:40:50 to 20:40:40 EtOAc/PhMe/pentane). The product was then recrystallized from CH_2Cl_2/pentane. The aldehyde **1m** was isolated in 33% yield (489 mg) as a white solid.

^{1}H NMR (400 MHz, $CDCl_3$): δ 9.71 (d, J = 7.8 Hz, 1H), 7.98 (d, J = 16.1 Hz, 1H), 7.61 (dd, J = 7.7, 1.7 Hz, 1H), 7.58 – 7.50 (m, 2H), 7.43 (t, J = 7.9 Hz, 1H), 7.37 (ddd, J = 8.7, 7.5, 1.7 Hz, 1H), 7.19 (ddd, J = 8.3, 2.8, 1.0 Hz, 1H), 7.05 (t, J = 7.3 Hz, 1H), 6.88 – 6.80 (m, 2H), 5.41 (s, 2H), 3.87 (s, 3H).

^{13}C NMR (100 MHz, $CDCl_3$): δ194.7, 193.5, 160.2, 156.7, 147.9, 135.6, 132.6, 130.1, 129.5, 129.0, 123.8, 122.0, 120.7, 120.5, 112.6, 112.5, 71.0, 55.7.

HRMS (ESI+) calculated for: $[C_{18}H_{16}O_4+Na]^+$ 319.0941; found: 319.0949.
IR, ṽ (cm^{-1}): C=O 1695, 1653.

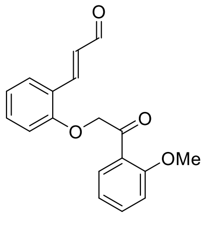

1n

Following the general procedure, the second step was over in 3 d. The aldehyde **1n** was purified by FC on silica (9:91 to 50:50 EtOAc/pentane). The product was then recrystallized from CH_2Cl_2/pentane. The aldehyde **1n** was isolated in 37% yield (548 mg) as a yellow solid.
^{1}H NMR (400 MHz, $CDCl_3$): δ 9.68 (d, J = 7.8 Hz, 1H), 7.99 – 7.91 (m, 2H), 7.61 – 7.53 (m, 2H), 7.37 – 7.32 (m, 1H), 7.11 – 7.06 (m, 1H), 7.05 – 6.99 (m, 2H), 6.86 – 6.75 (m, 2H), 5.35 (s, 2H), 3.96 (s, 3H).
^{13}C NMR (100 MHz, $CDCl_3$): δ 194.9, 194.8, 159.4, 157.2, 148.3, 135.1, 132.6, 131.2, 129.2, 128.8, 124.8, 123.5, 121.5, 121.4, 112.7, 111.6, 74.6, 55.8.
HRMS (ESI+) calculated for: $[C_{18}H_{16}O_4+Na]^+$ 319.0941; found: 319.0950.
IR, ṽ (cm^{-1}): C=O 1675, 1658.

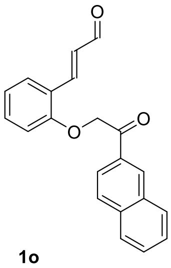

1o

Following the general procedure, the second step was over in 1 d. The aldehyde **1o** was purified by FC on silica (10:40:50 to 20:40:40 EtOAc/PhMe/pentane). The product was then recrystallized from CH_2Cl_2/pentane. The aldehyde **1o** was isolated in 34% yield (538 mg) as a yellow solid.
^{1}H NMR (400 MHz, $CDCl_3$): δ 9.68 (d, J = 7.8 Hz, 1H), 8.54 (s, 1H), 8.07 – 7.88 (m, 5H), 7.68 – 7.56 (m, 3H), 7.38 (ddd, J = 8.7, 7.4, 1.7 Hz, 1H), 7.05 (t, J = 7.5 Hz, 1H), 6.93 – 6.80 (m, 2H), 5.54 (s, 2H).
^{13}C NMR (100 MHz, $CDCl_3$): δ 194.6, 193.7, 156.8, 147.8, 136.1, 132.7, 132.5, 131.7, 130.1, 129.7, 129.6, 129.2, 129.1, 129.0, 128.1, 127.4, 123.8, 123.6, 122.1, 112.7, 71.2.
HRMS (ESI+) calculated for: $[C_{21}H_{16}O_3+Na]^+$ 339.0992; found: 339.0999.
IR, ṽ (cm^{-1}): C=O 1684, 1671.

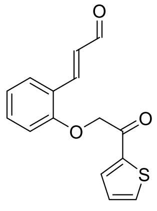

1p

Following the general procedure, the second step was over in 12 h. The aldehyde **1p** was purified by FC on silica (20:80 to 30:70 EtOAc/pentane). The product was then recrystallized from CH_2Cl_2/pentane. The aldehyde **1p** was isolated in 26% yield (354 mg) as a yellow solid.
^{1}H NMR (400 MHz, $CDCl_3$): δ 9.72 (d, *J* = 7.8 Hz, 1H), 7.99 (d, *J* = 16.1 Hz, 1H), 7.90 (dd, *J* = 3.8, 1.1 Hz, 1H), 7.74 (dd, *J* = 4.9, 1.1 Hz, 1H), 7.61 (dd, *J* = 7.8, 1.7 Hz, 1H), 7.38 (ddd, *J* = 8.6, 7.5, 1.7 Hz, 1H), 7.19 (dd, *J* = 4.9, 3.8 Hz, 1H), 7.06 (t, *J* = 7.5 Hz, 1H), 6.90 – 6.78 (m, 2H), 5.25 (s, 2H).
^{13}C NMR (100 MHz, $CDCl_3$): δ 194.6, 187.3, 156.6, 147.7, 140.4, 135.1, 132.9, 132.8, 129.6, 128.9, 128.6, 123.7, 122.2, 112.6, 71.4.
HRMS (ESI+) calculated for: $[C_{15}H_{12}O_3S+Na]^+$ 295.0399; found: 295.0405.
IR, ṽ (cm^{-1}): C=O 1677, 1661.

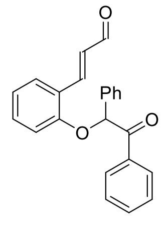

1q

Following the general procedure, the second step was over in 5 d. The aldehyde **1q** was purified by FC on silica (10:40:50 EtOAc/pentane). The product was then recrystallized from CH_2Cl_2/pentane. The aldehyde **1q** was isolated in 55% yield (942 mg) as a white solid.

^{1}H NMR (400 MHz, CDCl$_3$): δ 9.61 (d, *J* = 7.8 Hz, 1H), 8.03 – 7.98 (m, 2H), 7.92 (d, *J* = 16.1 Hz, 1H), 7.58 (dtd, *J* = 9.6, 7.7, 1.5 Hz, 4H), 7.47 – 7.34 (m, 5H), 7.30 (ddd, *J* = 8.7, 7.4, 1.7 Hz, 1H), 7.02 (t, *J* = 7.5 Hz, 1H), 6.85 (dd, *J* = 8.4, 1.0 Hz, 1H), 6.74 (dd, *J* = 16.1, 7.8 Hz, 1H), 6.48 (s, 1H).

^{13}C NMR (100 MHz, CDCl$_3$): δ 195.0, 194.6, 156.2, 147.7, 134.8, 134.6, 133.9, 132.7, 129.4, 129.4 (2C), 129.3, 129.2 (2C), 128.9 (2C), 128.7, 127.7 (2C), 124.1, 122.2, 113.9, 83.2.

HRMS (ESI+) calculated for: $[C_{23}H_{18}O_3+OH]^+$ 365.1148; found: 365.1154.

IR, ṽ (cm^{-1}): C=O 1668, 1595.

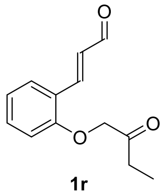

1r

Following the general procedure, the second step was over in 22h. The aldehyde **1r** was purified by FC on silica (1:15 to 1:5 EtOAc/ Pentane). The aldehyde **1r** was isolated in 68% yield (742 mg) as a pale yellow solid.

^{1}H NMR (400 MHz, CDCl$_3$): δ 9.73 (d, J = 7.8 Hz, 1H), 7.93 (d, J = 16.1 Hz, 1H), 7.61 (dd, J = 7.8, 1.7 Hz, 1H), 7.39 (ddd, J = 8.6, 7.4, 1.7 Hz, 1H), 7.06 (td, J = 7.6, 1.0 Hz, 1H), 6.82 (dd, J = 16.1, 7.8 Hz, 1H), 6.76 (dd, J = 8.4, 1.0 Hz, 1H), 4.69 (s, 2H), 2.63 (q, J = 7.3 Hz, 2H), 1.14 (t, J = 7.3 Hz, 3H).

^{13}C NMR (100 MHz, CDCl$_3$): δ 207.1, 194.5, 156.4, 147.5, 132.8, 129.5, 129.0, 123.5, 122.1, 112.2, 72.8, 32.6, 7.2.

HRMS (ESI+) calculated for: $[C_{13}H_{14}O_3+Na]^+$ 241.0835; found: 241.0840.

IR, ṽ (cm^{-1}): C=O 1718, 1670.

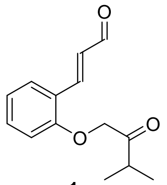

1s

Following the general procedure, the second step was over in 18h. The aldehyde **1s** was purified by FC on silica (1:15 to 1:5 EtOAc/ Pentane). The aldehyde **1s** was isolated in 42% yield (488 mg) as a yellow solid.

^{1}H NMR (400 MHz, CDCl$_3$): δ 9.72 (d, J = 7.8 Hz, 1H), 7.94 (d, J = 16.1 Hz, 1H), 7.60 (dd, J = 7.8, 1.7 Hz, 1H), 7.38 (ddd, J = 8.4, 7.4, 1.7 Hz, 1H), 7.05 (td, J = 7.4, 0.9 Hz, 1H), 6.82 (dd, J = 16.1, 7.8 Hz, 1H), 6.75 (dd, J = 8.4, 1.0 Hz, 1H), 4.79 (s, 2H), 2.90 (hept, J = 6.9 Hz, 1H), 1.20 (d, J = 6.9 Hz, 6H).

^{13}C NMR (100 MHz, CDCl$_3$): δ 209.5, 194.5, 156.5, 147.6, 132.7, 129.5, 129.0, 123.6, 122.0, 112.2, 71.6, 37.5, 18.1 (2C).

HRMS (ESI+) calculated for: $[C_{14}H_{16}O_3+Na]^+$ 255.0992; found: 255.0997.

IR, ṽ (cm^{-1}): C=O 1725, 1666.

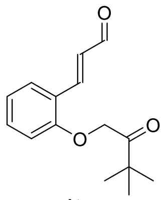

1t

Following the general procedure, the second step was over in 25h. The aldehyde **1t** was purified by FC on silica (1:9 to 1:5 EtOAc/ Pentane). The aldehyde **1t** was isolated in 75% yield (923 mg) as a yellow solid.

^{1}H NMR (400 MHz, $CDCl_3$): δ 9.72 (d, J = 7.8 Hz, 1H), 7.97 (d, J = 16.1 Hz, 1H), 7.59 (dd, J = 7.8, 1.7 Hz, 1H), 7.36 (ddd, J = 8.3, 7.4, 1.7 Hz, 1H), 7.06 – 7.00 (m, 1H), 6.84 (dd, J = 16.1, 7.8 Hz, 1H), 6.71 (dd, J = 8.4, 1.0 Hz, 1H), 5.01 (s, 2H), 1.27 (s, 9H).

^{13}C NMR (100 MHz, $CDCl_3$): δ 208.8, 194.8, 156.8, 147.9, 132.5, 129.4, 129.0, 123.8, 121.8, 112.3, 69.0, 43.3, 26.5 (3C).

HRMS (ESI+) calculated for: $[C_{15}H_{18}O_3+Na]^+$ 269.1148; found: 269.1154.

IR, ṽ (cm^{-1}): C=O 1722, 1670.

3. The asymmetric synthesis of cyclopenta[b]benzofurans

3.1 Optimization

Table 1. Brønsted base catalyst screening:[a]

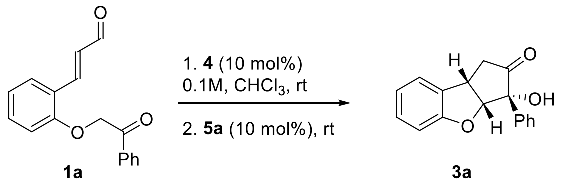

1a 3a

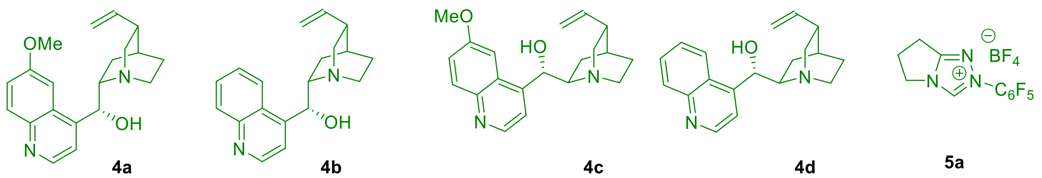

4a Quinine 4b Cinchonidine 4c Quinidine 4d Cinchonine 5a

Catalyst	t_1[b] (h)	t_2[b] (h)	dr_1[c]	dr_2[c]	yield (%)	ee[d] (%)
**4a**	24	24	10:1	20:1	54	92
**4b**	24	24	9:1	12:1	48	86
**4c**	24	24	10:1	19:1	54	-93
**4d**	24	24	7:1	16:1	53	-89

[a] Reactions were performed on a 0.1 mmol scale. [b] Determined by ^{1}H NMR of the crude reaction mixture. [c] Diastereomeric ratio was determined by ^{1}H NMR analysis of the crude reaction mixture. [d] Enantiomeric excess was determined by UPC^2.

Table 2. Solvent screening:[a]

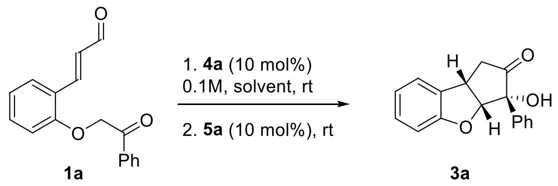

Solvent	$t_{1(conversion)}$[b]	$t_{2(conversion)}$[b]	dr_1[c]	dr_2[c]	yield (%)	ee[c] (%)
PhMe	48 h (100%)	>8 d (61%)	10:1	12:1	28	93
PhCl	24 h (100%)	>9 d (79%)	13:1	18:1	40	93
PhOMe	48 h (100%)	>8 d (62%)	15:1	18:1	32	93
THF	>24 h (13%)	-	-	-	-	-
MeCN	>48 h (76%)	-	-	-	-	-
CH_2Cl_2	48 h (100%)	1 d (100%)	12:1	>20:1	56	91
$ClCH_2CH_2Cl$	48 h (100%)	1 d (100%)	7:1	>20:1	61	91

[a] Reactions were performed on a 0.1 mmol scale. [b] Determined by ^1H NMR of the crude reaction mixture. [c] Diastereomeric ratio was determined by ^1H NMR analysis of the crude reaction mixture. [d] Enantiomeric excess was determined by UPC^2.

Table 3. Concentration and temperature screening:[a]

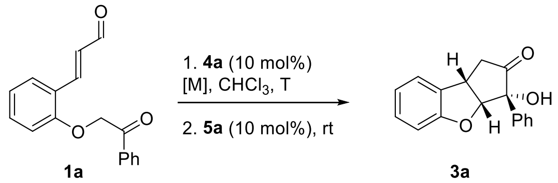

T	[conc.]	t_1[b] (h)	t_2[b] (h)	dr_1[c]	dr_2[c]	yield (%)	ee[d] (%)
0 °C	0.1M	96	24	12:1	-	-	-
0 °C	0.25M	72	<12	11:1	15:1	60	93
0 °C	0.5M	36	<12	10:1	16:1	51	91
rt	0.1M	>48	-	-	-	-	-
rt	0.25M	32	<12	13:1	19:1	62	93
rt	0.25M	32	<12	10:1	19:1	55	-94[e]

[a]Reactions were performed on a 0.25 mmol scale. [b]Determined by ^1H NMR of the crude reaction mixture. [c]Diastereomeric ratio was determined by ^1H NMR analysis of the crude reaction mixture. [d]Enantiomeric excess was determined by UPC^2.[e]Quinidide was used as a catalyst.

Table 4. Chiral NHC screening:[a]

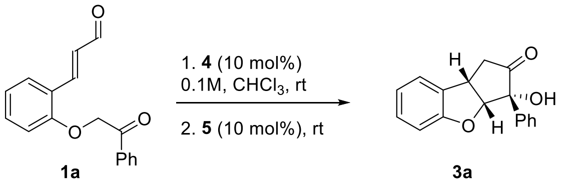

1a **3a**

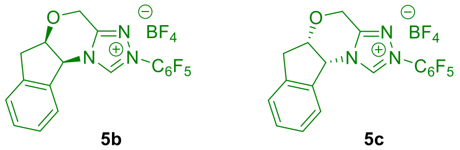

5b **5c**

NHC	t_1[b] (h)	t_2[b] (h)	dr_1[c]	dr_2[c]	yield (%)	ee[c] (%)
**5b**	20	16	7:1	5:1	44	95
**5c**	20	12	7:1	>20:1	60	93

[a] Reactions were performed on a 0.25 mmol scale. [b] Determined by ^{1}H NMR of the crude reaction mixture. [c] Diastereomeric ratio was determined by ^{1}H NMR analysis of the crude reaction mixture. [d] Enantiomeric excess was determined by UPC^2.

3.2. General procedure for organocatalytic synthesis of 3a-p

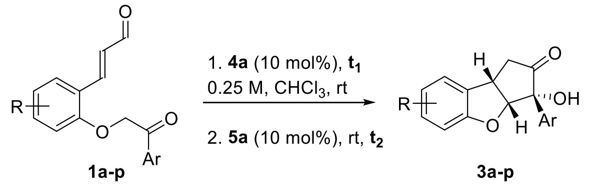

The respective aldehyde **1** (1 eq, 0.25 mmol) and quinine **4a** (0.1 eq, 0.025 mmol, 8.1 mg) were dissolved in dry $CHCl_3$ (1 mL). The reaction mixture was stirred at rt until full conversion. Afterwards, the NHC catalyst **5a** (0.1 eq, 0.025 mol, 9.1 mg) was added in one portion and the reaction mixture was stirred at rt until full conversion. The crude reaction mixture was then loaded in silica gel and purified by FC.

3.3. Results and characterization

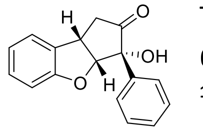

3a

The product **3a** was isolated by FC on silica (1:1:8 Et_2O/CH_2Cl_2/pentane) as a white solid, in 62% yield (41.3 mg), 19:1 dr and 93% ee; t_1: 28 h; t_2: 12 h.

^{1}H NMR (400 MHz, $CDCl_3$): δ 7.53 – 7.47 (m, 2H), 7.47 – 7.36 (m, 3H), 7.24 – 7.16 (m, 2H), 6.95 (t, *J* = 7.5, 1H), 6.90 (d, *J* = 8.0 Hz, 1H), 5.45 (d, *J* = 7.4 Hz, 1H), 4.15 (ddd, *J* = 11.1, 7.4, 3.6 Hz, 1H), 3.46 (s, 1H), 3.01 (dd, *J* = 18.7, 11.1 Hz, 1H), 2.26 (dd, *J* = 18.7, 3.6 Hz, 1H).

^{13}C NMR (100 MHz, $CDCl_3$): δ 213.4, 158.7, 137.5, 130.9, 129.3 (3C), 129.2, 125.8 (2C), 124.7, 122.2, 110.4, 88.1, 82.2, 40.2, 38.6.

HRMS (ESI+) m/z calculated for: $[C_{17}H_{14}O_3+Na]^+$ 289.0835; found: 289.0839.

IR, ṽ (cm^{-1}): O-H 3485; C=O 1750.

The ee was determined by UPC^2 using a Chiralpak IB column [CO_2/*i*PrOH, gradient, 120 bar, 40 °C]; flow rate 3.0 mL/min; τ_{major} = 3.73 min, τ_{minor} = 3.87 min (93% ee). $[\alpha]^{20}_D$ = 53.6.2 (c = 0.25, CH_2Cl_2).

For ***ent*-3a** 55% yield (36.6 mg), 19:1 dr, τ_{major} = 3.86 min, τ_{minor} = 3.75 min (-94% ee).

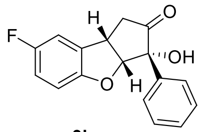

3b

The product **3b** was isolated by FC on silica (1:1:8 Et_2O/CH_2Cl_2/pentane) as a white solid, in 58% yield (41.2 mg), 12:1 dr and 95% ee; t_1: 28 h; t_2: 16 h.

^{1}H NMR (400 MHz, $CDCl_3$): δ 7.50 – 7.36 (m, 5H), 6.94 – 6.84 (m, 2H), 6.80 (dd, *J* = 8.7, 4.2 Hz, 1H), 5.48 (d, *J* = 7. 3 Hz, 1H), 4.10 (ddd, *J* = 11.2, 7.3, 3.7 Hz, 1H), 3.46 (s, 1H), 3.00 (dd, *J* = 18.8, 11.2 Hz, 1H), 2.24 (dd, *J* = 18.8, 3.7 Hz, 1H).

^{13}C NMR (100 MHz, $CDCl_3$): δ 212.9, 158.2 (d, *J* = 239.4 Hz), 154.7 (d, *J* = 1.7 Hz), 137.2, 132.2 (d, *J* = 8.6 Hz), 129.3 (3C), 125.76 (2C), 115.6 (d, *J* = 24.2 Hz), 111.7 (d, *J* = 25.0 Hz), 110.8 (d, *J* = 8.6 Hz), 88.8, 82.2, 39.9, 38.8 (d, *J* = 1.8 Hz).

^{19}F NMR (376 MHz, $CDCl_3$): δ -121.98.

HRMS (ESI+) m/z calculated for: $[C_{17}H_{13}O_3F+Na]^+$ 307.0741; found: 307.0739.

IR, ṽ (cm^{-1}): O-H 3471; C=O 1751.

The ee was determined by UPC^2 using a Chiralpak ID column [CO_2/*i*PrOH, gradient, 120 bar, 40 °C]; flow rate 3.0 mL/min; τ_{major} = 5.30 min, τ_{minor} = 4.79 min (95% ee). $[\alpha]^{20}_D$ = 54.0 (c = 0.2, CH_2Cl_2).

For ***ent*-3b** 65% yield (46.2 mg), 13:1 dr, τ_{major} = 4.78 min, τ_{minor} = 5.34 min (-95% ee).

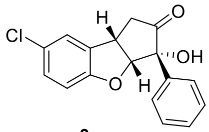

3c

The product **3c** was isolated by FC on silica (1:1:8 Et_2O/CH_2Cl_2/pentane) as a green oil, in 67% yield (50.4 mg), 14:1 dr and 95% ee; t_1: 20 h; t_2: 16 h.

^{1}H NMR (400 MHz, CDCl$_3$): δ 7.50 – 7.36 (m, 5H), 7.18 – 7.12 (m, 2H), 6.82 (d, *J* = 8.4 Hz, 1H), 5.48 (d, *J* = 7.3 Hz, 1H), 4.10 (ddd, *J* = 11.2, 7.3, 3.6 Hz, 1H), 3.42 (s, 1H), 3.01 (dd, *J* = 18.8, 11.2 Hz, 1H), 2.24 (dd, *J* = 18.8, 3.6 Hz, 1H).

^{13}C NMR (100 MHz, CDCl$_3$): δ 212.7, 157.5, 137.2, 132.8, 129.4, 129.3 (2C), 129.2, 126.8, 125.8 (2C), 124.8, 111.5, 88.8, 82.1, 39.9, 38.6.

HRMS (ESI+) m/z calculated for: $[C_{17}H_{13}O_3Cl+Na]^+$ 323.0445; found: 323.0446.

IR, ṽ (cm^{-1}): O-H 3475; C=O 1752.

The ee was determined by UPC^2 using a Chiralpak ID column [CO_2/*i*PrOH, gradient, 120 bar, 40 °C]; flow rate 3.0 mL/min; τ_{major} = 6.57 min, τ_{minor} = 5.49 min (95% ee). $[\alpha]^{20}_D$ = 16.0 (c = 0.25, CH_2Cl_2).

For ***ent*-3c** 64% yield (48.1 mg), 14:1 dr, τ_{major} = 5.50 min, τ_{minor} = 6.64 min (-95% ee).

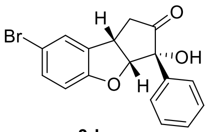

3d

The product **3d** was isolated by FC on silica (1:1:8 Et_2O/CH_2Cl_2/pentane) as a white solid, in 66% yield (57.0 mg), 11:1 dr and 95% ee; t_1: 20 h; t_2: 22 h.

^{1}H NMR (400 MHz, CDCl$_3$): δ δ 7.42 – 7.30 (m, 5H), 7.26 – 7.18 (m, 2H), 6.70 (d, *J* = 8.4 Hz, 1H), 5.40 (d, *J* = 7.4 Hz, 1H), 4.03 (ddd, *J* = 11.1, 7.4, 3.6 Hz, 1H), 3.38 (s, 1H), 2.93 (dd, *J* = 18.8, 11.1 Hz, 1H), 2.17 (dd, *J* = 18.8, 3.6 Hz, 1H).

^{13}C NMR (100 MHz, CDCl$_3$): δ 212.7, 158.0, 137.2, 133.3, 132.1, 129.4, 129.3 (2C), 127.7, 125.7 (2C), 113.8, 112.0, 88.7, 82.1, 39.9, 38.5.

HRMS (ESI+) m/z calculated for: $[C_{17}H_{13}O_3Br+Na]^+$ 366.9940; found: 366.9941.

IR, ṽ (cm^{-1}): O-H 3466; C=O 1750.

The ee was determined by UPC^2 using a Chiralpak ID column [CO_2/*i*PrOH, gradient, 120 bar, 40 °C]; flow rate 3.0 mL/min; τ_{major} = 7.59 min, τ_{minor} = 6.01 min (95% ee). $[\alpha]^{20}_D$ = 54.2 (c = 0.25, CH_2Cl_2).

For ***ent*-3d** 53% yield (45.7 mg), 12:1 dr, τ_{major} = 5.99 min, τ_{minor} = 7.63 min (-96% ee).

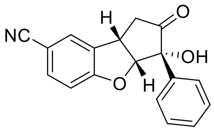

3e

The product **3e** was isolated by FC on silica (EtOAc/pentane 1:10 to 1:4) as a white solid, in 45% yield (32.5 mg), 9:1 dr and 87% ee; t_1: 7 h; t_2: 20 h.

^{1}H NMR (400 MHz, CDCl$_3$): δ 7.54 – 7.39 (m, 7H), 6.95 (d, J = 8.3 Hz, 1H), 5.55 (d, J = 7.4 Hz, 1H), 4.17 (ddd, J = 11.1, 7.4, 3.6 Hz, 1H), 3.47 (s, 1H), 3.05 (dd, J = 18.8, 11.3 Hz, 1H), 2.22 (dd, J = 18.8, 3.6 Hz, 1H).

^{13}C NMR (100 MHz, CDCl$_3$): δ 212.1, 162.4, 136.9, 134.6, 132.7, 129.5, 129.4, 128.8, 125.7, 119.0, 111.4, 105.5, 89.3, 82.0, 39.8, 38.0.

HRMS (ESI+) m/z calculated for: $[C_{18}H_{13}NO_3+Na]^+$ 314.0788; found: 314.0789.

IR, ṽ (cm^{-1}): C≡N 2230; C=O 1666.

The ee was determined by UPC^2 using a Chiralpak IC-3 column [CO_2/*i*PrOH, gradient, 120 bar, 40 °C]; flow rate 3.0 mL/min; τ_{major} = 5.67 min, τ_{minor} = 6.31 min (87% ee). $[\alpha]^{20}_D$ = -41.2 (c = 1.0, CH_2Cl_2).

For the other enantiomer: 52% yield, 89% ee.

For ***ent*-3e** 52% yield (37.9 mg), 8:1 dr, τ_{major} = 6.30 min, τ_{minor} = 5.67 min (-89% ee).

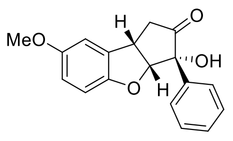

3f

The product **3f** was isolated by FC on silica (15:85 EtOAc/pentane) as a yellow oil, in 46% yield (34.1 mg), 12:1 dr and 95% ee. t_1: 48 h; t_2: 6 d.

^{1}H NMR (400 MHz, CDCl$_3$): δ 7.51 – 7.46 (m, 2H), 7.45 – 7.36 (m, 3H), 6.81 – 6.76 (m, 2H), 6.72 (dd, *J* = 8.7, 2.7 Hz, 1H), 5.43 (d, *J* = 7.3 Hz, 1H), 4.09 (ddd, *J* = 11.2, 7.3, 3.6 Hz, 1H), 3.76 (s, 3H), 3.49 (s, 1H), 2.99 (dd, *J* = 18.8, 11.2 Hz, 1H), 2.26 (dd, *J* = 18.8, 3.6 Hz, 1H).

^{13}C NMR (100 MHz, CDCl$_3$): δ 213.3, 155.2, 152.7, 137.5, 131.9, 129.2 (2C), 125.8 (2C), 114.0, 110.7, 110.4, 88.4, 82.2, 56.1, 40.0, 39.0.

HRMS (ESI+) m/z calculated for: $[C_{18}H_{16}O_4+Na]^+$ 319.0941; found: 319.0943.

IR, ṽ (cm^{-1}): O-H 3447; C=O 1751.

The ee was determined by UPC^2 using a Chiralpak ID column [CO_2/*i*PrOH, gradient, 120 bar, 40 °C]; flow rate 3.0 mL/min; τ_{major} = 7.49 min, τ_{minor} = 6.56 min (95% ee). $[\alpha]^{20}_{D}$ = 40.0 (c = 0.2, CH_2Cl_2).
For ***ent*-3f** 40% yield (29.6 mg), 14:1 dr, τ_{major} = 6.42 min, τ_{minor} = 7.57 min (-96% ee).

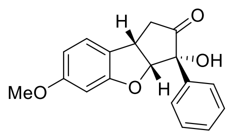

3g

The product **3g** was isolated by FC on silica (15:85 EtOAc/pentane) as a yellow oil, in 53% yield (39.3 mg), 12:1 dr and 92% ee; t_1: 48 h; t_2: 24 h.

^{1}H NMR (400 MHz, CDCl$_3$): δ 7.51 – 7.46$^{\#}$ (m, 4H), 7.46 – 7.31$^{\#}$ (m, 6H), 7.08$^{\#}$ (d, *J* = 8.1 Hz, 2H), 6.51 – 6.42$^{\#}$ (m, 3H), 6.20* (d, *J* = 2.3 Hz, 1H), 5.47 (d, *J* = 7.4 Hz, 1H), 5.14* (dd, *J* = 8.0, 2.3 Hz, 1H), 4.20 – 4.13* (m, 1H), 4.09 (ddd, *J* = 10.9, 7.4, 3.4 Hz, 1H), 3.77 (s, 3H), 3.70* (s, 3H), 3.47$^{\#}$ (m, 2H), 3.32* (dd, *J* = 19.2, 10.2 Hz, 1H), 2.97 (dd, *J* = 18.7, 10.9 Hz, 1H), 2.50* (dd, *J* = 19.2, 5.2 Hz, 1H), 2.22 (dd, *J* = 18.7, 3.4 Hz, 1H).

^{13}C NMR (100 MHz, CDCl$_3$): δ 213.5, 161.1, 160.0, 137.6, 129.3 (2C), 129.2, 125.7 (2C), 124.8, 122.8, 108.0, 96.8, 88.9, 82.07, 55.7, 40.4, 38.1.

HRMS (ESI+) m/z calculated for: $[C_{18}H_{16}O_4+Na]^+$ 319.0941; found: 319.0943.

IR, ṽ (cm^{-1}): O-H 3475; C=O 1751.

The ee was determined by UPC^2 using a Chiralpak ID column [CO_2/*i*PrOH, gradient, 120 bar, 40 °C]; flow rate 3.0 mL/min; τ_{major} = 6.70 min, τ_{minor} = 6.29 min (92% ee). $[\alpha]^{20}_{D}$ = 49.4 (c = 0.3, CH_2Cl_2).
For ***ent*-3g** 49% yield (36.3 mg), 15:1 dr, τ_{major} = 6.25 min, τ_{minor} = 6.74 min (-94% ee).

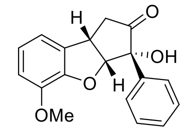

3h

The product **3h** was isolated by FC on silica (15:85 EtOAc/pentane) as a yellow oil, in 57% yield (42.2 mg), 19:1 dr and 93% ee; t_1: 48 h; t_2: 24 h.

^{1}H NMR (400 MHz, CDCl$_3$): δ 7.52–7.47$^{\#}$ (m, 4H), 7.46 – 7.34$^{\#}$ (m, 6H), 6.91 (t, *J* = 7.8 Hz, 1H), 6.85 – 6.76$^{\#}$ (m, 4H), 6.72 – 6.66* (m, 1H), 5.49 (d, *J* = 7.5 Hz, 1H), 5.20* (d, *J* = 8.2 Hz, 1H), 4.24* (ddd, *J* = 10.3, 8.2, 4.8 Hz, 1H), 4.16 (ddd, *J* = 11.1, 7.5, 3.6 Hz, 1H), 3.88$^{\#}$ (s, 4H), 3.65* (s, 3H), 3.50 (s, 1H), 3.33* (dd, *J* = 19.4, 10.3 Hz, 1H), 2.99 (dd, *J* = 18.7, 11.1 Hz, 1H), 2.60* (dd, *J* = 19.4, 4.8 Hz, 1H), 2.27 (dd, *J* = 18.7, 3.6 Hz, 1H).

^{13}C NMR (100 MHz, CDCl$_3$): δ 213.3, 146.9, 144.9, 137.5, 132.1, 129.2 (2C), 129.1, 125.8 (2C), 123.1, 116.5, 112.1, 88.7, 82.1, 56.1, 40.1, 39.1.

HRMS (ESI+) m/z calculated for: $[C_{18}H_{16}O_4+Na]^+$ 319.0941; found: 319.0942.

IR, ṽ (cm^{-1}): O-H 3484; C=O 1750.

The ee was determined by UPC^2 using a Chiralpak ID column [CO_2/*i*PrOH, gradient, 120 bar, 40 °C]; flow rate 3.0 mL/min; τ_{major} = 7.99 min, τ_{minor} = 6.38 min (93% ee). $[\alpha]^{20}_D$ = 50.0 (c = 0.2, CH_2Cl_2).
For ***ent*-3h** 52% yield (38.5 mg), 9:1 dr, τ_{major} = 6.36 min, τ_{minor} = 8.11 min (-96% ee).

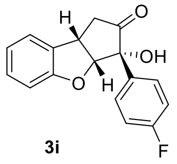

3i

The product **3i** was isolated by FC on silica (1:1:8 Et_2O/CH_2Cl_2/pentane) as a white solid, in 48% yield (34.1 mg), 7:1 dr and 93% ee; t_1: 28 h; t_2: 16 h.
^{1}H NMR (400 MHz, CDCl$_3$): δ 7.51 – 7.45 (m, 2H), 7.23 – 7.16 (m, 2H), 7.12 (t, *J* = 8.5 Hz, 2H), 6.95 (t, *J* = 7.4 Hz, 1H), 6.89 (d, *J* = 8.0 Hz, 1H), 5.41 (d, *J* = 7.4 Hz, 1H), 4.13 (ddd, *J* = 11.1, 7.4, 3.6 Hz, 1H), 3.53 – 3.43 (m, 1H), 2.98 (dd, *J* = 18.8, 11.1 Hz, 1H), 2.27 (dd, *J* = 18.8, 3.6 Hz, 1H).
^{13}C NMR (100 MHz, CDCl$_3$): δ 213.0, 163.1 (d, *J* = 249.0 Hz), 158.6, 133.4 (d, *J* = 3.2 Hz), 130.8, 129.3, 127.8 (d, *J* = 8.4 Hz) (2C), 124.7, 122.3, 116.3 (d, *J* = 21.7 Hz) (2C), 110.4, 87.93, 81.6, 40.1, 38.5.
^{19}F NMR (376 MHz, CDCl$_3$): δ -112.05
HRMS (ESI+) m/z calculated for: $[C_{17}H_{13}O_3F+Na]^+$ 307.0741; found: 307.0739.
IR, ṽ (cm^{-1}): O-H 3475; C=O 1751.

The ee was determined by UPC^2 using a Chiralpak ID column [CO_2/*i*PrOH, gradient, 120 bar, 40 °C]; flow rate 3.0 mL/min; τ_{major} = 5.14 min, τ_{minor} = 4.63 min (93% ee). $[\alpha]^{20}_D$ = 52.0 (c = 0.2, CH_2Cl_2).
For ***ent*-3i** 42% yield (29.9 mg), 8:1 dr, τ_{major} = 4.62 min, τ_{minor} = 5.18 min (-98% ee).

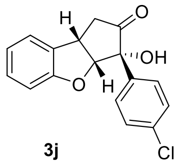

3j

The product **3j** was isolated by FC on silica (1:1:8 Et_2O/CH_2Cl_2/pentane) as a yellow oil, in 54% yield (40.6 mg), 12:1 dr and 93% ee; t_1: 18 h; t_2: 16 h.
^{1}H NMR (400 MHz, CDCl$_3$): δ 7.46 – 7.38 (m, 4H), 7.24 – 7.16 (m, 2H), 6.96 (td, *J* = 7.5, 1.0 Hz, 1H), 6.89 (d, *J* = 8.0 Hz, 1H), 5.39 (d, *J* = 7.5 Hz, 1H), 4.15 (ddd, *J* = 11.1, 7.5, 3.6 Hz, 1H), 3.51 – 3.44 (m, 1H), 2.99 (dd, *J* = 18.8, 11.1 Hz, 1H), 2.29 (dd, *J* = 18.8, 3.6 Hz, 1H).
^{13}C NMR (100 MHz, CDCl$_3$): δ 212.8, 158.6, 136.1, 135.3, 130.7, 129.4 (2C), 129.3, 127.2 (2C), 124.7, 122.3, 110.4, 87.8, 81.6, 40.1, 38.6.
HRMS (ESI+) m/z calculated for: $[C_{17}H_{13}O_3Cl+Na]^+$ 323.0445; found: 323.0445.
IR, ṽ (cm^{-1}): O-H 3491; C=O 1751.

The ee was determined by UPC^2 using a Chiralpak ID column [CO_2/*i*PrOH, gradient, 120 bar, 40 °C]; flow rate 3.0 mL/min; τ_{major} = 6.08 min, τ_{minor} = 5.30 min (93% ee). $[\alpha]^{20}_D$ = 63.2 (c = 0.25, CH_2Cl_2).
For ***ent*-3j** 49% yield (36.8 mg), 8:1 dr, τ_{major} = 6.15 min, τ_{minor} = 5.30 min (-95% ee).

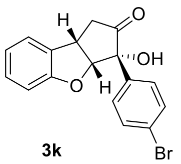

3k

The product **3k** was isolated by FC on silica (1:1:8 Et_2O/CH_2Cl_2/pentane) as a white solid, in 48% yield (41.4 mg), 7:1 dr and 96% ee; t_1: 20 h; t_2: 22 h.
^{1}H NMR (400 MHz, CDCl$_3$): δ 7.46 – 7.38 (m, 4H), 7.24 – 7.16 (m, 2H), 6.96 (td, *J* = 7.5, 1.0 Hz, 1H), 6.89 (d, *J* = 8.0 Hz, 1H), 5.39 (d, *J* = 7.5 Hz, 1H), 4.15 (ddd, *J* = 11.1, 7.5, 3.6 Hz, 1H), 3.51 – 3.44 (m, 1H), 2.99 (dd, *J* = 18.8, 11.1 Hz, 1H), 2.29 (dd, *J* = 18.8, 3.6 Hz, 1H).
^{13}C NMR (100 MHz, CDCl$_3$): δ 212.8, 158.6, 136.1, 135.3, 130.7, 129.4 (2C), 129.3, 127.2 (2C), 124.7, 122.3, 110.4, 87.8, 81.6, 40.1, 38.6.
HRMS (ESI+) m/z calculated for: $[C_{17}H_{13}O_3Cl+Na]^+$ 323.0445; found: 323.0445.
IR, ṽ (cm^{-1}): O-H 3502; C=O 1751.

The ee was determined by UPC^2 using a Chiralpak ID column [CO_2/*i*PrOH, gradient, 120 bar, 40 °C]; flow rate 3.0 mL/min; τ_{major} = 6.76 min, τ_{minor} = 5.77 min (96% ee). $[\alpha]^{20}_D$ = 54.2 (c = 0.25, CH_2Cl_2).

For ***ent*-3k** 40% yield (34.5 mg), 7:1 dr, τ_{major} = 5.77 min, τ_{minor} = 6.83 min (-95% ee).

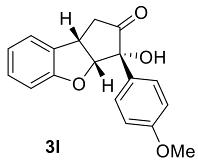

3l

The product **3l** was isolated by FC on silica (15:85 EtOAc/pentane) as a yellow oil, in 42% yield (31.1 mg), 11:1 dr and 94% ee; t_1: 72 h; t_2: 20 h.
^{1}H NMR (400 MHz, $CDCl_3$): δ 7.44 – 7.38 (m, 2H), 7.18 (ddd, *J* = 9.0, 7.5, 1.7 Hz, 2H), 6.91 (dd, *J* = 25.3, 8.2 Hz, 4H), 5.44 (d, *J* = 7.3 Hz, 1H), 4.08 (ddd, *J* = 11.1, 7.3, 3.5 Hz, 1H), 3.82 (s, 3H), 3.43 – 3.38 (m, 1H), 2.97 (dd, *J* = 18.7, 11.1 Hz, 1H), 2.20 (dd, *J* = 18.7, 3.5 Hz, 1H).
^{13}C NMR (100 MHz, $CDCl_3$): δ 213.5, 160.4, 158.7, 131.1, 129.3, 129.2, 127.3 (2C), 124.6, 122.1, 114.6 (2C), 110.4, 88.0, 81.9, 55.5, 40.0, 38.4.
HRMS (ESI+) m/z calculated for: $[C_{18}H_{16}O_4+Na]^+$ 319.0941; found: 319.0942.
IR, ṽ (cm^{-1}): O-H 3500; C=O 1748.

The ee was determined by UPC^2 using a Chiralpak ID column [CO_2/*i*PrOH, gradient, 120 bar, 40 °C]; flow rate 3.0 mL/min; τ_{major} = 7.49 min, τ_{minor} = 6.74 min (94% ee). $[\alpha]^{20}_D$ = 106.0 (c = 0.2, CH_2Cl_2).
For ***ent*-3l** 39% yield (28.9 mg), 15:1 dr, τ_{major} = 6.72 min, τ_{minor} = 7.53 min (-94% ee).

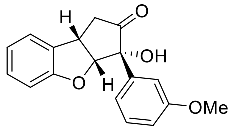

3m

The product **3m** was isolated by FC on silica (EtOAc/pentane 1:15 to 1:5) as a pale yellow oil, in 55% yield (40.7 mg), 15:1 dr and 95% ee; t_1: 48 h; t_2: 24 h.
^{1}H NMR (400 MHz, $CDCl_3$): δ 7.33 (t, J = 8.0 Hz, 1H), 7.23 – 7.15 (m, 2H), 7.09 (t, J = 2.2 Hz, 1H), 7.02 (ddd, J = 7.8, 1.8, 0.9 Hz, 1H), 6.97 – 6.85 (m, 3H), 5.41 (d, J = 7.4 Hz, 1H), 4.14 (ddd, J = 11.1, 7.5, 3.6 Hz, 1H), 3.82 (s, 3H), 3.53 (s, 1H), 3.02 (dd, J = 18.7, 11.1 Hz, 1H), 2.24 (dd, J = 18.8, 3.5 Hz, 1H).
^{13}C NMR (100 MHz, $CDCl_3$): δ 213.2, 160.2, 158.6, 139.0, 130.9, 130.2, 129.2, 124.7, 122.1, 117.5, 114.6, 111.7, 110.3, 88.1, 82.0, 55.5, 40.1, 38.6.
HRMS (ESI+) m/z calculated for: $[C_{18}H_{16}O_4+Na]^+$ 319.0941; found: 319.0947.
IR, ṽ (cm^{-1}): O-H 3475; C=O 1750.

The ee was determined by UPC^2 using a Chiralpak IC column [CO_2/*i*PrOH, gradient, 120 bar, 40 °C]; flow rate 3.0 mL/min; τ_{major} = 5.43 min, τ_{minor} = 5.19 min (95% ee). $[\alpha]^{20}_D$ = +36.8 (c = 1.0, CH_2Cl_2).
For ***ent*-3m** 58% yield (43.0 mg), 15:1 dr, τ_{major} = 5.18 min, τ_{minor} = 5.43 min (-96% ee).

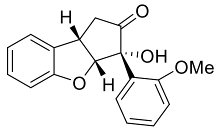

3n

The product **3n** was isolated by FC on silica (EtOAc/pentane 1:20 to 1:5) as a colorless oil, in 44% yield (32.2 mg), 8:1 dr and 94% ee; t_1: 7 d; t_2: 16 h.
^{1}H NMR (400 MHz, $CDCl_3$): δ 7.69 (dd, J = 7.6, 1.7 Hz, 1H), 7.34 (td, J = 7.8, 1.7 Hz, 1H), 7.27 – 7.17 (m, 2H), 7.08 (td, J = 7.5, 1.0 Hz, 1H), 7.01 – 6.95 (m, 1H), 6.94 – 6.88 (m, 2H), 5.43 (d, J = 9.3 Hz, 1H), 4.27 (td, J = 9.8, 5.8 Hz, 1H), 3.81 (s, 3H), 3.56 (s, 1H), 3.11 (dd, J = 19.1, 10.3 Hz, 1H), 2.80 (dd, J = 19.1, 5.8 Hz, 1H).
^{13}C NMR (100 MHz, $CDCl_3$): δ 213.0, 159.2, 154.8, 130.7, 130.2, 129.5, 129.0, 126.7, 124.9, 122.2, 121.4, 111.0, 110.1, 89.8, 77.6, 55.5, 42.3, 40.5.
HRMS (ESI+) m/z calculated for: $[C_{18}H_{16}O_4+Na]^+$ 319.0941; found: 319.0947.
IR, ṽ (cm^{-1}): O-H 3425; C=O 1731.

The ee was determined by UPC^2 using a Chiralpak ID-4 column [CO_2/*i*PrOH, gradient, 120 bar, 40 °C]; flow rate 3.0 mL/min; τ_{major} = 5.57 min, τ_{minor} = 6.44 min (94% ee). $[\alpha]^{20}_D$ = -46.8 (c = 1.0, CH_2Cl_2).
For ***ent*-3n** 40% yield (29.6 mg), 11:1 dr, τ_{major} = 6.40 min, τ_{minor} = 5.59 min (-95% ee).

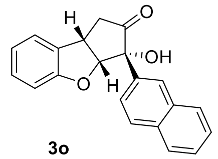

3o

The product **3o** was isolated by FC on silica (EtOAc/pentane 1:20 to 1:5) as a yellow oil, in 51% yield (40.2 mg), 13:1 dr and 94% ee; t_1: 30 h; t_2: 40 h.
^{1}H NMR (400 MHz, CDCl$_3$): δ 7.94 – 7.82 (m, 4H), 7.64 (dd, J = 8.6, 2.0 Hz, 1H), 7.57 – 7.50 (m, 2H), 7.21 (ddd, J = 9.1, 7.5, 1.3 Hz, 2H), 6.96 (td, J = 7.5, 1.0 Hz, 1H), 6.91 (d, J = 8.0 Hz, 1H), 5.57 (d, J = 7.4 Hz, 1H), 4.19 (ddd, J = 11.1, 7.5, 3.6 Hz, 1H), 3.64 (s, 1H), 3.04 (dd, J = 18.7, 11.1 Hz, 1H), 2.29 (dd, J = 18.8, 3.6 Hz, 1H).
^{13}C NMR (100 MHz, CDCl$_3$): δ 213.4, 158.7, 134.8, 133.4, 133.1, 130.9, 129.3, 129.2, 128.4, 127.8, 127.1, 126.8, 124.9, 124.7, 123.4, 122.2, 110.4, 88.0, 82.2, 40.2, 38.6.
HRMS (ESI+) m/z calculated for: $[C_{21}H_{16}O_3+Na]^+$ 339.0992; found: 339.0996.
IR, ṽ (cm^{-1}): O-H 3466; C=O 1751.

The ee was determined by UPC^2 using a Chiralpak ID column [CO_2/*i*PrOH, gradient, 120 bar, 40 °C]; flow rate 3.0 mL/min; τ_{major} = 8.69 min, τ_{minor} = 7.27 min (94% ee). $[\alpha]^{20}_D$ = +117.4 (c = 1.0, CH_2Cl_2).
For ***ent*-3o** 58% yield (45.9 mg), 14:1 dr, τ_{major} = 7.26 min, τ_{minor} = 8.70 min (-95% ee).

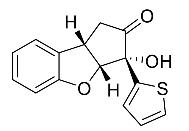

3p

The product **3p** was isolated by FC on silica (1:1:8 Et2O/CH_2Cl_2/pentane) as a yellow oil, in 29% yield (19.7 mg), 6:1 dr and 89% ee; t_1: 18 h; t_2: 14 h.
^{1}H NMR (400 MHz, CDCl$_3$): δ 7.41 (dd, *J* = 5.1, 1.2 Hz, 1H), 7.37* (dd, *J* = 4.8, 1.4 Hz, 1H), 7.24 – 7.15# (m, 4H), 7.14-7.08# (m, 2H), 7.05 (dd, *J* = 5.1, 3.6 Hz, 1H), 7.00 – 6.91# (m, 3H), 6.88 (d, *J* = 8.2, 1H), 6.70* (d, *J* = 8.1, 1H), 5.42 (d, *J* = 7.2 Hz, 1H), 5.24* (d, *J* = 8.4 Hz, 1H), 4.21* (ddd, *J* = 10.5, 8.4, 5.3 Hz, 1H), 4.12 (ddd, *J* = 11.1, 7.2, 3.6 Hz, 1H), 3.56 – 3.50# (m, 2H), 3.29* (dd, *J* = 19.0, 10.5 Hz, 1H), 3.11 (dd, *J* = 18.8, 11.1 Hz, 1H), 2.58* (dd, *J* = 19.0, 5.3, 1H), 2.25 (dd, *J* = 18.8, 3.6 Hz, 1H).
^{13}C NMR (100 MHz, CDCl$_3$): δ 211.7*, 211.2, 159.2*, 158.7, 140.6, 139.3*, 130.6*, 130.8, 129.3, 129.1*, 127.5, 127.3, 127.1*, 127.0*, 126.8*, 125.4, 124.6, 124.5*, 122.3, 121.7*, 110.4, 110.3*, 90.6*, 88.4, 80.4, 79.9*, 41.7*, 39.6, 39.0*, 38.4.
HRMS (ESI+) m/z calculated for: $[C_{15}H_{12}O_3S+Na]^+$ 295.0399; found: 295.0399.
IR, ṽ (cm^{-1}): O-H 3456; C=O 1749.

The ee was determined by UPC^2 using a Chiralpak ID column [CO_2/*i*PrOH, gradient, 120 bar, 40 °C]; flow rate 3.0 mL/min; τ_{major} = 6.21 min, τ_{minor} = 6.04 min (89% ee). $[\alpha]^{20}_D$ = 22.0 (c = 0.2, CH_2Cl_2).
For ***ent*-3p** 29% yield (19.7 mg), 6:1 dr, τ_{major} = 6.02 min, τ_{minor} = 6.232 min (-92% ee).

3.4 Procedure for the synthesis of 3a at 4.0 mmol scale

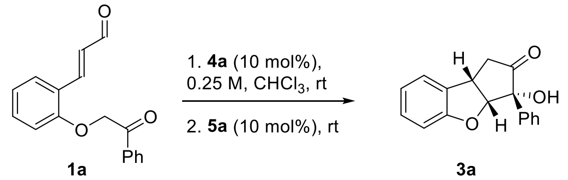

The respective aldehyde **1** (1 eq, 4 mmol, 1.06 g) and quinine **4a** (0.1 eq, 0.4 mmol, 129.8 mg) were dissolved in dry $CHCl_3$ (16 mL). The reaction mixture was stirred at rt for 28 h. Afterwards, the NHC catalyst **5a** (0.1 eq, 0.4 mmol, 145.2 mg) was added in one portion and the reaction mixture was stirred at rt for 12 h. The crude reaction mixture was then loaded in silica gel and purified by FC. The product **3a** was isolated as a white solid in 53% yield (565 mg), 11:1 dr and 93% ee.

4. Transformations

4.1 Reduction

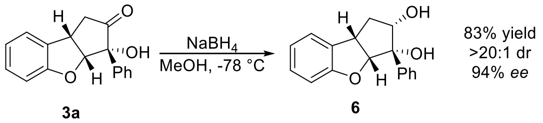

Compound **3a** (1.0 eq, 0.25 mmol, 66.6 mg) was dissolved in dry MeOH (2 mL), and the solution was cooled to -78 °C. $NaBH_4$ (2.5 eq, 0.625 mmol, 23.6mg) was added and the reaction mixture was stirred at -78 °C for 40 min. The reaction was then quenched with $NH_4Cl_{(aq)}$, extracted with CH_2Cl_2, dried over Na_2SO_4, concentrated *in vacuo* and purified by FC over silica gel.

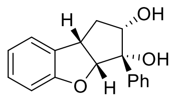

6

The product **6** was isolated by FC on silica (20:80 EtOAc/pentane) as a white foam, in 83% yield (55.4 mg), >20:1 dr and 94% ee.

^{1}H NMR (400 MHz, $CDCl_3$): δ 7.60 – 7.53 (m, 2H), 7.42 (dd, J = 8.5, 6.9 Hz, 2H), 7.37 – 7.29 (m, 1H), 7.24 – 7.12 (m, 2H), 6.94 (td, J = 7.4, 1.0 Hz, 1H), 6.86 (d, J = 8.0 Hz, 1H), 5.30 (d, J = 9.7 Hz, 1H), 4.27 (dd, J = 8.5, 6.2 Hz, 1H), 3.93 (td, J = 9.3, 7.3 Hz, 1H), 3.27 (s, 1H), 2.65 (ddd, J = 12.8, 9.0, 6.2 Hz, 1H), 2.29 (s, 1H), 2.03 (ddd, J = 12.8, 8.6, 7.3 Hz, 1H).

^{13}C NMR (100 MHz, $CDCl_3$): δ 159.2, 142.5, 131.6, 128.7, 128.4, 127.8, 125.5, 124.5, 121.6, 109.7, 91.6, 81.2, 79.2, 42.1, 39.3.

HRMS (ESI+) m/z calculated for: $[C_{17}H_{16}O_3+Na]^+$ 291.0992; found: 291.0996.

IR, ṽ (cm^{-1}): O-H 3470.

The ee was determined by UPC^2 using a Chiralpak ID-4 column [CO_2/*i*PrOH, gradient, 120 bar, 40 °C]; flow rate 3.0 mL/min; τ_{major} = 5.26 min, τ_{minor} = 6.44 min (94% ee). $[\alpha]^{20}_D$ = -88.2 (c = 1.0, CH_2Cl_2).

4.2 Allylation

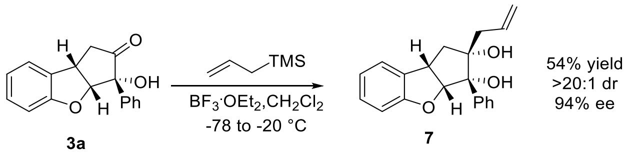

Compound **3a** (1.0 eq, 0.25 mmol, 66.6 mg) was dissolved in dry CH_2Cl_2 (2.5 mL), and allyltrimethylsilane (2.0 eq, 0.5 mmol, 79.5 µL) was added. The solution was cooled to -20 °C and $BF_3 \cdot Et_2O$ (4 eq, 1.0 mmol, 123.4 µL) was added. The reaction mixture was stirred at -20 °C for 30 h. The reaction was then quenched with $NaHCO_{3(aq)}$, extracted with CH_2Cl_2, concentrated *in vacuo* and purified by FC over silica gel

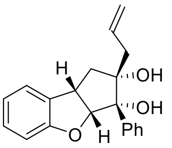

7

The product **7** was isolated by FC on silica (5:95 to 17:83 Et_2O/pentane) as a yellow oil, in 54% yield (41.8 mg), > 20:1 dr and 94% ee.

^{1}H NMR (400 MHz, $CDCl_3$): δ 7.57 – 7.50 (m, 2H), 7.46 – 7.39 (m, 2H), 7.38 – 7.32 (m, 1H), 7.22 (dt, J = 7.4, 1.2 Hz, 1H), 7.16 (td, J = 7.8, 1.4 Hz, 1H), 6.94 (td, J = 7.4, 1.0 Hz, 1H), 6.84 (d, J = 8.0 Hz, 1H), 5.79 (dddd, J = 16.8, 10.1, 8.0, 6.5 Hz, 1H), 5.42 (d, J = 9.2 Hz, 1H), 5.08 (ddt, J = 10.1, 2.0, 1.0 Hz, 1H), 4.99 (dq, J = 17.0, 1.5 Hz, 1H), 4.04 (td, J = 9.3, 4.0 Hz, 1H), 3.66 (s, 1H), 2.48 (dd, J = 13.7, 9.3 Hz, 1H), 2.38 (s, 1H), 2.11 (dd, J = 13.7, 4.0 Hz, 1H), 2.01 (ddt, J = 13.9, 6.5, 1.4 Hz, 1H), 1.65 (ddd, J = 14.0, 8.1, 1.1 Hz, 1H).

^{13}C NMR (100 MHz, $CDCl_3$): δ 159.5, 141.0, 133.6, 132.1, 128.4, 128.3, 127.8, 126.0, 124.3, 121.3, 118.9, 109.4, 90.9, 84.0, 83.4, 42.9, 42.5, 40.9.
HRMS (ESI+) m/z calculated for: $[C_{20}H_{20}O_3+Na]^+$ 331.1305; found: 331.1312.
IR, ṽ (cm^{-1}): O-H 3428; C=C 1597.

The ee was determined by UPC^2 using a Chiralpak ID column [CO_2/*i*PrOH, gradient, 120 bar, 40 °C]; flow rate 3.0 mL/min; τ_{major} = 4.26 min, τ_{minor} = 5.11 min (94% ee). $[\alpha]^{20}_D$ = +11.8 (c = 1.0, CH_2Cl_2).

4.3 Reductive amination

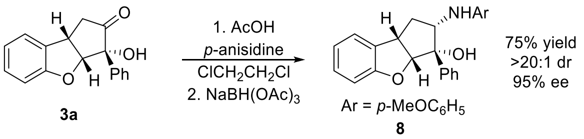

Compound **3a** (1.0 eq, 0.25 mmol, 66.6 mg) was dissolved in $ClCH_2CH_2Cl$ (1.25 mL). *p*-Anisidine (1.2 eq, 0.3 mmol, 36.9 mg) and acetic acid (1.2 eq, 0.3 mmol, 17.2 µL) were added afterwards. The reaction was stirred at rt for 2 h. Afterwards, $NaHB(OAC)_3$ (2.0 eq, 0.5 mmol, 105 mg) was added and stirred at rt for 44 h. After full conversion, the reaction was quenched with $NaHCO_{3(aq)}$, extracted with CH_2Cl_2, concentrated *in vacuo* and purified by FC over silica gel.

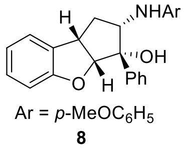

The product **8** was isolated by FC on silica (5:95 to 9:91 EtOAc/pentane) as a yellow oil, in 75% yield (70.0 mg), >20:1 dr and 95% ee.
^1H NMR (400 MHz, $CDCl_3$): δ 7.55 – 7.44 (m, 4H), 7.44 – 7.38 (m, 1H), 7.28 – 7.21 (m, 2H), 7.01 (td, J = 7.4, 1.0 Hz, 1H), 6.94 (d, J = 8.0 Hz, 1H), 6.76 – 6.70 (m, 2H), 6.55 – 6.48 (m, 2H), 5.43 (d, J = 8.7 Hz, 1H), 4.13 (td, J = 8.8, 2.9 Hz, 1H), 3.91 (dd, J = 9.0, 6.0 Hz, 1H), 3.74 (s, 3H), 3.24 (s, 1H), 2.94 (s, 1H), 2.26 (ddd, J = 13.0, 6.0, 3.1 Hz, 1H), 2.07 (dt, J = 13.0, 9.0 Hz, 1H).
^{13}C NMR (100 MHz, $CDCl_3$): δ 159.2, 152.3, 141.4, 139.4, 131.5, 128.6, 128.6, 128.2, 126.7, 124.6, 121.7, 115.3, 114.7, 109.6, 90.0, 83.9, 62.4, 55.8, 42.7, 37.0.
HRMS (ESI+) m/z calculated for: $[C_{24}H_{23}NO_3+Na]^+$ 374.1751; found: 374.1767.
IR, ṽ (cm^{-1}): O-H 3522.

The ee was determined by UPC^2 using a Chiralpak ID column [CO_2/*i*PrOH, gradient, 120 bar, 40 °C]; flow rate 3.0 mL/min; τ_{major} = 6.28 min, τ_{minor} = 6.89 min (95% ee). $[\alpha]^{20}_D$ = +31.1 (c = 2.0, CH_2Cl_2).

4.4 Barton's vinyl iodide synthesis

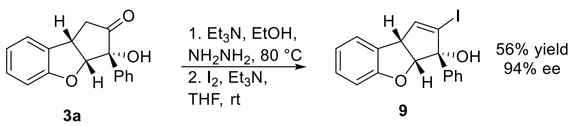

Compound **3a** (1.0 eq, 0.25 mmol, 66.6 mg) was dissolved in absolute EtOH (500 µL). Afterwards, hydrazine monohydrate (20 eq, 5 mmol, 243 µL) and Et_3N (15 eq, 3.75 mmol, 521 µL) were added. The reaction mixture was heated at 80 °C for 3 h. The crude reaction was then diluted with water (20 mL) and extracted with CH_2Cl_2 (3x10 mL), dried over with Na_2SO_4, filtered over cotton, concentrated *in vacuo* and used in the next step without further purification.

In a flame dried vial, the reaction crude was dissolved in dry THF (2 mL). Afterwards, Et_3N (3 eq, 0.75 mmol, 104 µL) was added in one portion. At rt, a solution of iodine (2.2 eq, 0.55 mmol, 140 mg) in dry THF (500 µL) was added dropwise. The reaction mixture was stirred at rt for 30 min. The reaction crude was diluted with Et_2O (30 mL), washed with 2x10 mL of $Na_2SO_{3(aq)}$,10 mL of $NaHCO_{3(aq)}$, dried over $MgSO_4$, filtered over cotton, concentrated *in vacuo* and purified by FC over silica gel (2:98 to 5:95 Et_2O/pentane). The product **9** was obtained as a yellow oil, in 56% yield (52.7 mg) and 94% ee.

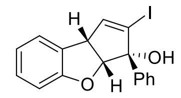

^{1}H NMR (400 MHz, $CDCl_3$): δ 7.53 – 7.47 (m, 2H), 7.47 – 7.39 (m, 2H), 7.35 (td, *J* = 7.1, 1.6 Hz, 1H), 7.29 – 7.18 (m, 2H), 6.96 (t, *J* = 7.4 Hz, 1H), 6.90 (d, *J* = 8.0 Hz, 1H), 6.37 (t, *J* = 1.8 Hz, 1H), 5.02 (dd, *J* = 7.0, 1.4 Hz, 1H), 4.41 (dd, *J* = 6.9, 1.8 Hz, 1H), 3.61 (d, *J* = 1.7 Hz, 1H).
^{13}C NMR (100 MHz, $CDCl_3$): δ 158.6, 141.4, 139.5, 129.2, 128.8 (2C), 128.1, 127.5, 125.3 (2C), 124.2, 122.0, 110.5, 108.8, 88.6, 88.5, 54.1.
HRMS (ESI+) m/z calculated for: $[C_{17}H_{13}O_{3I}+-OH]^+$; 358.9927 found: 358.9928.
IR, ṽ (cm^{-1}): O-H 3531; C=C 1595.

The ee was determined by UPC[2] using a Chiralpak ID column [CO_2/*i*PrOH, gradient, 120 bar, 40 °C]; flow rate 3.0 mL/min; τ_{major} = 5.24 min, τ_{minor} = 4.42 min (94% ee). $[\alpha]^{20}_D$ = -153.6 (c = 0.25, CH_2Cl_2).

5. Diastereodivergence

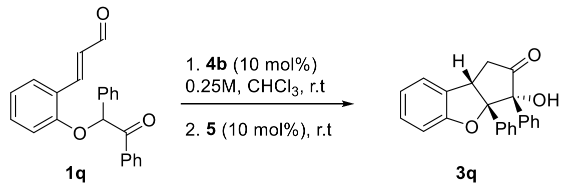

1q 3q

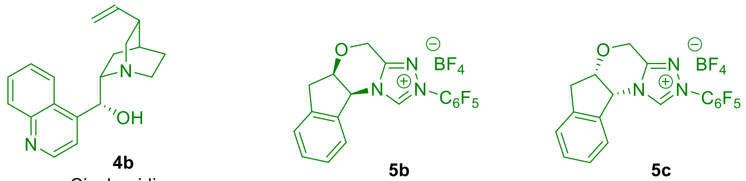

4b Cinchonidine 5b 5c

Aldehyde **1q** (1 eq, 0.1 mol) and cinchonidine (0.1 eq, 0.01 mol, 2.9 mg) were dissolved in dry $CHCl_3$ (400 µL). The reaction mixture was stirred at rt until full conversion. Afterwards, the NHC catalyst **5b** or **5c** (0.1 eq, 0.01 mol, 4.7 mg) was added in one portion and the reaction mixture was stirred at rt until full conversion. The crude reaction mixture was then loaded in silica gel and purified by FC.

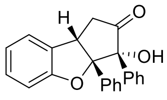

3q

The product **3q** was isolated by FC on silica (1:1:8 Et_2O/CH_2Cl_2/pentane) as a white solid, in 65% yield (55.6 mg), 6:1 dr and 92% ee. t_1: 16 h ; t_2: 24 h. NHC catalyst **5c** was used.

^{1}H NMR (400 MHz, $CDCl_3$): δ 7.38 – 7.33 (m, 2H), 7.29 – 7.26 (m, 2H), 7.22 – 7.08 (m, 8H), 6.92 (t, *J* = 7.5 Hz, 1H), 6.86 (d, *J* = 7.9 Hz, 1H), 4.50 (dd, *J* = 11.4, 4.2 Hz, 1H), 3.74 (s, 1H), 3.39 (dd, *J* = 19.3, 11.4 Hz, 1H), 2.41 (dd, *J* = 19.3, 4.2 Hz, 1H).

^{13}C NMR (100 MHz, $CDCl_3$): δ 213.3, 157.0, 137.1, 136.6, 132.7, 129.2, 128.5, 128.4, 128.3 (2C), 128.1 (2C), 127.1 (2C), 126.5 (2C), 124.0, 122.2, 111.1, 97.9, 86.7, 42.5, 41.6.

HRMS (ESI+) m/z calculated for: $[C_{23}H_{18}O_3+Na]^+$ 365.1148; found: 365.1147.

IR, ṽ (cm^{-1}): O-H 3475; C=O 1745.

The ee was determined by UPC^2 using a Chiralpak ID column [CO_2/MecN, gradient, 120 bar, 40 °C]; flow rate 3.0 mL/min; τ_{major} = 4.59 min, τ_{minor} = 4.88 min (92% ee). $[\alpha]^{20}_{D}$ = 77.0 (c = 0.2, CH_2Cl_2).

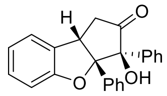

***epi*-3q**

The product ***epi*-3q** was isolated by FC on silica (1:1:8 Et_2O/CH_2Cl_2/pentane) as a white solid, in 63% yield (53.9 mg), 2:1 dr and 96% ee. t_1: 16 h ; t_2: 48 h. NHC catalyst **5b** was used.

^{1}H NMR (400 MHz, $CDCl_3$): δ 7.38 – 7.32 (m, 3H), 7.31 – 7.24 (m, 4H), 7.23 – 7.14 (m, 2H), 7.13 – 7.06 (m, 3H), 6.86 (t, *J* = 7.5, 1H), 6.78 (d, *J* = 7.9 Hz, 1H), 4.43 (dd, *J* = 11.1, 4.4 Hz, 1H), 3.63 (dd, *J* = 19.1, 11.1 Hz, 1H), 2.57 – 2.55 (m, 1H), 2.43 (dd, *J* = 19.1, 4.4 Hz, 1H).

^{3}C NMR (100 MHz, $CDCl_3$): δ 211.4, 157.7, 136.5, 135.2, 132.2, 128.8, 128.7 (2C), 128.4 (2C), 127.9 (2C), 127.5 (2C), 127.4 (2C), 124.4, 121.9, 110.6, 98.4, 82.1, 45.0, 42.9.

HRMS (ESI+) m/z calculated for: $[C_{23}H_{18}O_3+Na]^+$ 365.1148; found: 365.1147.

IR, ṽ (cm^{-1}): O-H 3584; C=O 1751.

The ee was determined by UPC^2 using a Chiralpak ID column [CO_2/*i*PrOH, gradient, 120 bar, 40 °C]; flow rate 3.0 mL/min; τ_{major} = 4.05 min, τ_{minor} = 3.92 min (96% ee). $[\alpha]^{20}_{D}$ = -94.0 (c = 0.2, CH_2Cl_2).

6. Attempts to react with alkyl substituted substrates

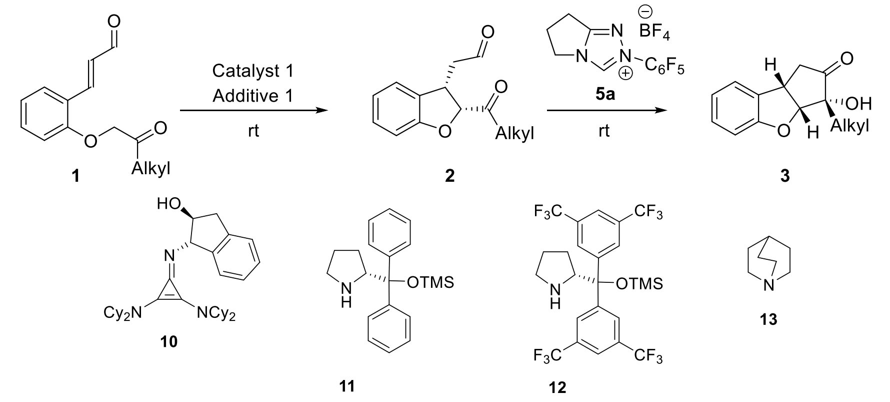

Entry	Substrate	Cat. 1 (mol %)	Additive 1 (mol %)	Cat. 2 (mol %)	Solv.1	Solv.2	Conv. 1	Conv.2
**1**[a]	**3r** (Et)	**10** (10)	-	**5a**(10)	CH_2Cl_2	CH_2Cl_2	>95%	Complex mixture
**2**	**3t** (*t*Bu)	**10** (20)	-	**5a**(10)	THF	$CHCl_3$	>95%	Complex mixture
**3**	**3t** (*t*Bu)	**10** (20)	-	**5a**(10)	PhMe	$CHCl_3$	>95%	Complex mixture
**4**[b]	**3t** (*t*Bu)	**10** (20)	-	**5a**(10)+**13**(10)	PhMe	$CHCl_3$	>95%	Complex mixture
**5**	**3t** (*t*Bu)	**11** (20)	-/**DABCO**(20)/ **DABCO**(20)+**PhCOOH**(40)	-	$CHCl_3$	-	0%	0%
**6**	**3t** (*t*Bu)	**12** (20)	-/**DABCO**(20)/ **DABCO**(20)+**PhCOOH**(40)	-	$CHCl_3$	-	0%	0%

[a] Reaction was performed at -20 °C. [b] Reaction was performed stepwise.

7. Determination of the absolute configuration

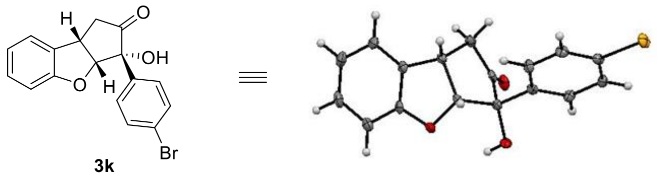

3k

Item	Value
Molecular formula	C17H13BrO3
Formula weight	345.19
Crystal system	orthorhombic
Space Group	P 21 21 21
a (Å)	10.4889
b (Å)	15.637
c (Å)	17.361
α (°)	90
β (°)	90
γ (°)	90
Volume ($Å^3$)	2847.6
Z	8
T (K)	100
ρ (g cm^{-1})	1.61
λ (Å)	0.56086
μ (mm^{-1})	1.556
# measured refl	36258
# unique refl	5817
R_{int}	0.0613
# parameters	384
$R(F^2)$, all refl	0.0221
$R_w(F^2)$, all refl	0.0528
Goodness of fit	1.043

CCDC number 1545253.

8. NMR spectra

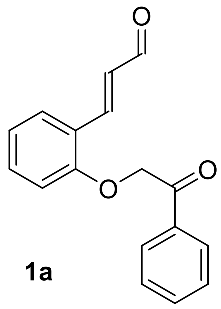

1a

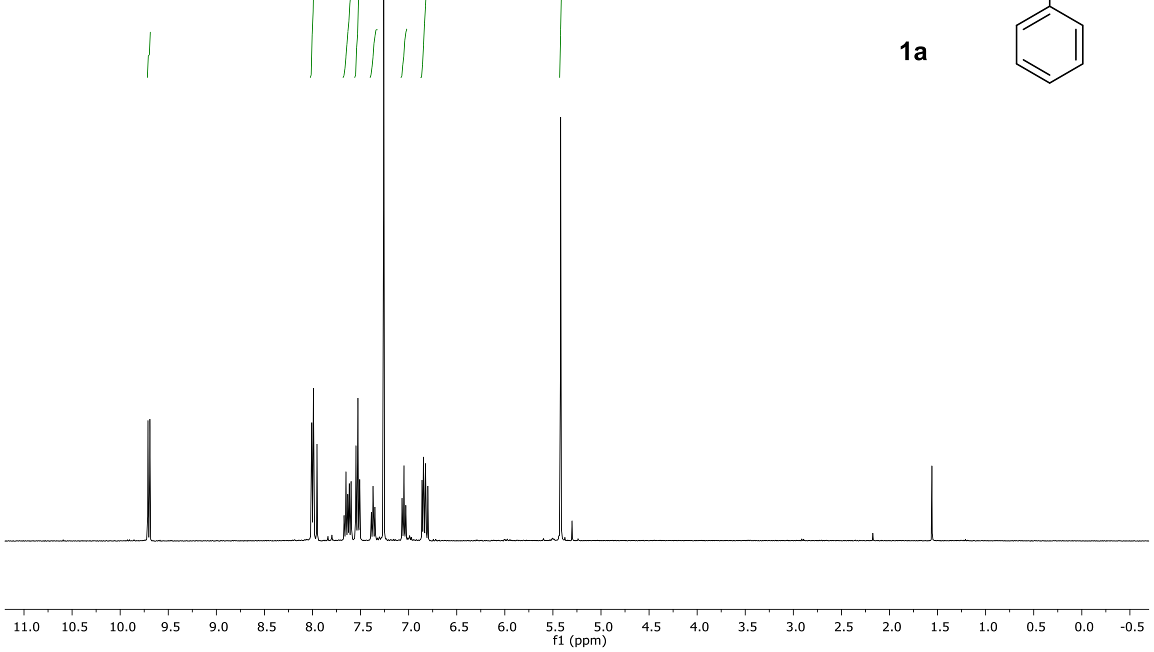

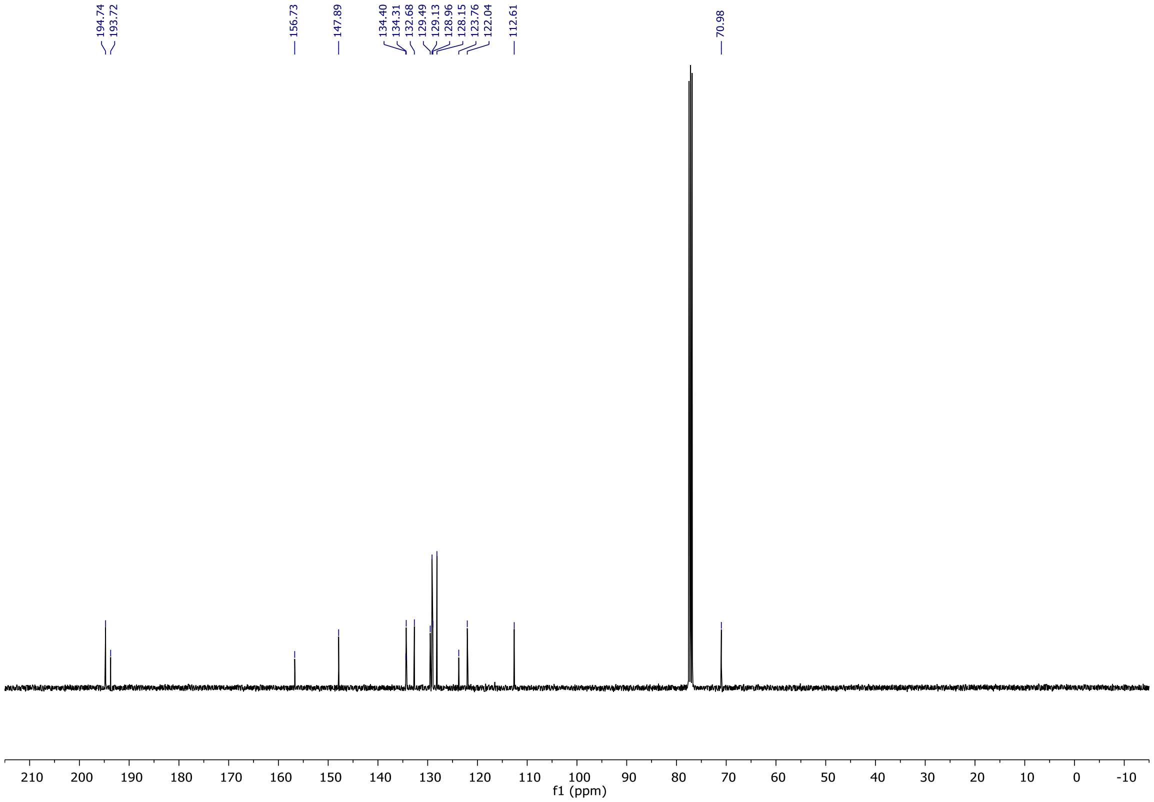

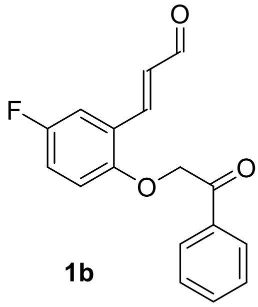
F
O
O
O

1b

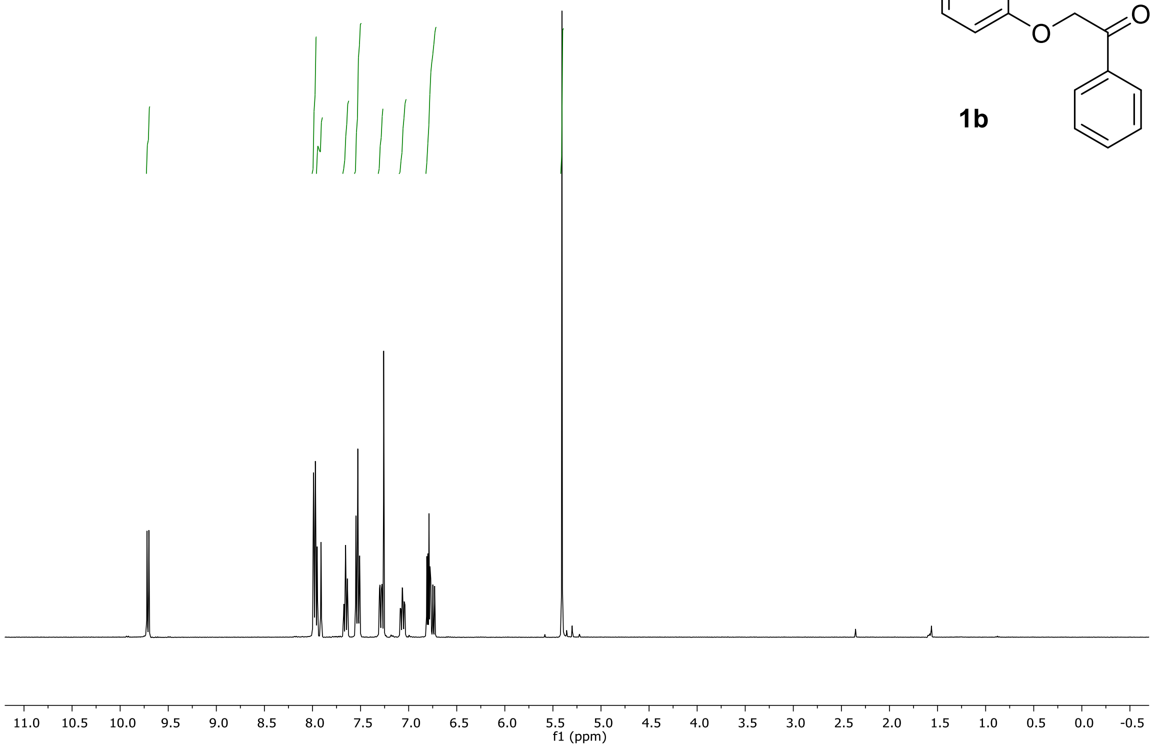
11.0 10.5 10.0 9.5 9.0 8.5 8.0 7.5 7.0 6.5 6.0 5.5 5.0 4.5 4.0 3.5 3.0 2.5 2.0 1.5 1.0 0.5 0.0 -0.5
f1 (ppm)

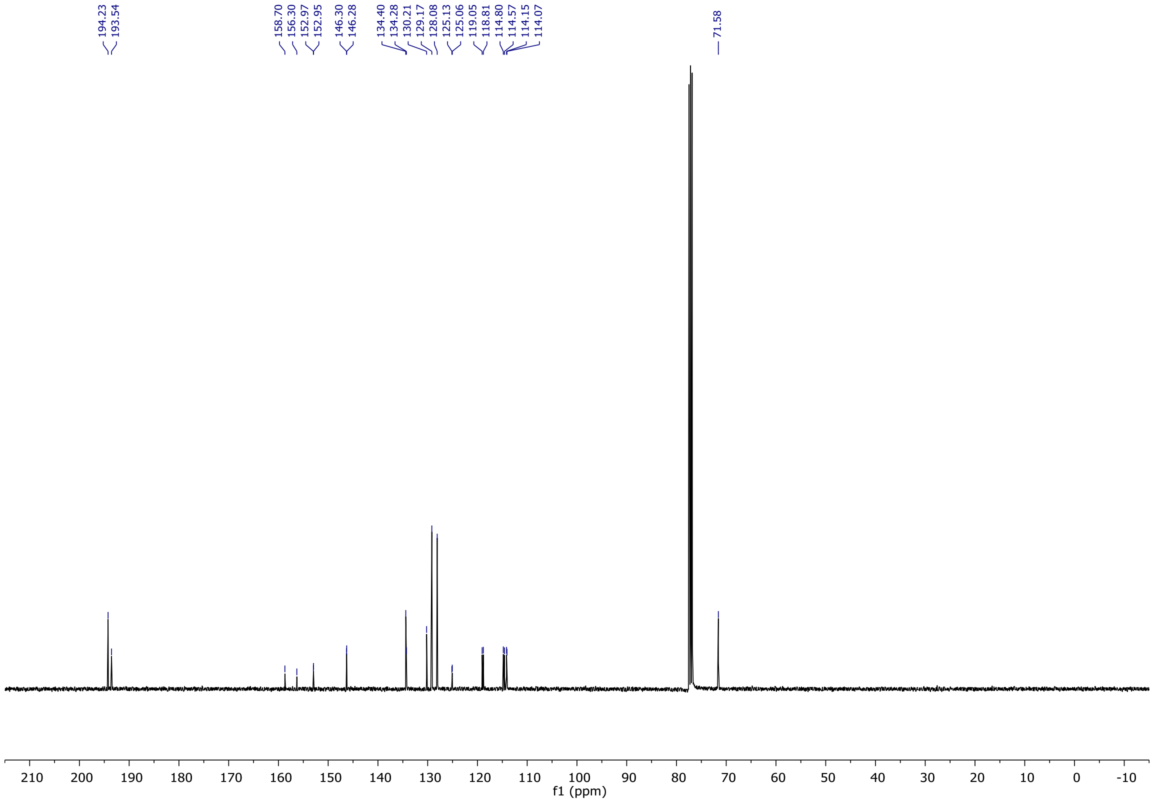
194.23
193.54
158.70
156.30
152.97
152.95
146.30
146.28
134.40
134.28
130.21
129.17
128.08
125.13
125.06
119.05
118.81
114.80
114.57
114.15
114.07
71.58
210 200 190 180 170 160 150 140 130 120 110 100 90 80 70 60 50 40 30 20 10 0 -10
f1 (ppm)

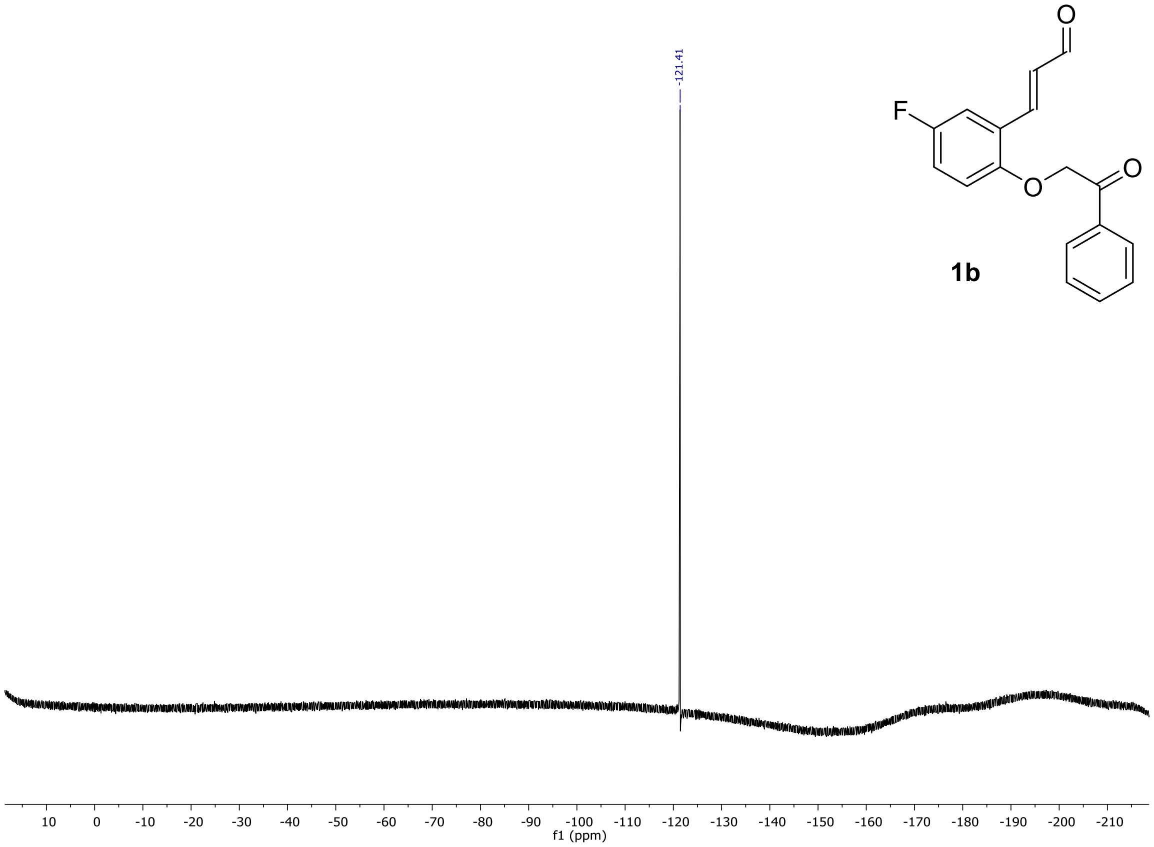
-121.41
F
O
O
O
1b
10
0
-10
-20
-30
-40
-50
-60
-70
-80
-90
-100
-110
-120
-130
-140
-150
-160
-170
-180
-190
-200
-210
f1 (ppm)

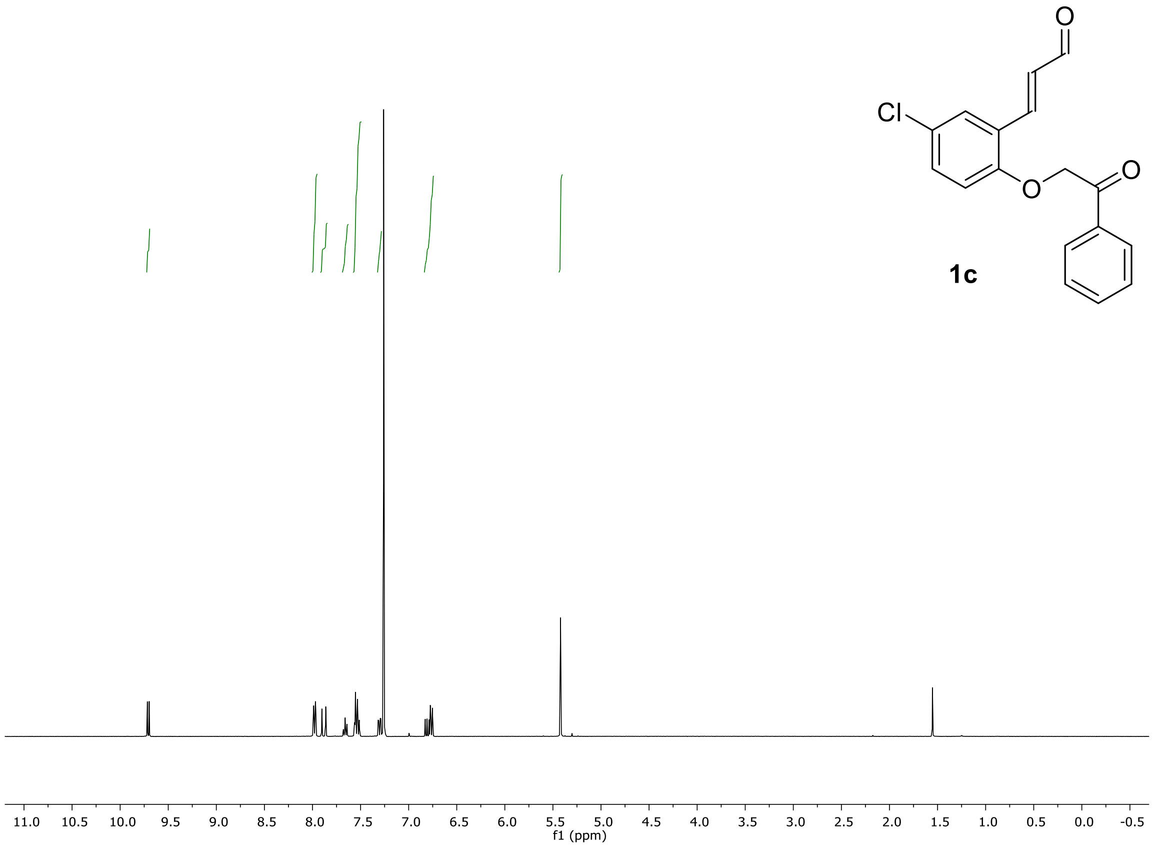
Cl
O
O
O
1c
11.0 10.5 10.0 9.5 9.0 8.5 8.0 7.5 7.0 6.5 6.0 5.5 5.0 4.5 4.0 3.5 3.0 2.5 2.0 1.5 1.0 0.5 0.0 -0.5
f1 (ppm)

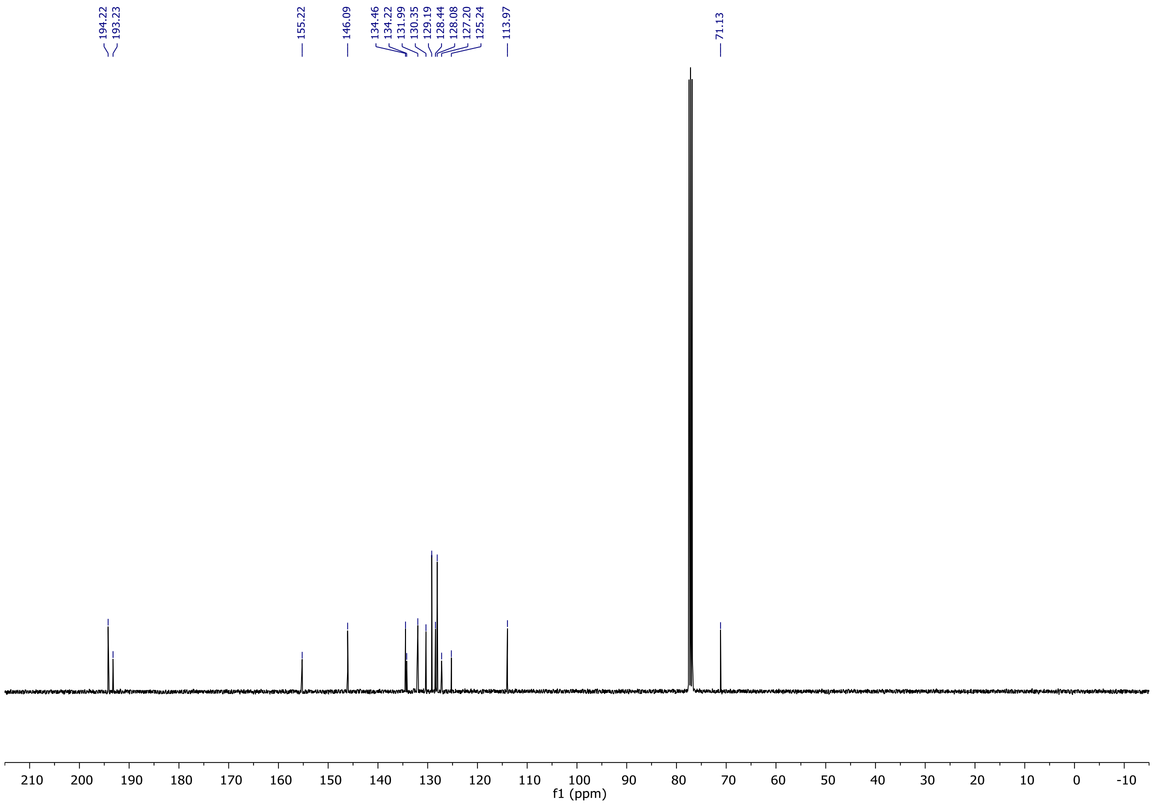
194.22
193.23
155.22
146.09
134.46
134.22
131.99
130.35
129.19
128.44
128.08
127.20
125.24
113.97
71.13
210 200 190 180 170 160 150 140 130 120 110 100 90 80 70 60 50 40 30 20 10 0 -10
f1 (ppm)

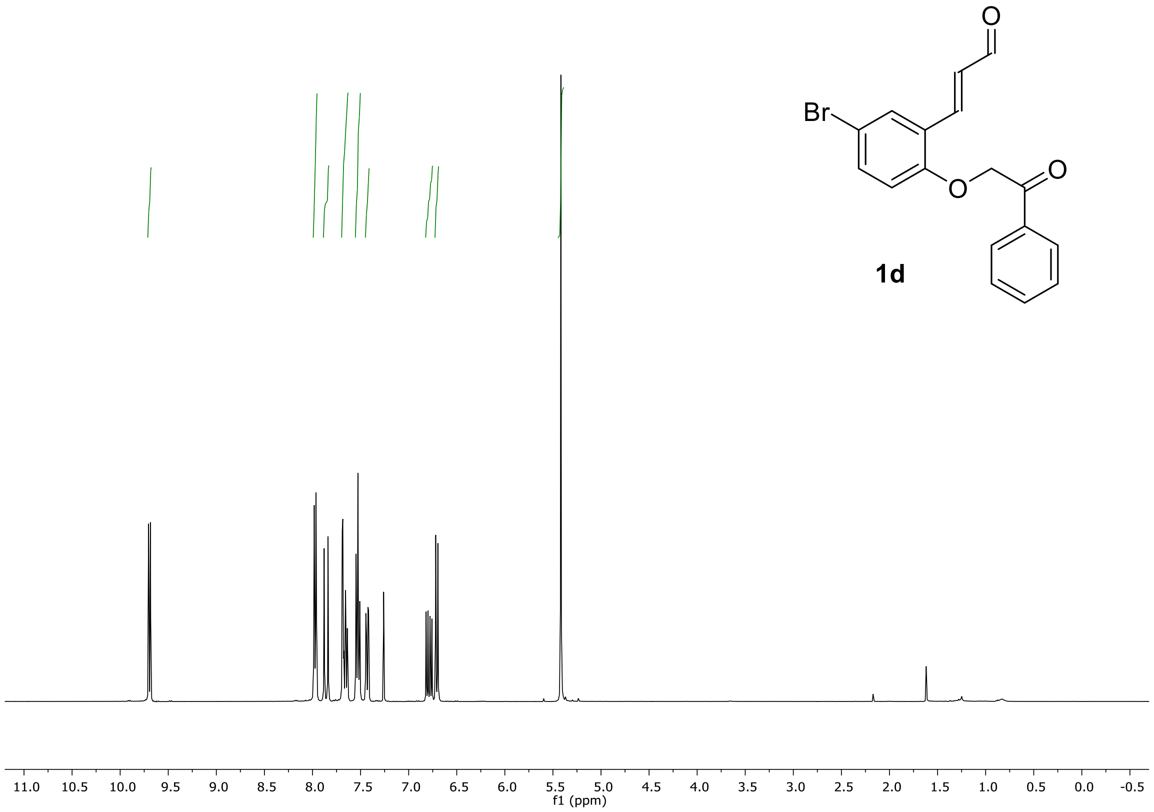
Br
O
O
O
11.0 10.5 10.0 9.5 9.0 8.5 8.0 7.5 7.0 6.5 6.0 5.5 5.0 4.5 4.0 3.5 3.0 2.5 2.0 1.5 1.0 0.5 0.0 -0.5
f1 (ppm)

1d

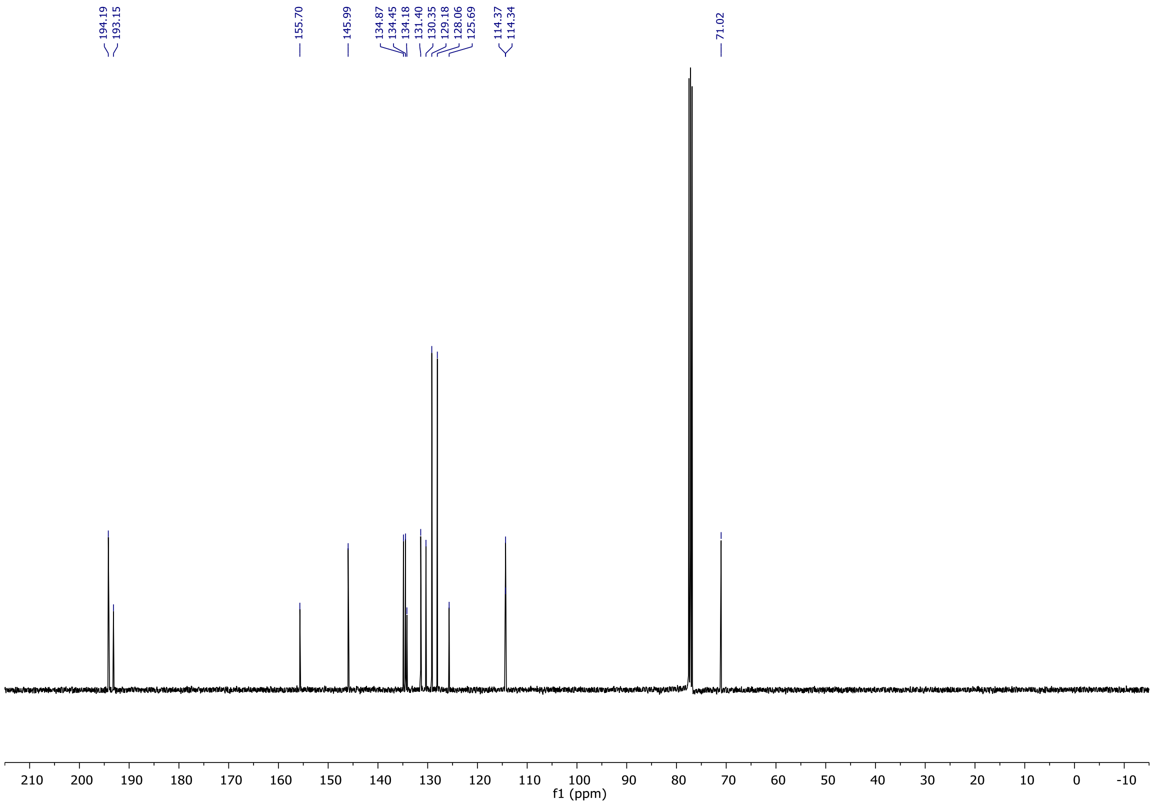
194.19
193.15
155.70
145.99
134.87
134.45
134.18
131.40
130.35
129.18
128.06
125.69
114.37
114.34
71.02
210 200 190 180 170 160 150 140 130 120 110 100 90 80 70 60 50 40 30 20 10 0 -10
f1 (ppm)

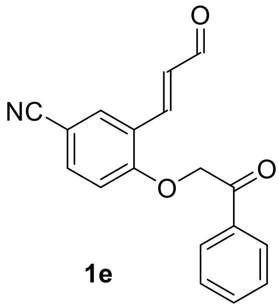
NC
O
O
O

1e

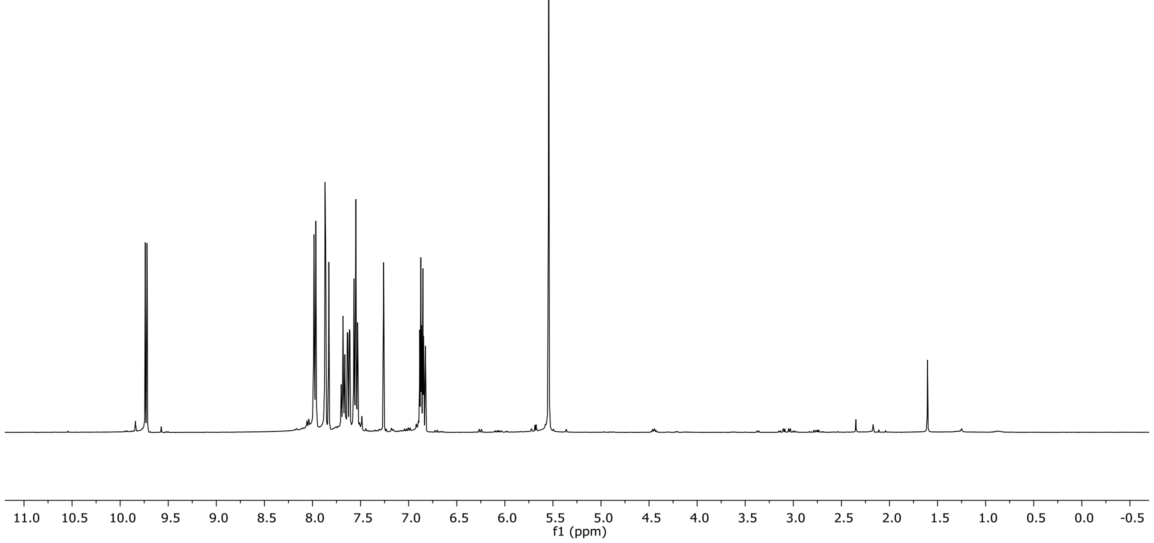
11.0 10.5 10.0 9.5 9.0 8.5 8.0 7.5 7.0 6.5 6.0 5.5 5.0 4.5 4.0 3.5 3.0 2.5 2.0 1.5 1.0 0.5 0.0 -0.5
f1 (ppm)

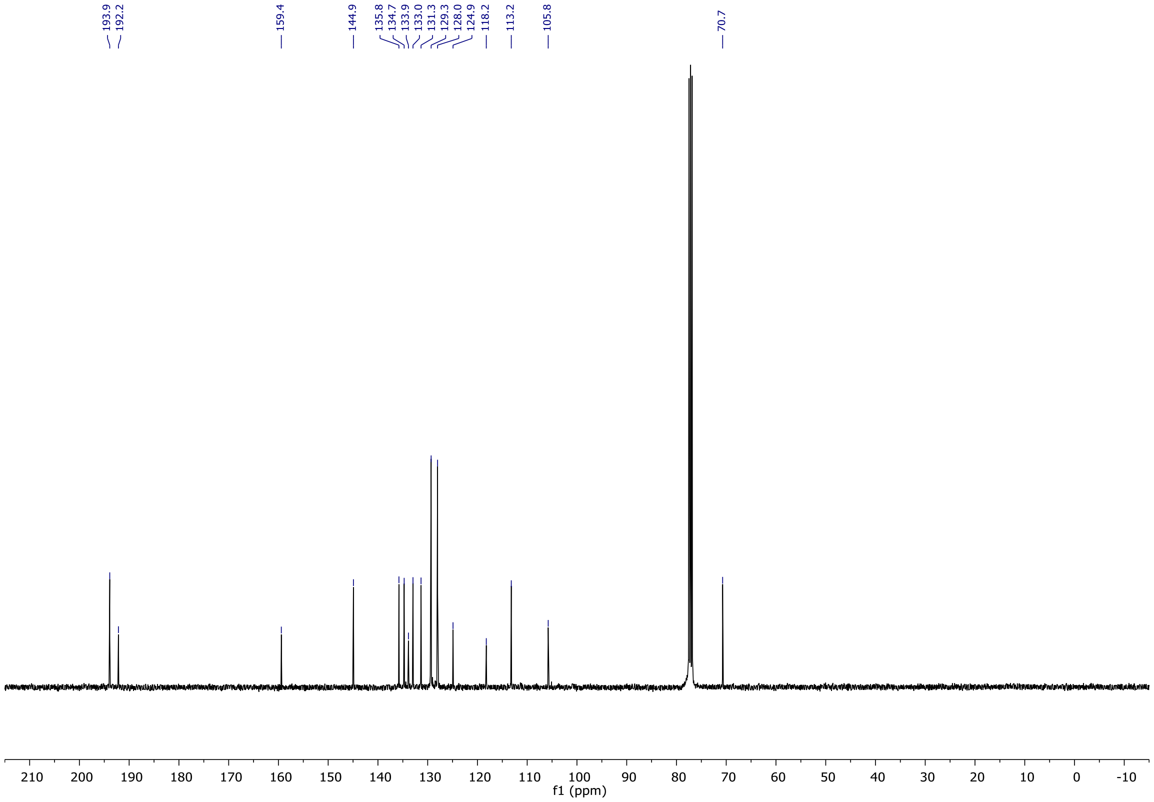
193.9
192.2
159.4
144.9
135.8
134.7
133.9
133.0
131.3
129.3
128.0
124.9
118.2
113.2
105.8
70.7
210 200 190 180 170 160 150 140 130 120 110 100 90 80 70 60 50 40 30 20 10 0 -10
f1 (ppm)

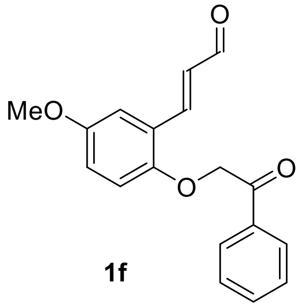

1f

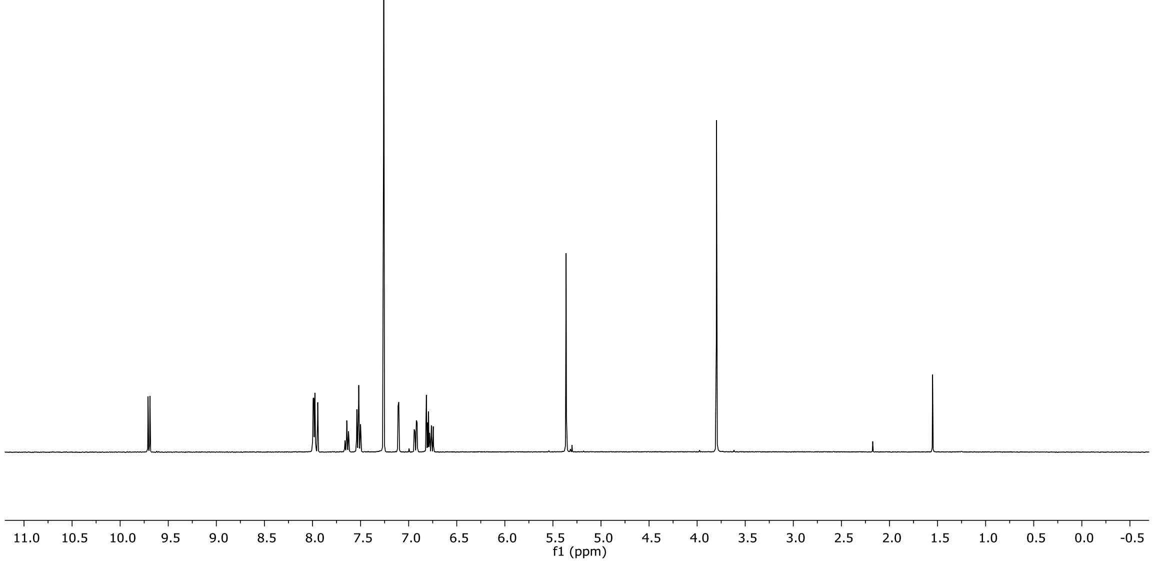
11.0 10.5 10.0 9.5 9.0 8.5 8.0 7.5 7.0 6.5 6.0 5.5 5.0 4.5 4.0 3.5 3.0 2.5 2.0 1.5 1.0 0.5 0.0 -0.5
f1 (ppm)

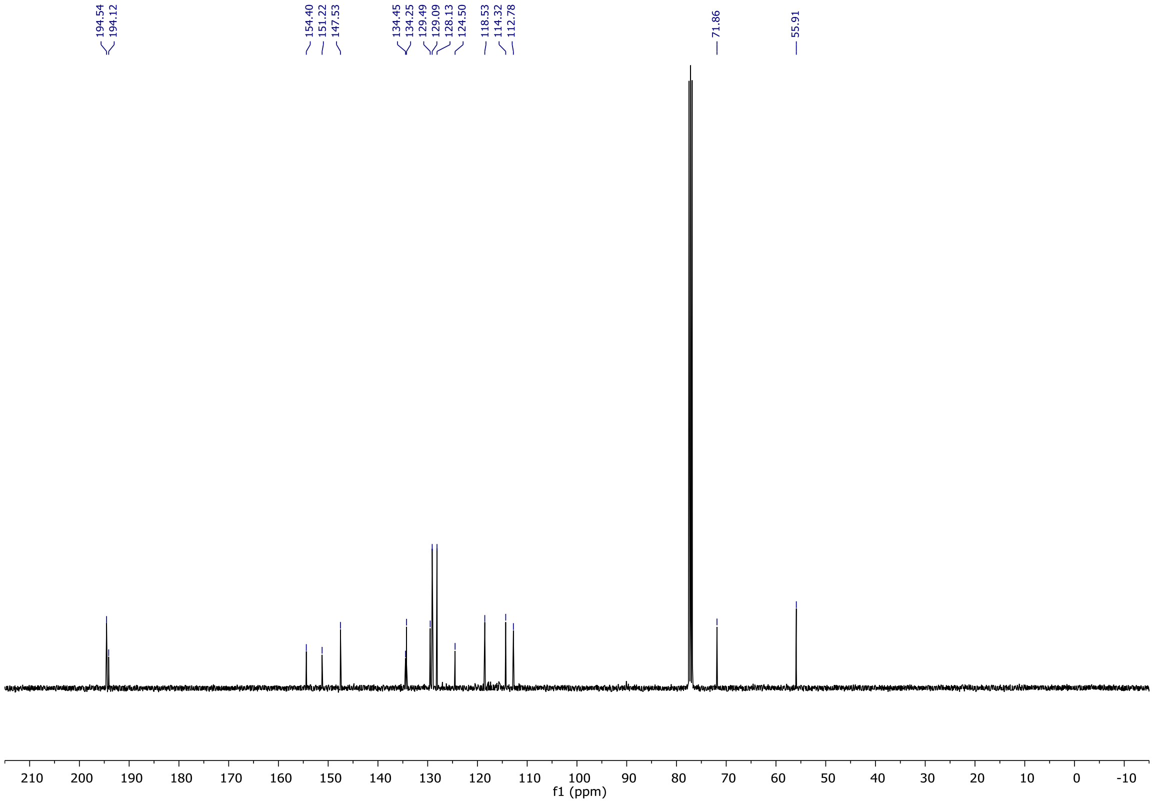
194.54
194.12
154.40
151.22
147.53
134.45
134.25
129.49
129.09
128.13
124.50
118.53
114.32
112.78
71.86
55.91
210 200 190 180 170 160 150 140 130 120 110 100 90 80 70 60 50 40 30 20 10 0 -10
f1 (ppm)

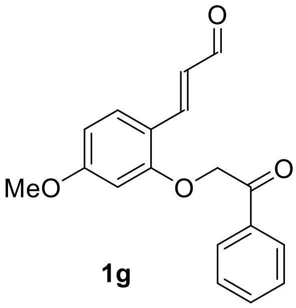
O
MeO
O
O

1g

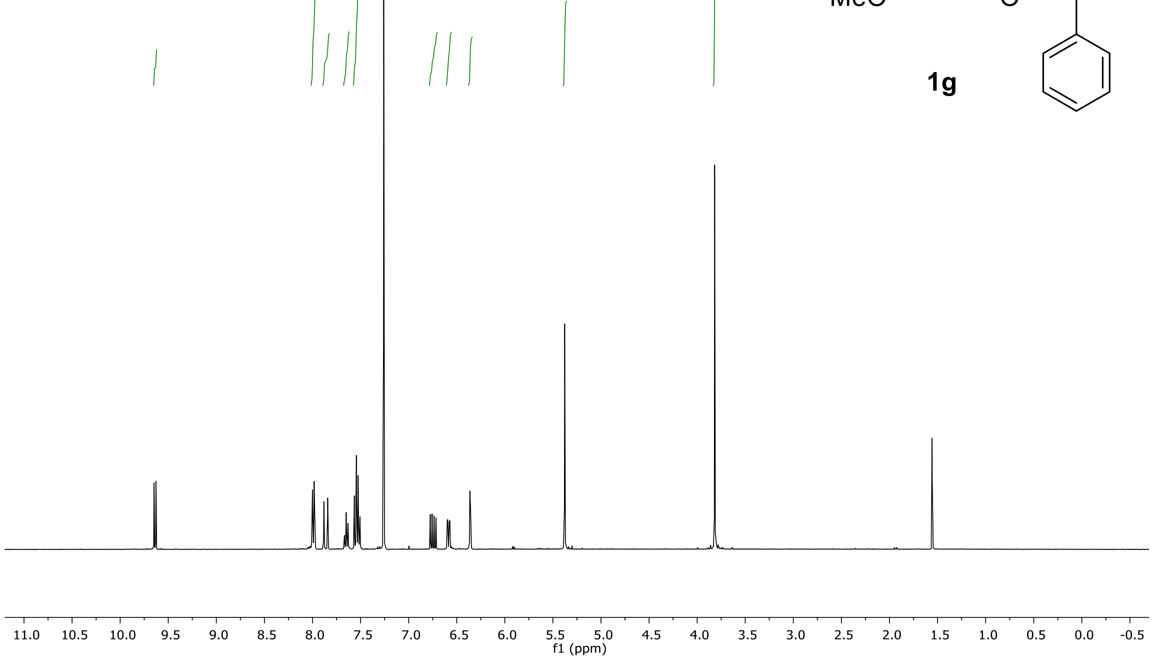
11.0 10.5 10.0 9.5 9.0 8.5 8.0 7.5 7.0 6.5 6.0 5.5 5.0 4.5 4.0 3.5 3.0 2.5 2.0 1.5 1.0 0.5 0.0 -0.5
f1 (ppm)

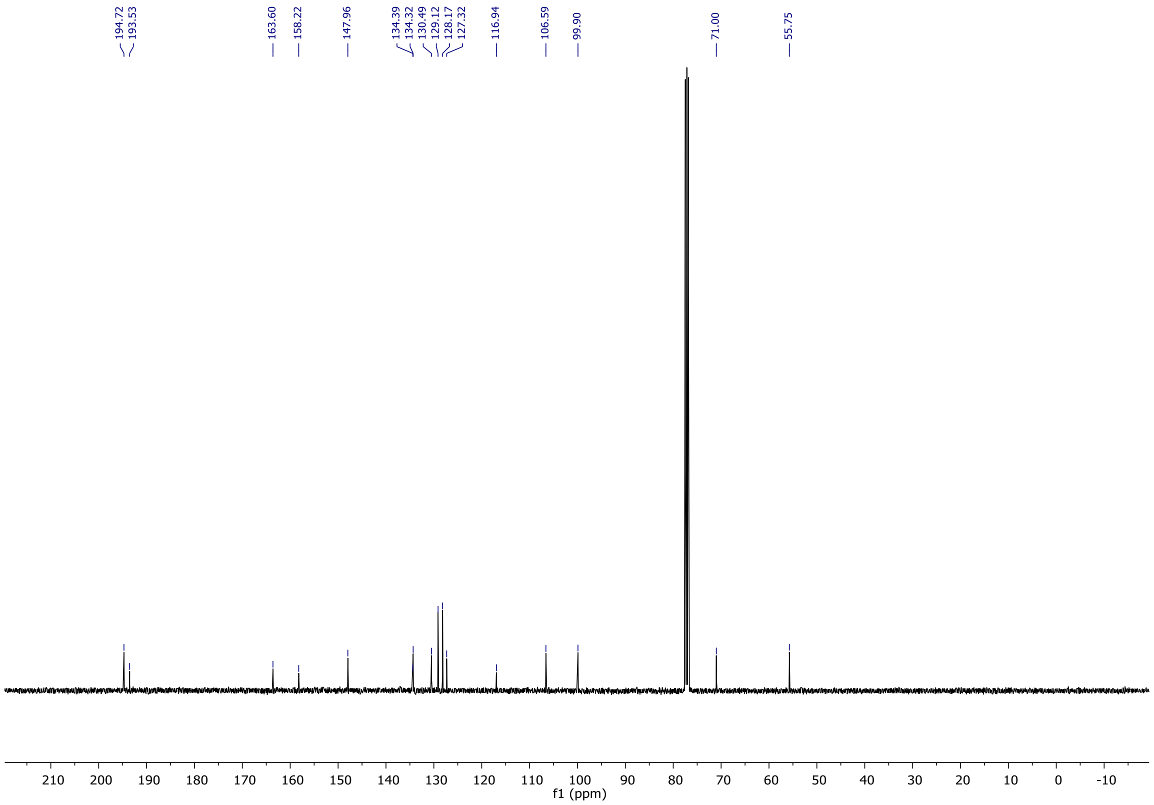
194.72
193.53
163.60
158.22
147.96
134.39
134.32
130.49
129.12
128.17
127.32
116.94
106.59
99.90
71.00
55.75
210 200 190 180 170 160 150 140 130 120 110 100 90 80 70 60 50 40 30 20 10 0 -10
f1 (ppm)

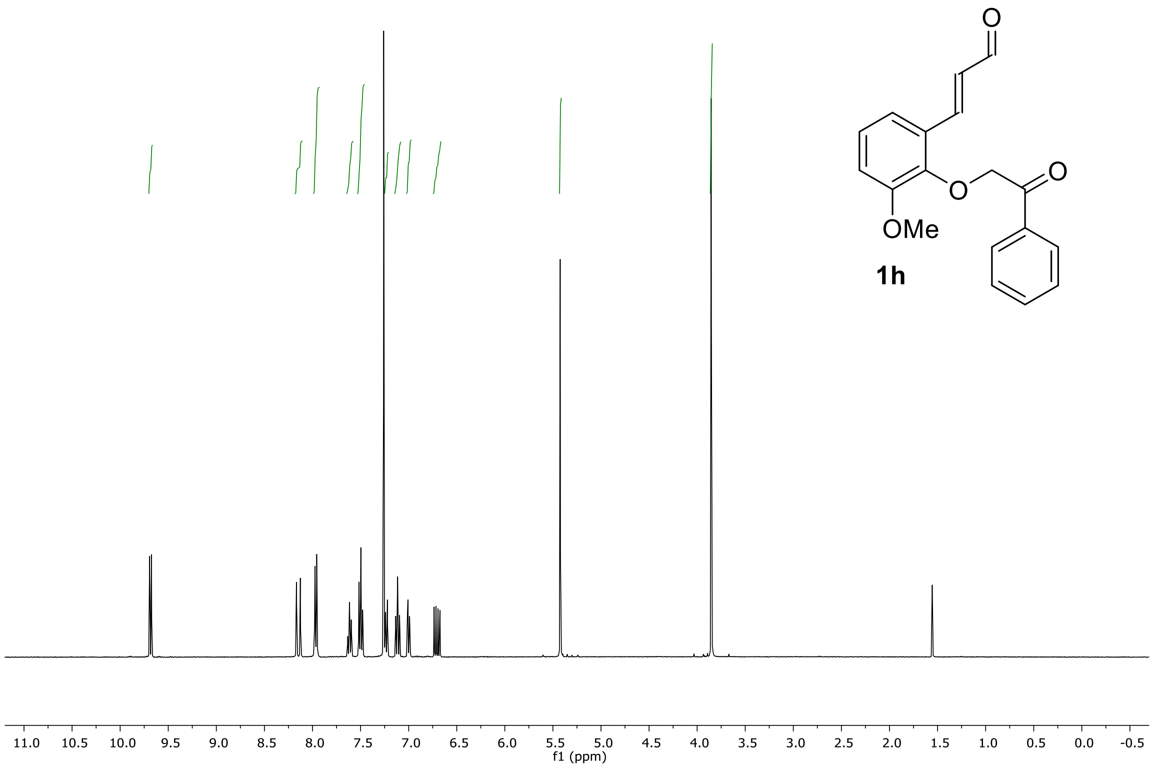
11.0 10.5 10.0 9.5 9.0 8.5 8.0 7.5 7.0 6.5 6.0 5.5 5.0 4.5 4.0 3.5 3.0 2.5 2.0 1.5 1.0 0.5 0.0 -0.5
f1 (ppm)
O
O
O
OMe

1h

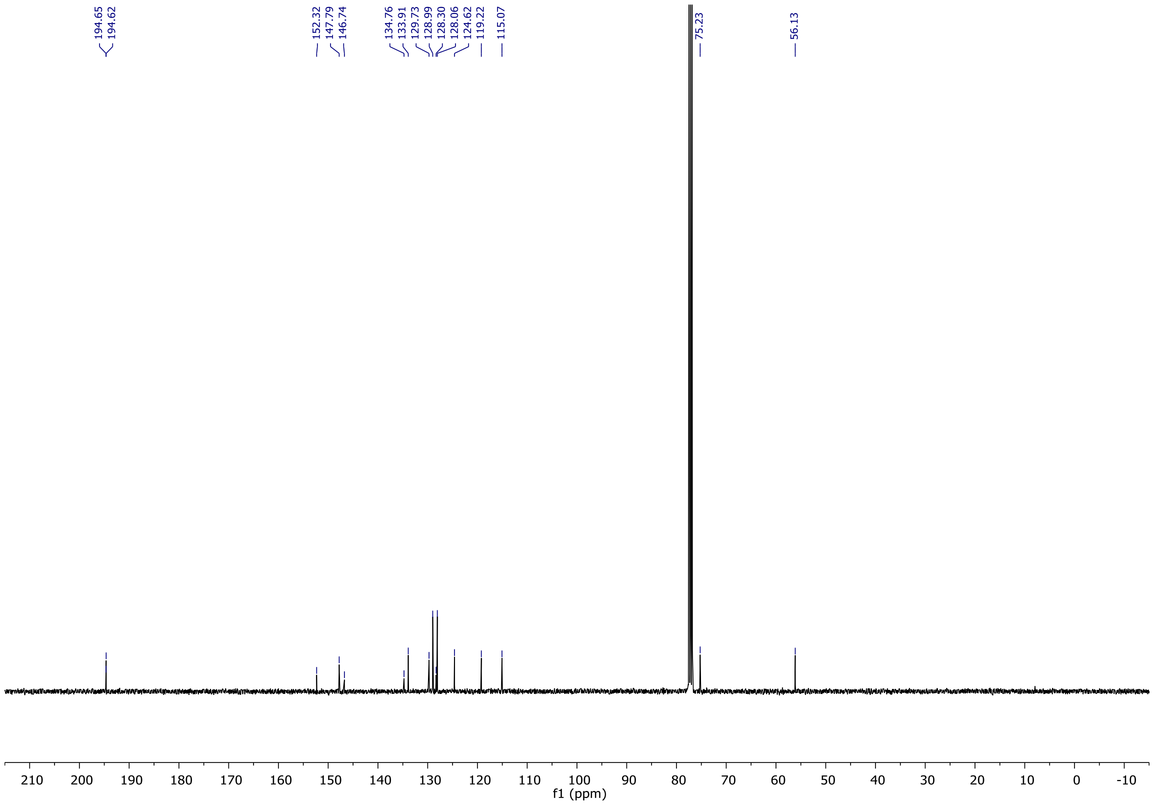
194.65
194.62
152.32
147.79
146.74
134.76
133.91
129.73
128.99
128.30
128.06
124.62
119.22
115.07
75.23
56.13
210 200 190 180 170 160 150 140 130 120 110 100 90 80 70 60 50 40 30 20 10 0 -10
f1 (ppm)

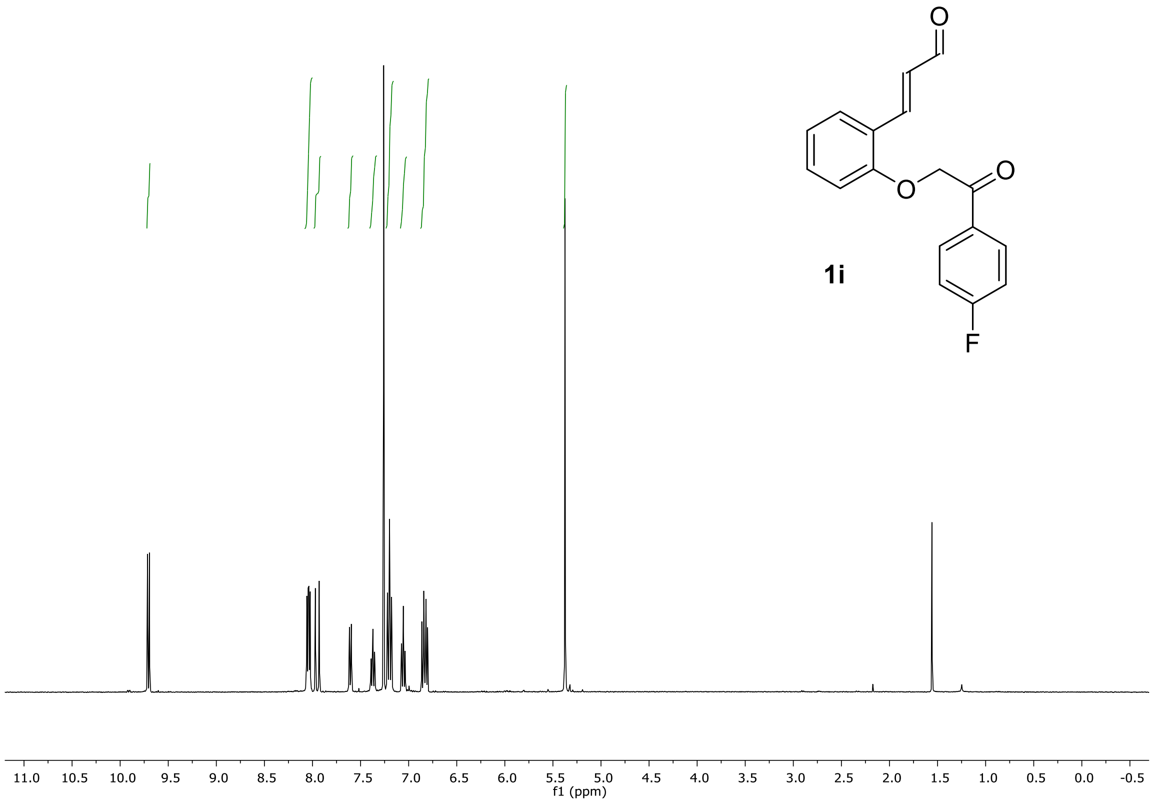
11.0 10.5 10.0 9.5 9.0 8.5 8.0 7.5 7.0 6.5 6.0 5.5 5.0 4.5 4.0 3.5 3.0 2.5 2.0 1.5 1.0 0.5 0.0 -0.5
f1 (ppm)
O
O
O
F

1i

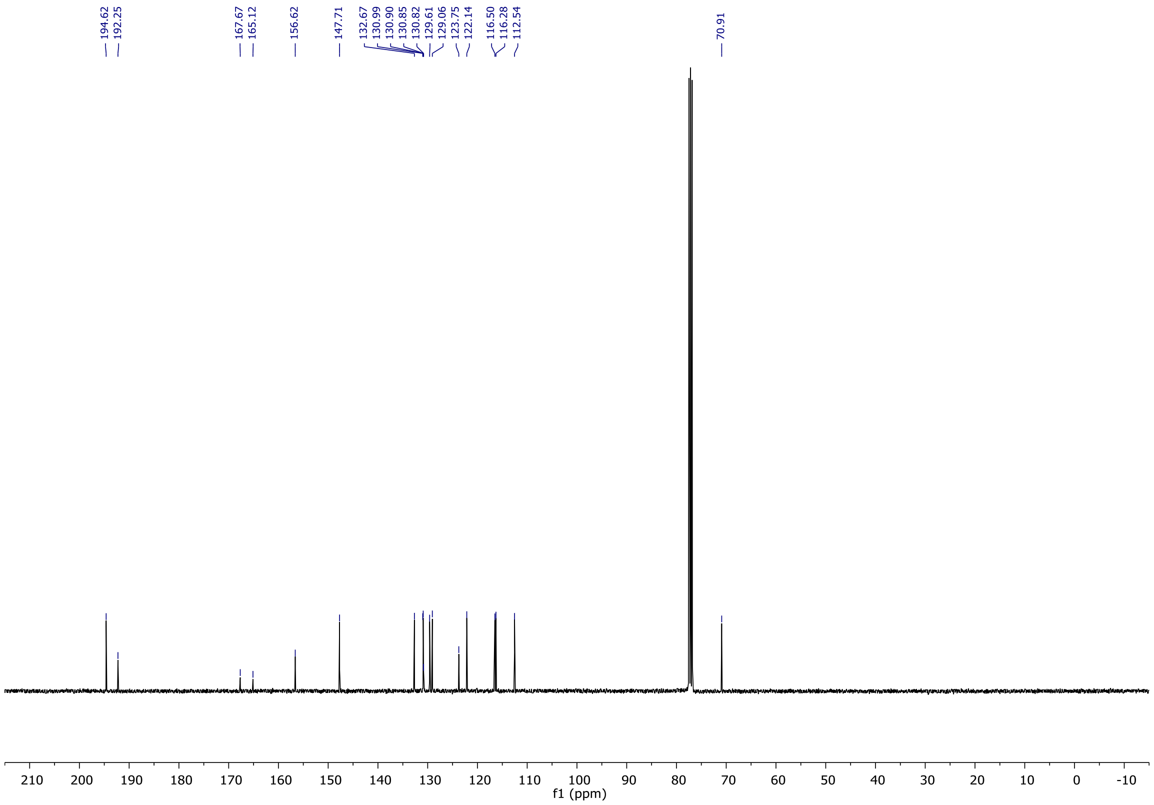
194.62
192.25
167.67
165.12
156.62
147.71
132.67
130.99
130.90
130.85
130.82
129.61
129.06
123.75
122.14
116.50
116.28
112.54
70.91
210 200 190 180 170 160 150 140 130 120 110 100 90 80 70 60 50 40 30 20 10 0 -10
f1 (ppm)

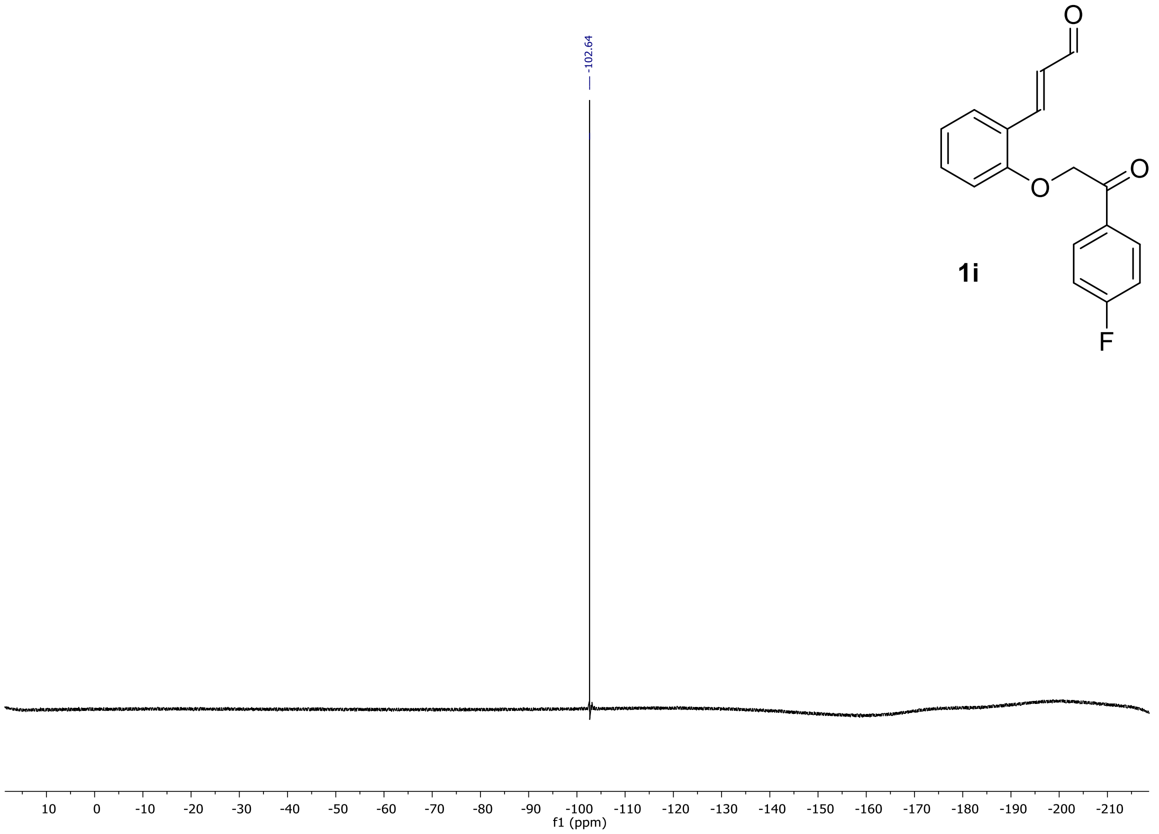
-102.64
10 0 -10 -20 -30 -40 -50 -60 -70 -80 -90 -100 -110 -120 -130 -140 -150 -160 -170 -180 -190 -200 -210
f1 (ppm)
1i

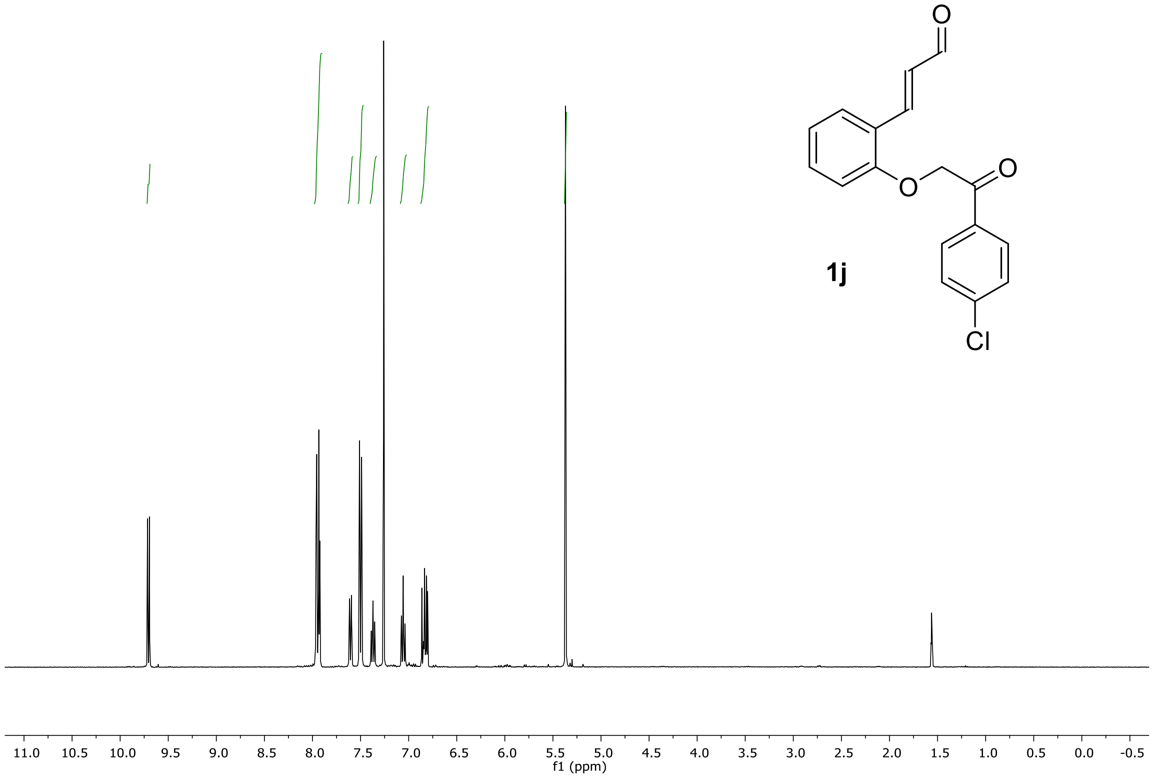
1j
f1 (ppm)

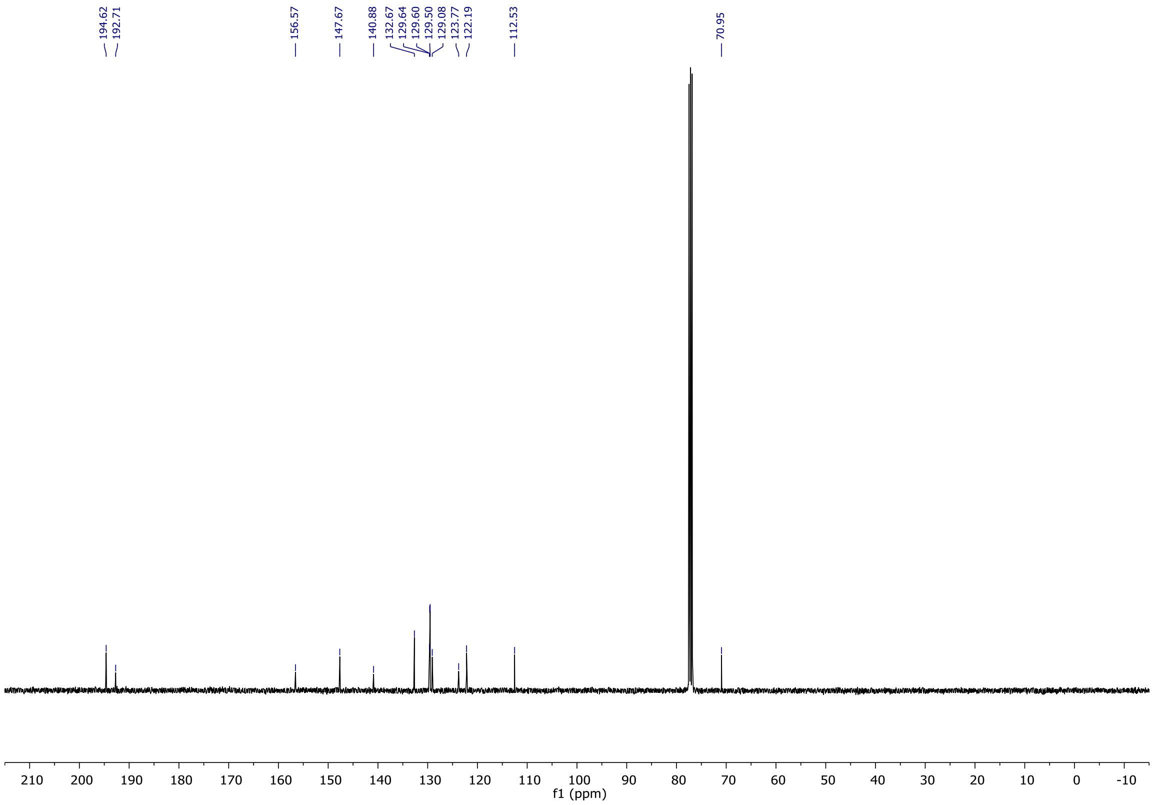
194.62
192.71
156.57
147.67
140.88
132.67
129.64
129.60
129.50
129.08
123.77
122.19
112.53
70.95
f1 (ppm)

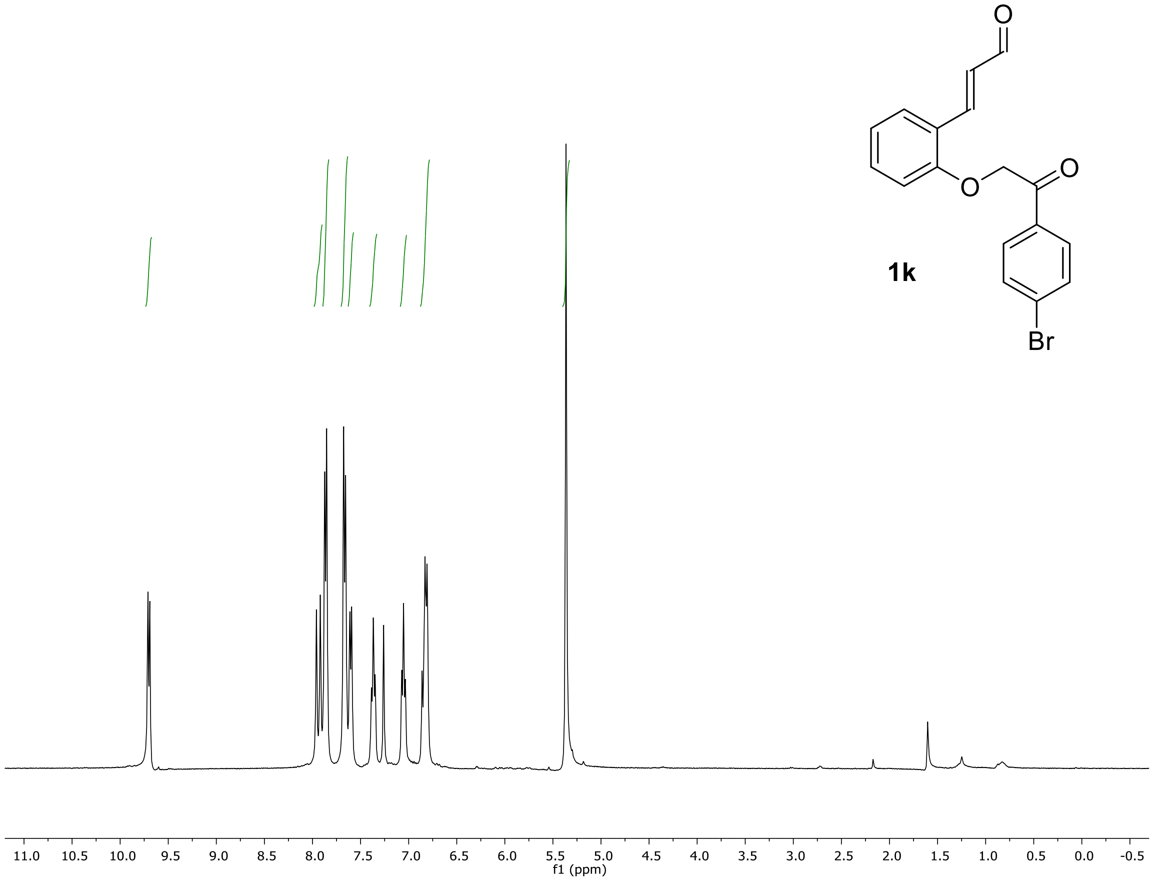

1k
11.0 10.5 10.0 9.5 9.0 8.5 8.0 7.5 7.0 6.5 6.0 5.5 5.0 4.5 4.0 3.5 3.0 2.5 2.0 1.5 1.0 0.5 0.0 -0.5
f1 (ppm)

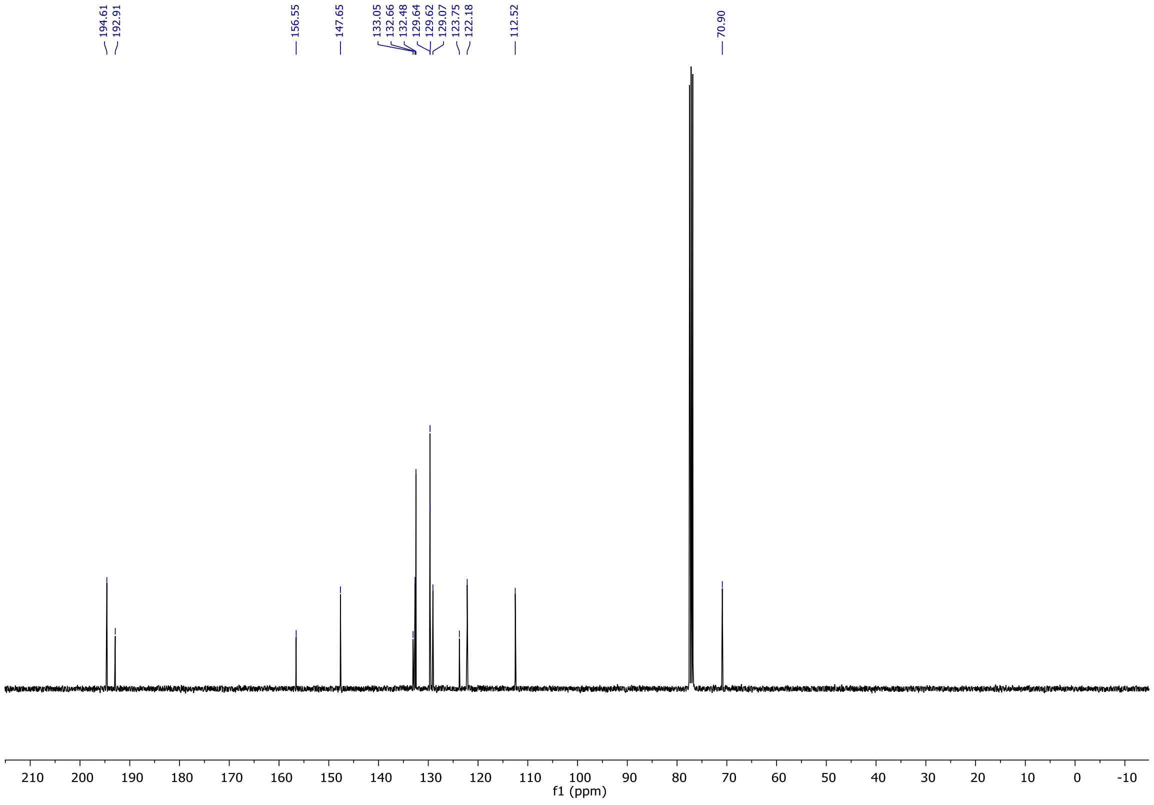
194.61
192.91
156.55
147.65
133.05
132.66
132.48
129.64
129.62
129.07
123.75
122.18
112.52
70.90
210 200 190 180 170 160 150 140 130 120 110 100 90 80 70 60 50 40 30 20 10 0 -10
f1 (ppm)

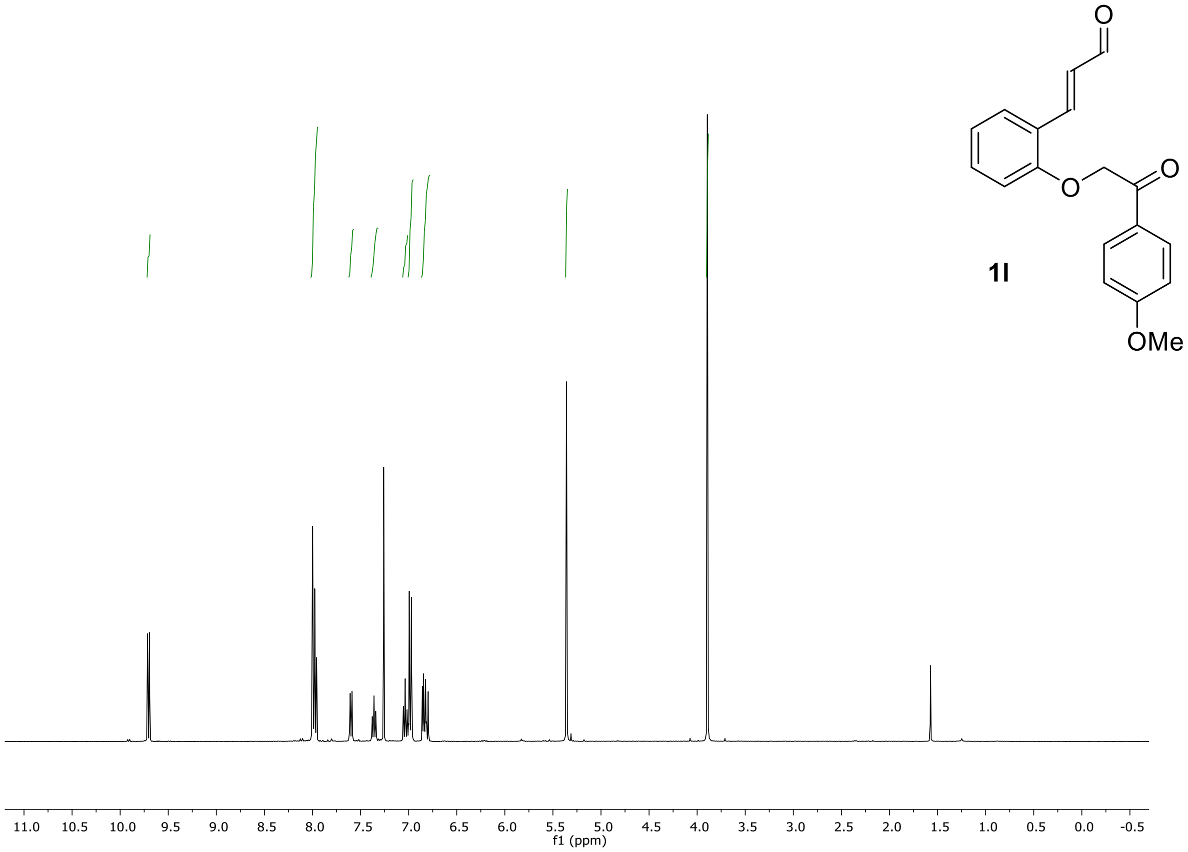
O
O
O
OMe
1l
f1 (ppm)

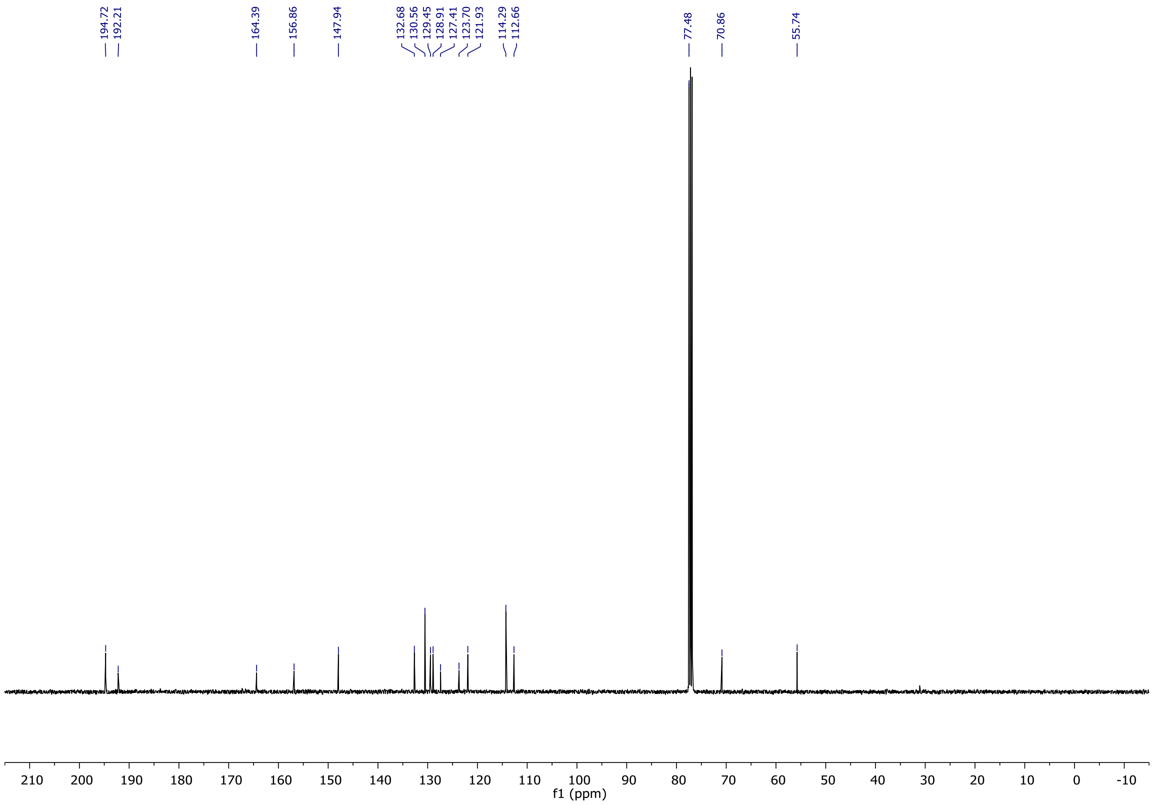
194.72
192.21
164.39
156.86
147.94
132.68
130.56
129.45
128.91
127.41
123.70
121.93
114.29
112.66
77.48
70.86
55.74
f1 (ppm)

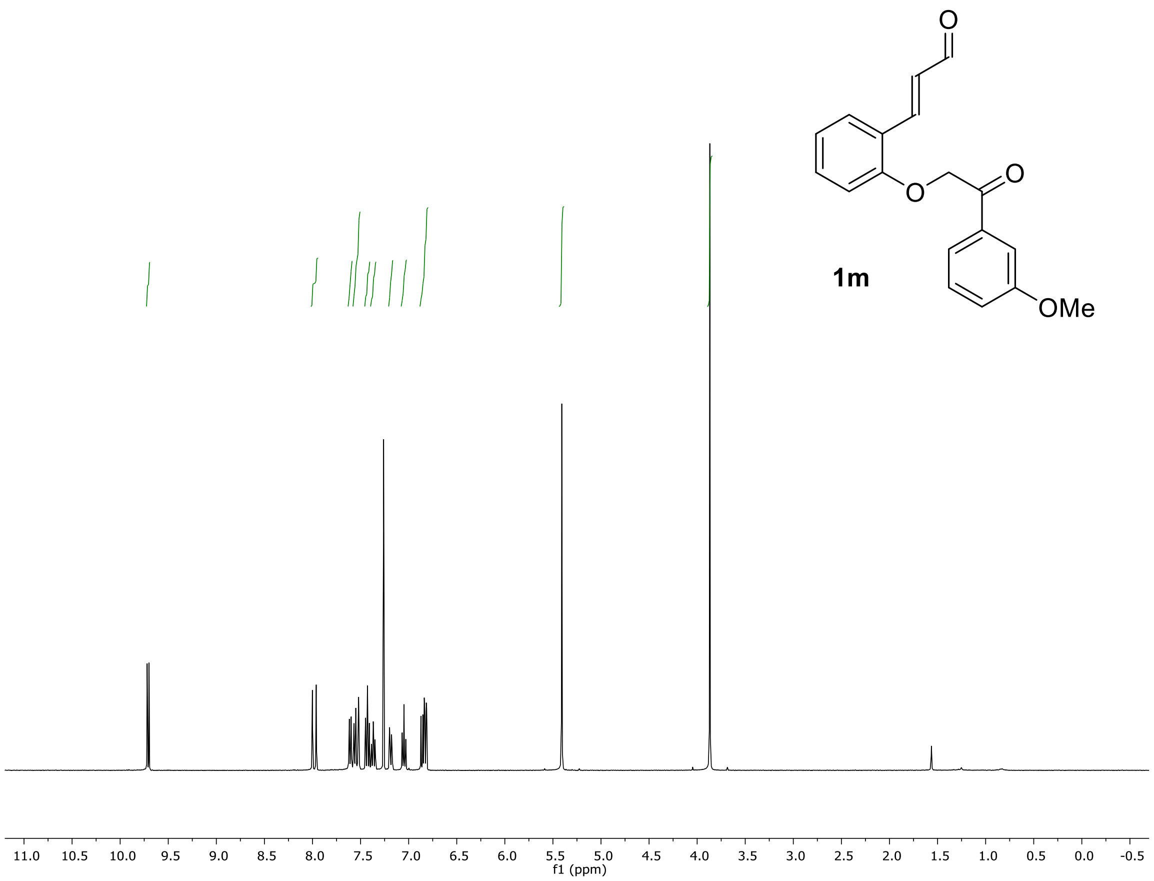
1m
OMe
11.0 10.5 10.0 9.5 9.0 8.5 8.0 7.5 7.0 6.5 6.0 5.5 5.0 4.5 4.0 3.5 3.0 2.5 2.0 1.5 1.0 0.5 0.0 -0.5
f1 (ppm)

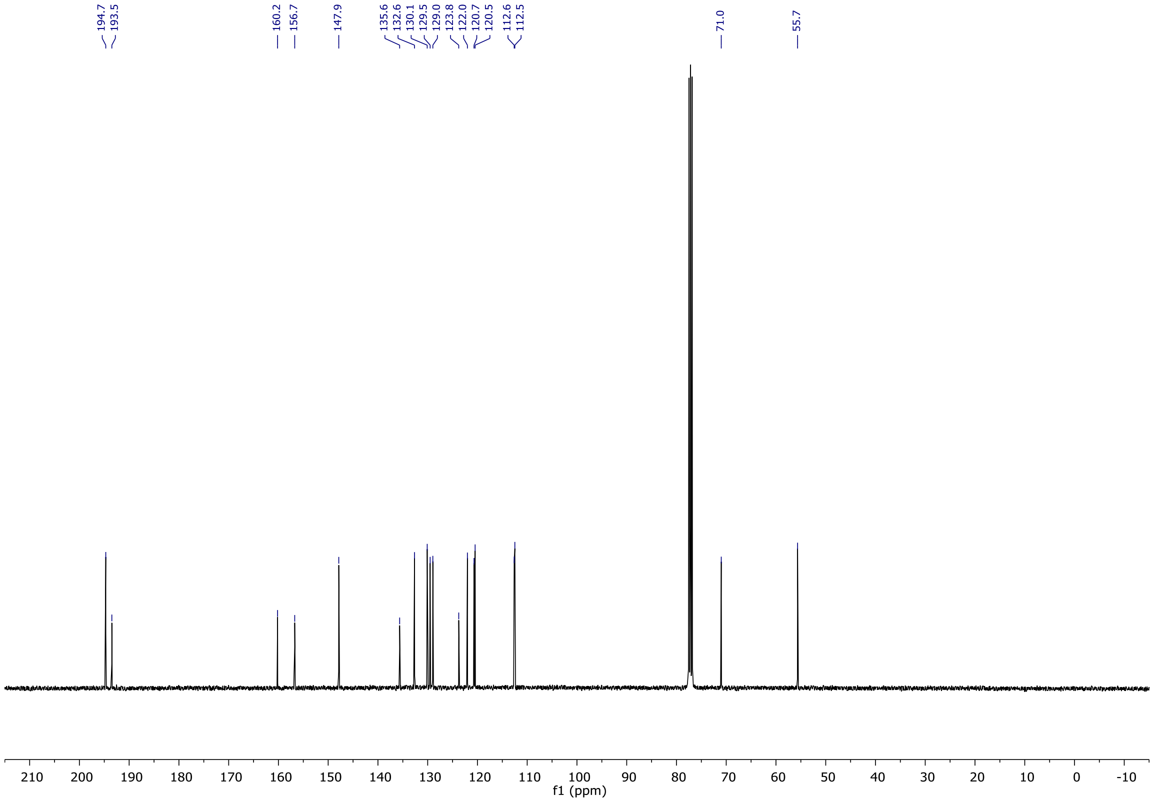
194.7
193.5
160.2
156.7
147.9
135.6
132.6
130.1
129.5
129.0
123.8
122.0
120.7
120.5
112.6
112.5
71.0
55.7
210 200 190 180 170 160 150 140 130 120 110 100 90 80 70 60 50 40 30 20 10 0 -10
f1 (ppm)

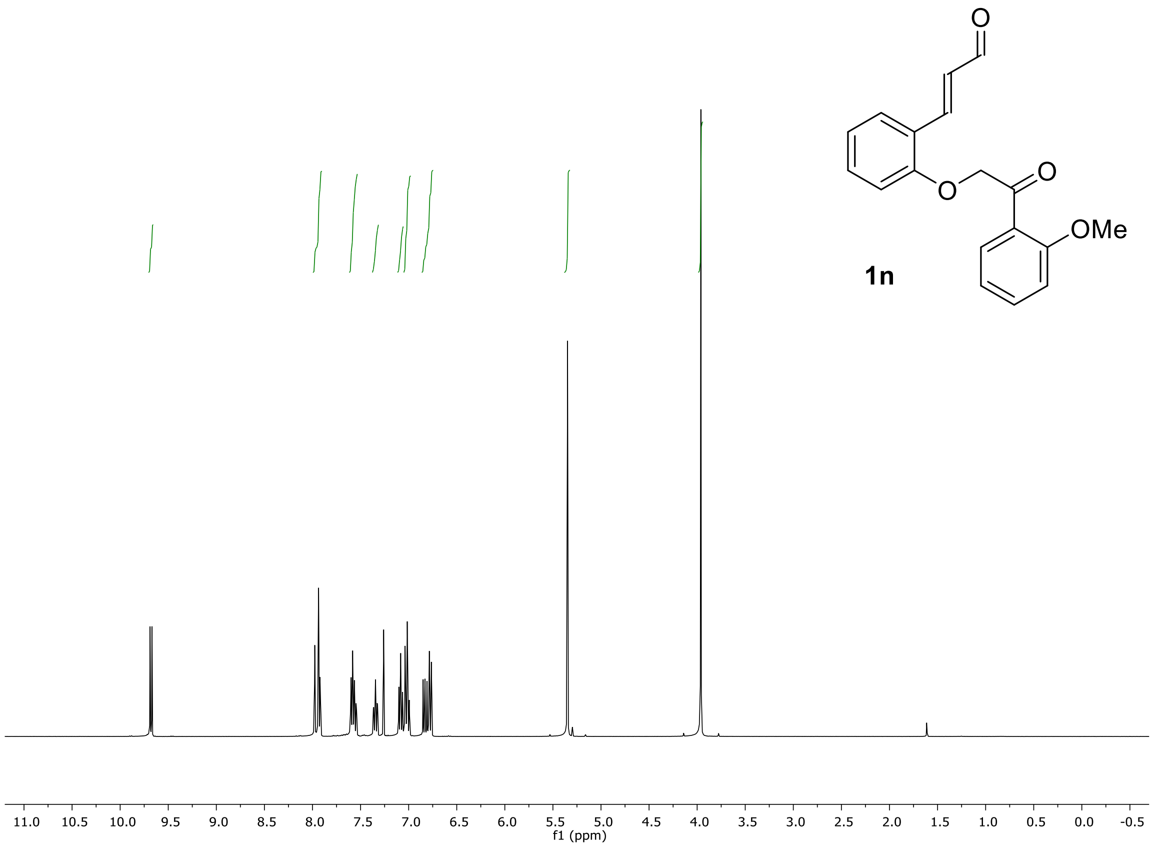
O
O
O
OMe
1n
11.0 10.5 10.0 9.5 9.0 8.5 8.0 7.5 7.0 6.5 6.0 5.5 5.0 4.5 4.0 3.5 3.0 2.5 2.0 1.5 1.0 0.5 0.0 -0.5
f1 (ppm)

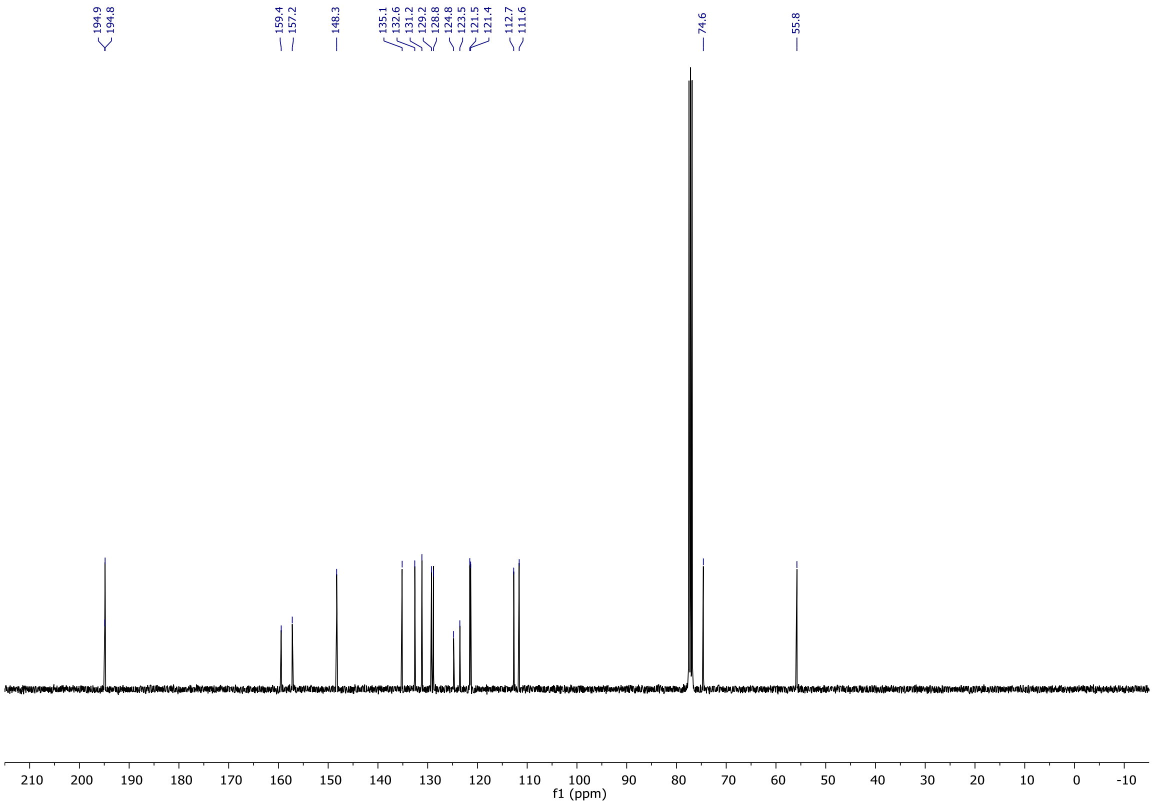
194.9
194.8
159.4
157.2
148.3
135.1
132.6
131.2
129.2
128.8
124.8
123.5
121.5
121.4
112.7
111.6
74.6
55.8
210 200 190 180 170 160 150 140 130 120 110 100 90 80 70 60 50 40 30 20 10 0 -10
f1 (ppm)

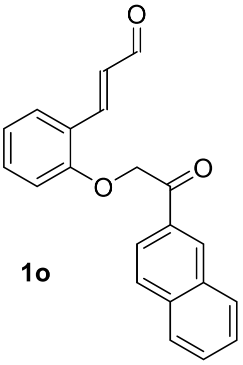
O
O
O

1o

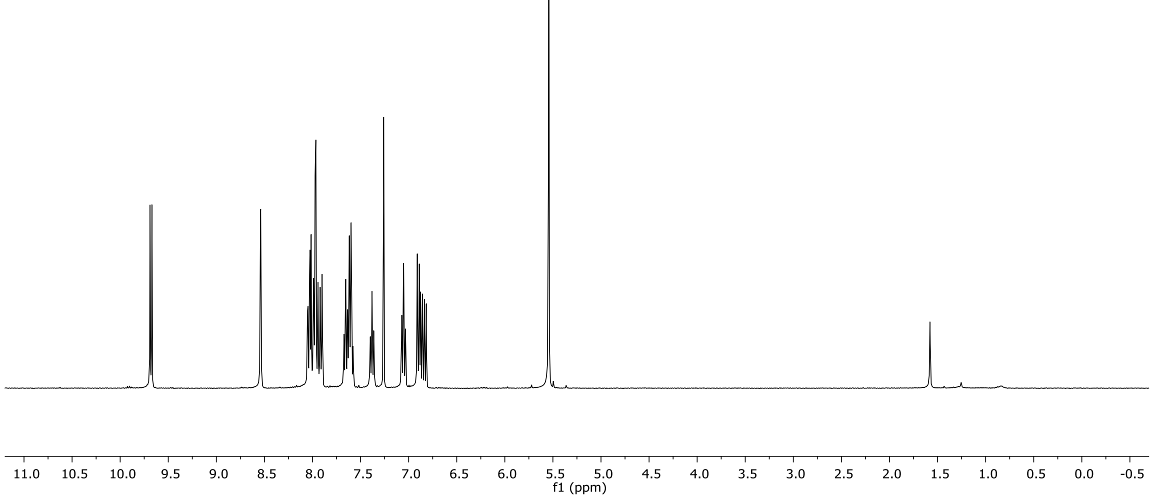
f1 (ppm)

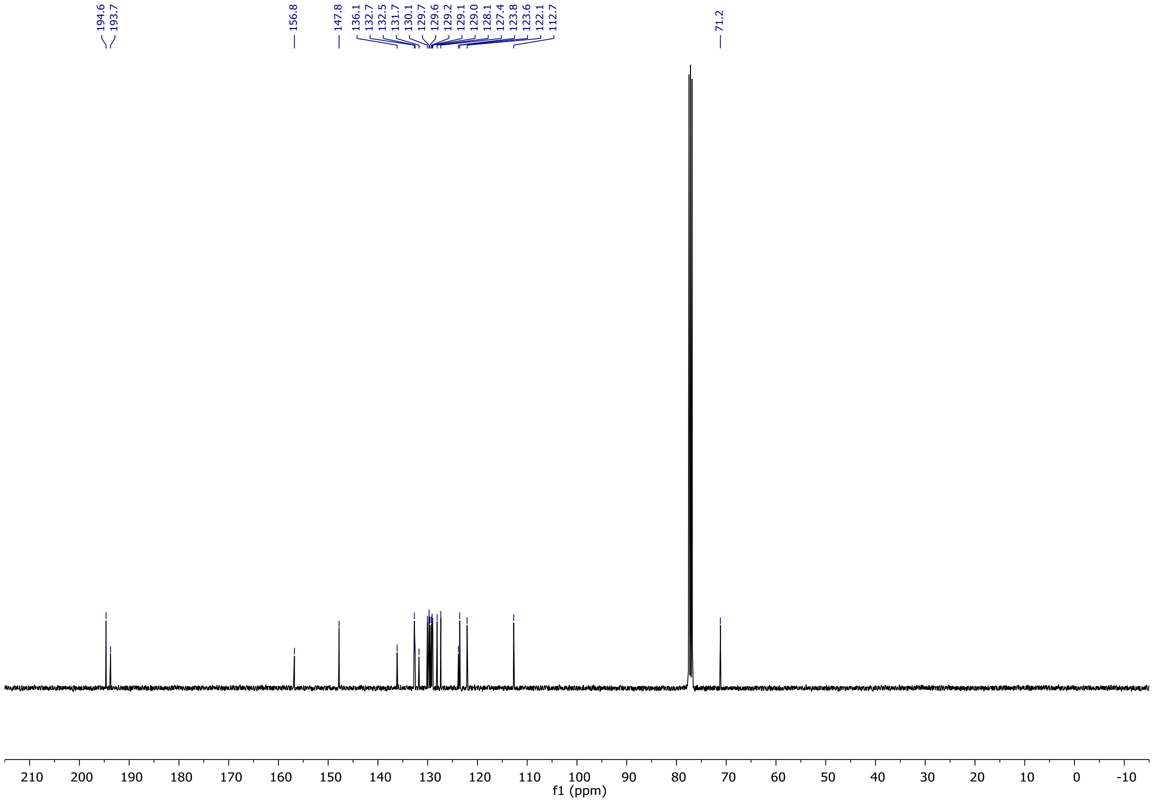
194.6
193.7
156.8
147.8
136.1
132.7
132.5
131.7
130.1
129.7
129.6
129.2
129.1
129.0
128.1
127.4
123.8
123.6
122.1
112.7
71.2
f1 (ppm)

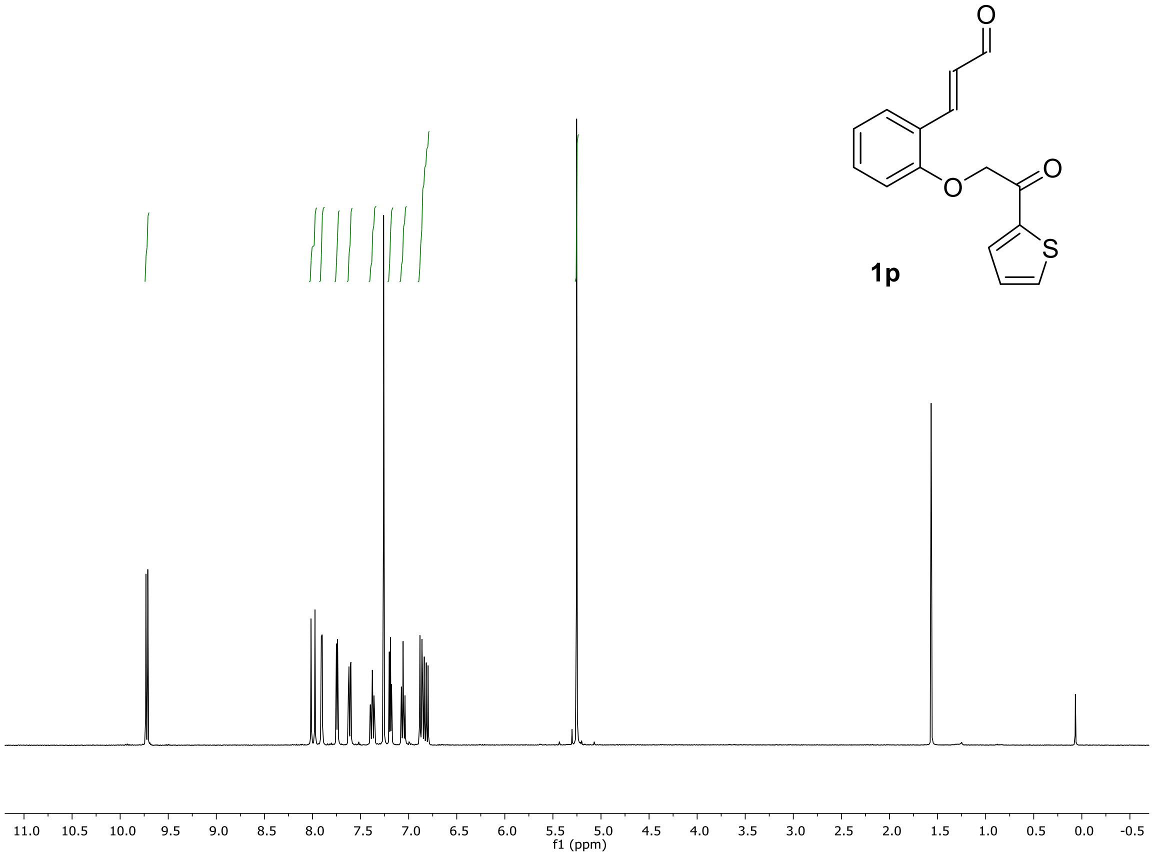
1p
11.0 10.5 10.0 9.5 9.0 8.5 8.0 7.5 7.0 6.5 6.0 5.5 5.0 4.5 4.0 3.5 3.0 2.5 2.0 1.5 1.0 0.5 0.0 -0.5
f1 (ppm)

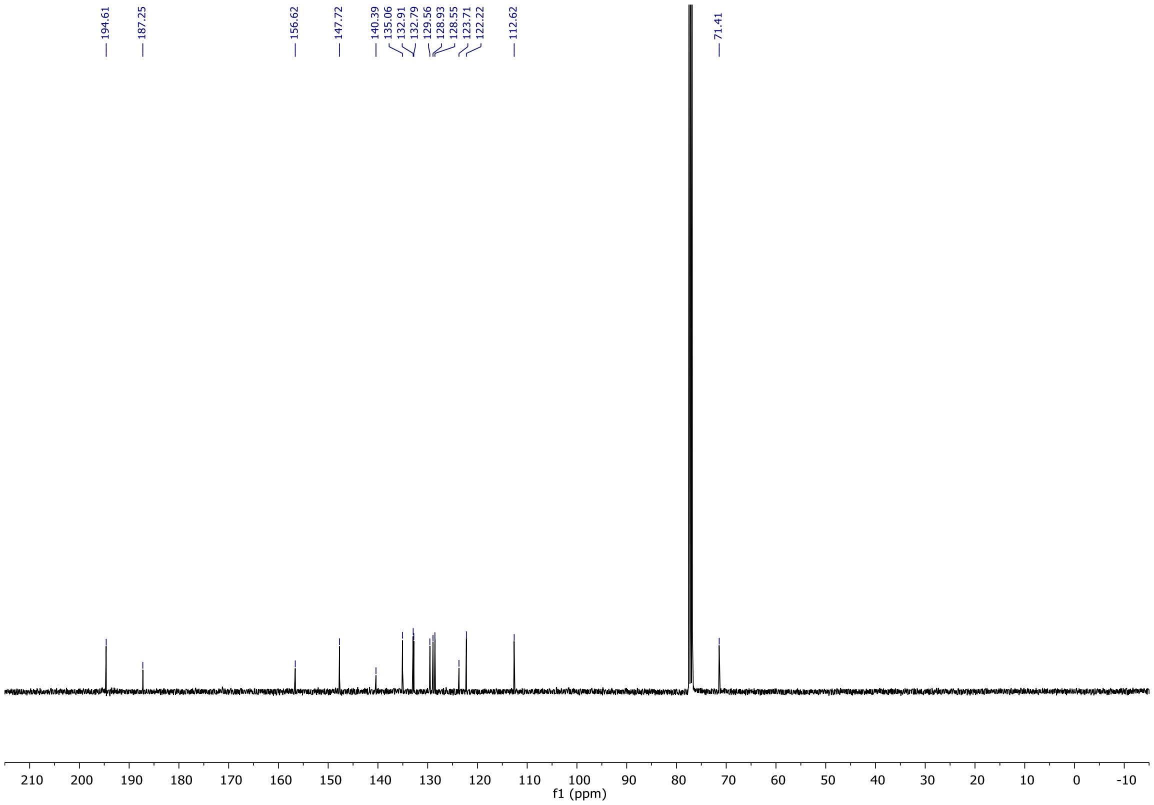
194.61
187.25
156.62
147.72
140.39
135.06
132.91
132.79
129.56
128.93
128.55
123.71
122.22
112.62
71.41
210 200 190 180 170 160 150 140 130 120 110 100 90 80 70 60 50 40 30 20 10 0 -10
f1 (ppm)

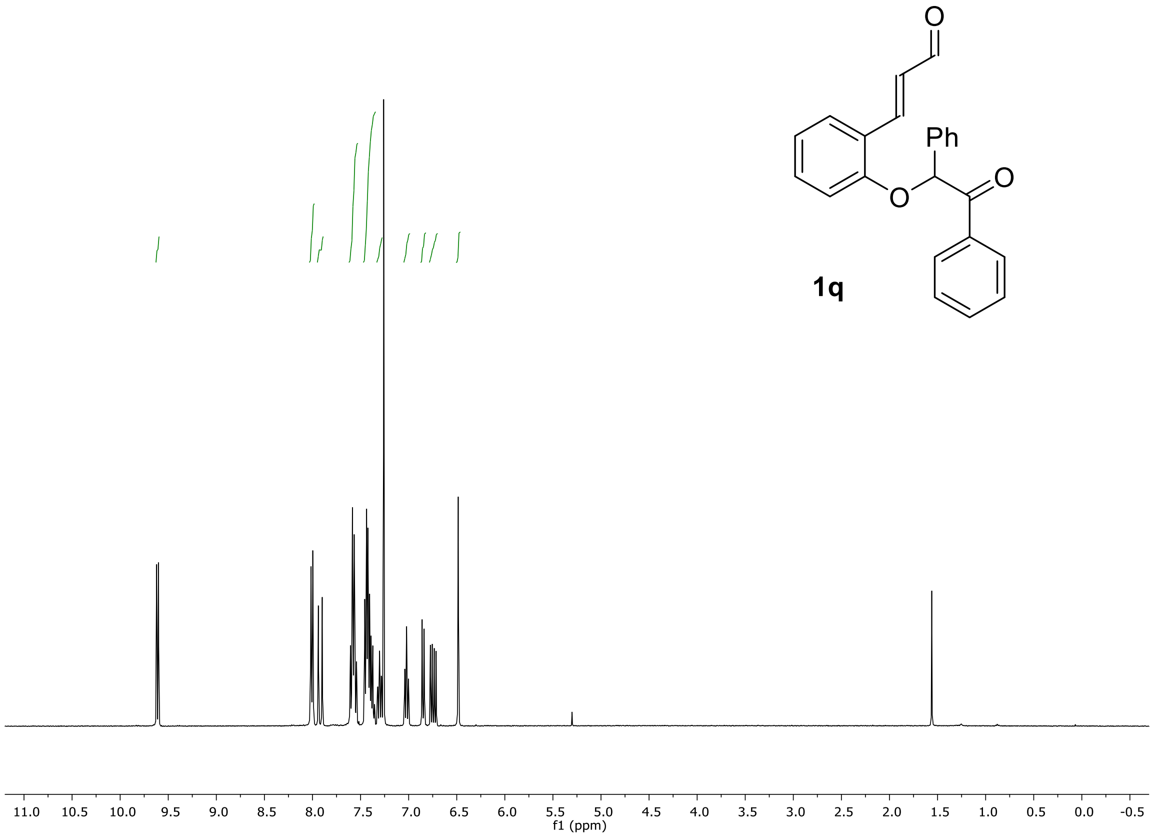

1q
f1 (ppm)

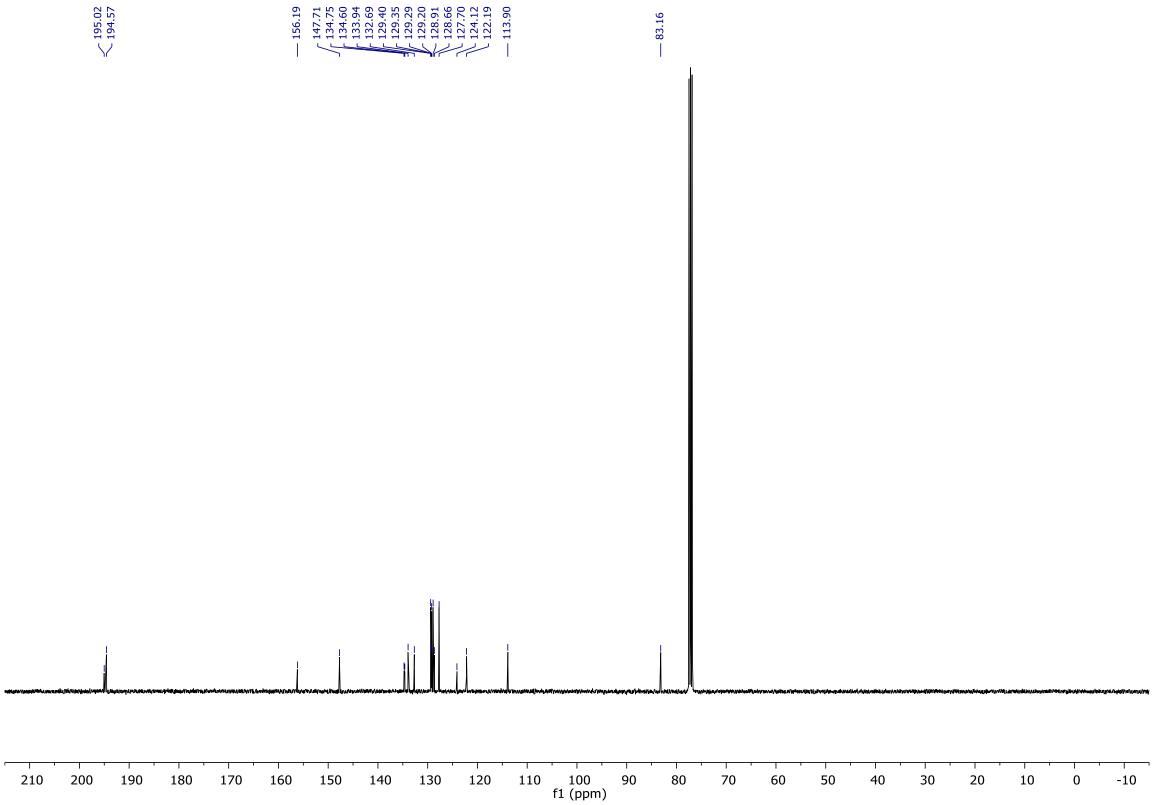
195.02
194.57
156.19
147.71
134.75
134.60
133.94
132.69
129.40
129.35
129.29
129.20
128.91
128.66
127.70
124.12
122.19
113.90
83.16
f1 (ppm)

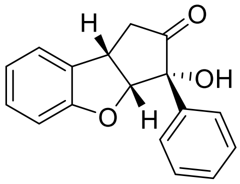
H
O
OH
O
H

3a

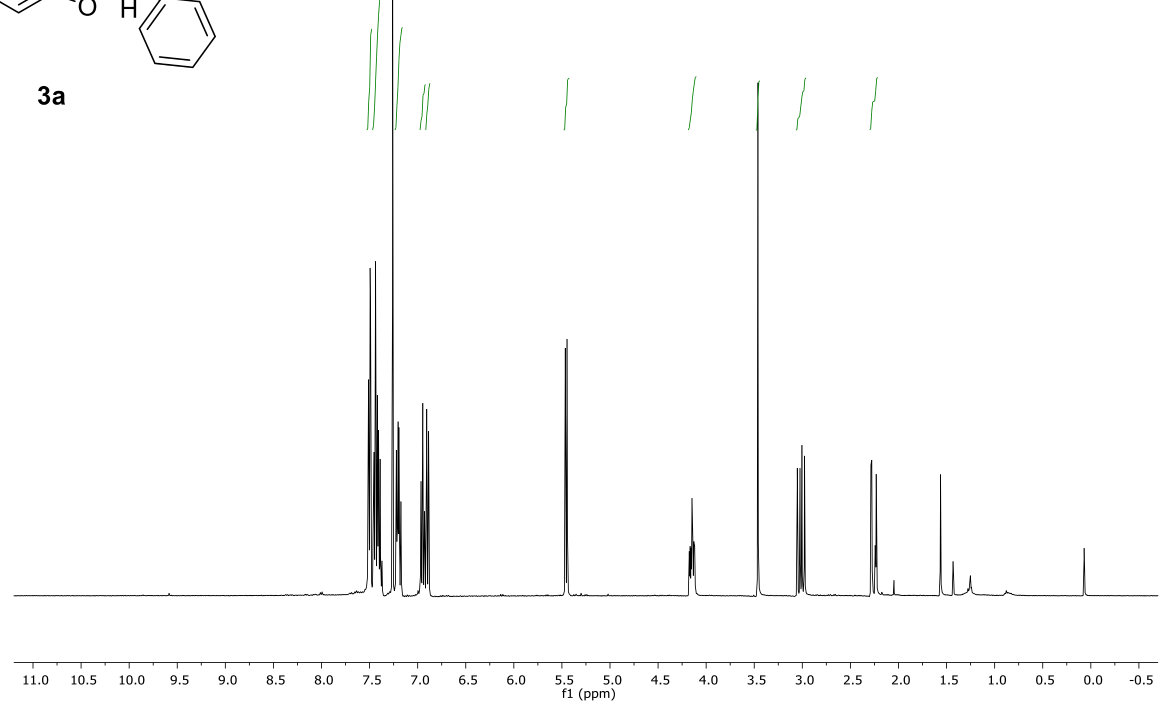
11.0 10.5 10.0 9.5 9.0 8.5 8.0 7.5 7.0 6.5 6.0 5.5 5.0 4.5 4.0 3.5 3.0 2.5 2.0 1.5 1.0 0.5 0.0 -0.5
f1 (ppm)

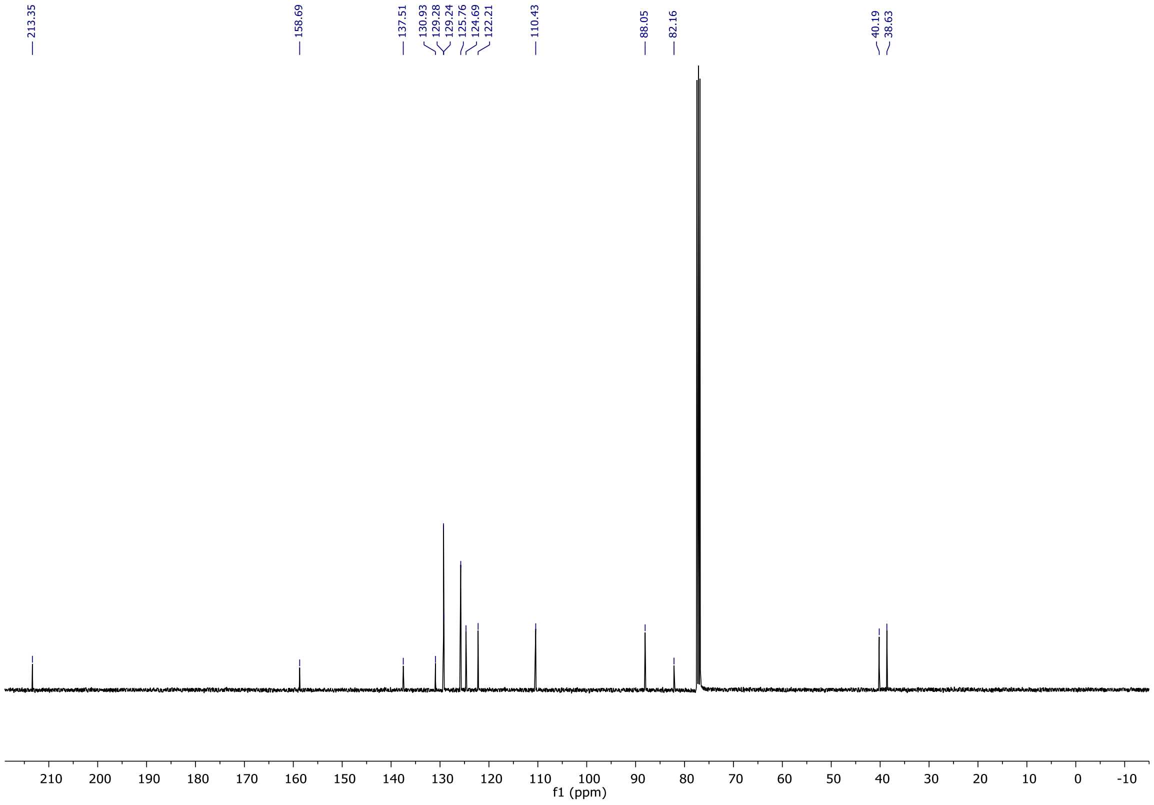
213.35
158.69
137.51
130.93
129.28
129.24
125.76
124.69
122.21
110.43
88.05
82.16
40.19
38.63
210 200 190 180 170 160 150 140 130 120 110 100 90 80 70 60 50 40 30 20 10 0 -10
f1 (ppm)

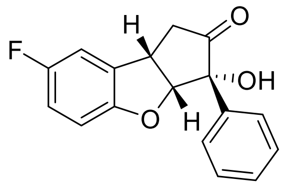
F
H
O
OH
O
H

3b

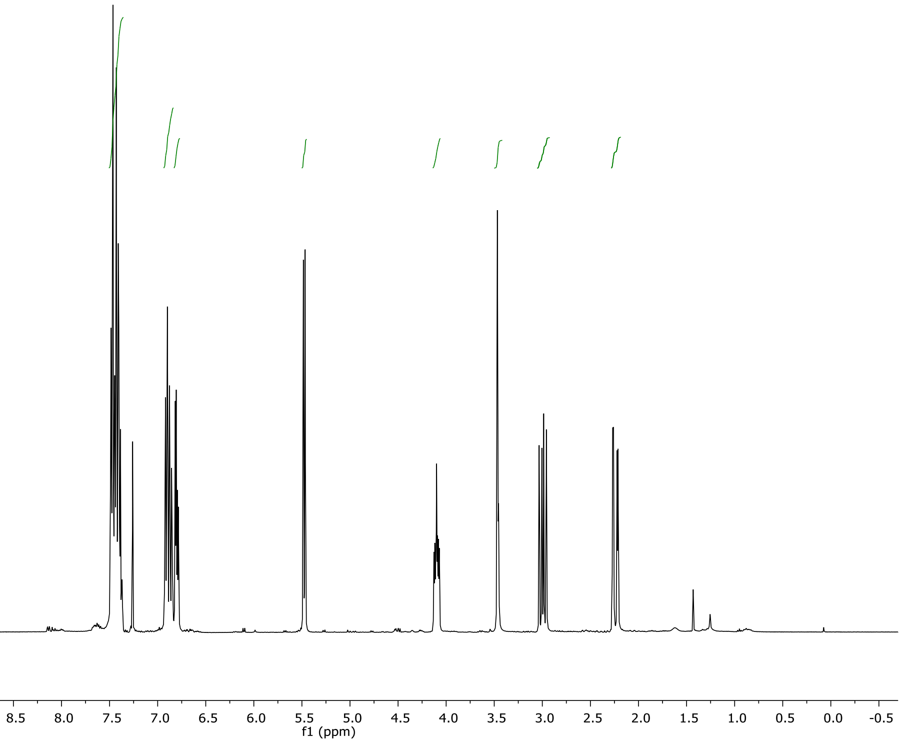
11.0 10.5 10.0 9.5 9.0 8.5 8.0 7.5 7.0 6.5 6.0 5.5 5.0 4.5 4.0 3.5 3.0 2.5 2.0 1.5 1.0 0.5 0.0 -0.5
f1 (ppm)

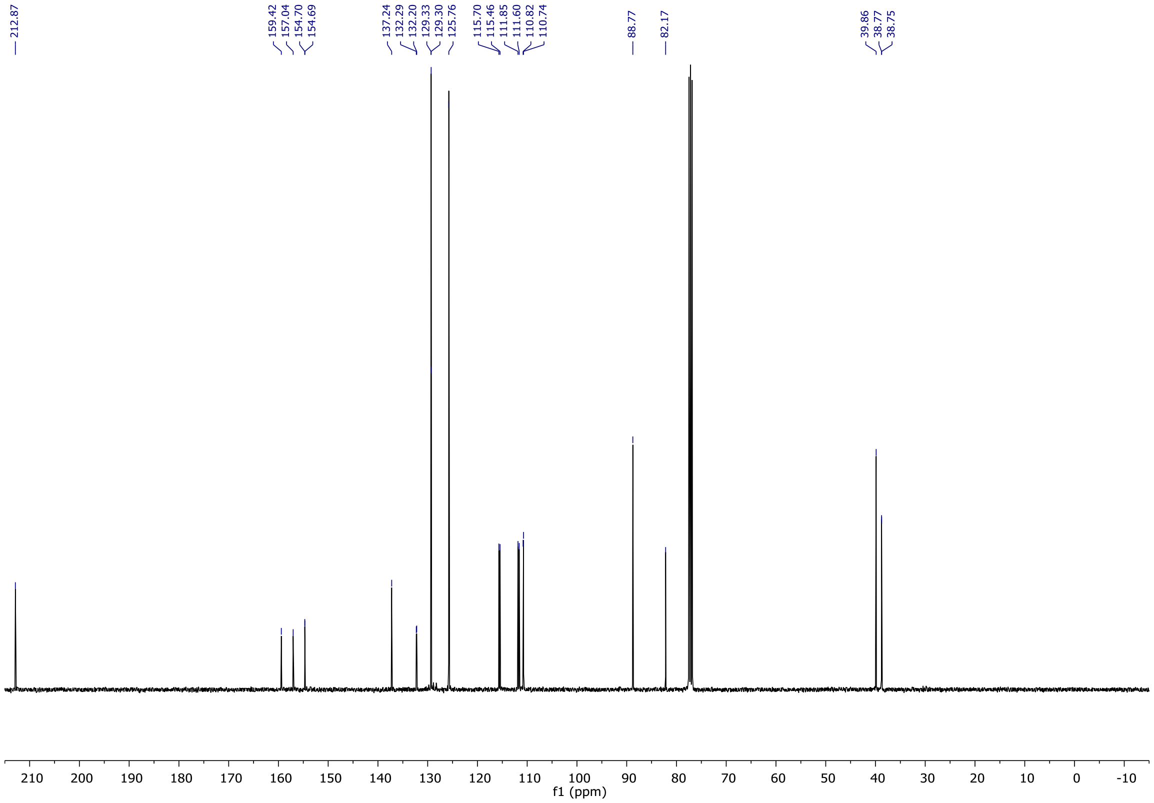
212.87
159.42
157.04
154.70
154.69
137.24
132.29
132.20
129.33
129.30
125.76
115.70
115.46
111.85
111.60
110.82
110.74
88.77
82.17
39.86
38.77
38.75
210 200 190 180 170 160 150 140 130 120 110 100 90 80 70 60 50 40 30 20 10 0 -10
f1 (ppm)

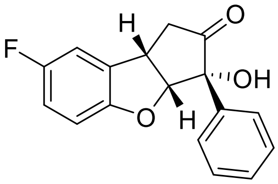

3b

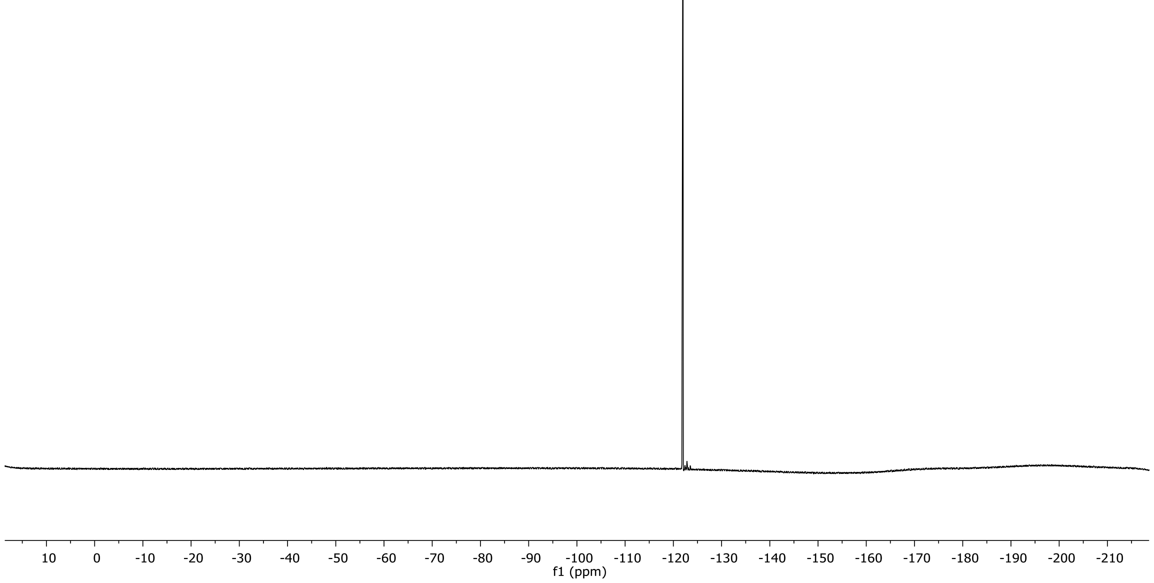

-121.98
10 0 -10 -20 -30 -40 -50 -60 -70 -80 -90 -100 -110 -120 -130 -140 -150 -160 -170 -180 -190 -200 -210
f1 (ppm)

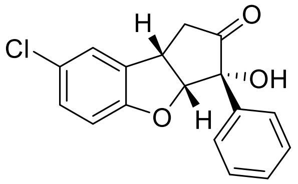
Cl
H
O
OH
O
H

3c

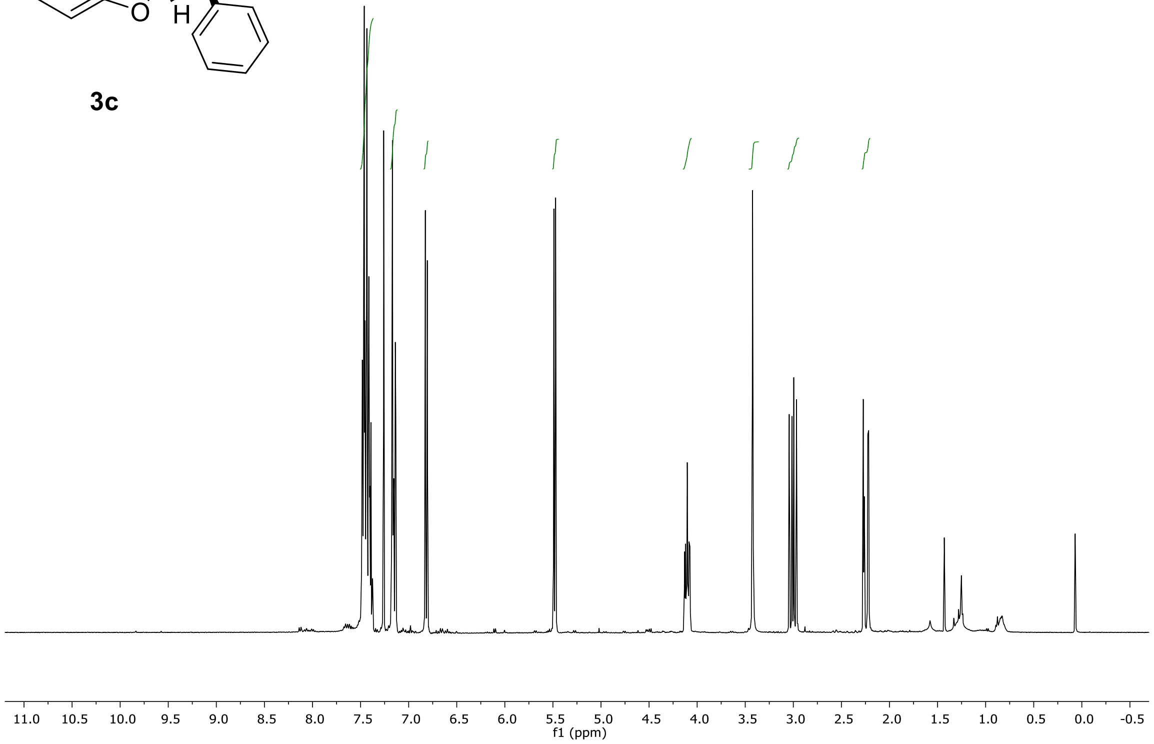
11.0 10.5 10.0 9.5 9.0 8.5 8.0 7.5 7.0 6.5 6.0 5.5 5.0 4.5 4.0 3.5 3.0 2.5 2.0 1.5 1.0 0.5 0.0 -0.5
f1 (ppm)

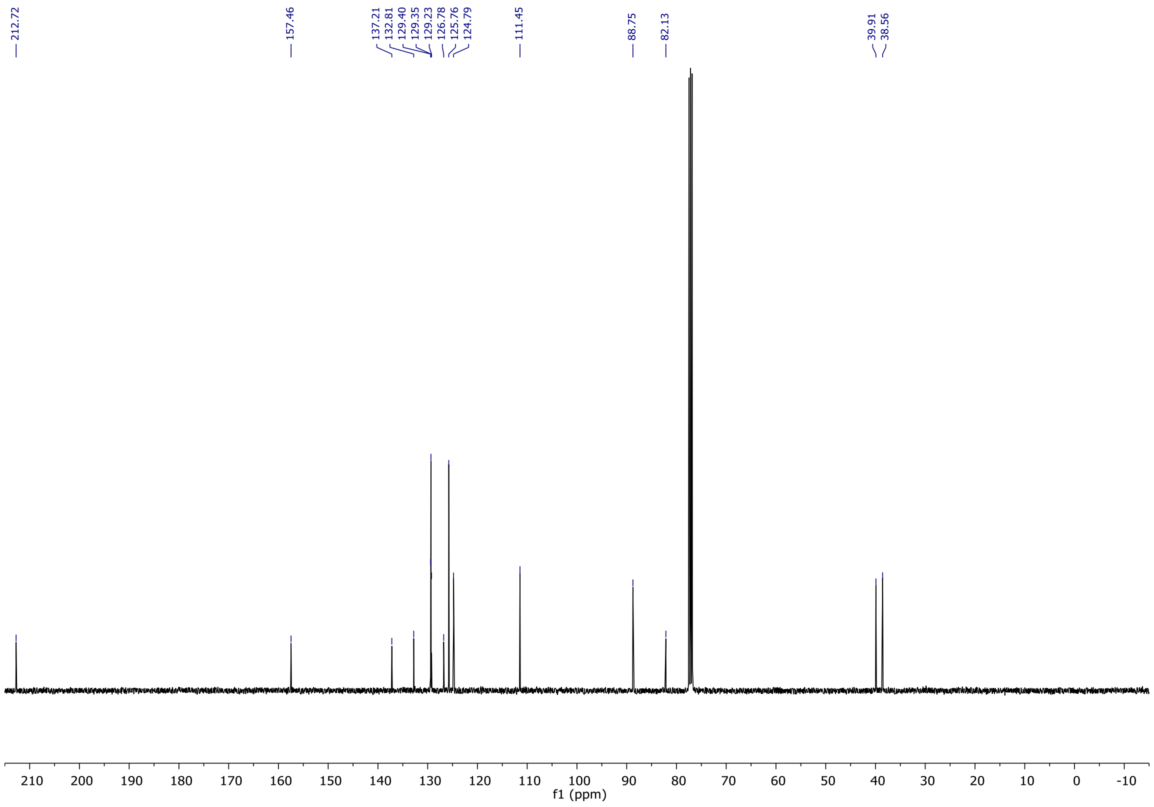
212.72
157.46
137.21
132.81
129.40
129.35
129.23
126.78
125.76
124.79
111.45
88.75
82.13
39.91
38.56
210 200 190 180 170 160 150 140 130 120 110 100 90 80 70 60 50 40 30 20 10 0 -10
f1 (ppm)

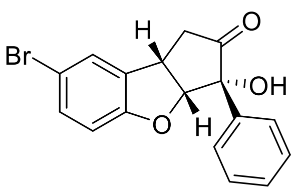
Br
H
O
OH
O
H

3d

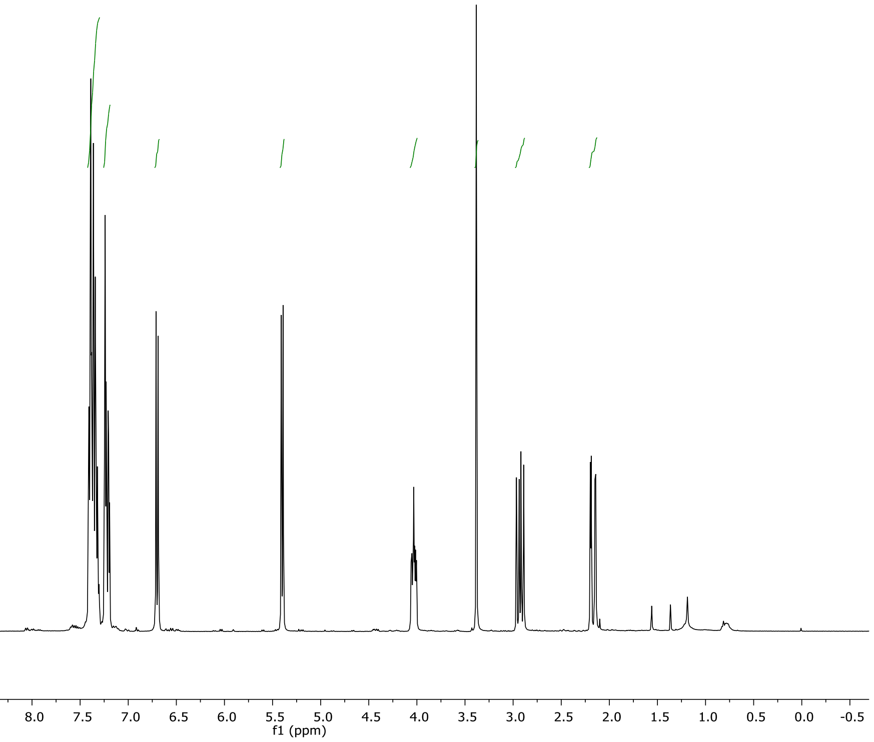
11.0 10.5 10.0 9.5 9.0 8.5 8.0 7.5 7.0 6.5 6.0 5.5 5.0 4.5 4.0 3.5 3.0 2.5 2.0 1.5 1.0 0.5 0.0 -0.5
f1 (ppm)

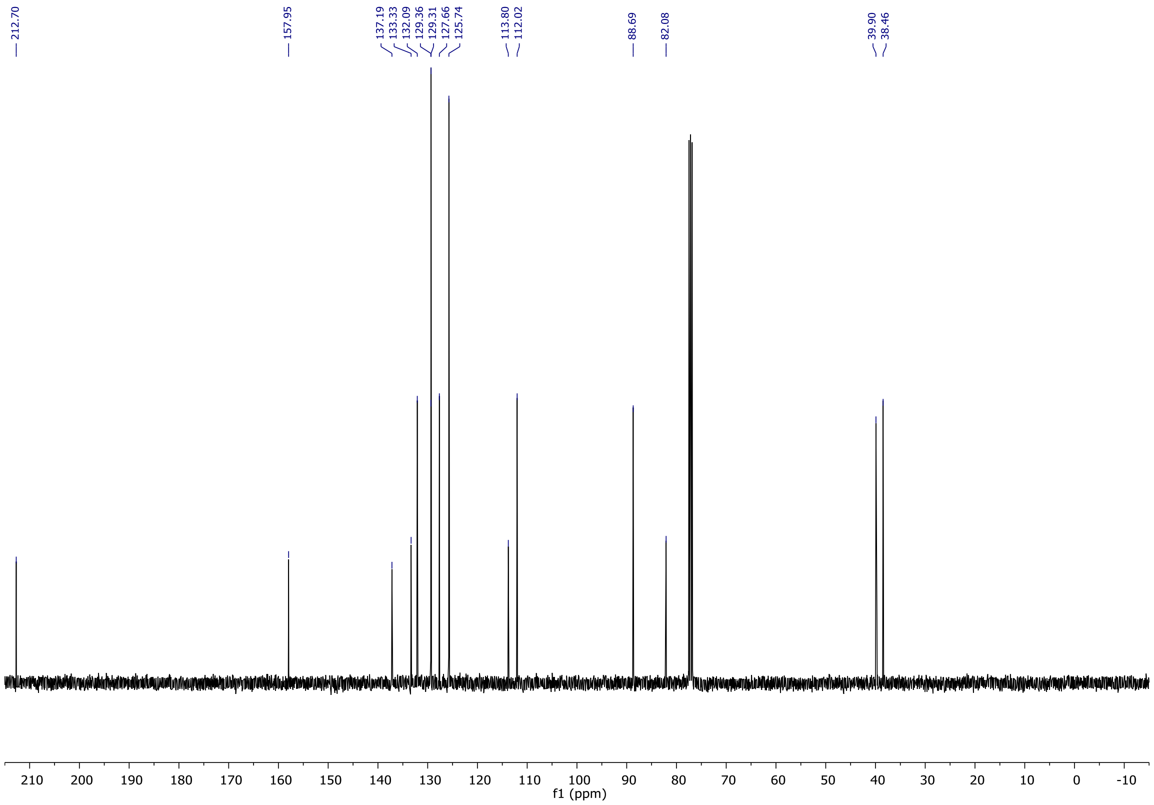
212.70
157.95
137.19
133.33
132.09
129.36
129.31
127.66
125.74
113.80
112.02
88.69
82.08
39.90
38.46
210 200 190 180 170 160 150 140 130 120 110 100 90 80 70 60 50 40 30 20 10 0 -10
f1 (ppm)

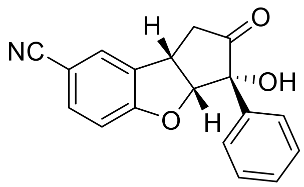
NC
H
O
OH
O
H

3e

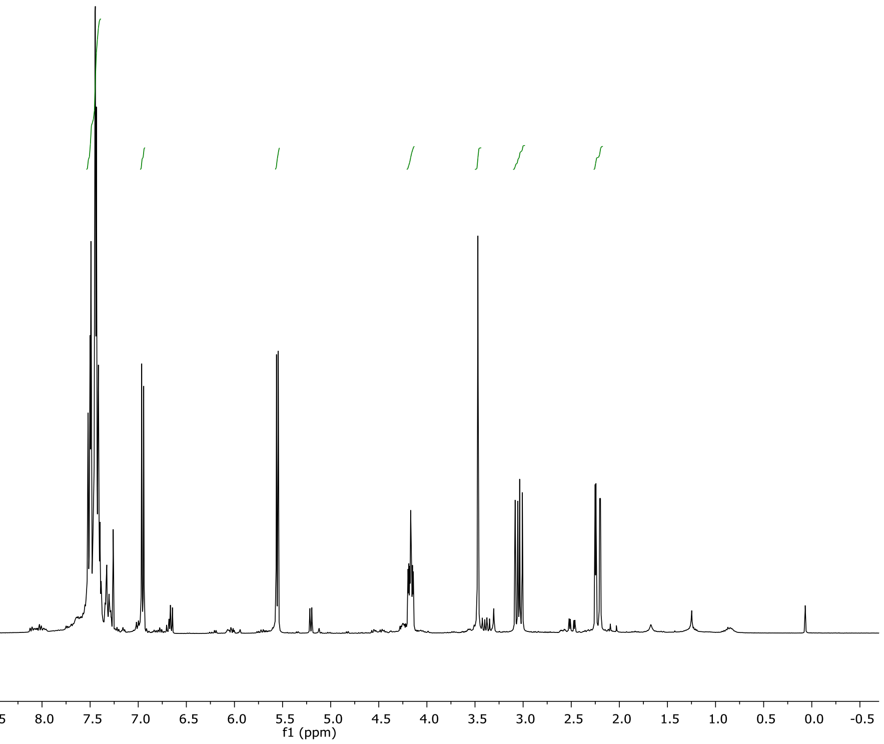
11.0 10.5 10.0 9.5 9.0 8.5 8.0 7.5 7.0 6.5 6.0 5.5 5.0 4.5 4.0 3.5 3.0 2.5 2.0 1.5 1.0 0.5 0.0 -0.5
f1 (ppm)

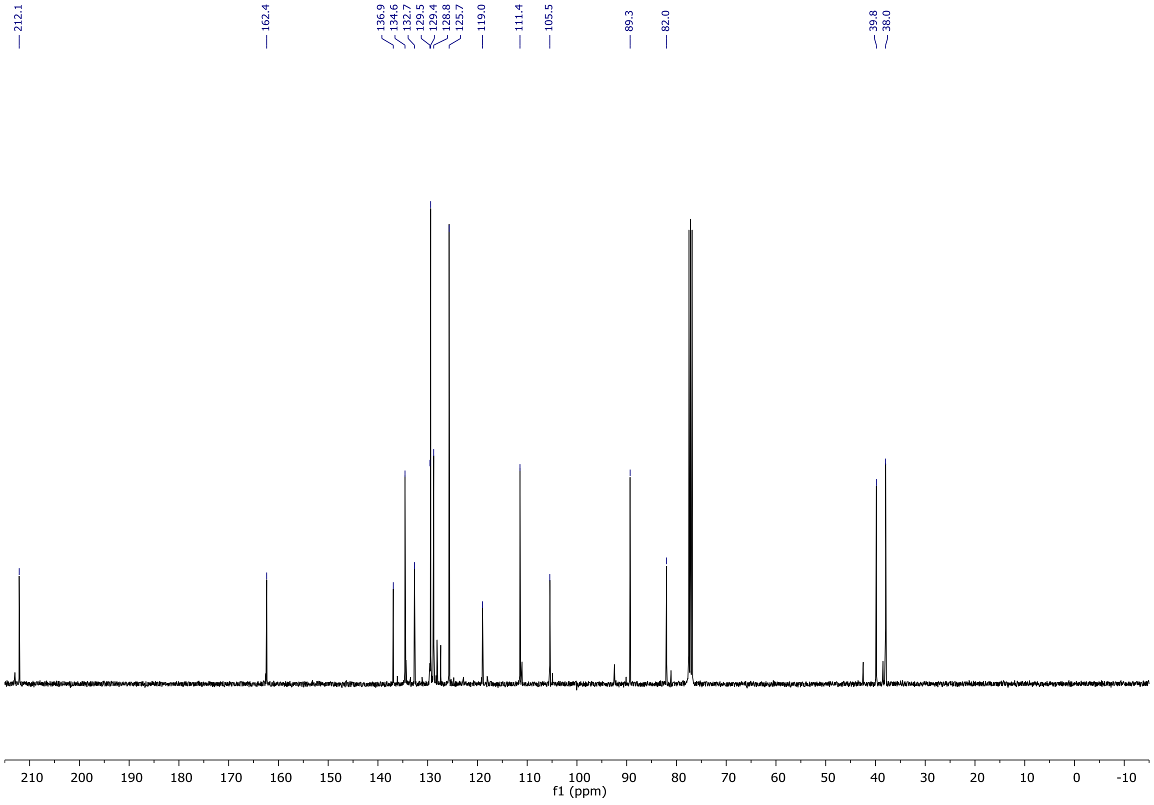
212.1
162.4
136.9
134.6
132.7
129.5
129.4
128.8
125.7
119.0
111.4
105.5
89.3
82.0
39.8
38.0
210 200 190 180 170 160 150 140 130 120 110 100 90 80 70 60 50 40 30 20 10 0 -10
f1 (ppm)

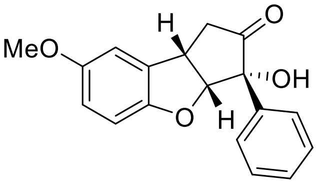
MeO
H
O
OH
O
H

3f

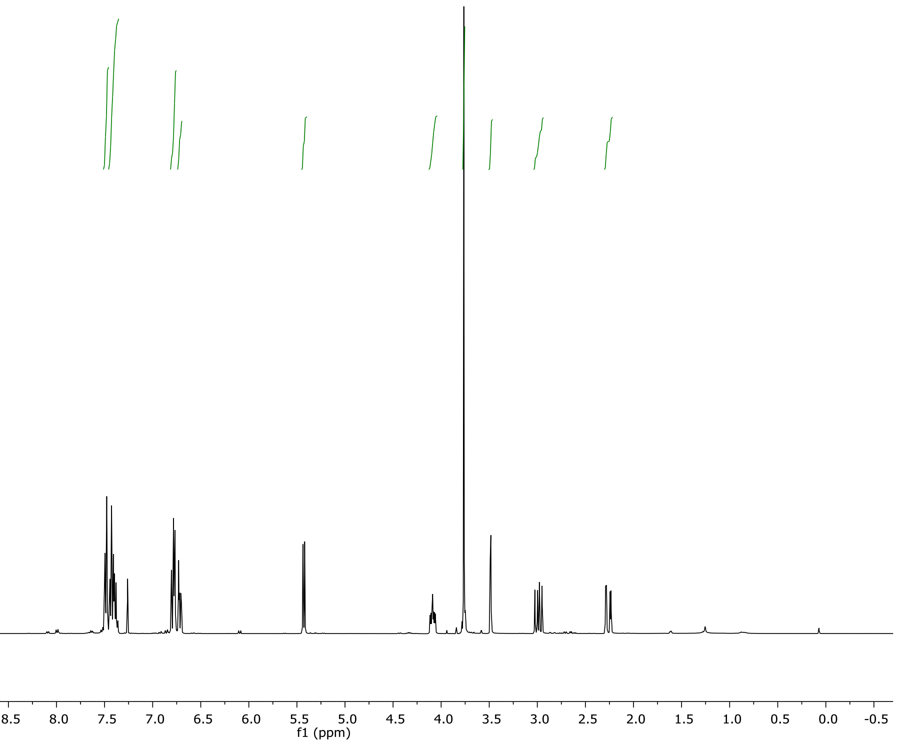
f1 (ppm)

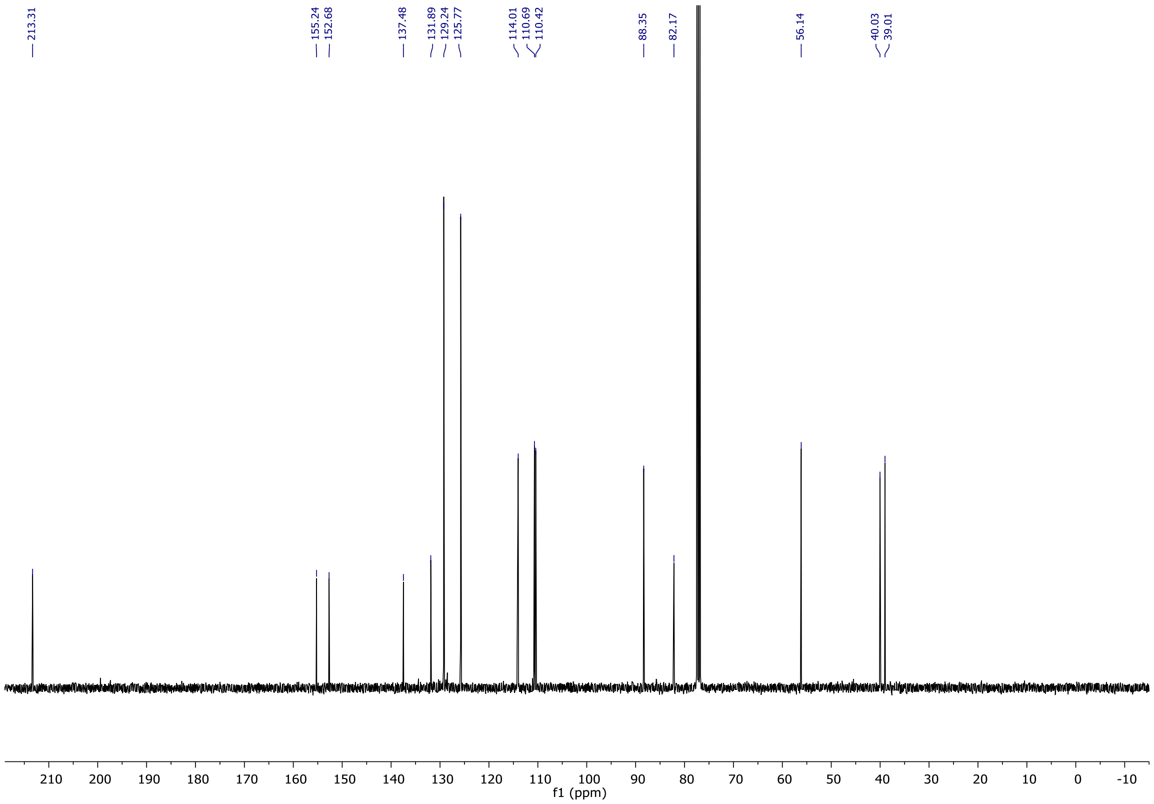
213.31
155.24
152.68
137.48
131.89
129.24
125.77
114.01
110.69
110.42
88.35
82.17
56.14
40.03
39.01
f1 (ppm)

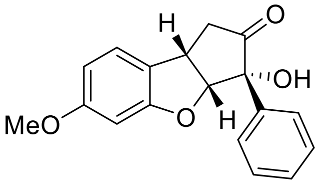
H
O
OH
MeO
O
H

3g

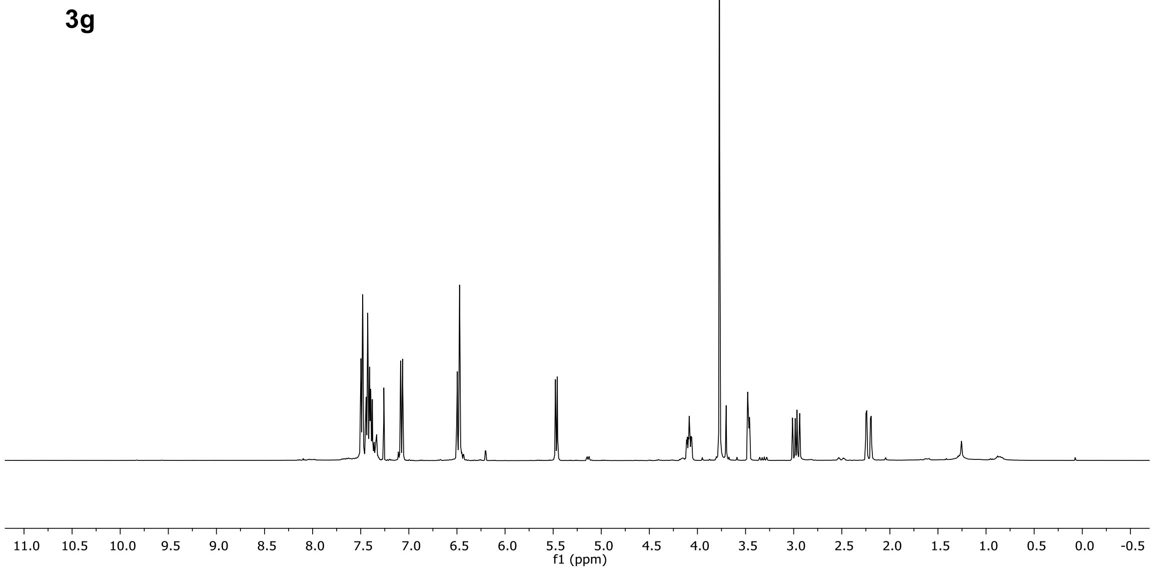
11.0 10.5 10.0 9.5 9.0 8.5 8.0 7.5 7.0 6.5 6.0 5.5 5.0 4.5 4.0 3.5 3.0 2.5 2.0 1.5 1.0 0.5 0.0 -0.5
f1 (ppm)

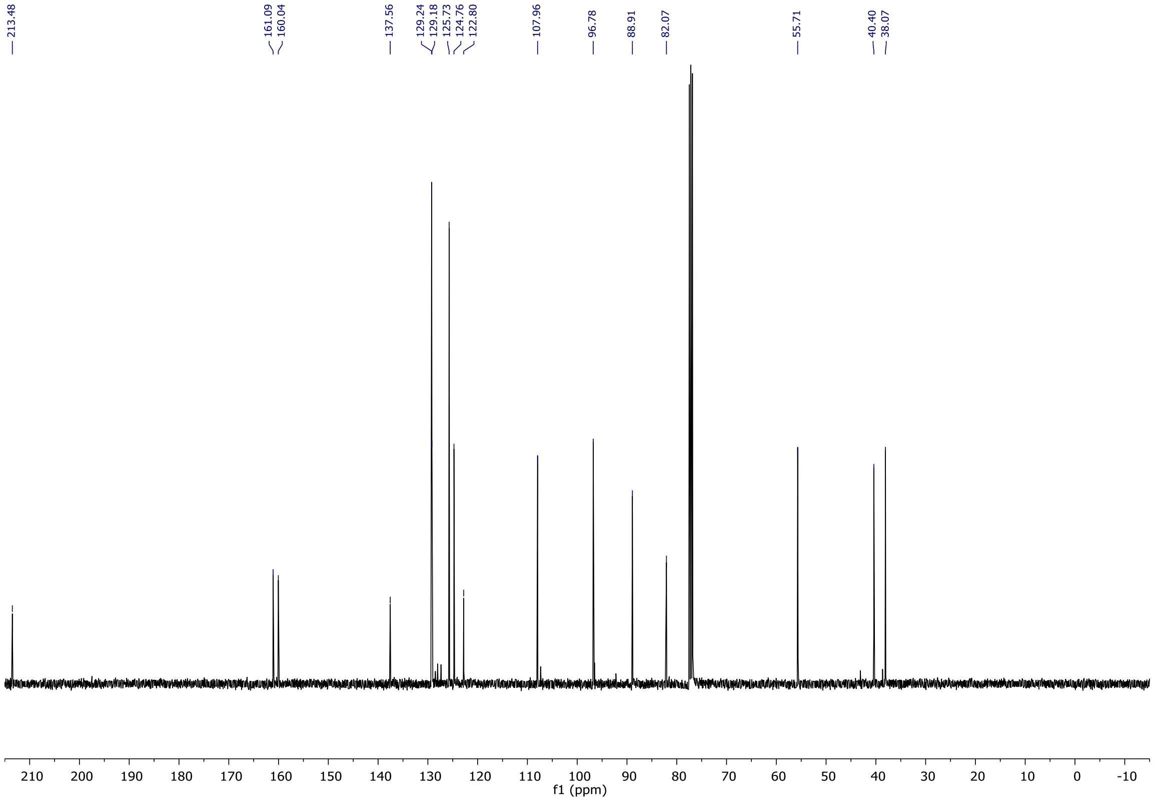
213.48
161.09
160.04
137.56
129.24
129.18
125.73
124.76
122.80
107.96
96.78
88.91
82.07
55.71
40.40
38.07
210 200 190 180 170 160 150 140 130 120 110 100 90 80 70 60 50 40 30 20 10 0 -10
f1 (ppm)

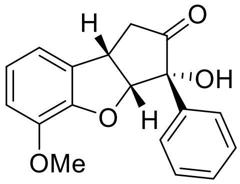
H
O
OH
O
H
OMe

3h

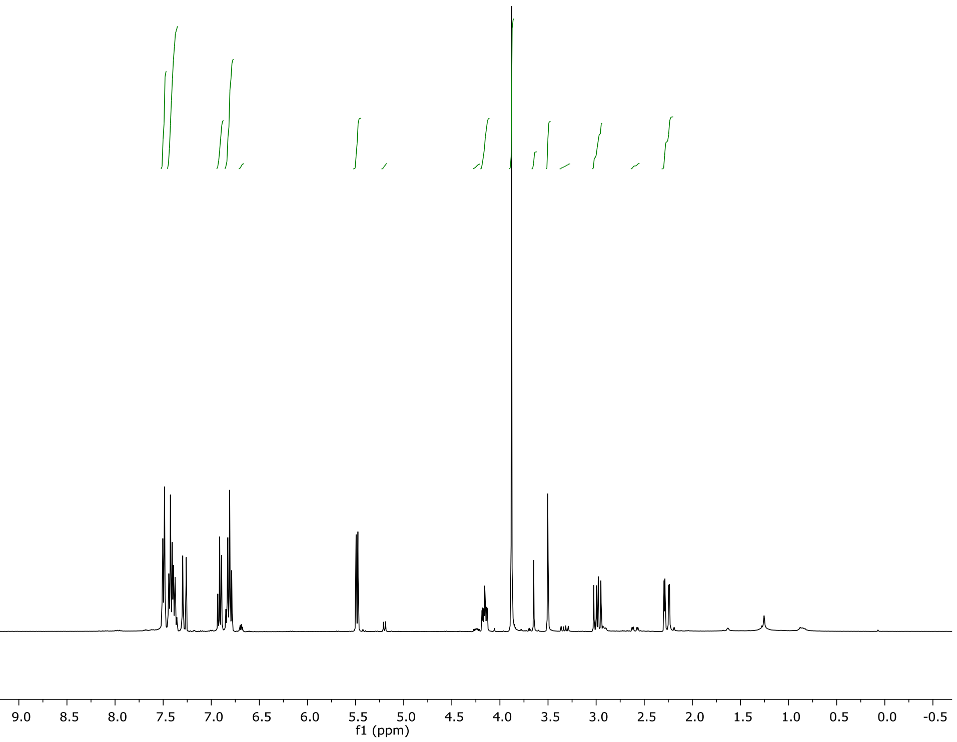
11.0 10.5 10.0 9.5 9.0 8.5 8.0 7.5 7.0 6.5 6.0 5.5 5.0 4.5 4.0 3.5 3.0 2.5 2.0 1.5 1.0 0.5 0.0 -0.5
f1 (ppm)

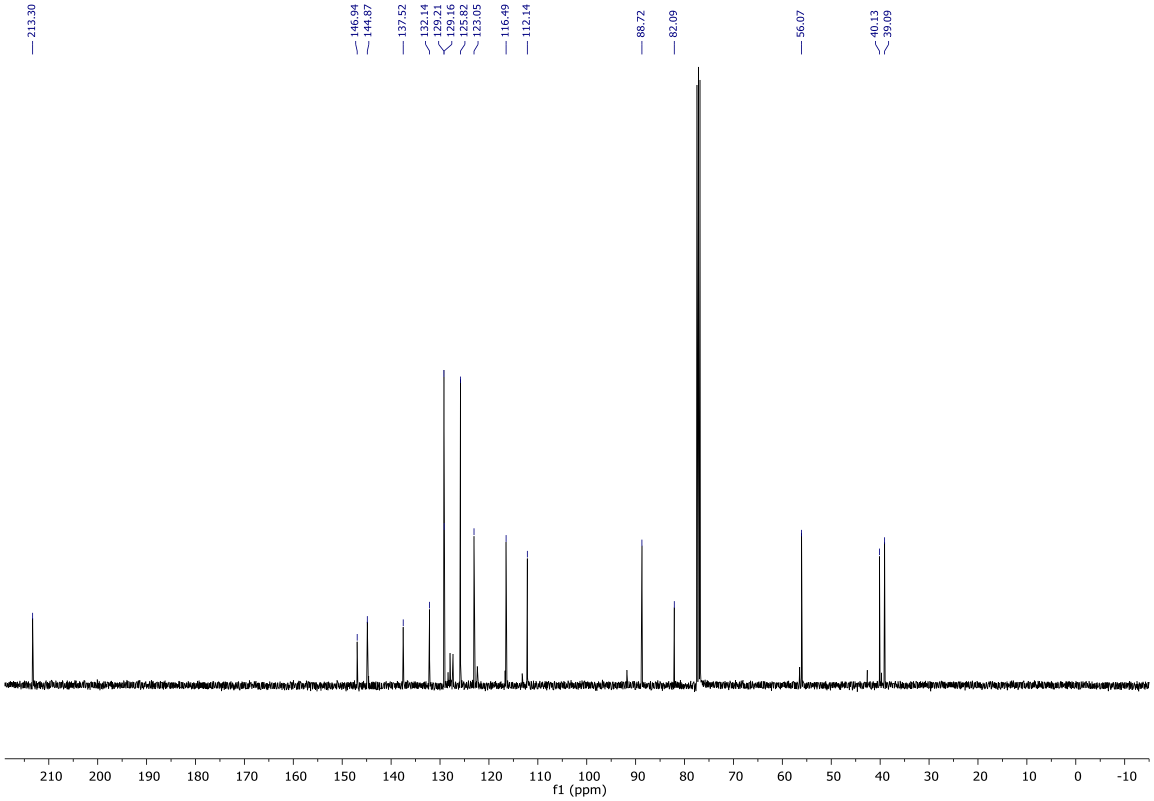
213.30
146.94
144.87
137.52
132.14
129.21
129.16
125.82
123.05
116.49
112.14
88.72
82.09
56.07
40.13
39.09
210 200 190 180 170 160 150 140 130 120 110 100 90 80 70 60 50 40 30 20 10 0 -10
f1 (ppm)

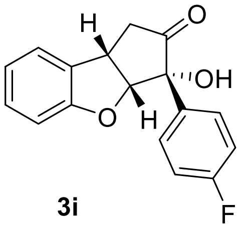
H
O
OH
O
H
F

3i

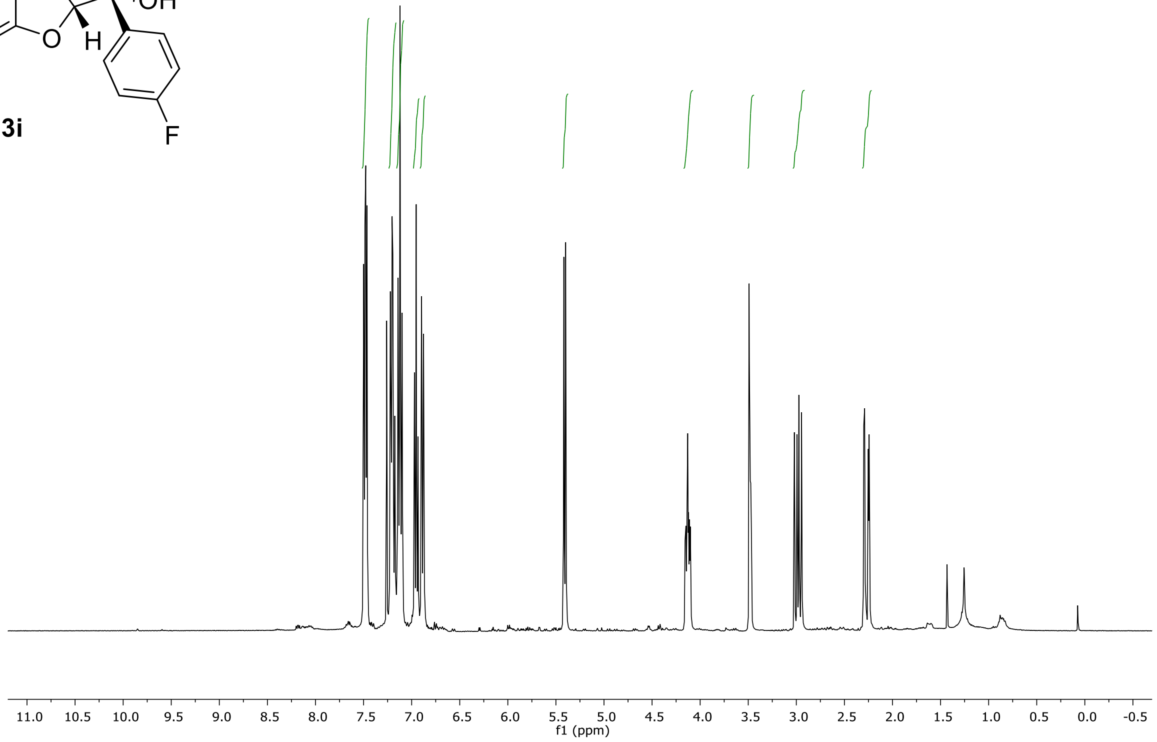
11.0 10.5 10.0 9.5 9.0 8.5 8.0 7.5 7.0 6.5 6.0 5.5 5.0 4.5 4.0 3.5 3.0 2.5 2.0 1.5 1.0 0.5 0.0 -0.5
f1 (ppm)

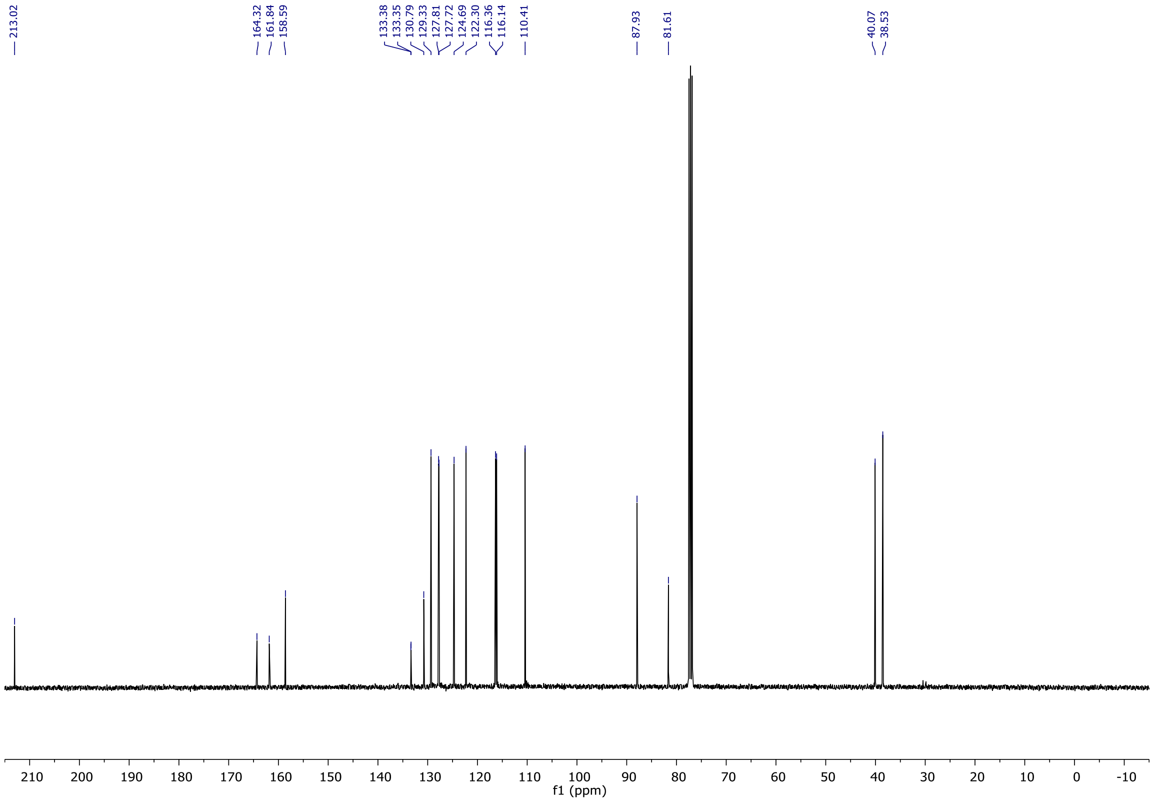
213.02
164.32
161.84
158.59
133.38
133.35
130.79
129.33
127.81
127.72
124.69
122.30
116.36
116.14
110.41
87.93
81.61
40.07
38.53
210 200 190 180 170 160 150 140 130 120 110 100 90 80 70 60 50 40 30 20 10 0 -10
f1 (ppm)

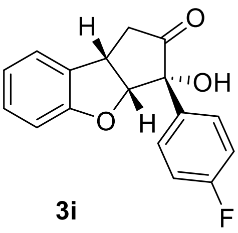
H
O
OH
O
H
F

3i

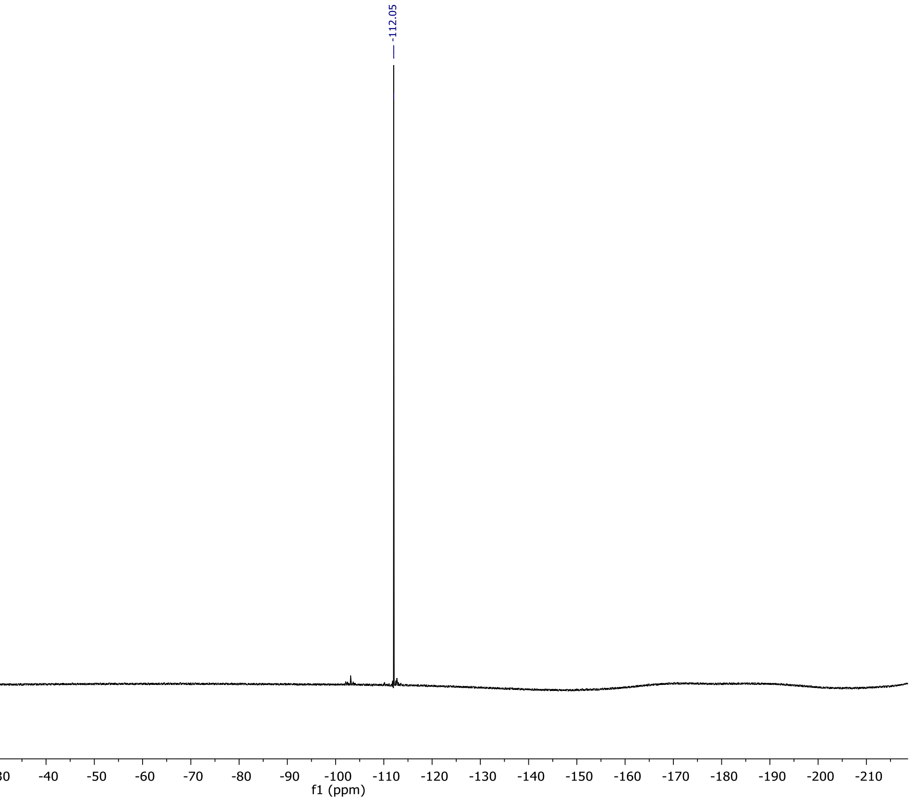
-112.05
10 0 -10 -20 -30 -40 -50 -60 -70 -80 -90 -100 -110 -120 -130 -140 -150 -160 -170 -180 -190 -200 -210
f1 (ppm)

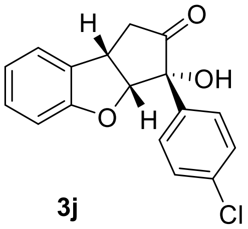
H
O
OH
O
H
Cl

3j

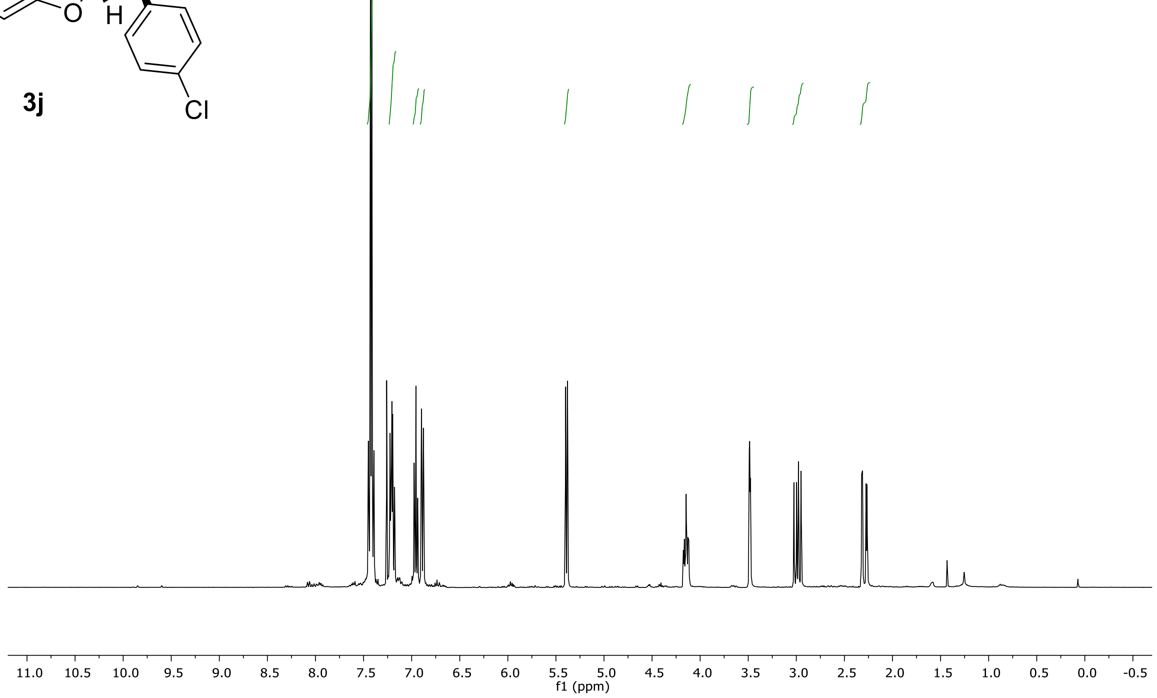
11.0 10.5 10.0 9.5 9.0 8.5 8.0 7.5 7.0 6.5 6.0 5.5 5.0 4.5 4.0 3.5 3.0 2.5 2.0 1.5 1.0 0.5 0.0 -0.5
f1 (ppm)

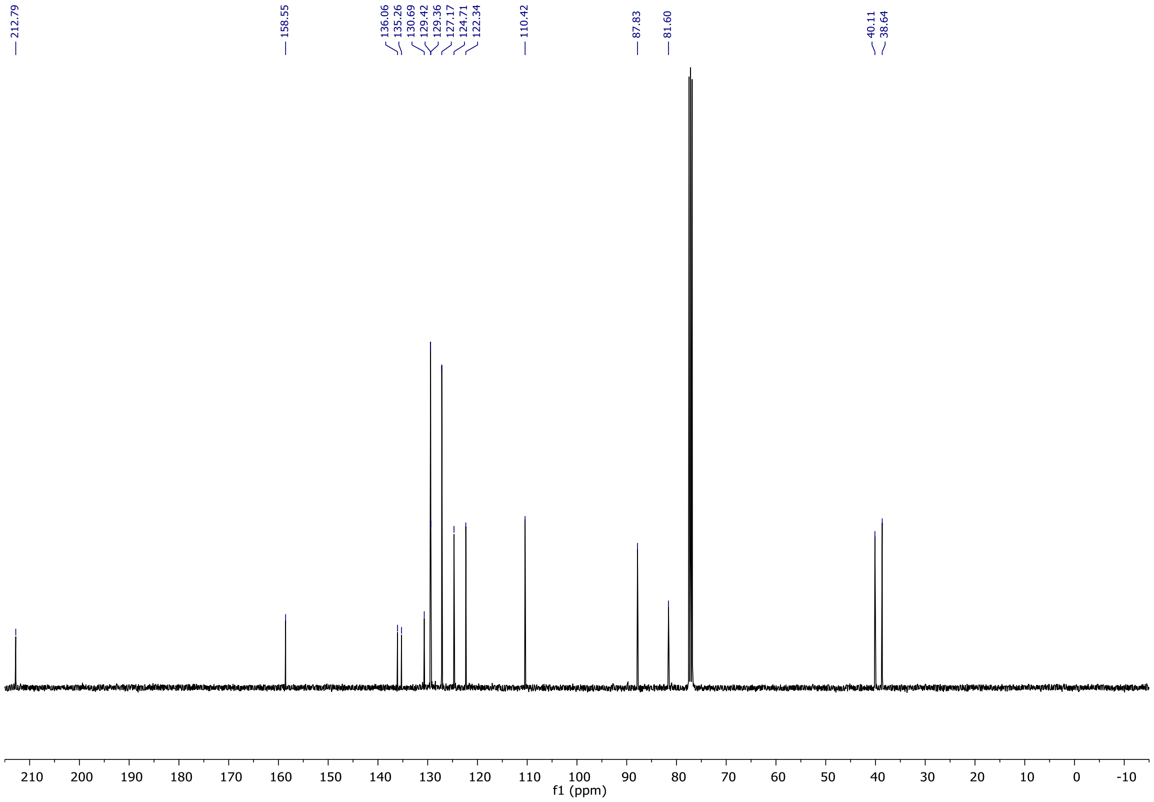
212.79
158.55
136.06
135.26
130.69
129.42
129.36
127.17
124.71
122.34
110.42
87.83
81.60
40.11
38.64
210 200 190 180 170 160 150 140 130 120 110 100 90 80 70 60 50 40 30 20 10 0 -10
f1 (ppm)

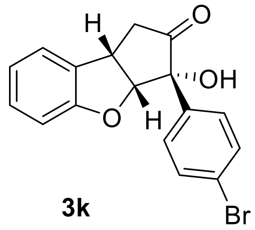
H
O
OH
O
H
Br

3k

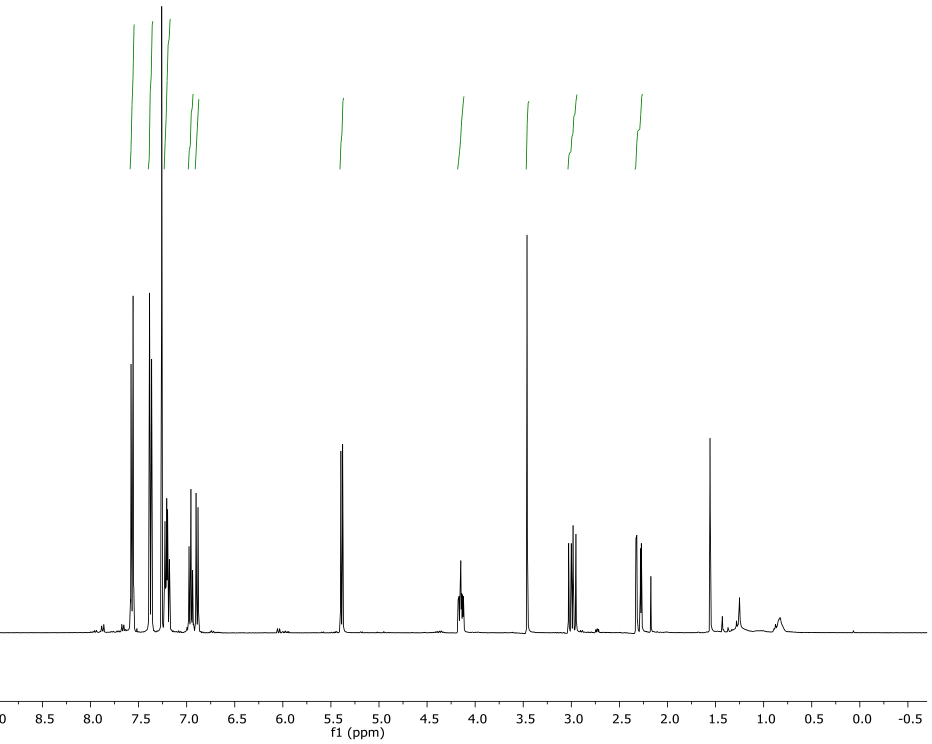
11.0 10.5 10.0 9.5 9.0 8.5 8.0 7.5 7.0 6.5 6.0 5.5 5.0 4.5 4.0 3.5 3.0 2.5 2.0 1.5 1.0 0.5 0.0 -0.5
f1 (ppm)

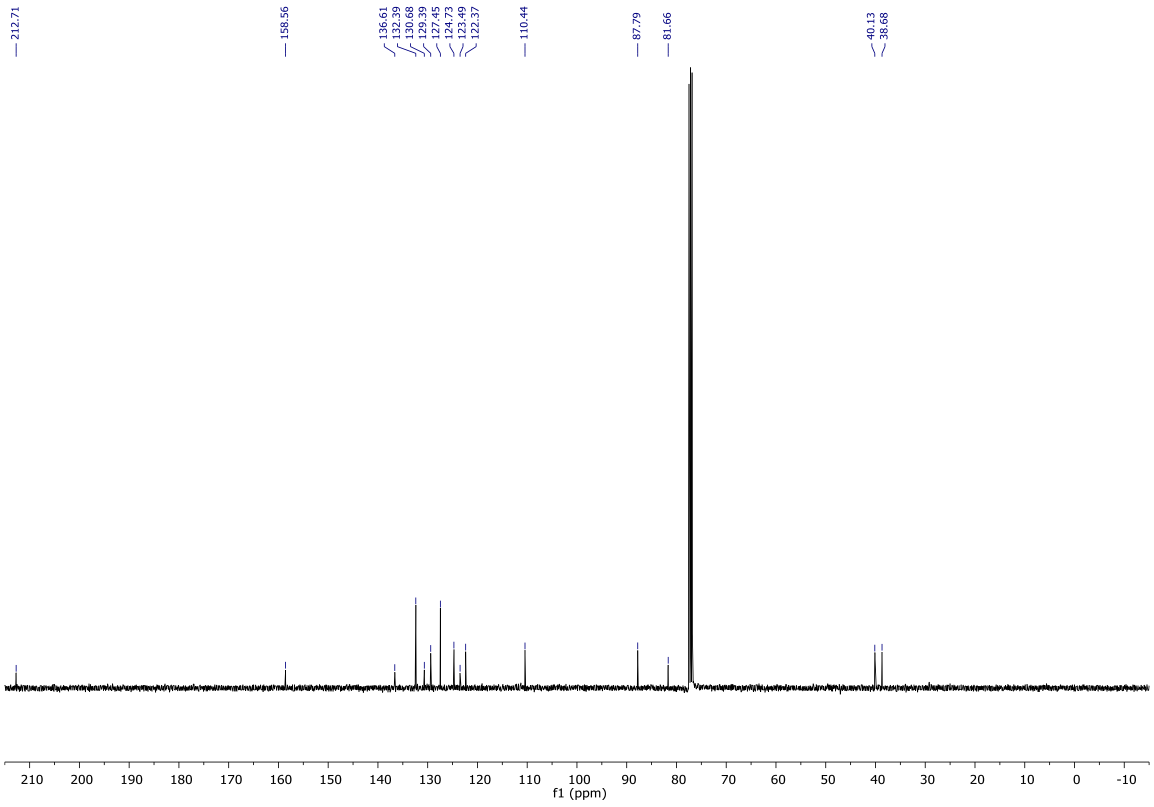
212.71
158.56
136.61
132.39
130.68
129.39
127.45
124.73
123.49
122.37
110.44
87.79
81.66
40.13
38.68
210 200 190 180 170 160 150 140 130 120 110 100 90 80 70 60 50 40 30 20 10 0 -10
f1 (ppm)

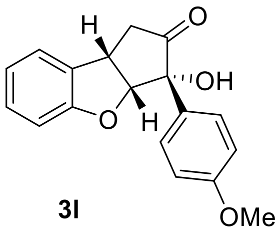
H
O
OH
O
H
OMe

3l

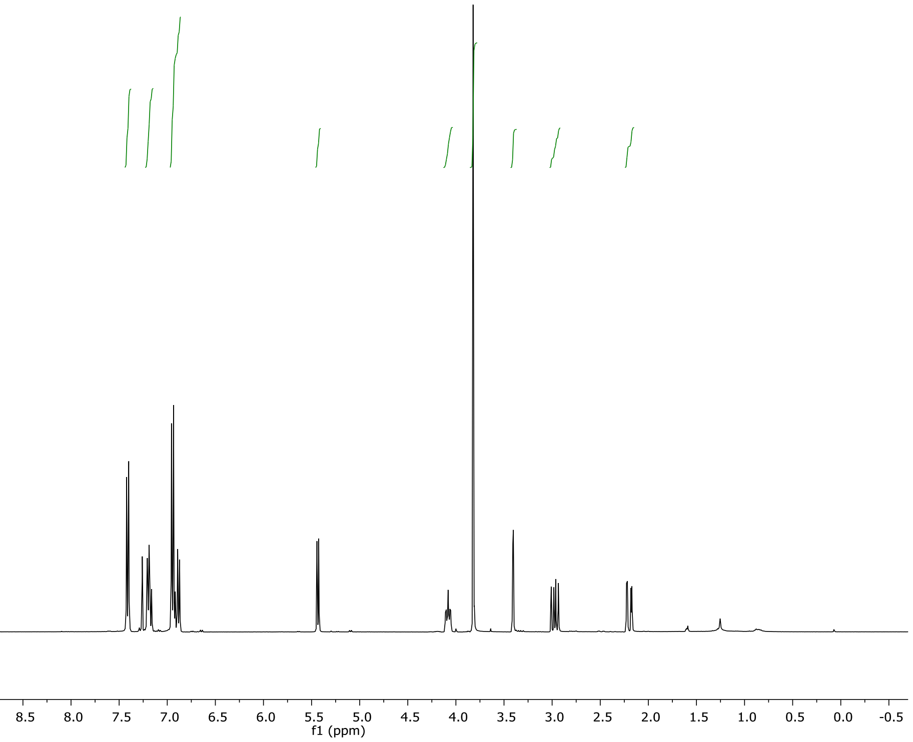
11.0 10.5 10.0 9.5 9.0 8.5 8.0 7.5 7.0 6.5 6.0 5.5 5.0 4.5 4.0 3.5 3.0 2.5 2.0 1.5 1.0 0.5 0.0 -0.5
f1 (ppm)

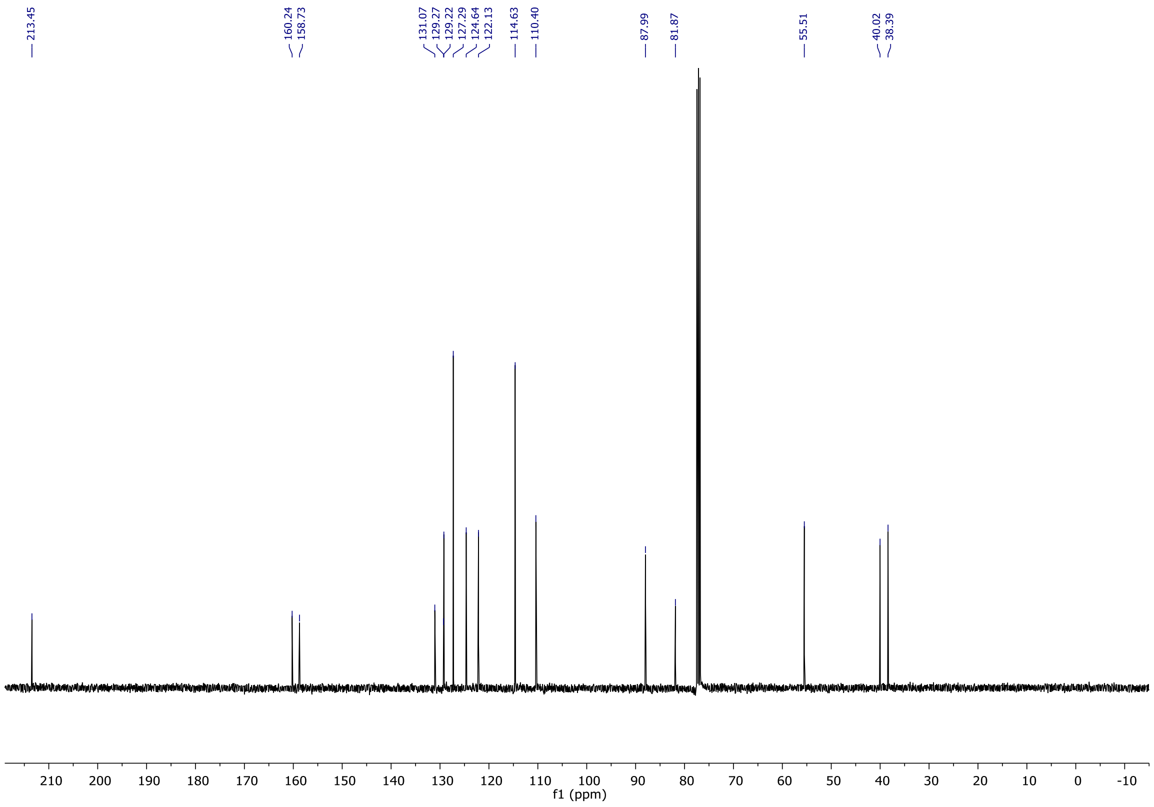
213.45
160.24
158.73
131.07
129.27
129.22
127.29
124.64
122.13
114.63
110.40
87.99
81.87
55.51
40.02
38.39
210 200 190 180 170 160 150 140 130 120 110 100 90 80 70 60 50 40 30 20 10 0 -10
f1 (ppm)

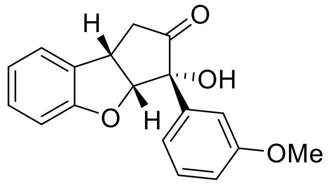
H
O
OH
O
H
OMe

3m

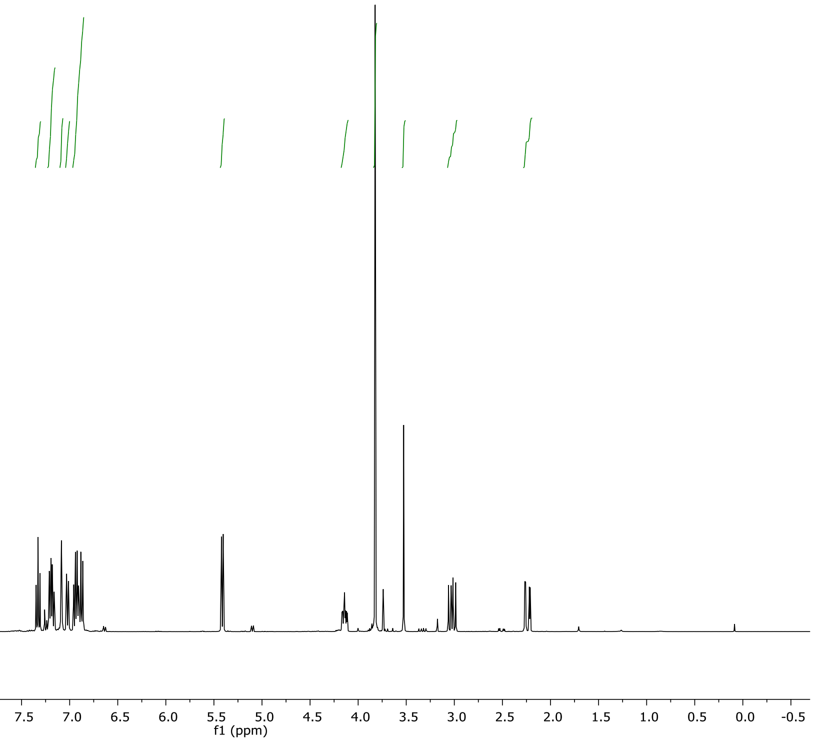
11.0 10.5 10.0 9.5 9.0 8.5 8.0 7.5 7.0 6.5 6.0 5.5 5.0 4.5 4.0 3.5 3.0 2.5 2.0 1.5 1.0 0.5 0.0 -0.5
f1 (ppm)

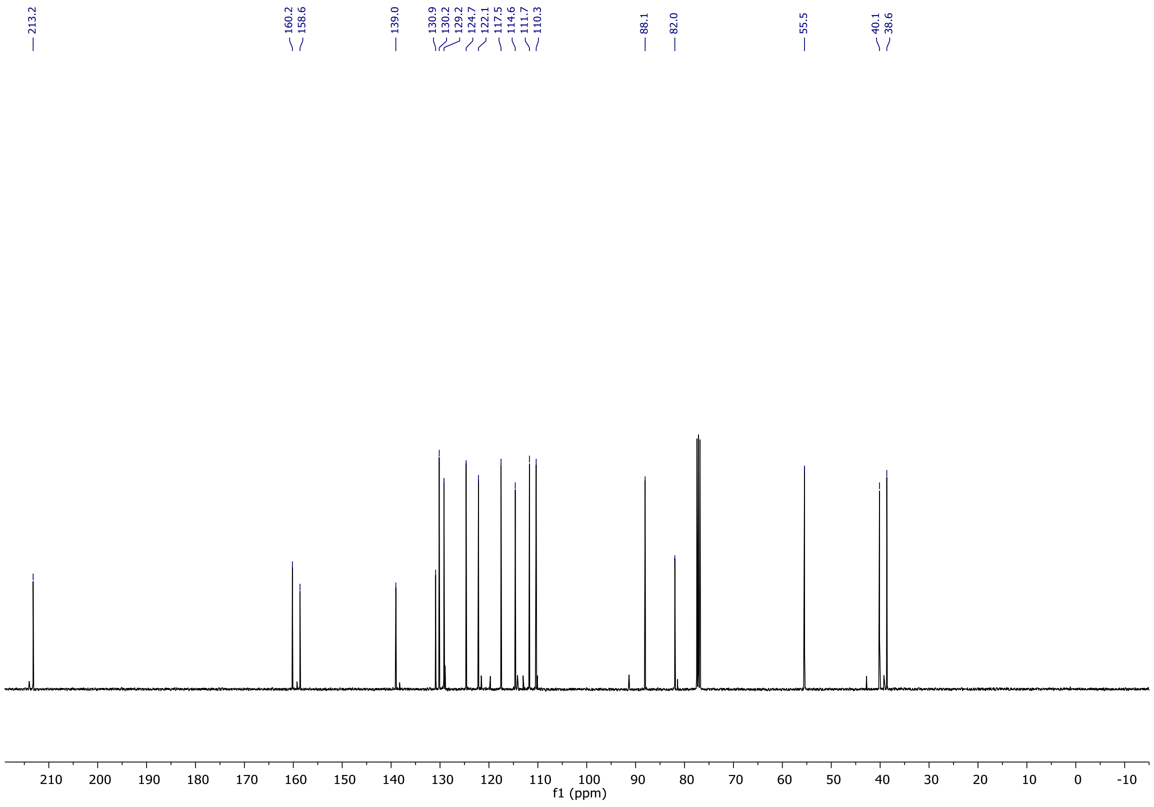
213.2
160.2
158.6
139.0
130.9
130.2
129.2
124.7
122.1
117.5
114.6
111.7
110.3
88.1
82.0
55.5
40.1
38.6
210 200 190 180 170 160 150 140 130 120 110 100 90 80 70 60 50 40 30 20 10 0 -10
f1 (ppm)

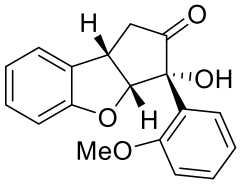

3n

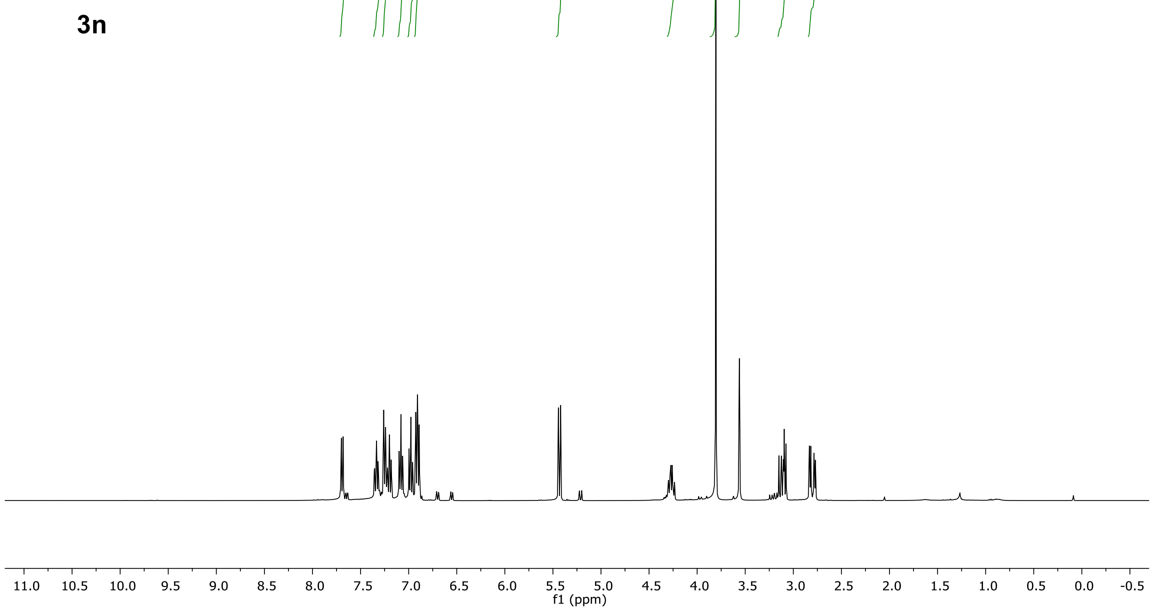

11.0 10.5 10.0 9.5 9.0 8.5 8.0 7.5 7.0 6.5 6.0 5.5 5.0 4.5 4.0 3.5 3.0 2.5 2.0 1.5 1.0 0.5 0.0 -0.5
f1 (ppm)

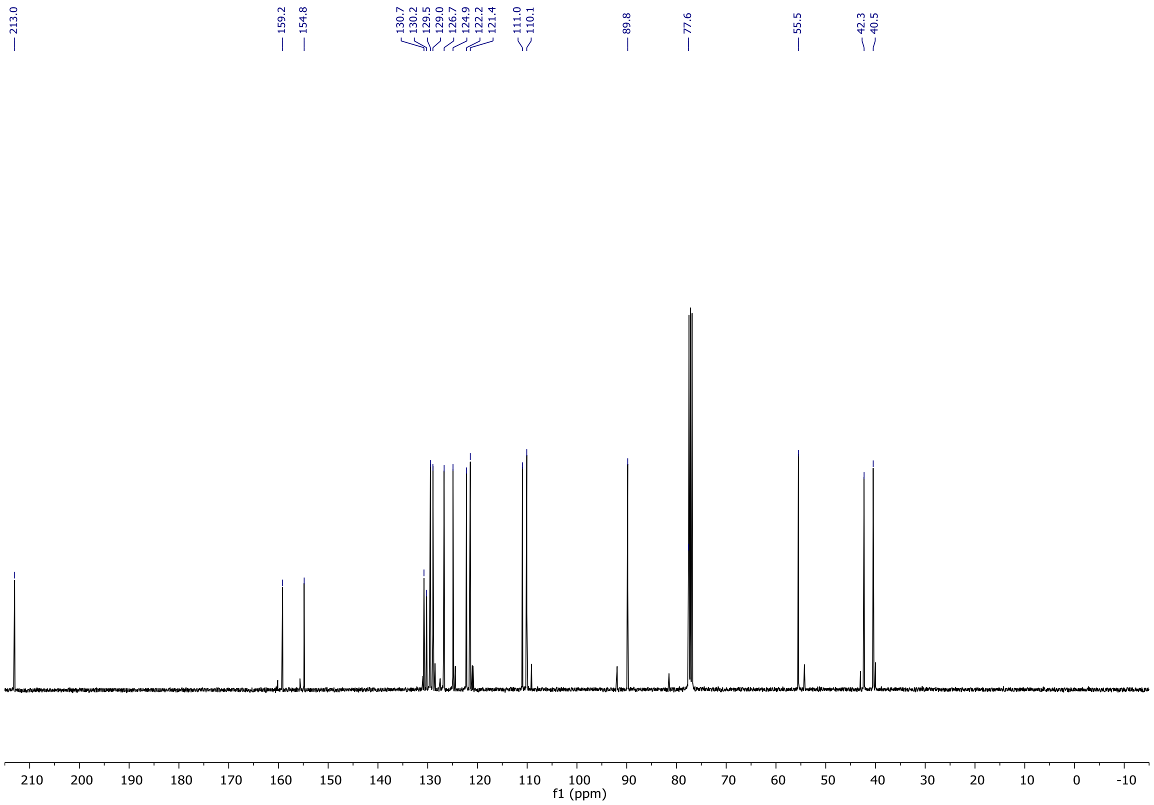

213.0
159.2
154.8
130.7
130.2
129.5
129.0
126.7
124.9
122.2
121.4
111.0
110.1
89.8
77.6
55.5
42.3
40.5
210 200 190 180 170 160 150 140 130 120 110 100 90 80 70 60 50 40 30 20 10 0 -10
f1 (ppm)

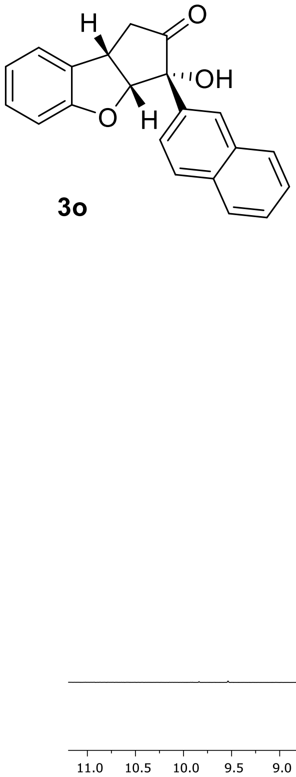
H
O
OH
O
H

3o

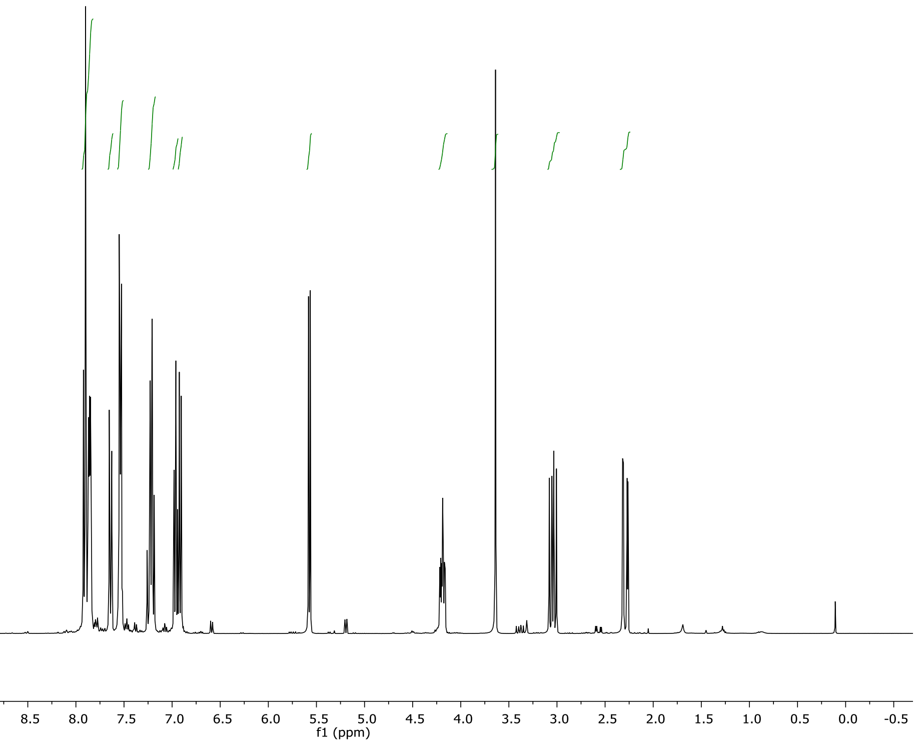
11.0 10.5 10.0 9.5 9.0 8.5 8.0 7.5 7.0 6.5 6.0 5.5 5.0 4.5 4.0 3.5 3.0 2.5 2.0 1.5 1.0 0.5 0.0 -0.5
f1 (ppm)

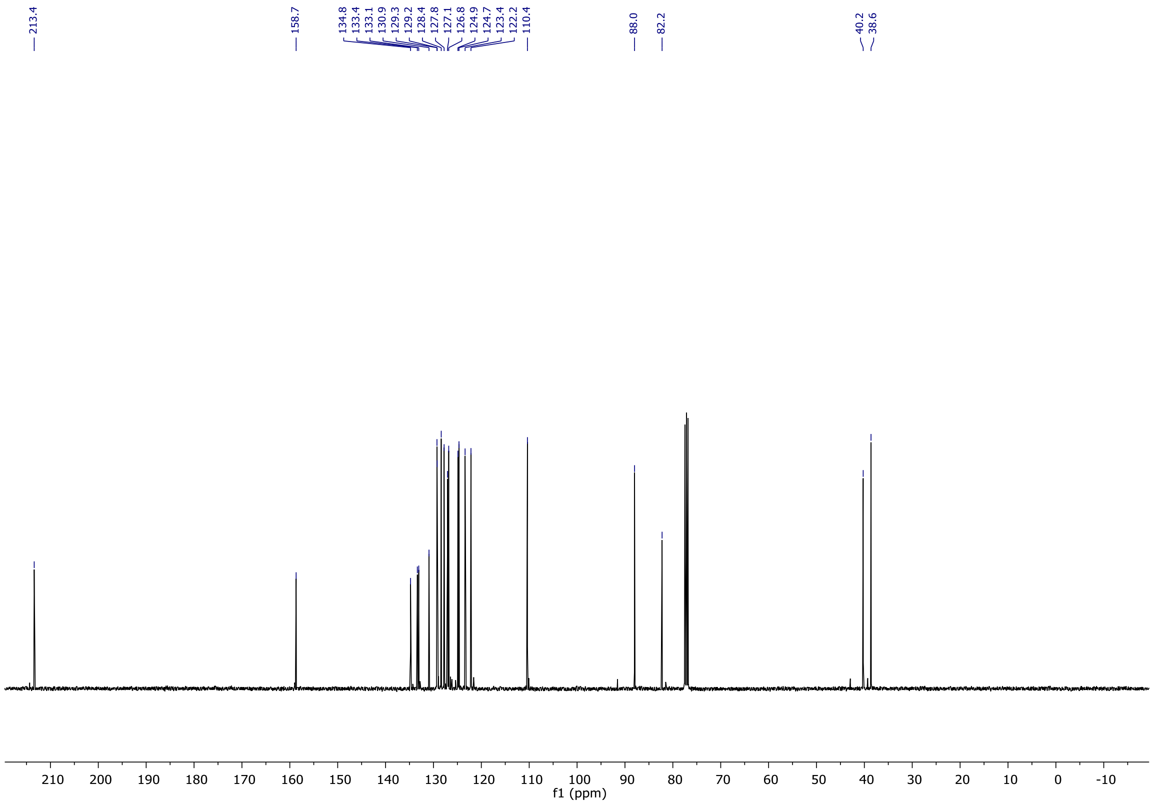
213.4
158.7
134.8
133.4
133.1
130.9
129.3
129.2
128.4
127.8
127.1
126.8
124.9
124.7
123.4
122.2
110.4
88.0
82.2
40.2
38.6
210 200 190 180 170 160 150 140 130 120 110 100 90 80 70 60 50 40 30 20 10 0 -10
f1 (ppm)

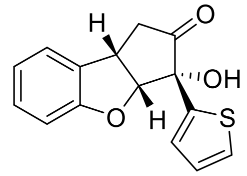
H
O
OH
S
O
H

3p

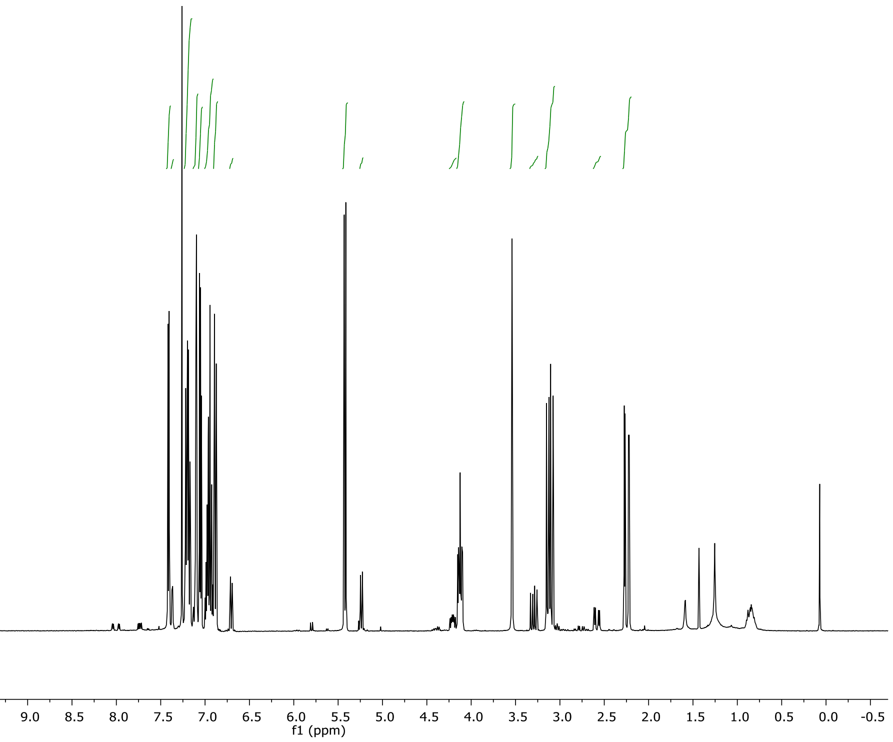
12.0 11.5 11.0 10.5 10.0 9.5 9.0 8.5 8.0 7.5 7.0 6.5 6.0 5.5 5.0 4.5 4.0 3.5 3.0 2.5 2.0 1.5 1.0 0.5 0.0 -0.5
f1 (ppm)

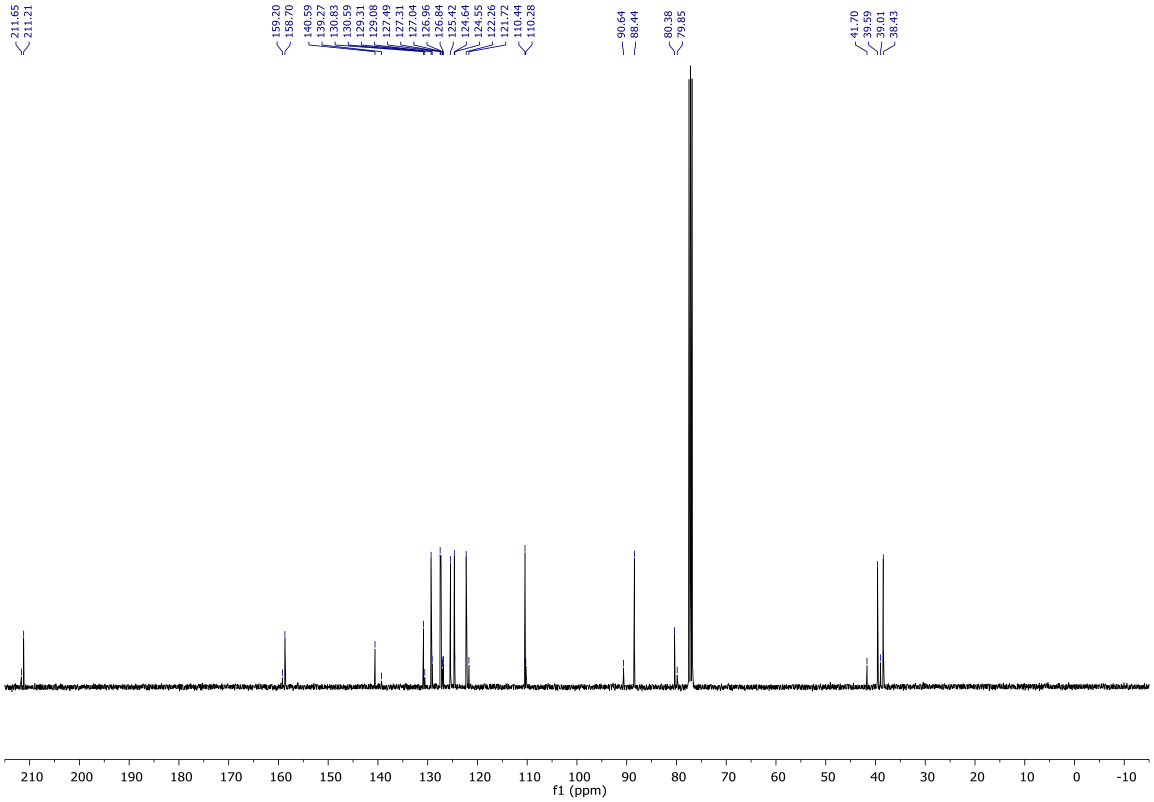
211.65
211.21
159.20
158.70
140.59
139.27
130.83
130.59
129.31
129.08
127.49
127.31
127.04
126.96
126.84
125.42
124.64
124.55
122.26
121.72
110.44
110.28
90.64
88.44
80.38
79.85
41.70
39.59
39.01
38.43
210 200 190 180 170 160 150 140 130 120 110 100 90 80 70 60 50 40 30 20 10 0 -10
f1 (ppm)

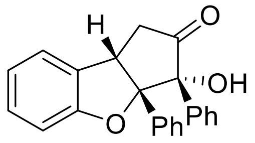

3q

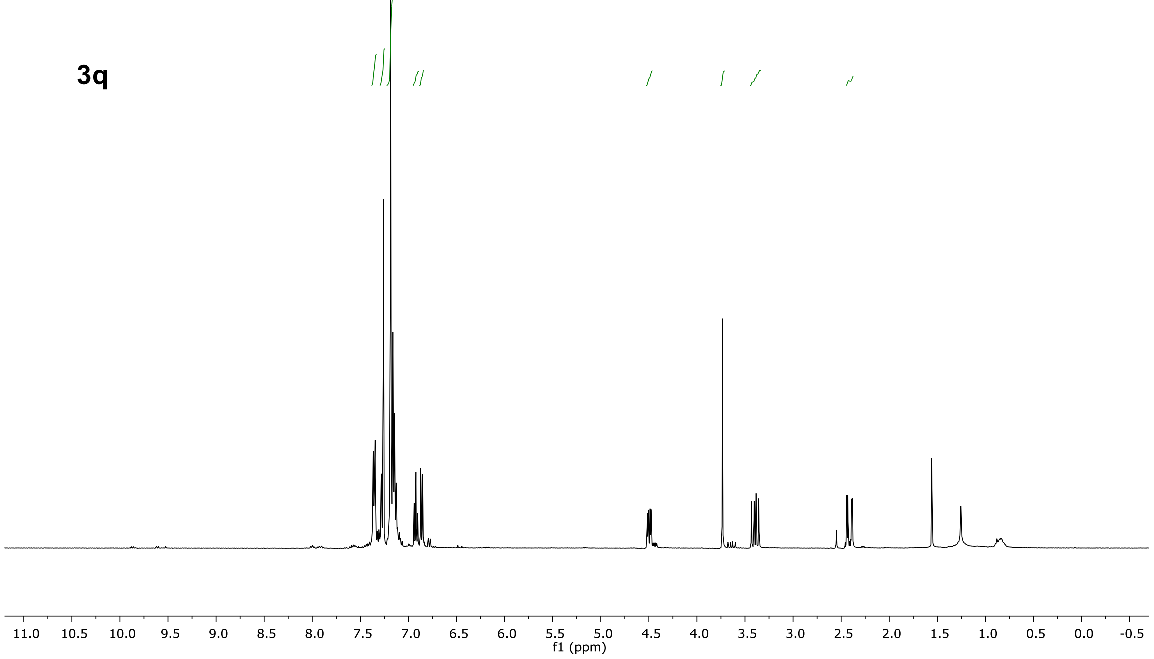

f1 (ppm)

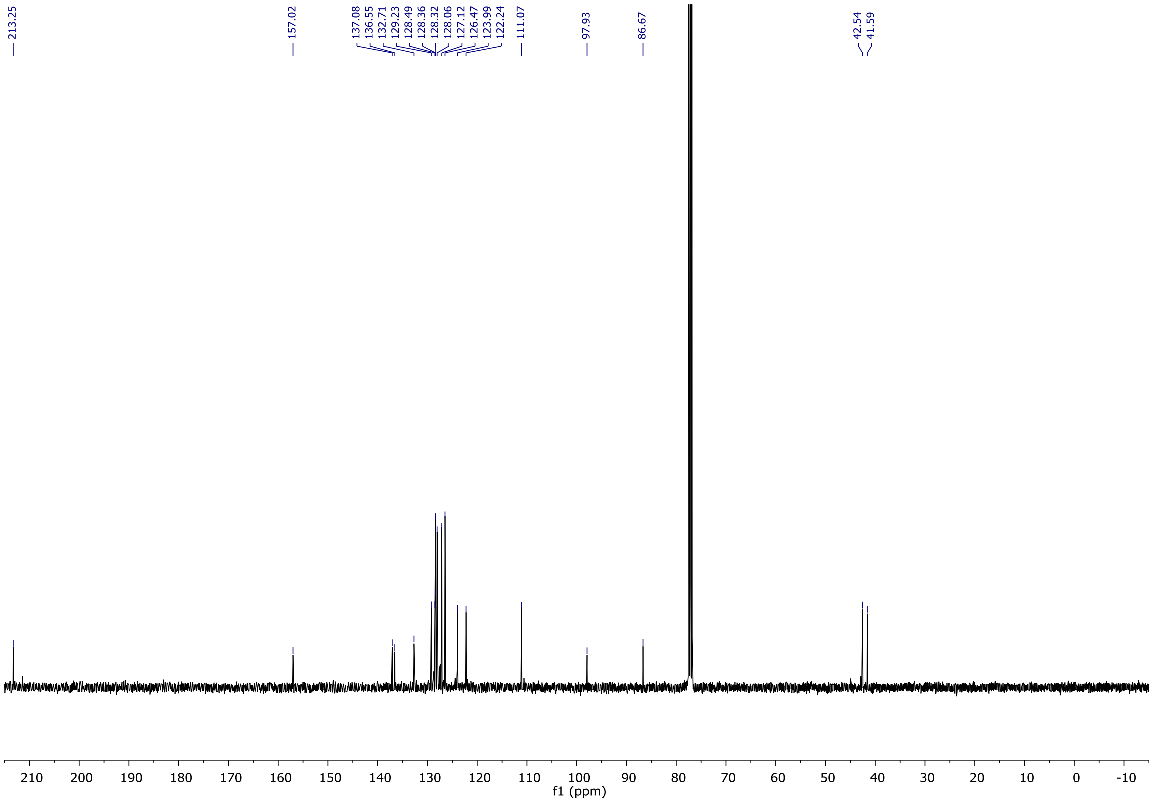

213.25
157.02
137.08
136.55
132.71
129.23
128.49
128.36
128.32
128.06
127.12
126.47
123.99
122.24
111.07
97.93
86.67
42.54
41.59
f1 (ppm)

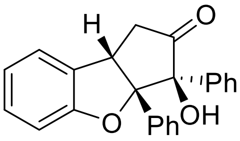
H
O
Ph
OH
O
Ph

***epi*-3q**

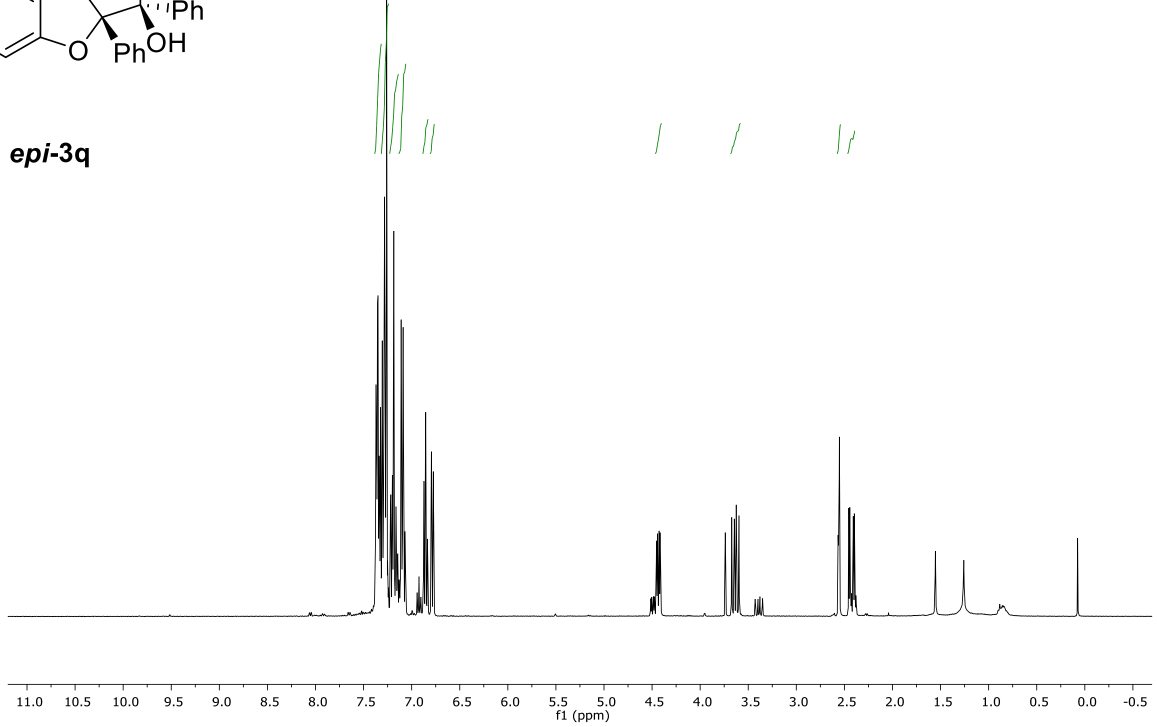
11.0 10.5 10.0 9.5 9.0 8.5 8.0 7.5 7.0 6.5 6.0 5.5 5.0 4.5 4.0 3.5 3.0 2.5 2.0 1.5 1.0 0.5 0.0 -0.5
f1 (ppm)

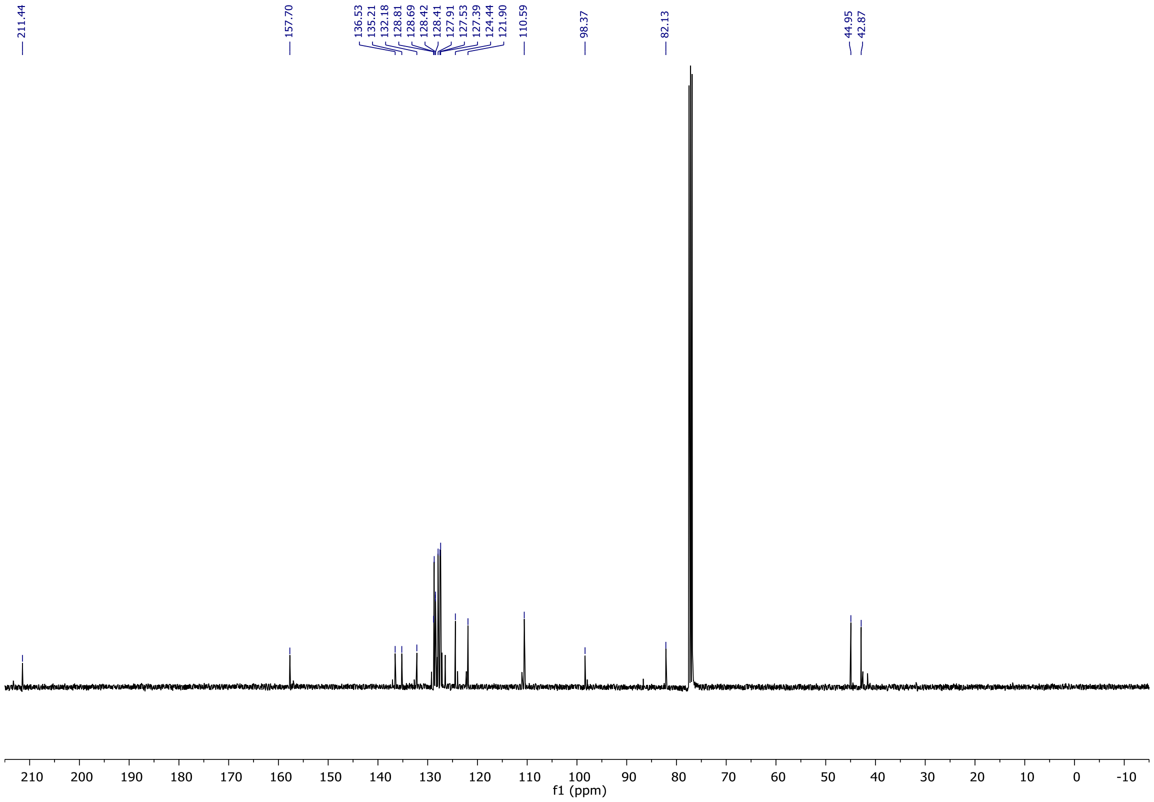
211.44
157.70
136.53
135.21
132.18
128.81
128.69
128.42
128.41
127.91
127.53
127.39
124.44
121.90
110.59
98.37
82.13
44.95
42.87
210 200 190 180 170 160 150 140 130 120 110 100 90 80 70 60 50 40 30 20 10 0 -10
f1 (ppm)

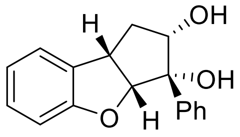
H
OH
OH
O
H
Ph

6

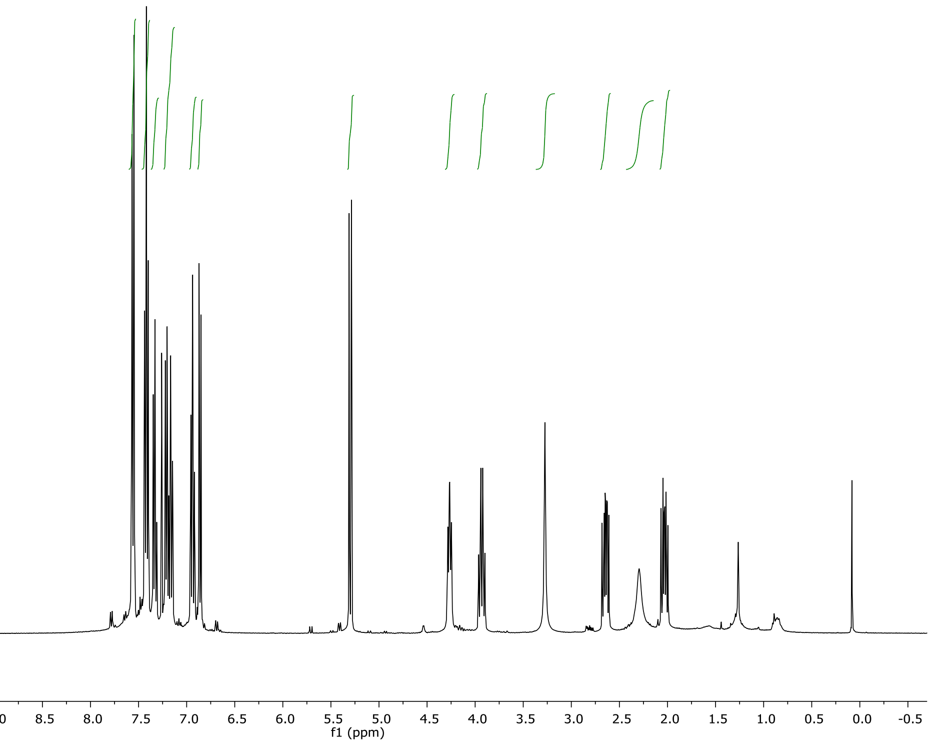
11.0 10.5 10.0 9.5 9.0 8.5 8.0 7.5 7.0 6.5 6.0 5.5 5.0 4.5 4.0 3.5 3.0 2.5 2.0 1.5 1.0 0.5 0.0 -0.5
f1 (ppm)

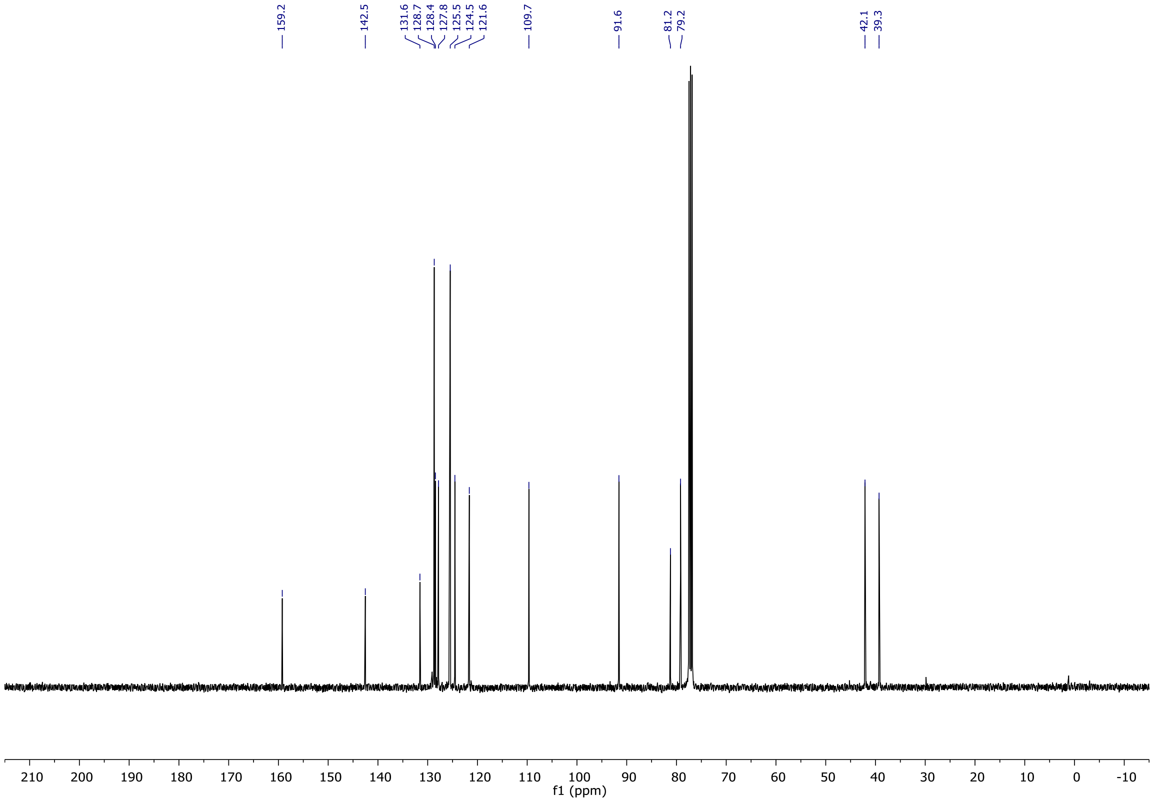
159.2
142.5
131.6
128.7
128.4
127.8
125.5
124.5
121.6
109.7
91.6
81.2
79.2
42.1
39.3
210 200 190 180 170 160 150 140 130 120 110 100 90 80 70 60 50 40 30 20 10 0 -10
f1 (ppm)

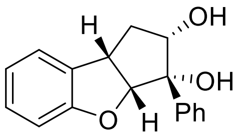

6

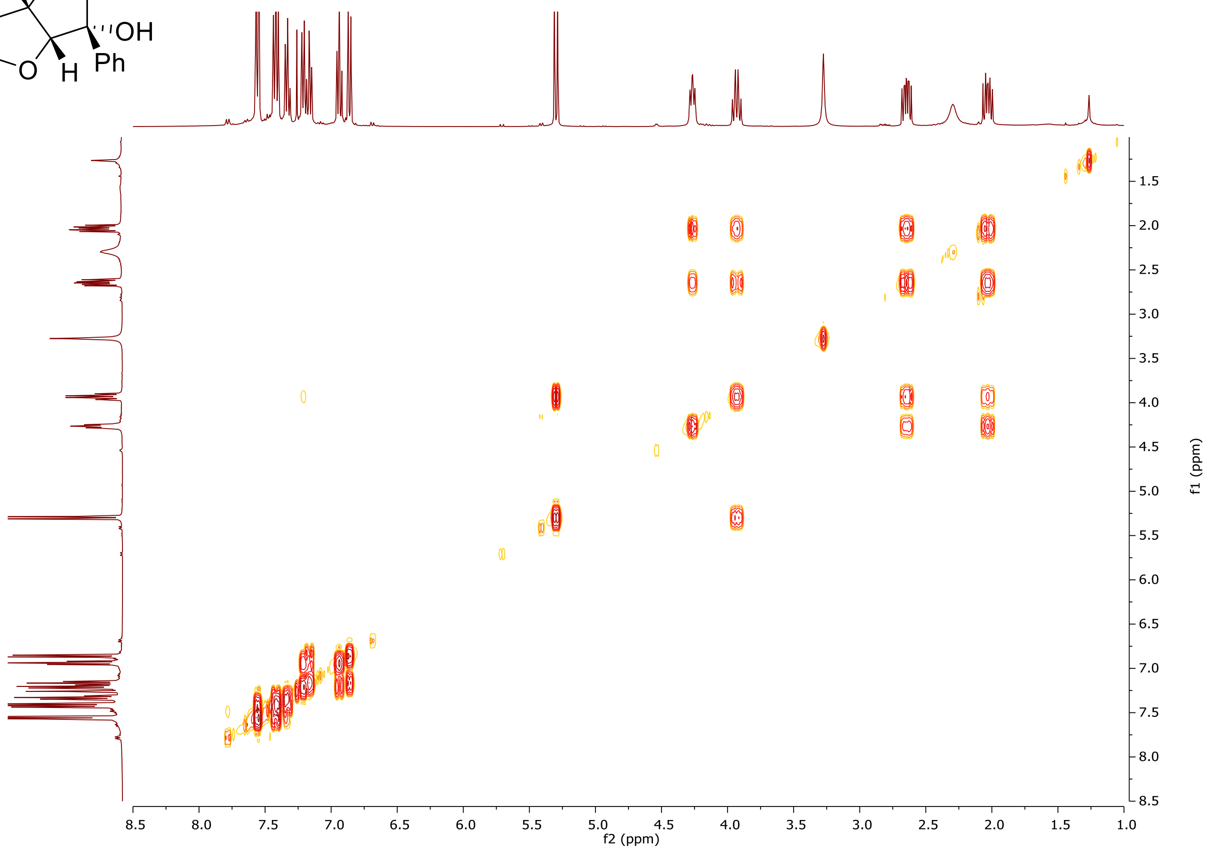

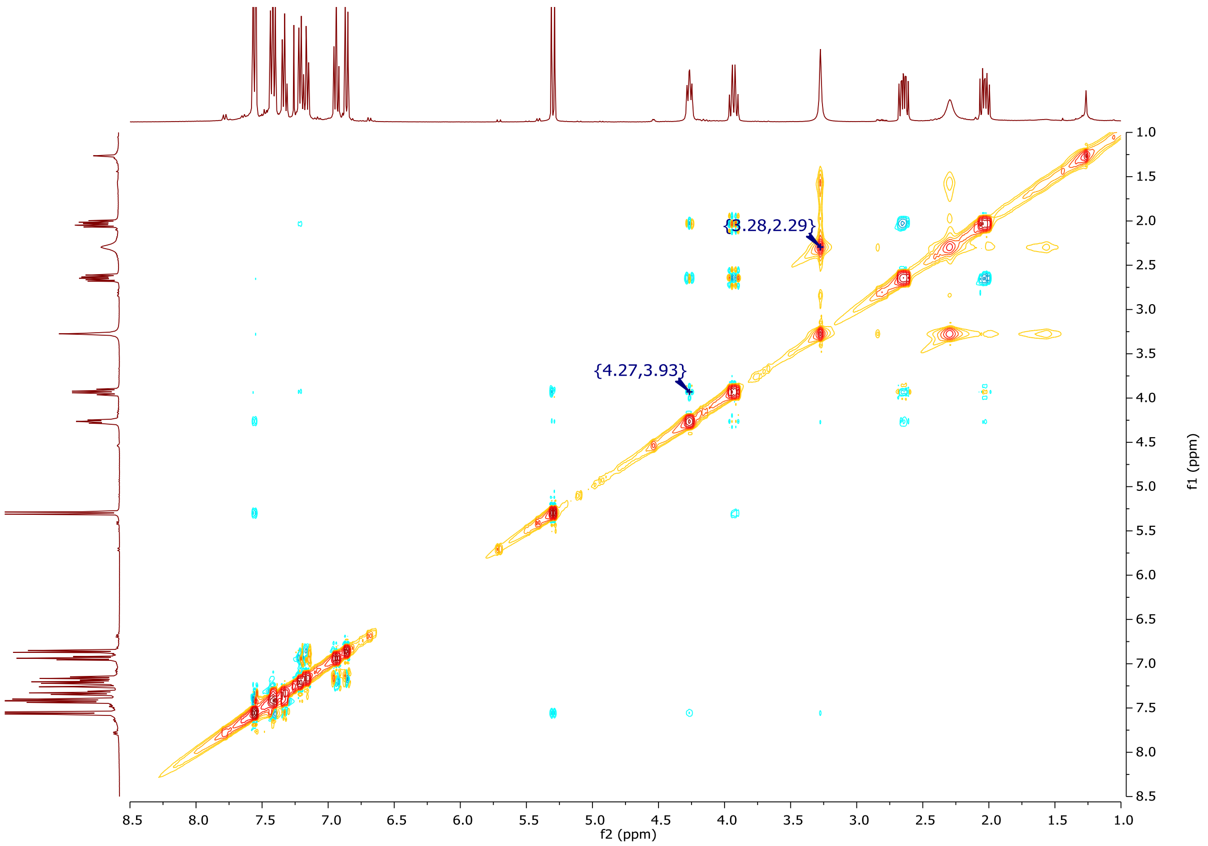

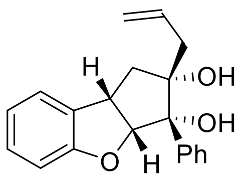
H
OH
OH
O
H
Ph

7

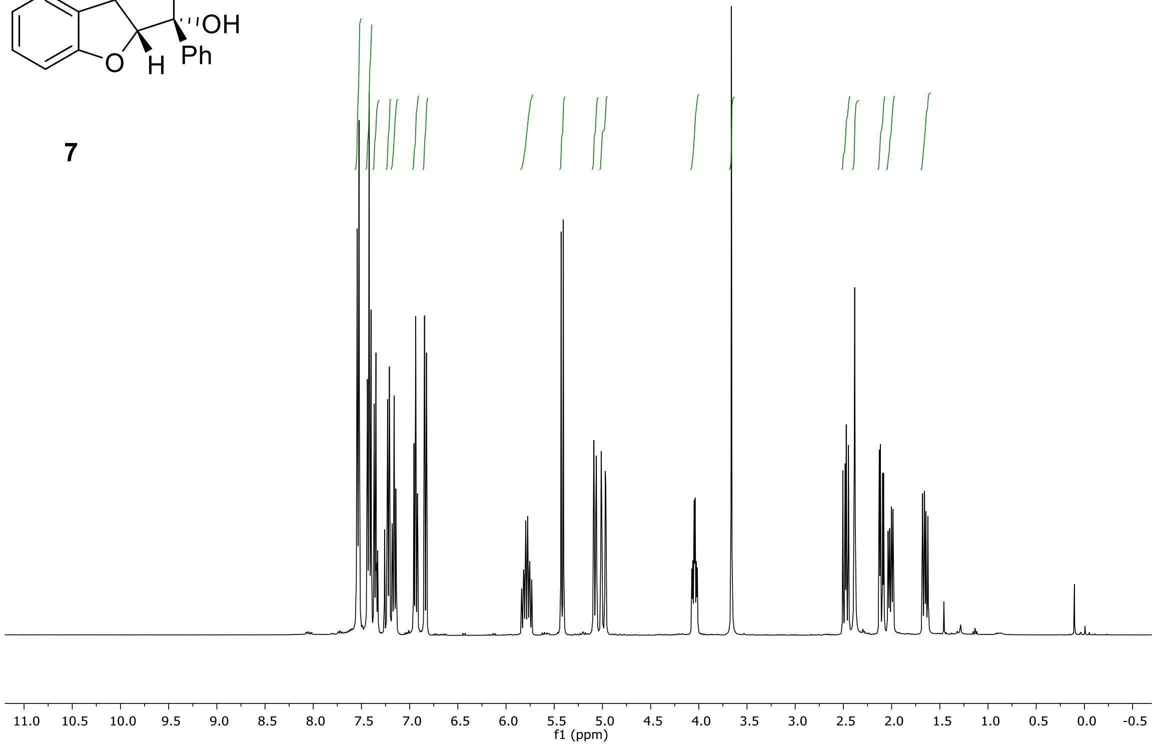
11.0 10.5 10.0 9.5 9.0 8.5 8.0 7.5 7.0 6.5 6.0 5.5 5.0 4.5 4.0 3.5 3.0 2.5 2.0 1.5 1.0 0.5 0.0 -0.5
f1 (ppm)

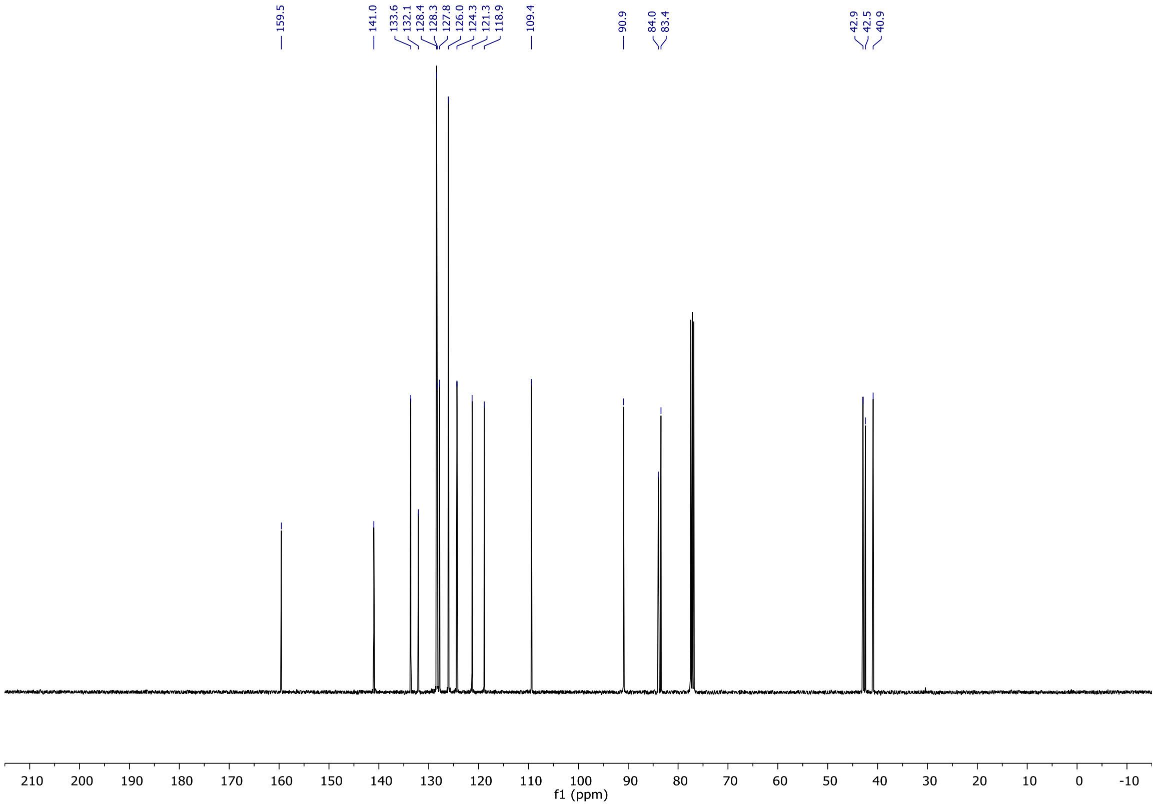
159.5
141.0
133.6
132.1
128.4
128.3
127.8
126.0
124.3
121.3
118.9
109.4
90.9
84.0
83.4
42.9
42.5
40.9
210 200 190 180 170 160 150 140 130 120 110 100 90 80 70 60 50 40 30 20 10 0 -10
f1 (ppm)

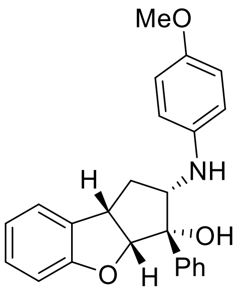
MeO
NH
H
OH
O
H
Ph

8

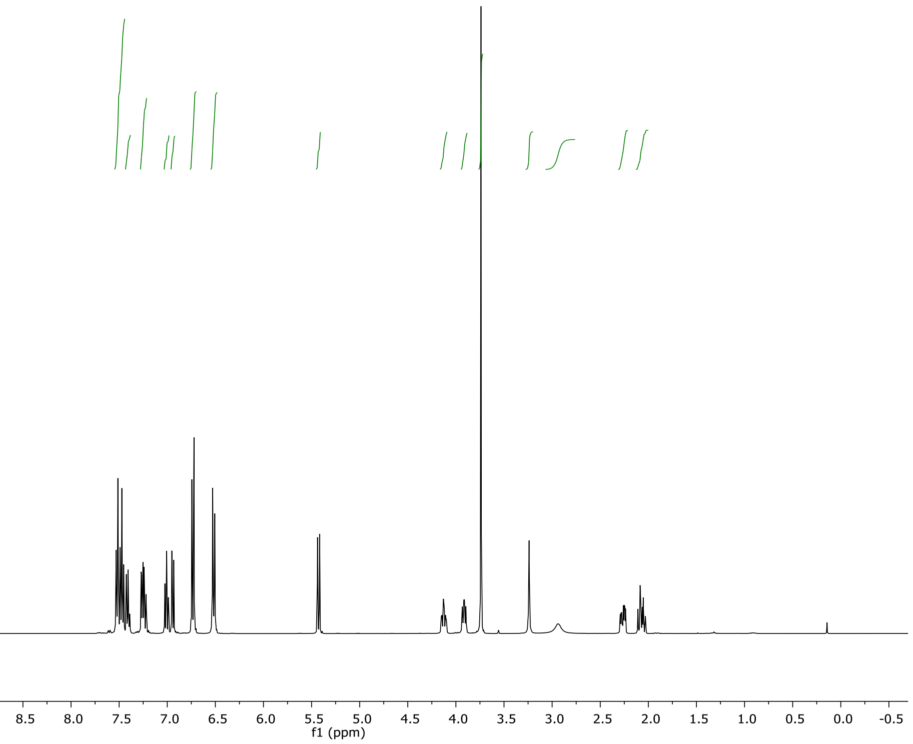
11.0 10.5 10.0 9.5 9.0 8.5 8.0 7.5 7.0 6.5 6.0 5.5 5.0 4.5 4.0 3.5 3.0 2.5 2.0 1.5 1.0 0.5 0.0 -0.5
f1 (ppm)

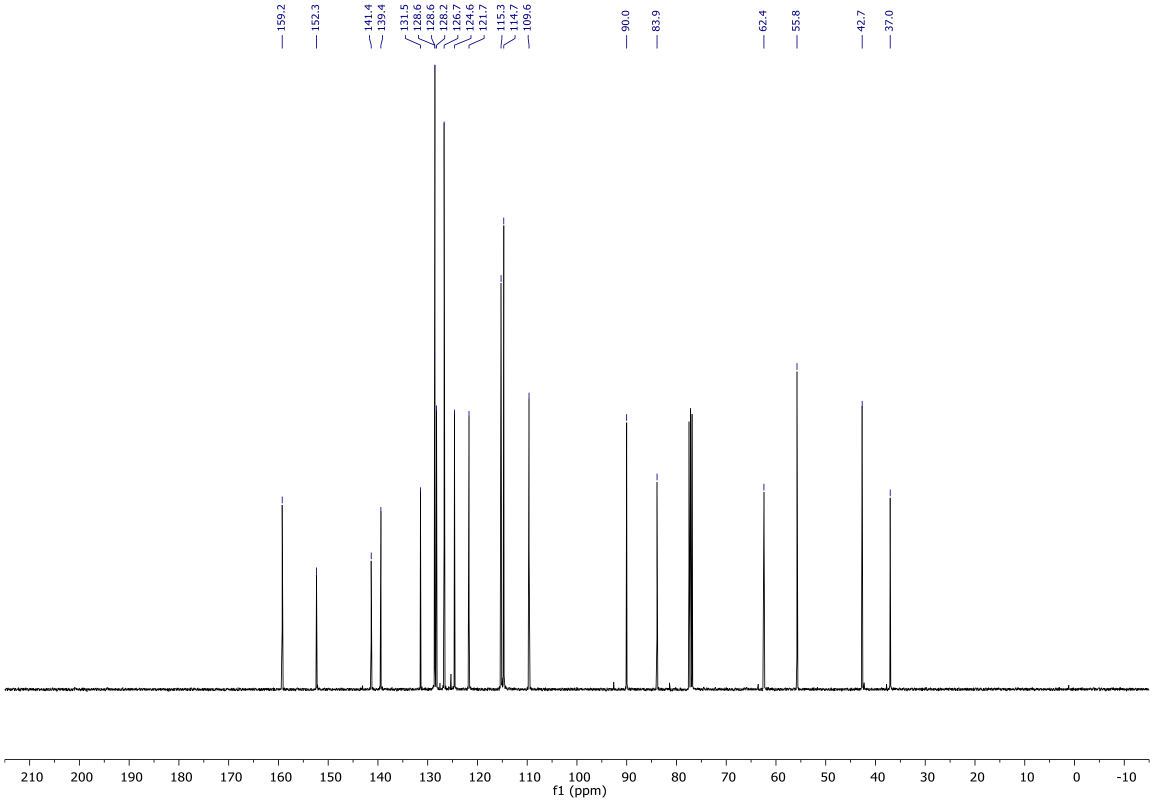
159.2
152.3
141.4
139.4
131.5
128.6
128.2
126.7
124.6
121.7
115.3
114.7
109.6
90.0
83.9
62.4
55.8
42.7
37.0
210 200 190 180 170 160 150 140 130 120 110 100 90 80 70 60 50 40 30 20 10 0 -10
f1 (ppm)

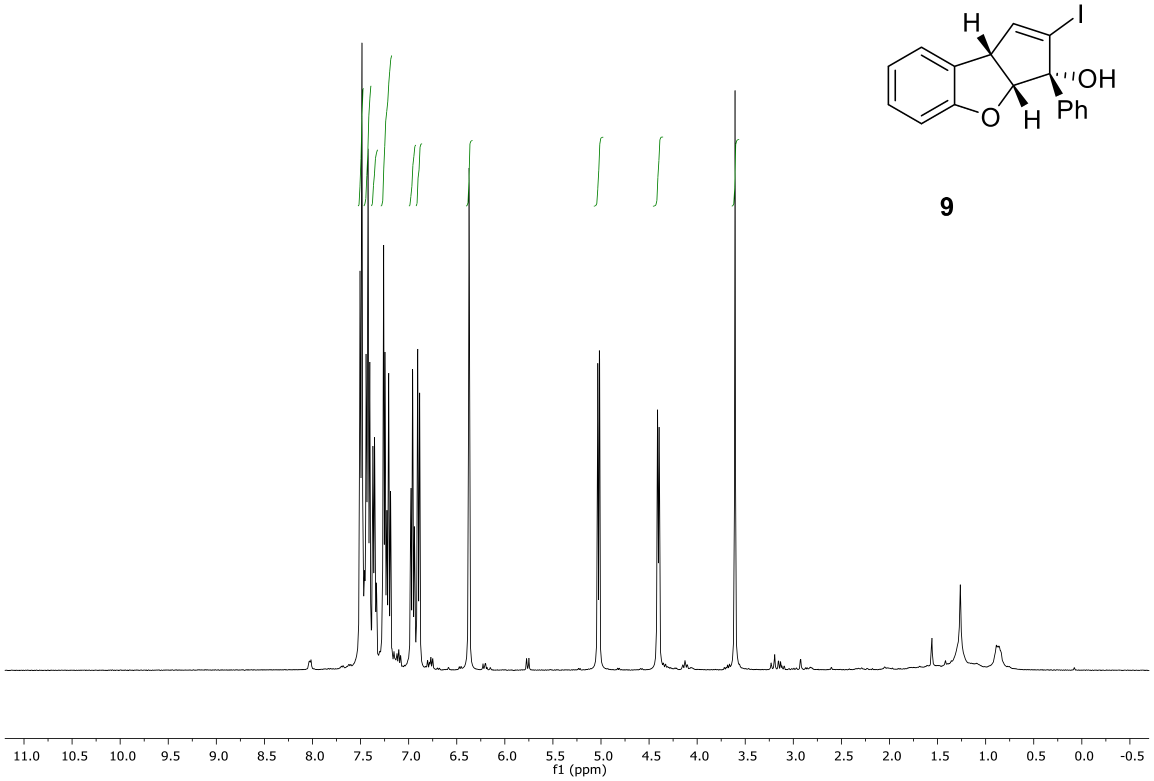
H
I
OH
Ph
O
H
9
11.0 10.5 10.0 9.5 9.0 8.5 8.0 7.5 7.0 6.5 6.0 5.5 5.0 4.5 4.0 3.5 3.0 2.5 2.0 1.5 1.0 0.5 0.0 -0.5
f1 (ppm)

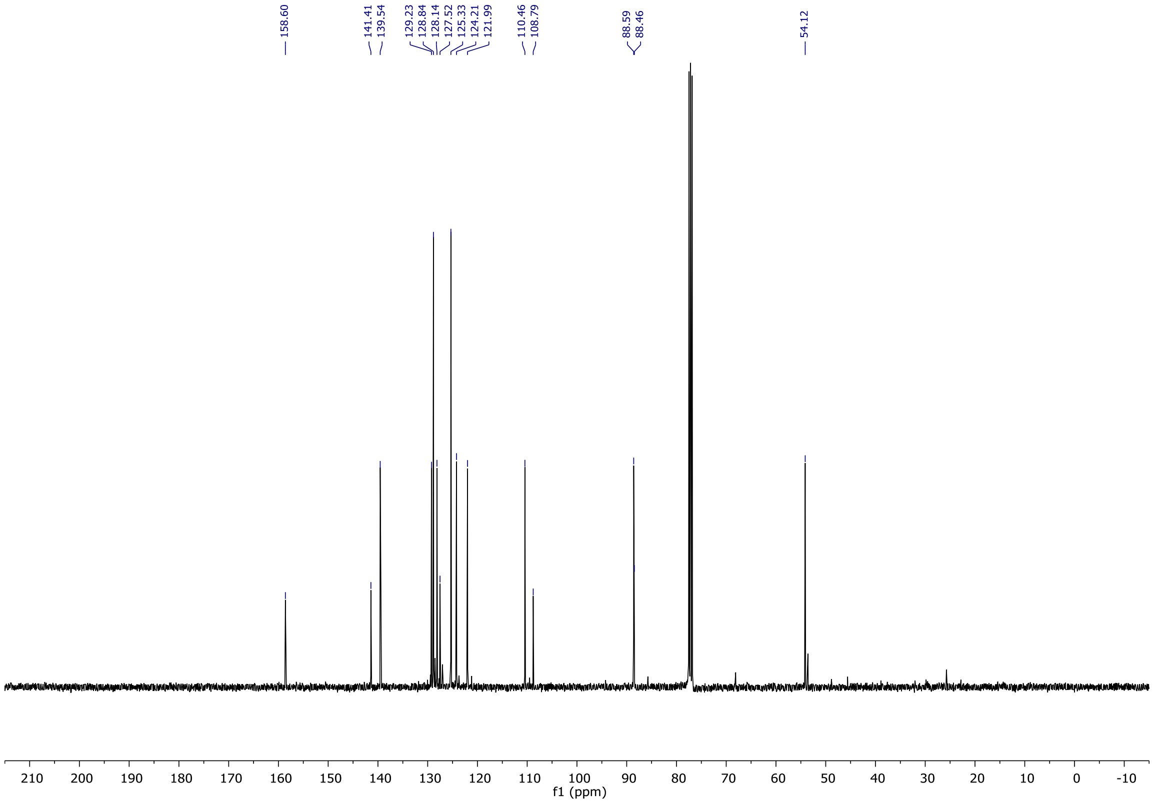
158.60
141.41
139.54
129.23
128.84
128.14
127.52
125.33
124.21
121.99
110.46
108.79
88.59
88.46
54.12
210 200 190 180 170 160 150 140 130 120 110 100 90 80 70 60 50 40 30 20 10 0 -10
f1 (ppm)

8. UPC^2 traces

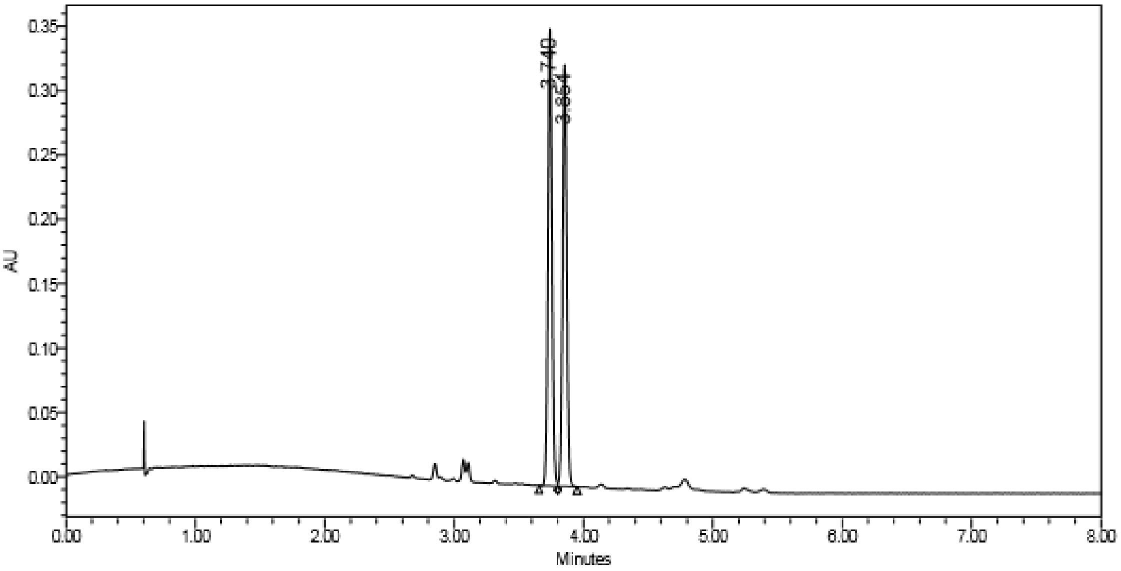

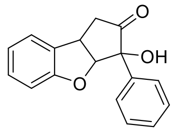

rac-3a

	Retention Time (min)	% Area
1	3.740	51.65
2	3.854	48.35

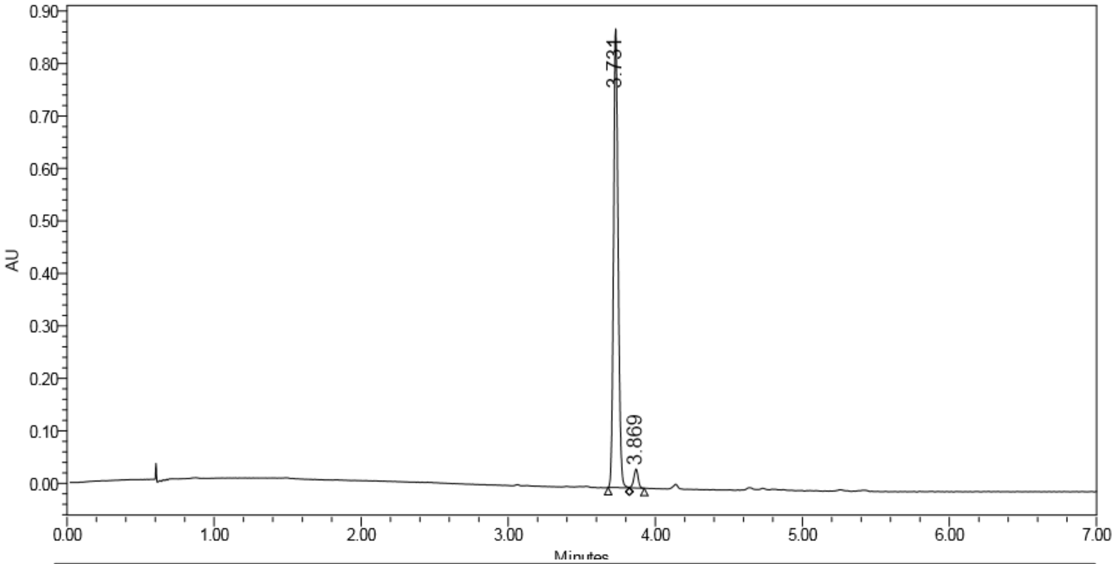

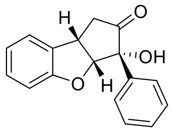

3a

	Retention Time (min)	% Area
1	3.731	96.24
2	3.869	3.76

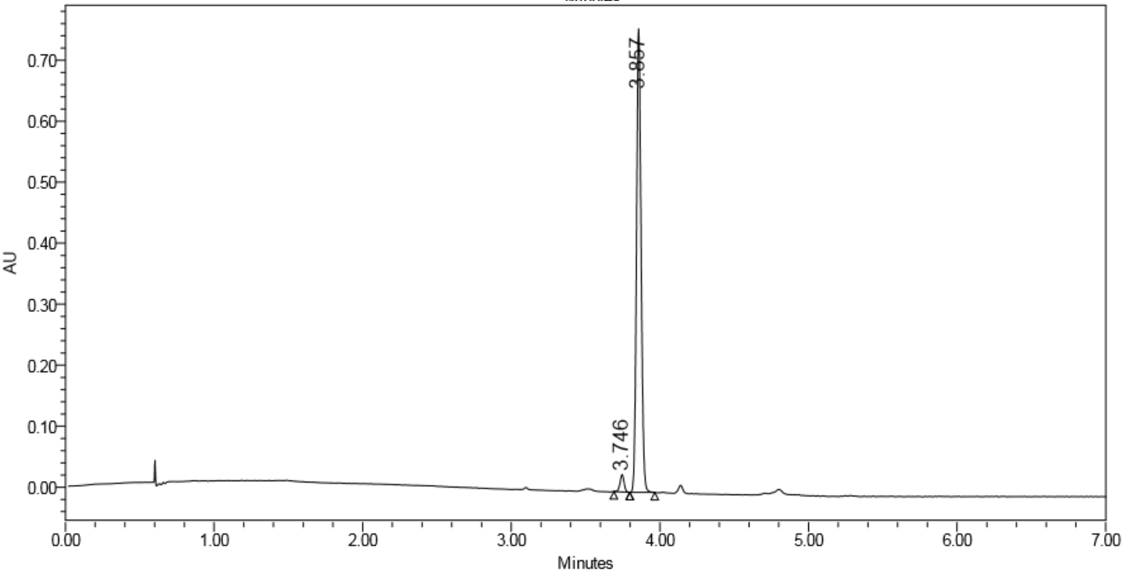

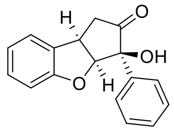

***ent*-3a**

	Retention Time (min)	% Area
1	3.746	3.24
2	3.857	96.76

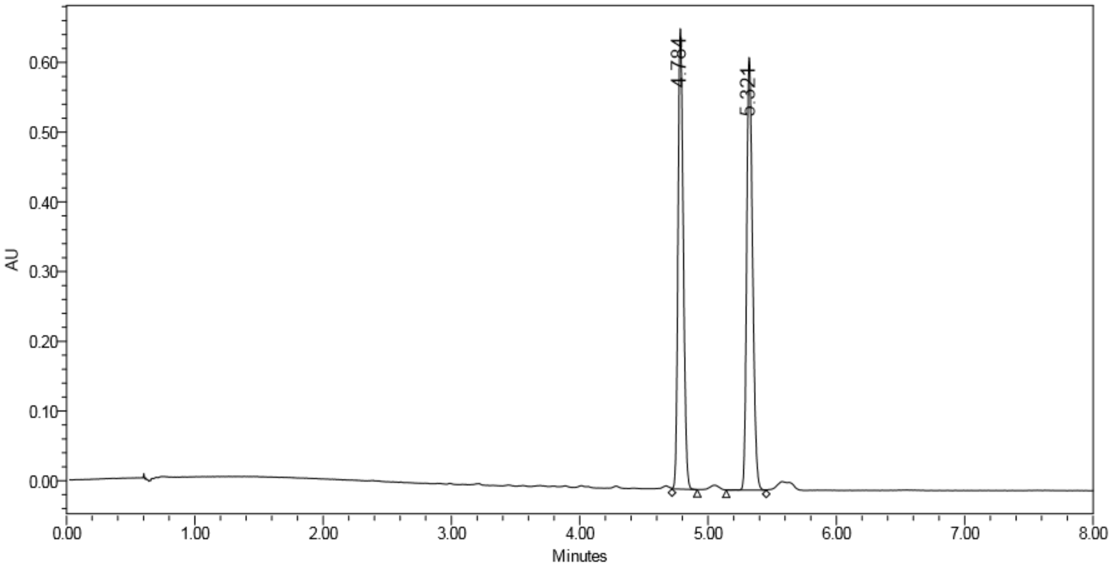

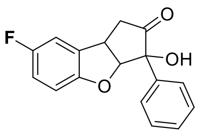

rac-3b

	Retention Time (min)	% Area
1	4.784	48.68
2	5.321	51.32

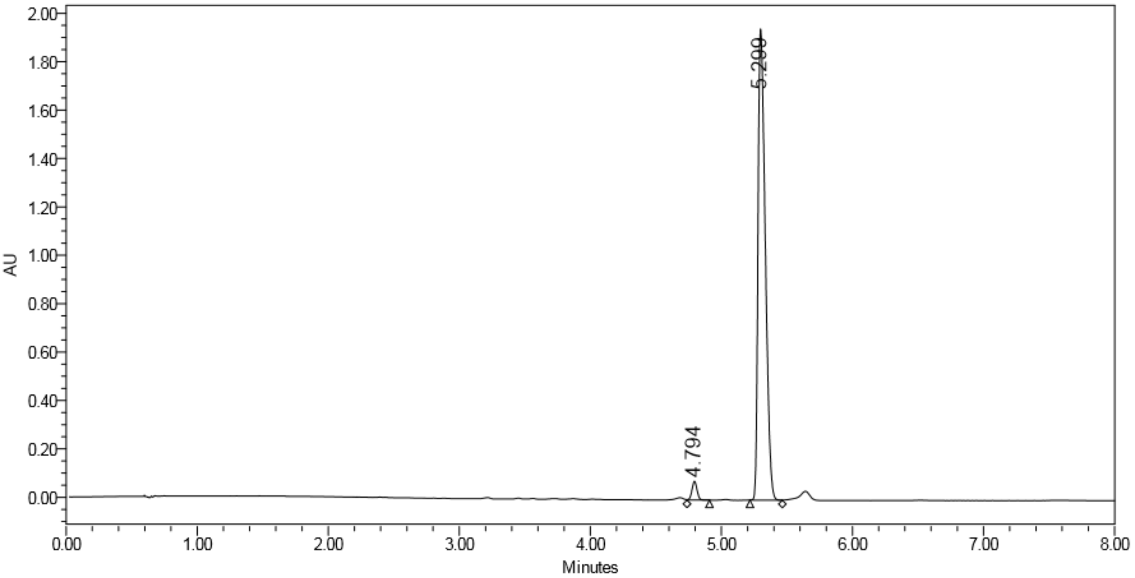

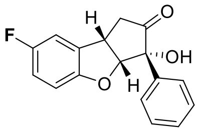

3b

	Retention Time (min)	% Area
1	4.794	2.67
2	5.299	97.33

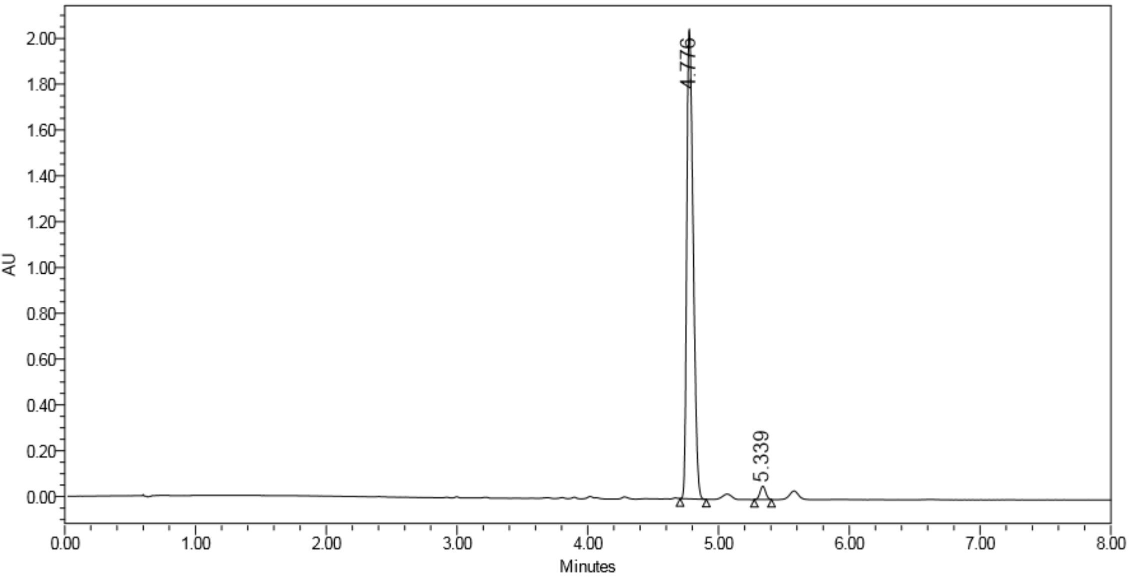

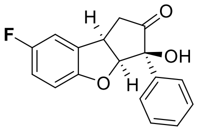

***ent*-3b**

	Retention Time (min)	% Area
1	4.776	97.57
2	5.339	2.43

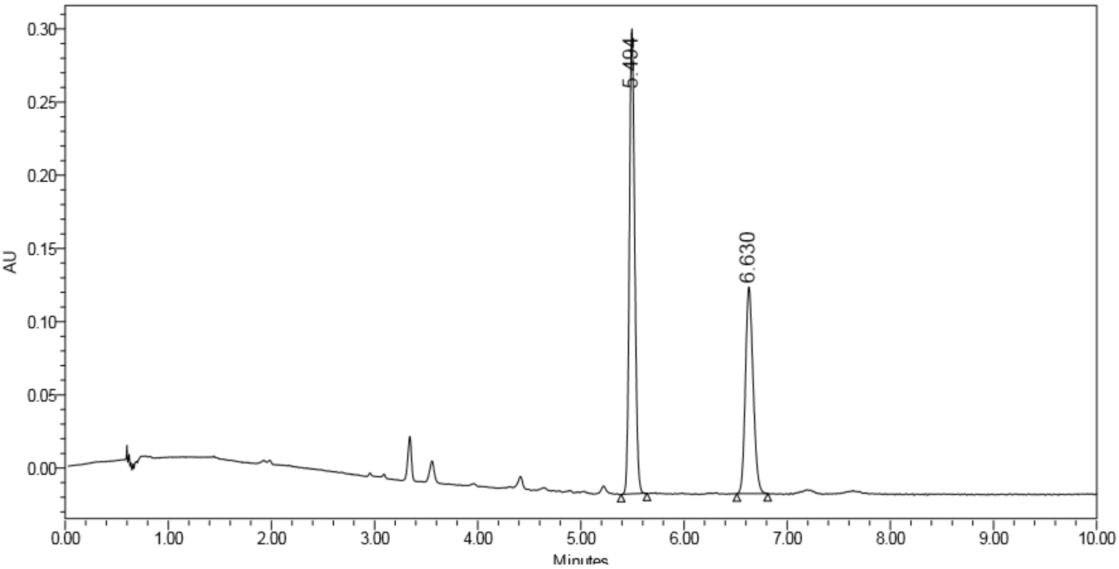

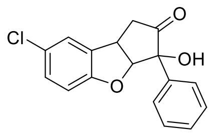

rac-3c

	Retention Time (min)	% Area
1	5.494	60.96
2	6.630	39.04

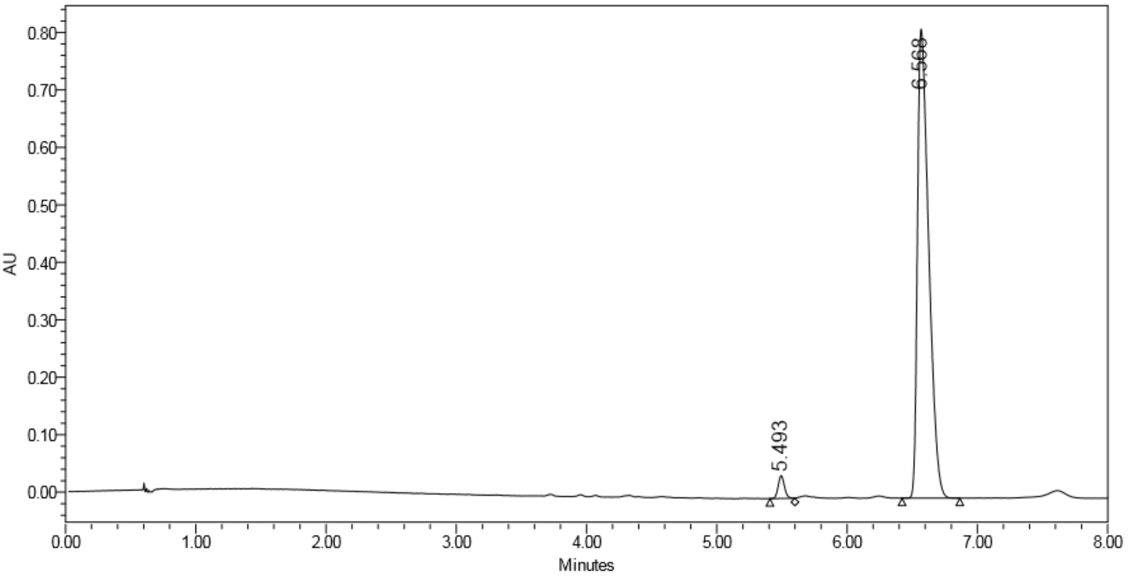

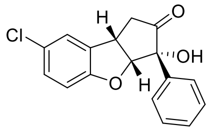

3c

	Retention Time (min)	% Area
1	5.493	2.63
2	6.568	97.37

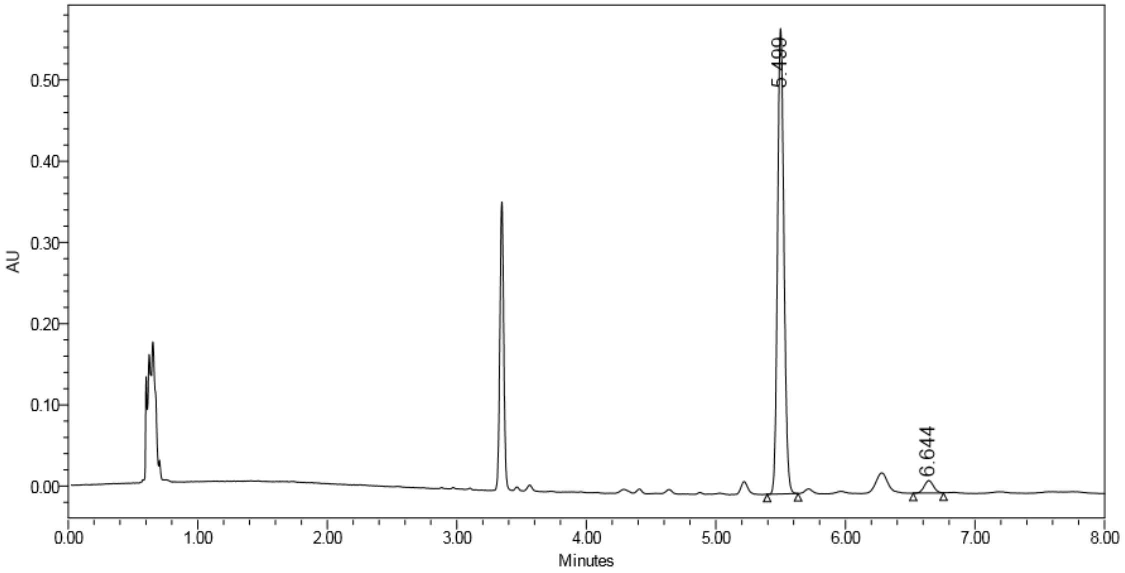

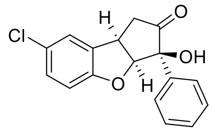

***ent*-3c**

	Retention Time (min)	% Area
1	5.499	96.23
2	6.644	3.77

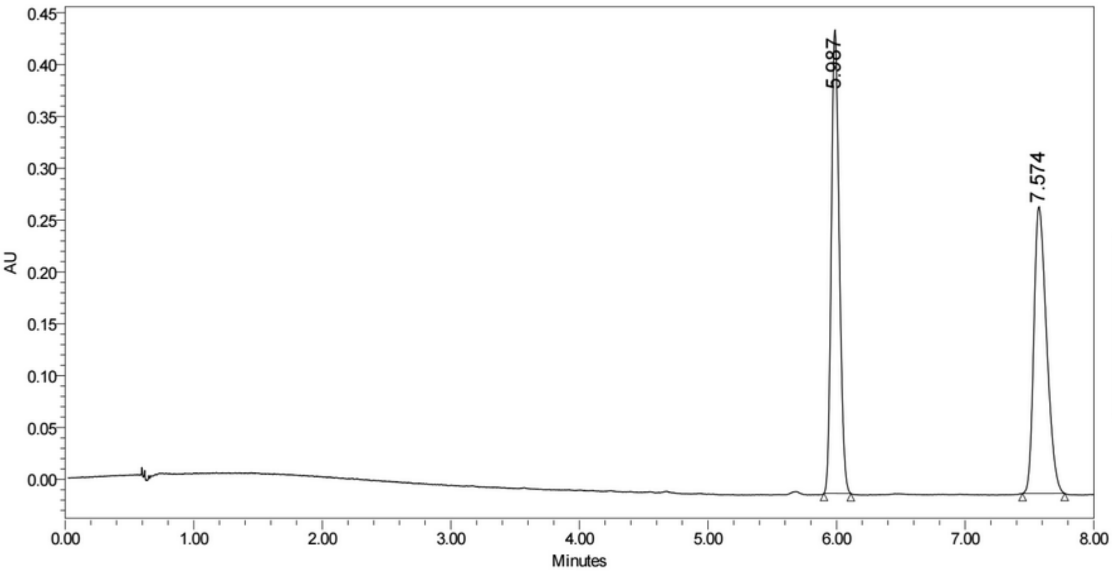

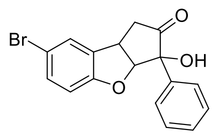

rac-3d

	Retention Time (min)	% Area
1	5.987	50.35
2	7.574	49.65

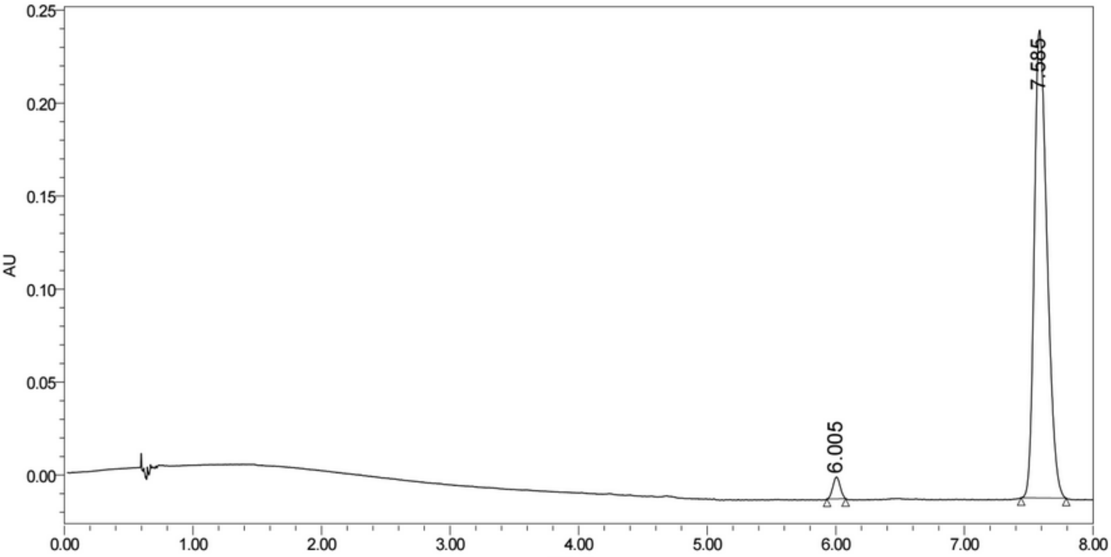

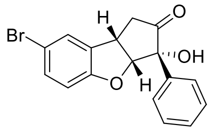

3d

	Retention Time (min)	% Area
1	6.005	2.60
2	7.585	97.40

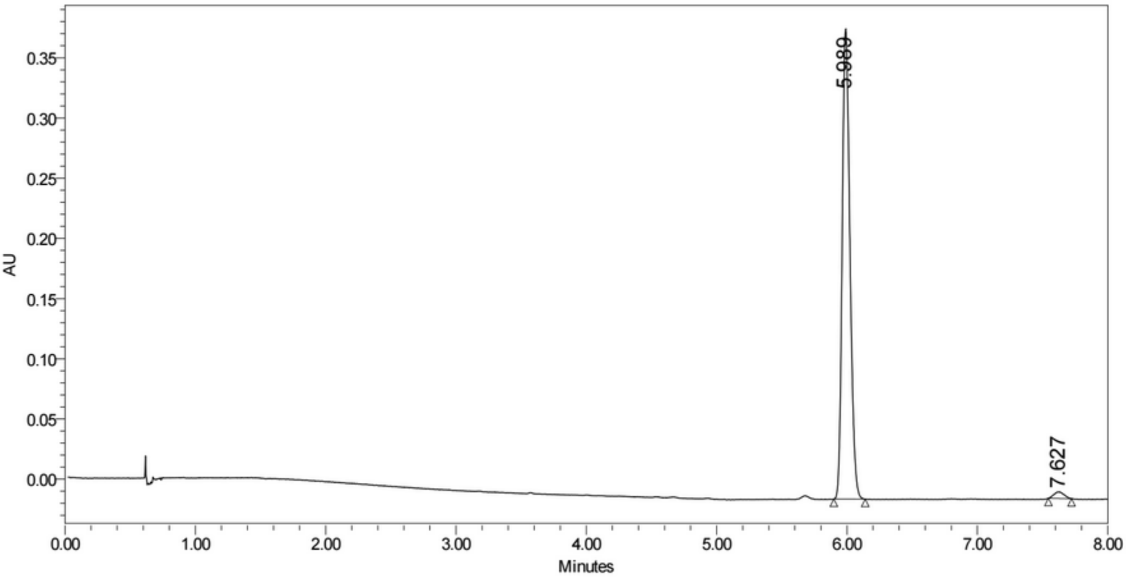

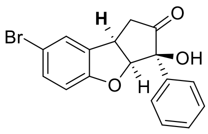

***ent*-3d**

	Retention Time (min)	% Area
1	5.989	98.21
2	7.627	1.79

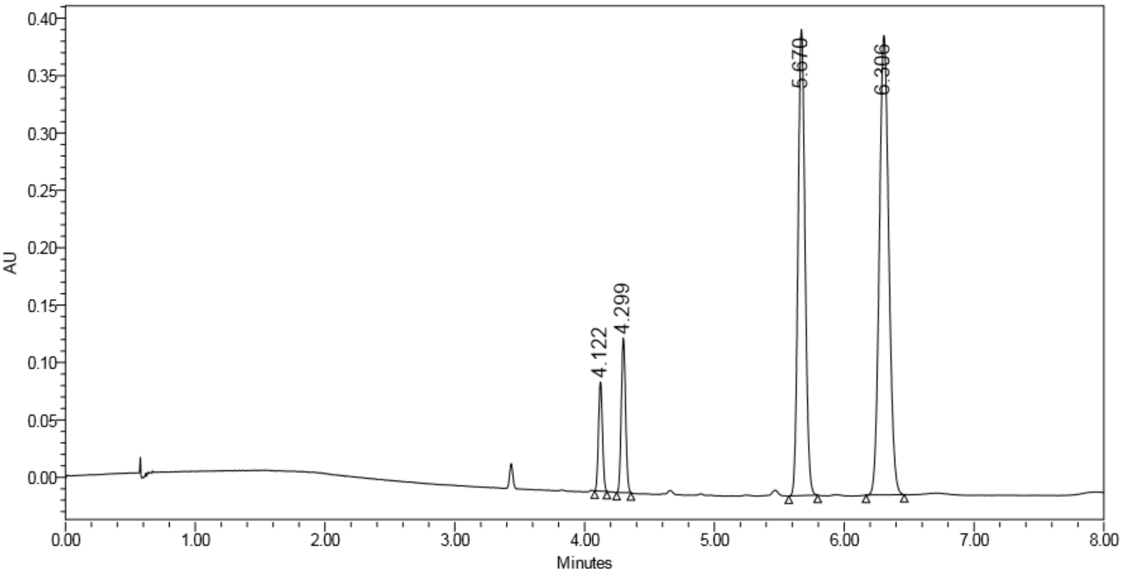

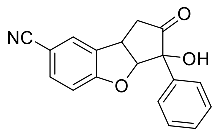

rac-3e

	Retention Time (min)	% Area
1	4.122	5.03
2	4.299	7.63
3	5.670	37.54
4	6.306	49.81

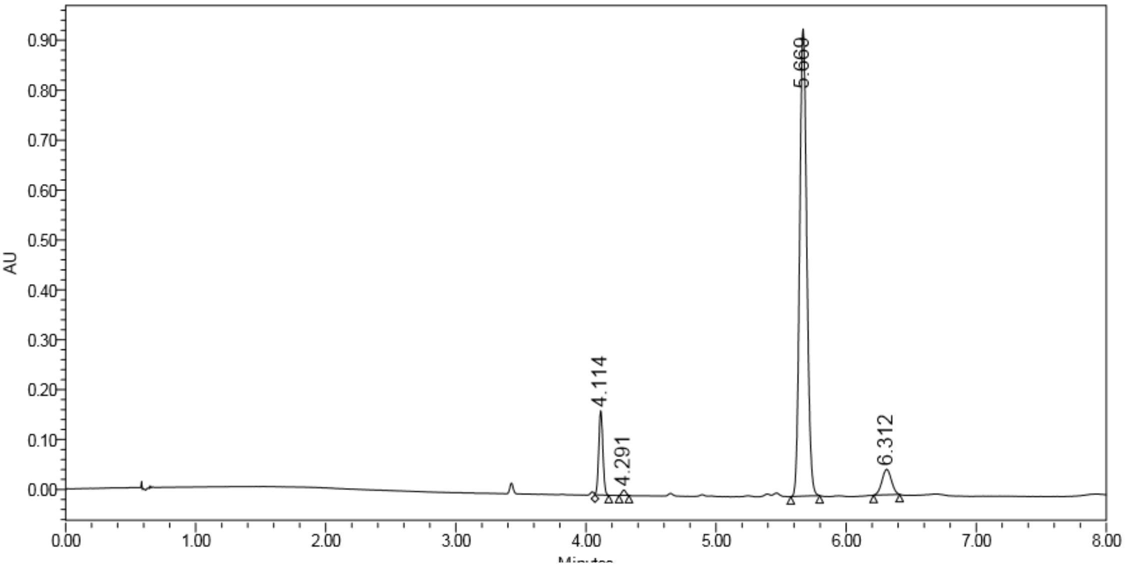

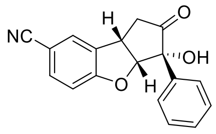

3e

	Retention Time (min)	% Area
1	4.114	8.82
2	4.291	0.55
3	5.669	84.55
4	6.312	6.08

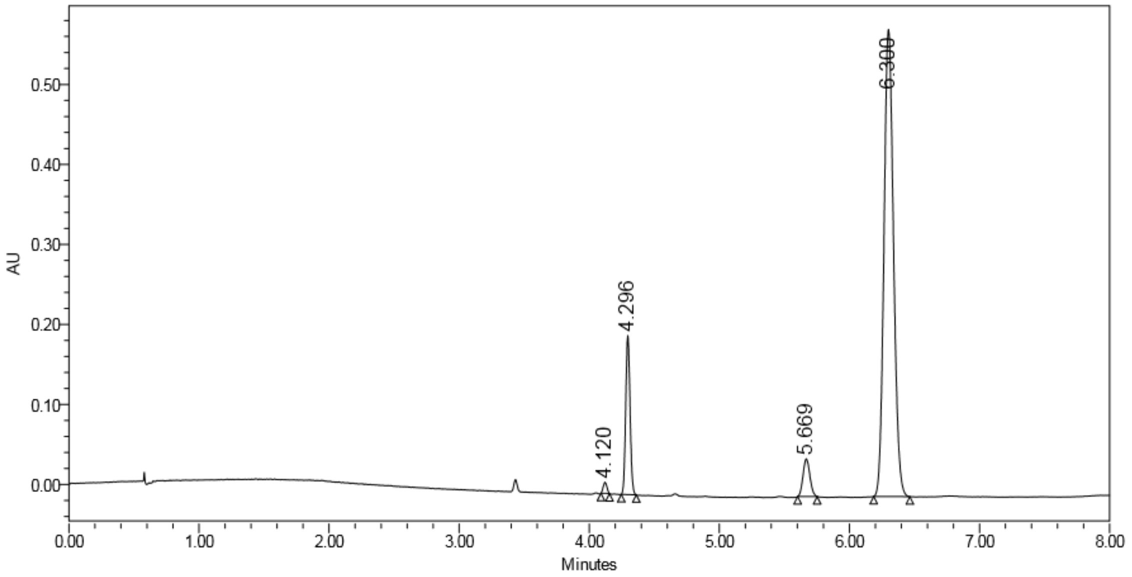

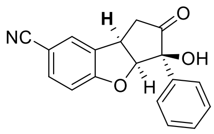

***ent*-3e**

	Retention Time (min)	% Area
1	4.120	0.76
2	4.296	12.72
3	5.669	4.76
4	6.300	81.76

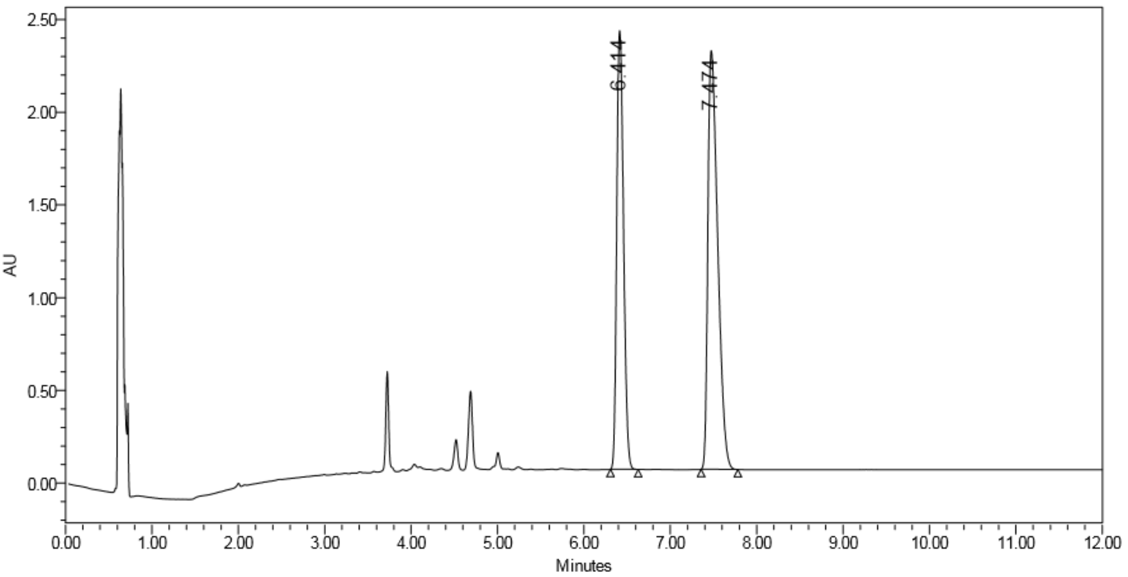

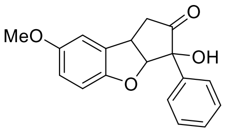

rac-3f

	Retention Time (min)	% Area
1	6.414	41.95
2	7.474	58.05

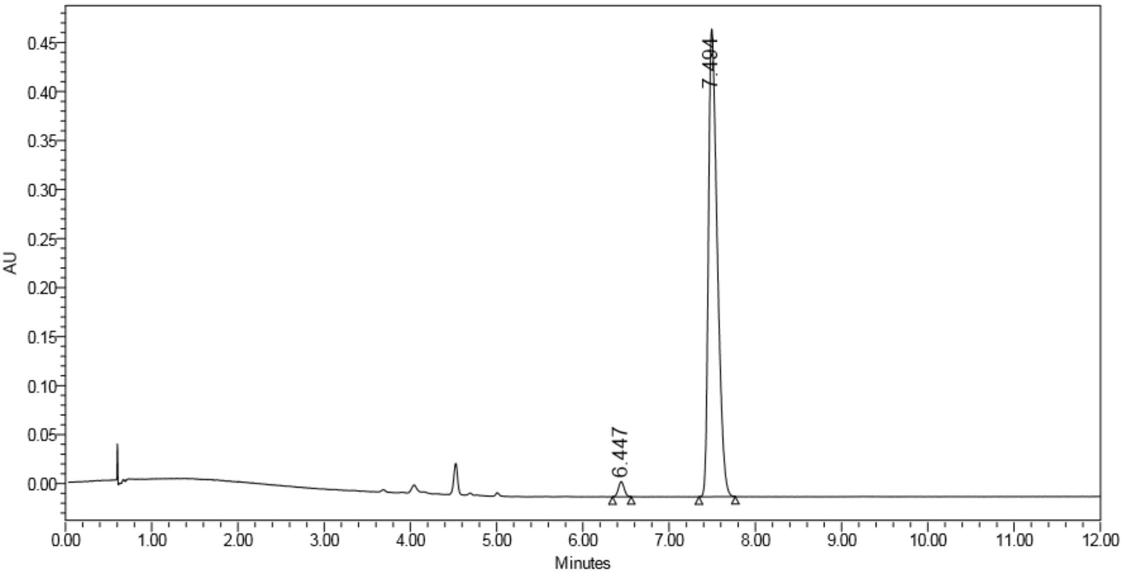

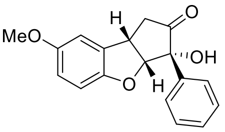

3f

	Retention Time (min)	% Area
1	6.447	2.09
2	7.494	97.91

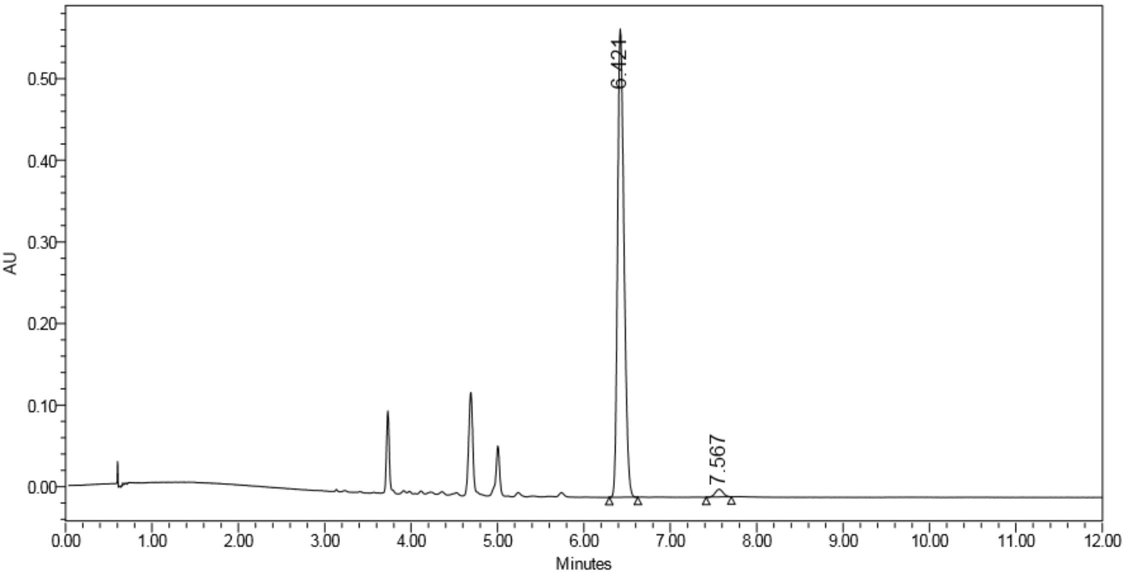

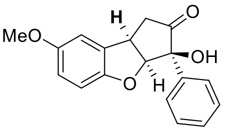

***ent*-3f**

	Retention Time (min)	% Area
1	6.421	98.07
2	7.567	1.93

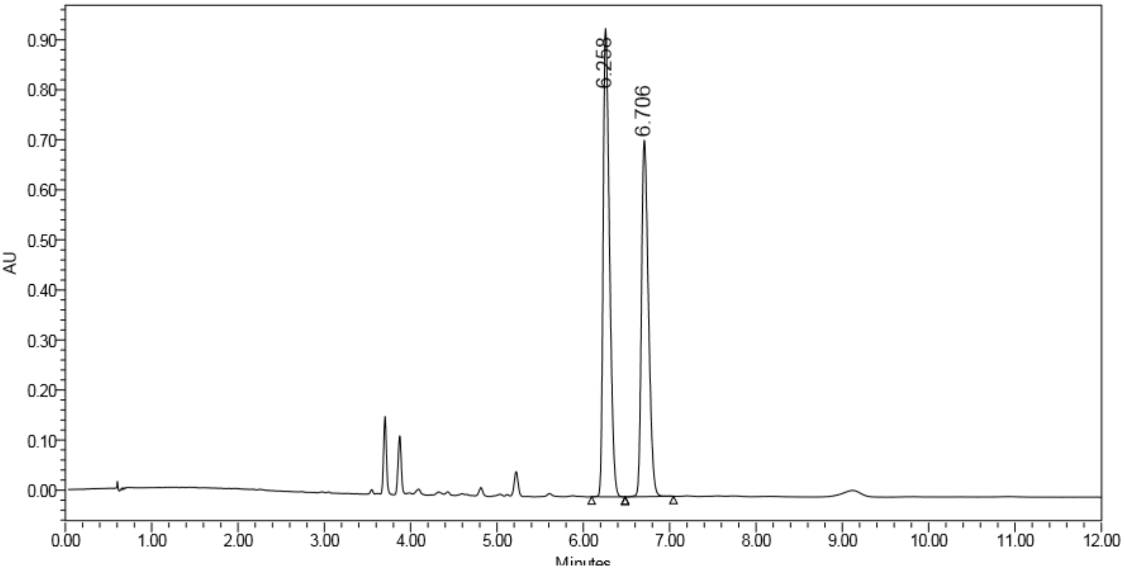

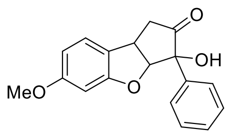

rac-3g

	Retention Time (min)	% Area
1	6.258	54.35
2	6.706	45.65

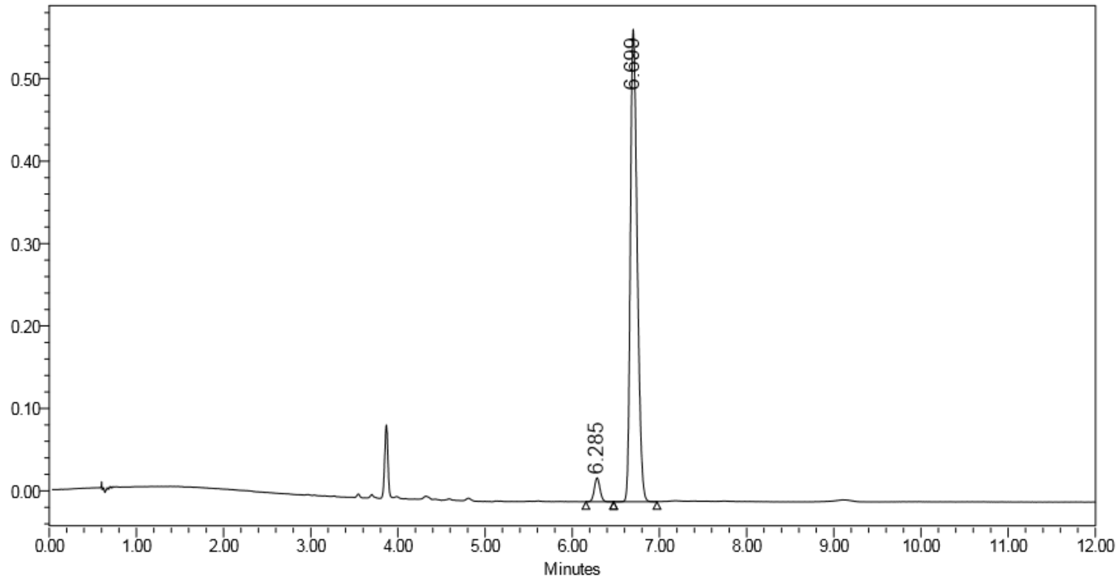

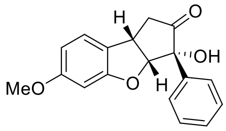

3g

	Retention Time (min)	% Area
1	6.285	4.08
2	6.699	95.92

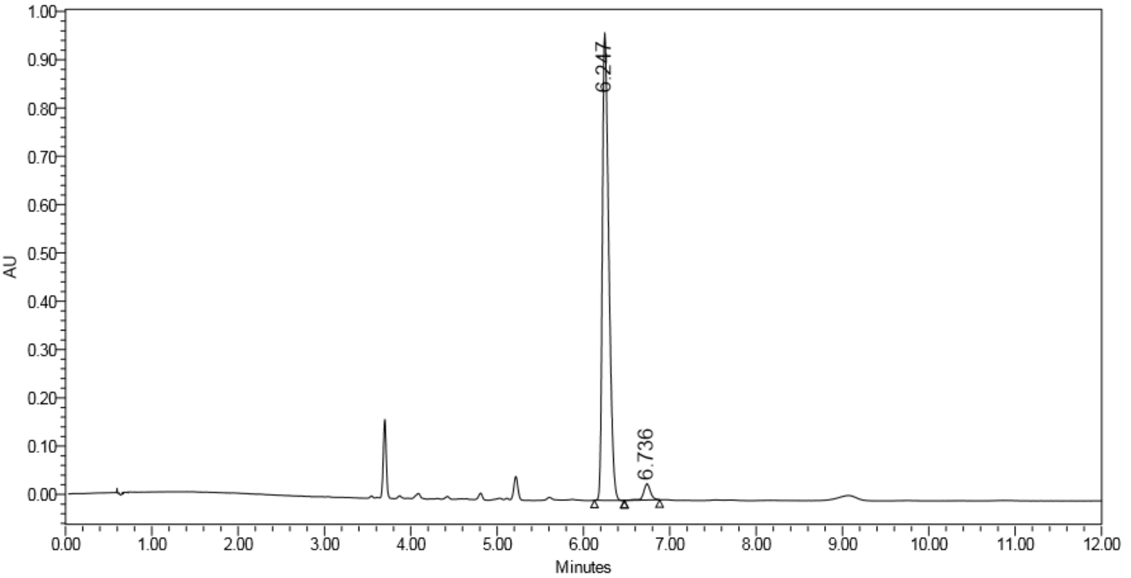

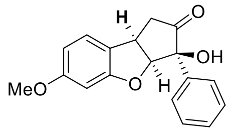

ent-3g

	Retention Time (min)	% Area
1	6.247	96.44
2	6.736	3.56

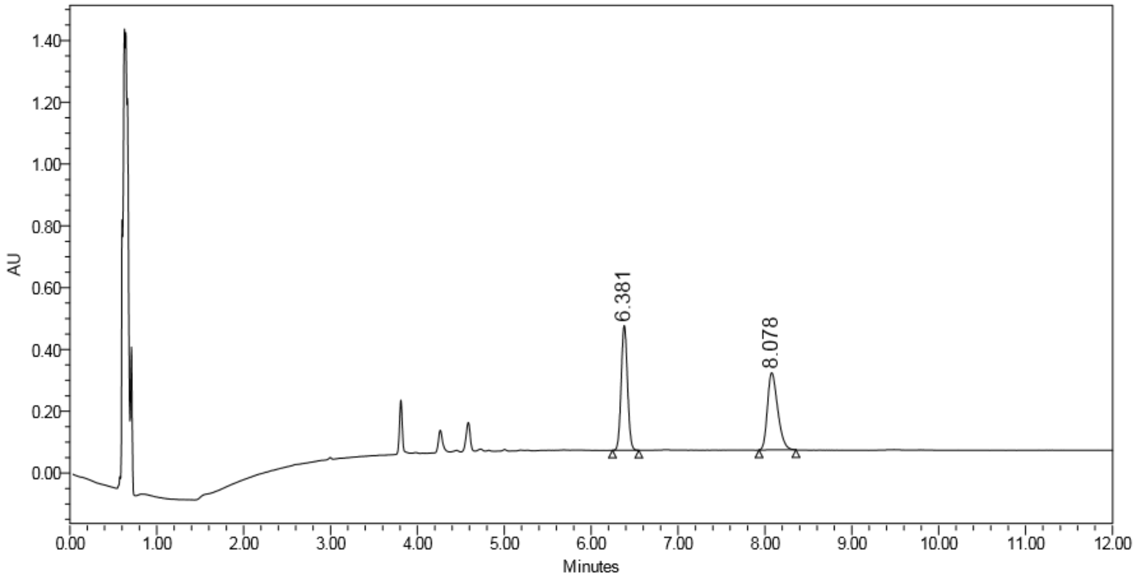

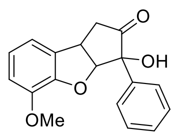

rac-3h

	Retention Time (min)	% Area
1	6.381	49.80
2	8.078	50.20

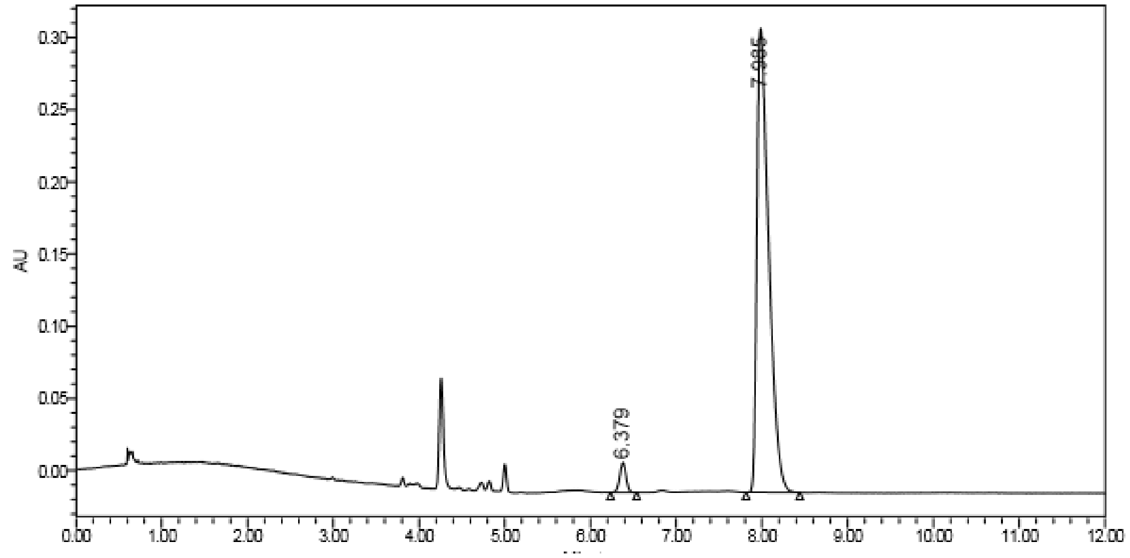

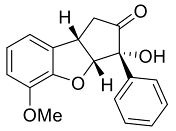

3h

	Retention Time (min)	% Area
1	6.379	3.27
2	7.985	96.73

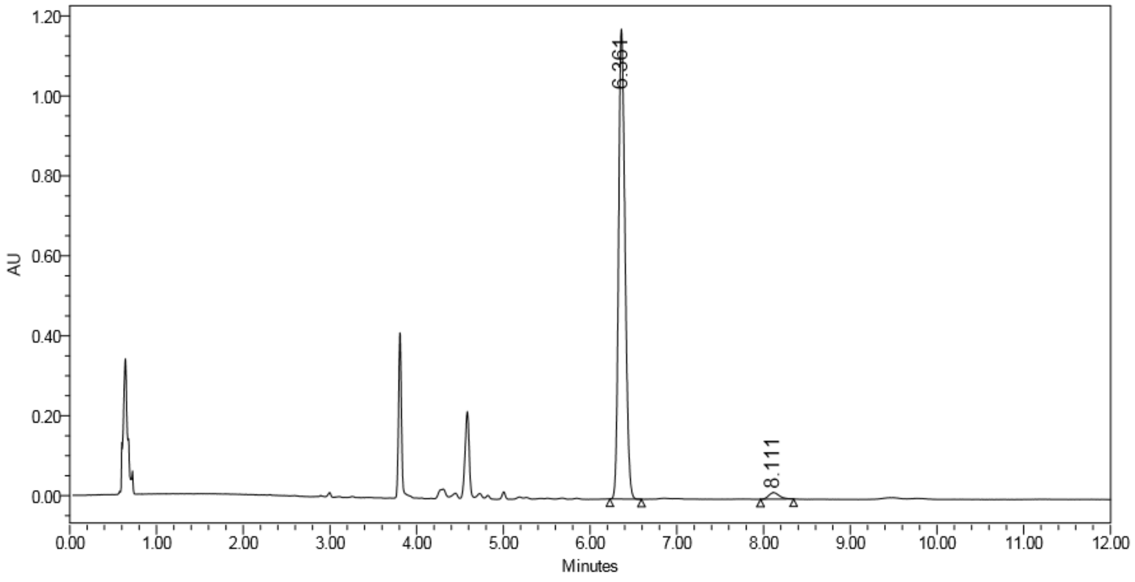

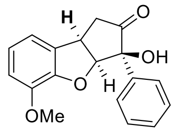

ent-3h

	Retention Time (min)	% Area
1	6.361	97.79
2	8.11'	2.21

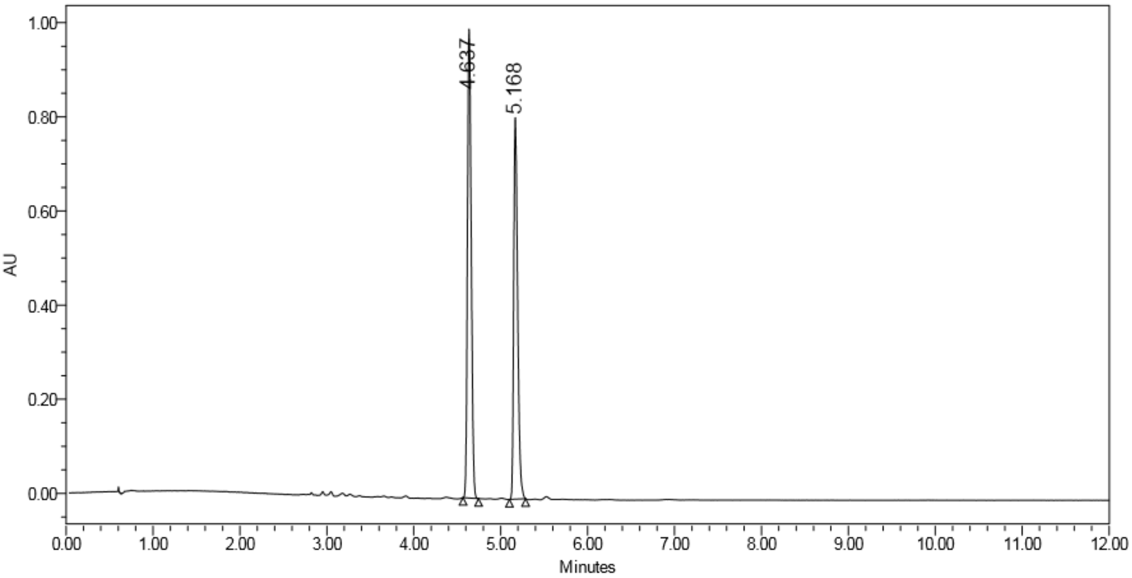

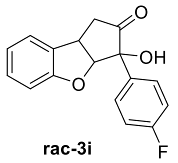

rac-3i

	Retention Time (min)	% Area
1	4.637	54.38
2	5.168	45.62

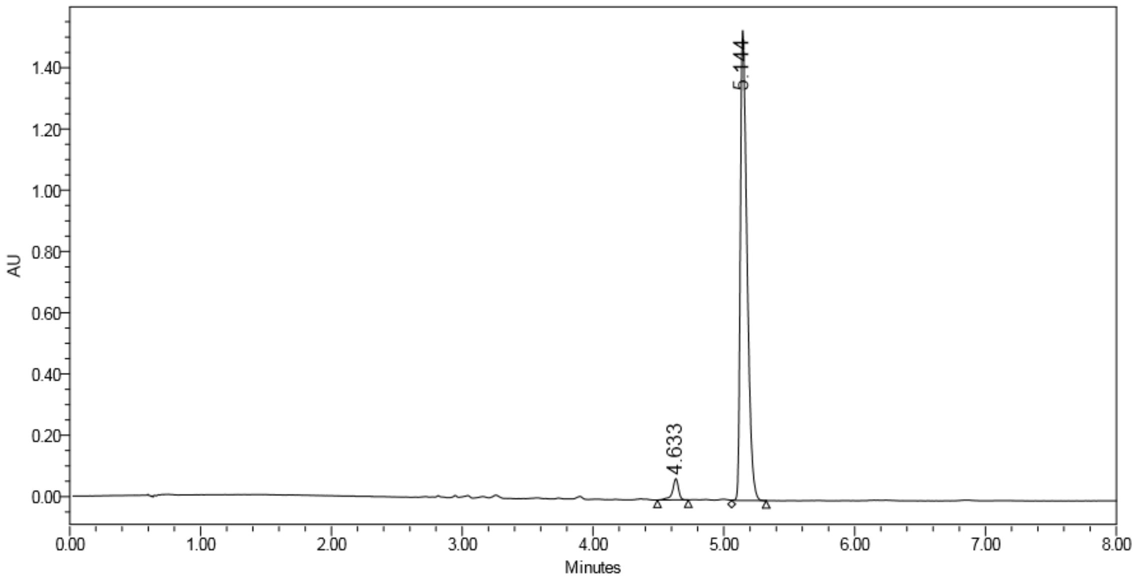

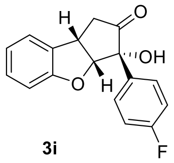

3i

	Retention Time (min)	% Area
1	4.633	3.88
2	5.144	96.12

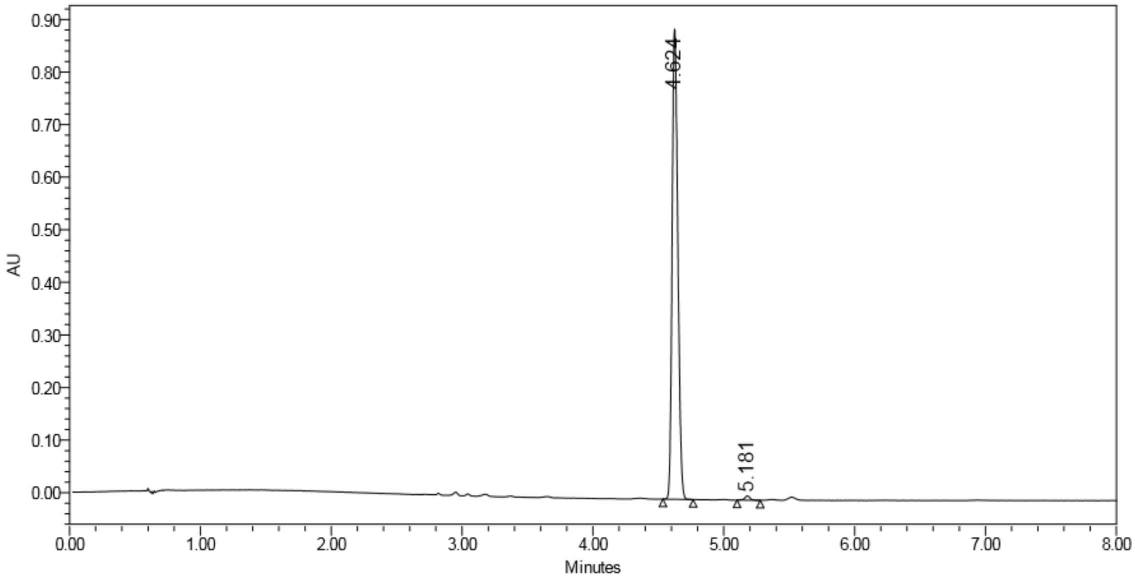

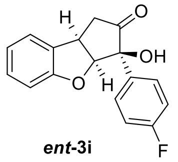

***ent*-3i**

	Retention Time (min)	% Area
1	4.624	99.18
2	5.181	0.82

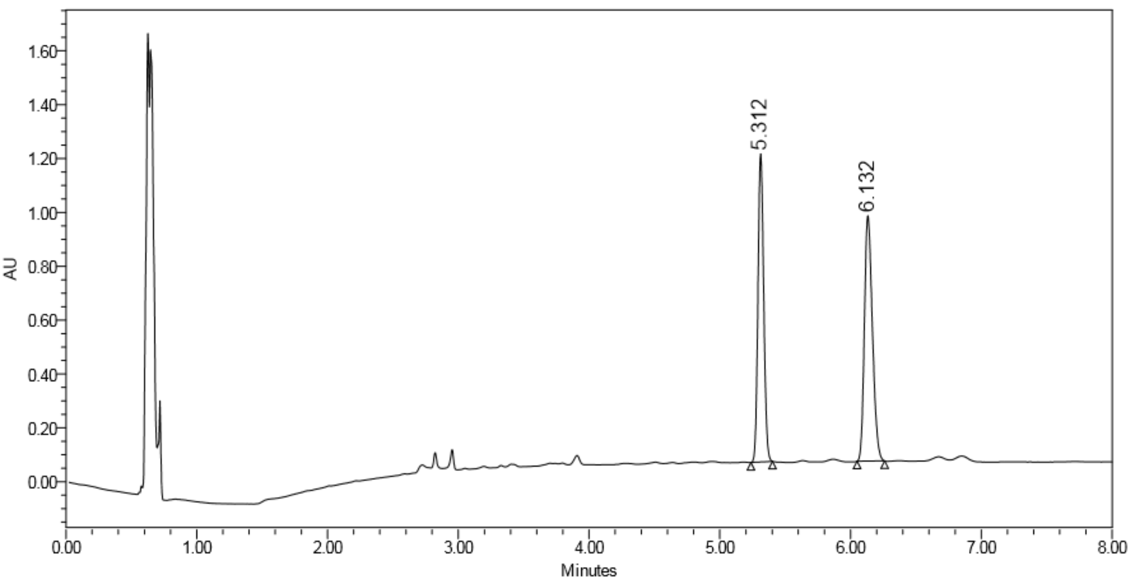

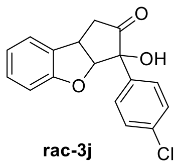

rac-3j

	Retention Time (min)	% Area
1	5.312	47.27
2	6.132	52.73

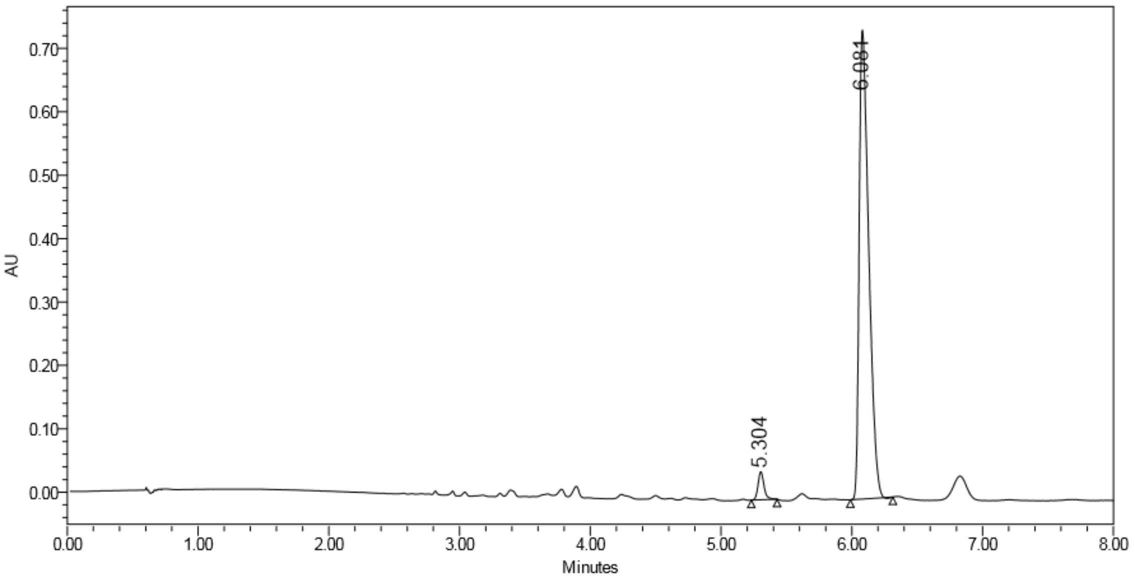

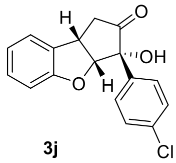

3j

	Retention Time (min)	% Area
1	5.304	3.61
2	6.081	96.39

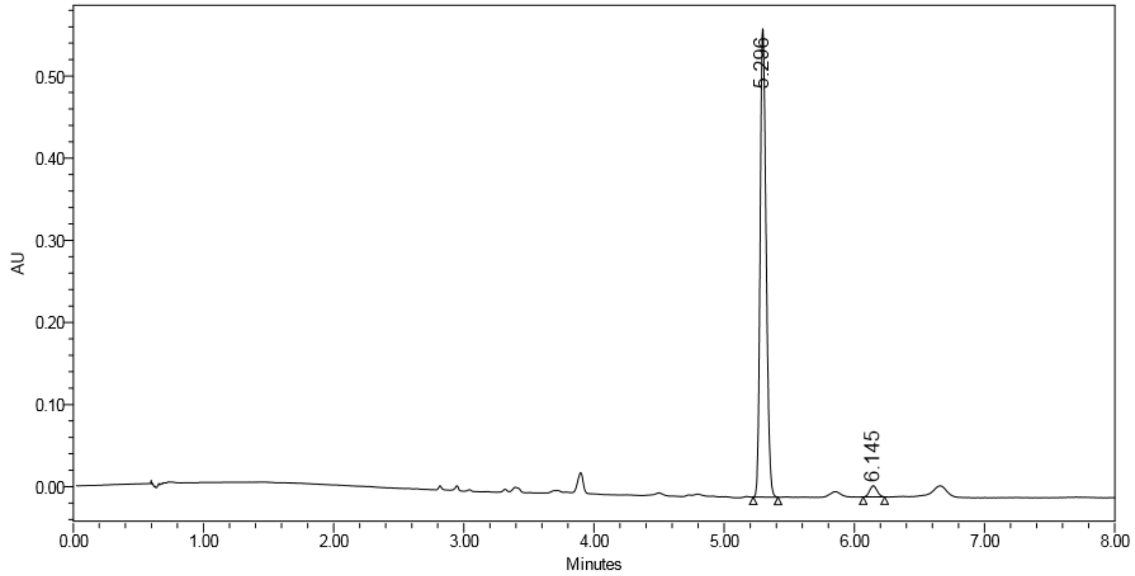

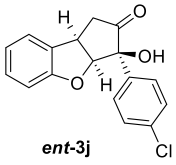

ent-3j

	Retention Time (min)	% Area
1	5.296	97.11
2	6.145	2.89

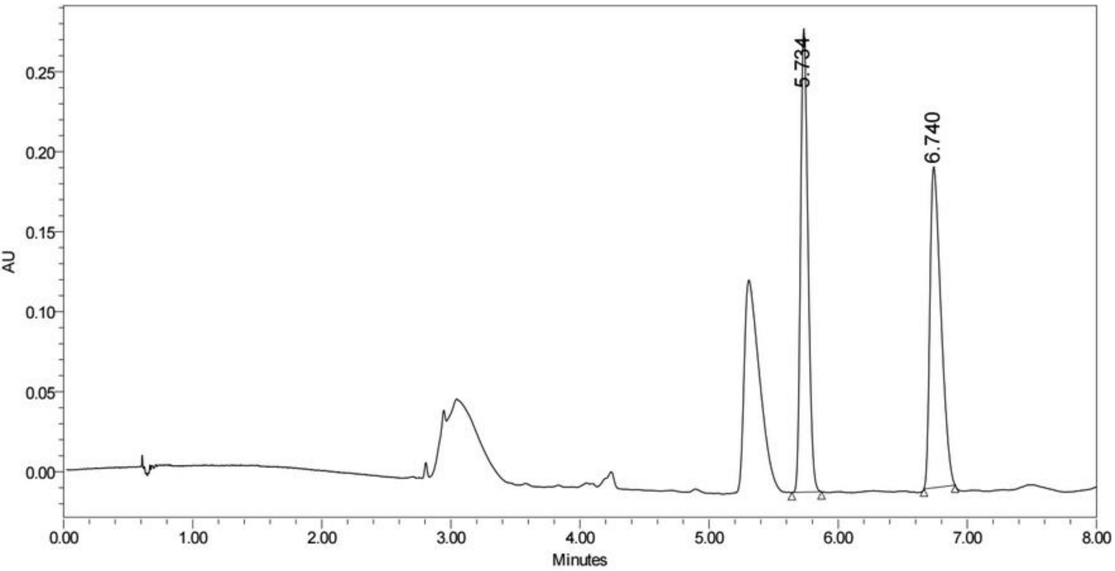

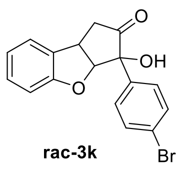

rac-3k

	Retention Time (min)	% Area
1	5.734	49.36
2	6.740	50.64

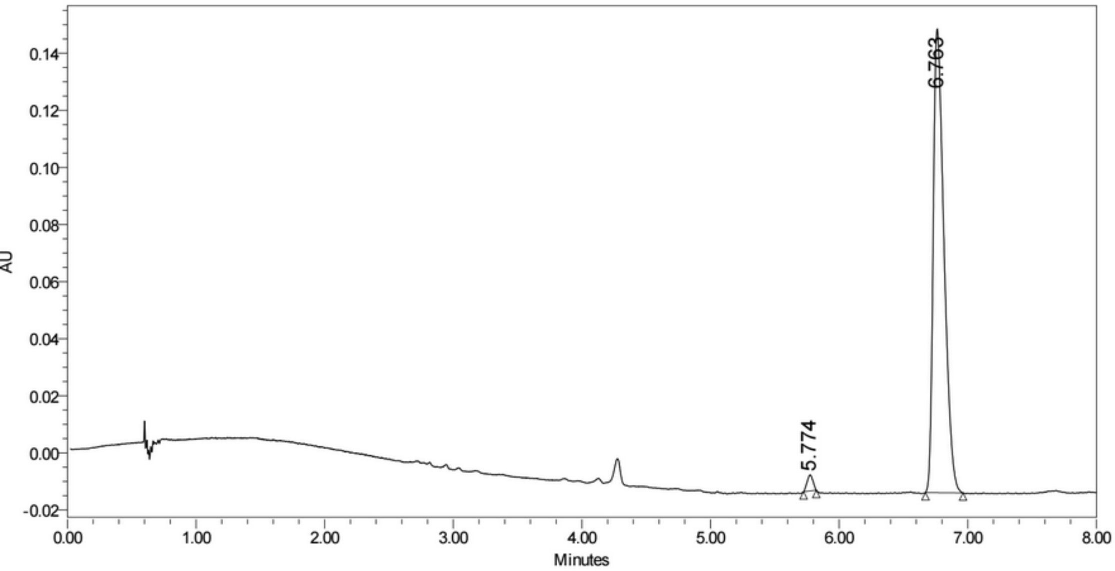

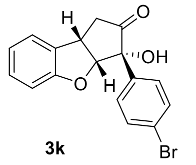

3k

	Retention Time (min)	% Area
1	5.774	1.83
2	6.763	98.17

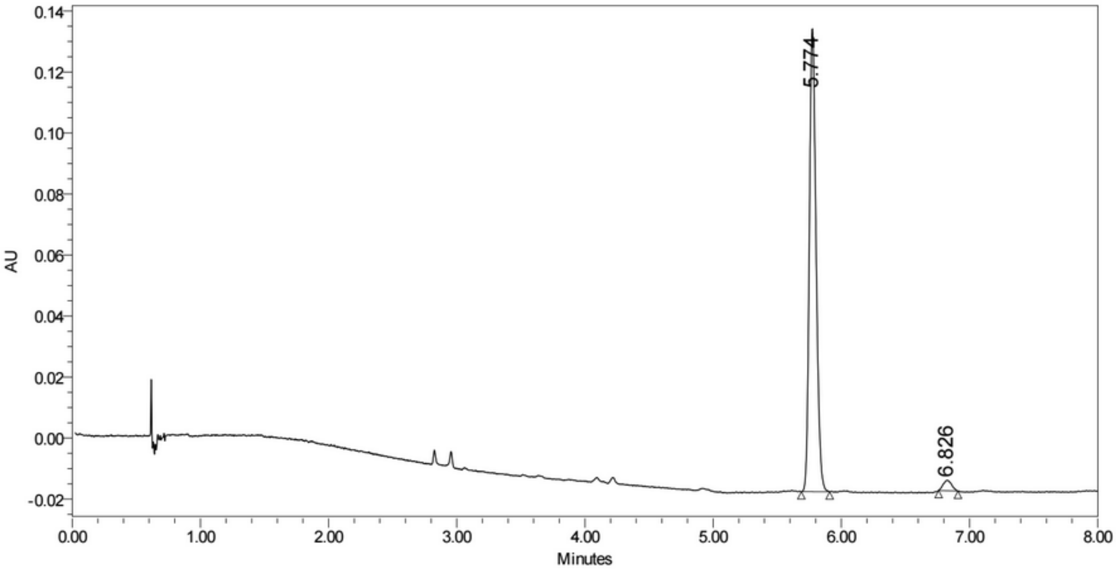

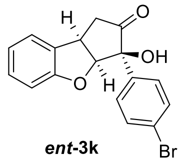

ent-3k

	Retention Time (min)	% Area
1	5.774	97.26
2	6.826	2.74

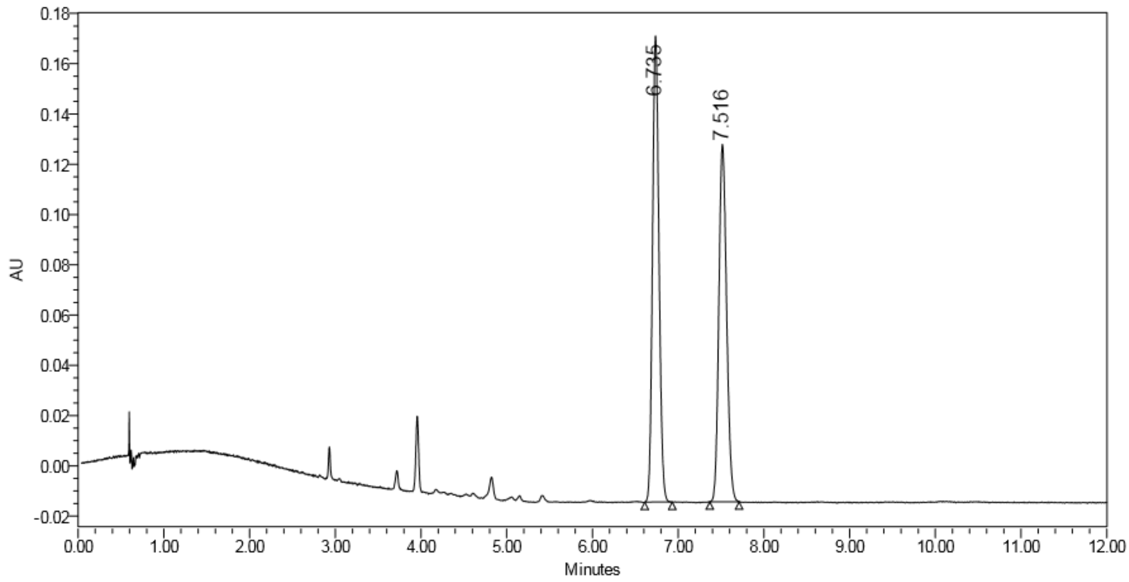

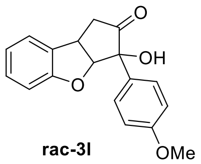

rac-3l

	Retention Time (min)	% Area
1	6.735	51.97
2	7.516	48.03

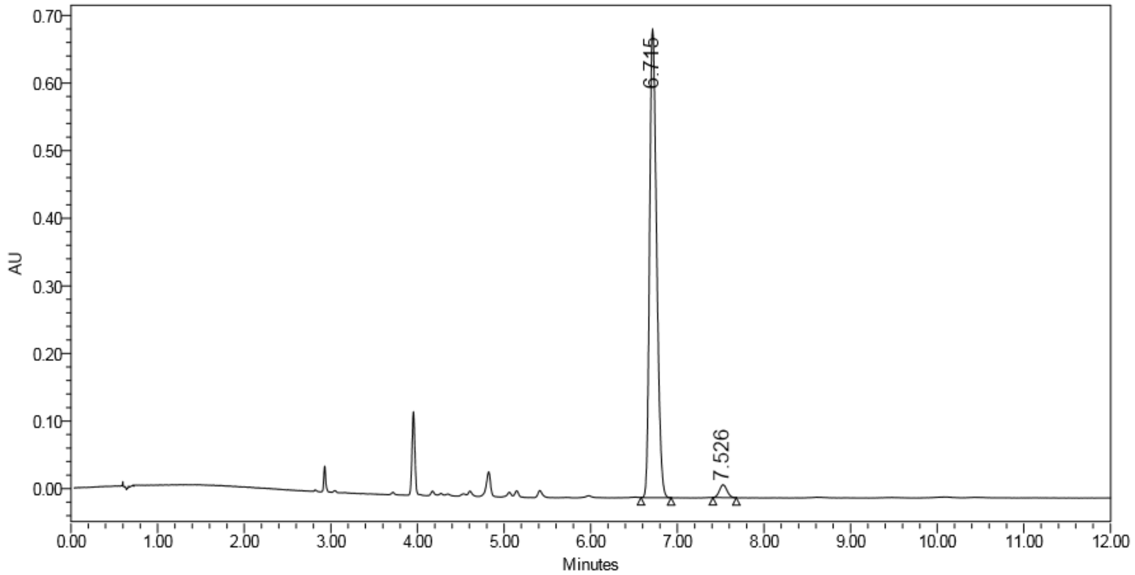

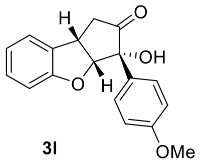

3l

	Retention Time (min)	% Area
1	6.742	2.92
2	7.493	97.08

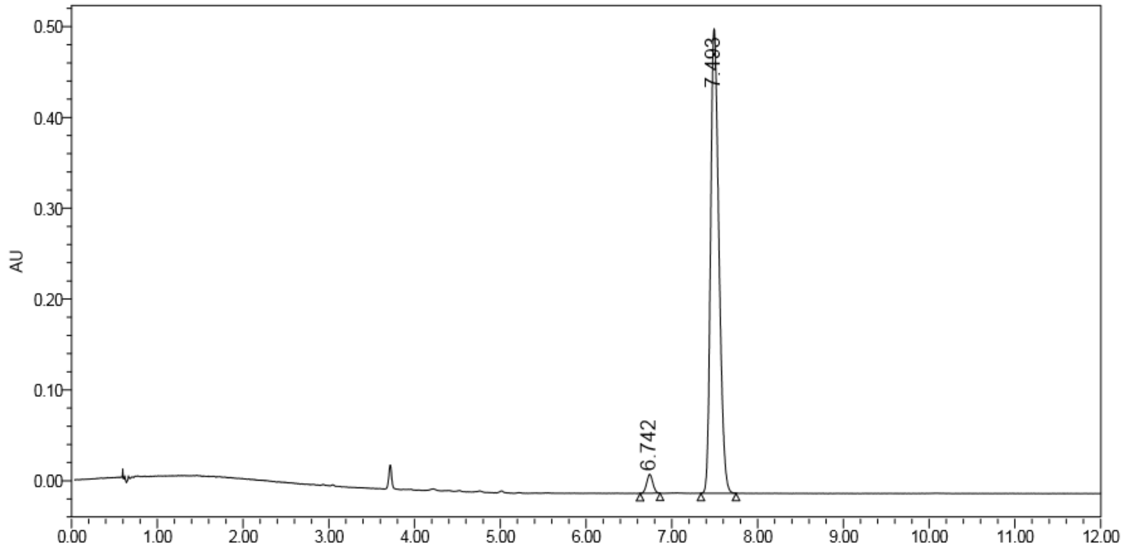

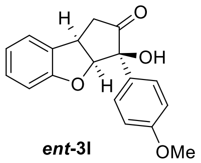

ent-3l

	Retention Time (min)	% Area
1	6.715	97.12
2	7.526	2.88

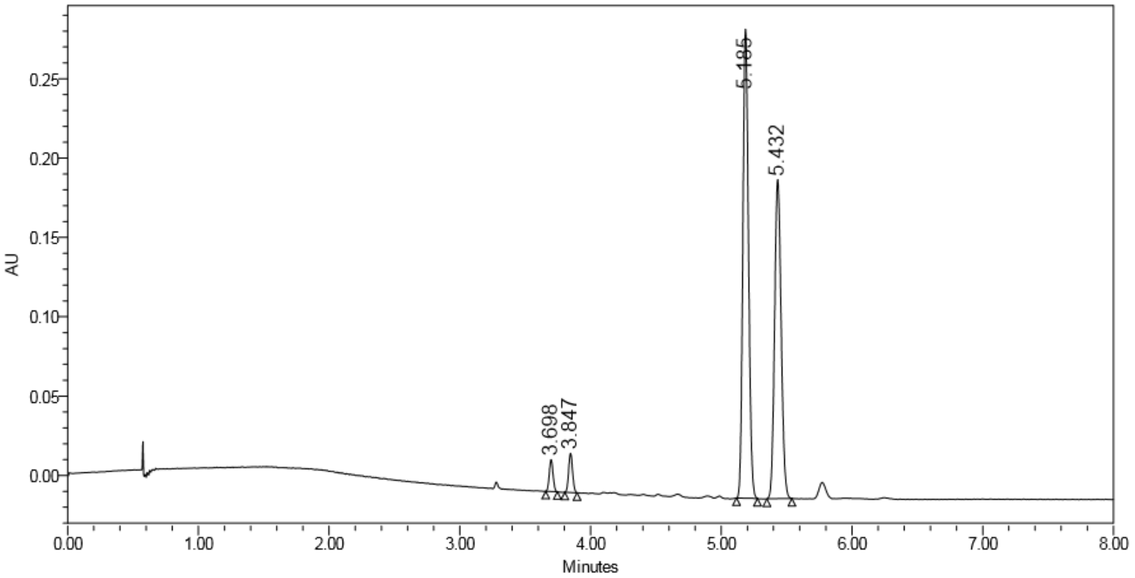

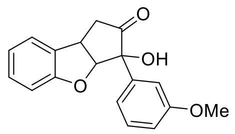

rac-3m

	Retention Time (min)	% Area
1	3.698	2.39
2	3.847	3.08
3	5.185	52.56
4	5.432	41.97

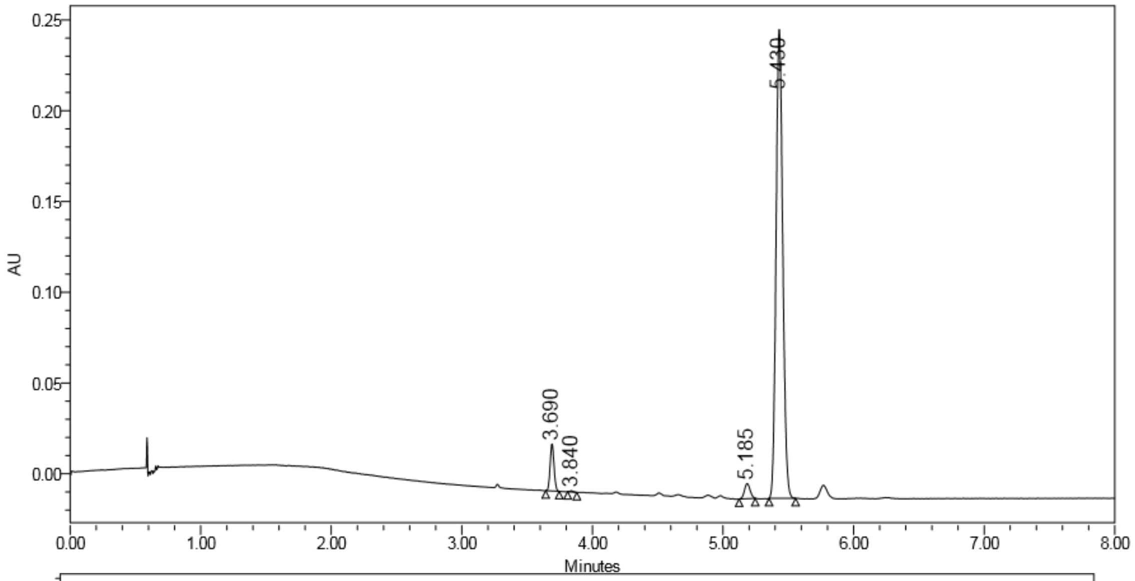

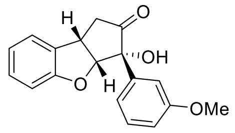

3m

	Retention Time (min)	% Area
1	3.690	5.35
2	3.840	0.12
3	5.185	2.53
4	5.430	91.99

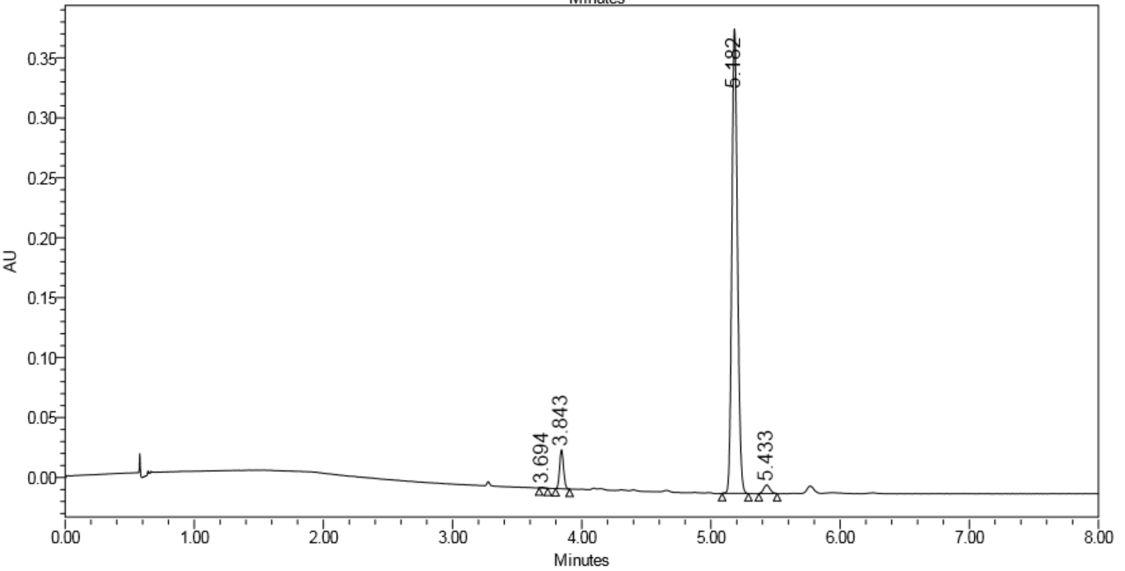

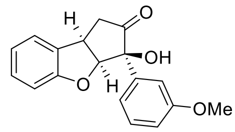

***ent*-3m**

	Retention Time (min)	% Area
1	3.694	0.10
2	3.843	5.45
3	5.182	92.54
4	5.433	1.91

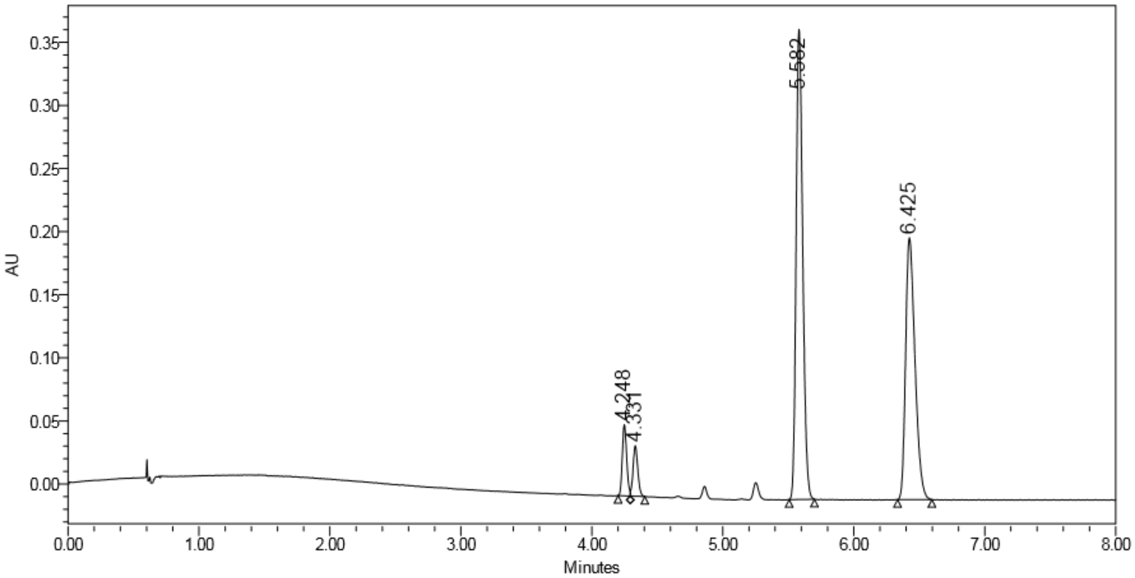

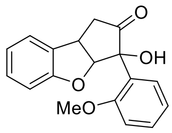

rac-3n

	Retention Time (min)	% Area
1	4.248	5.09
2	4.331	3.92
3	5.582	50.80
4	6.425	40.18

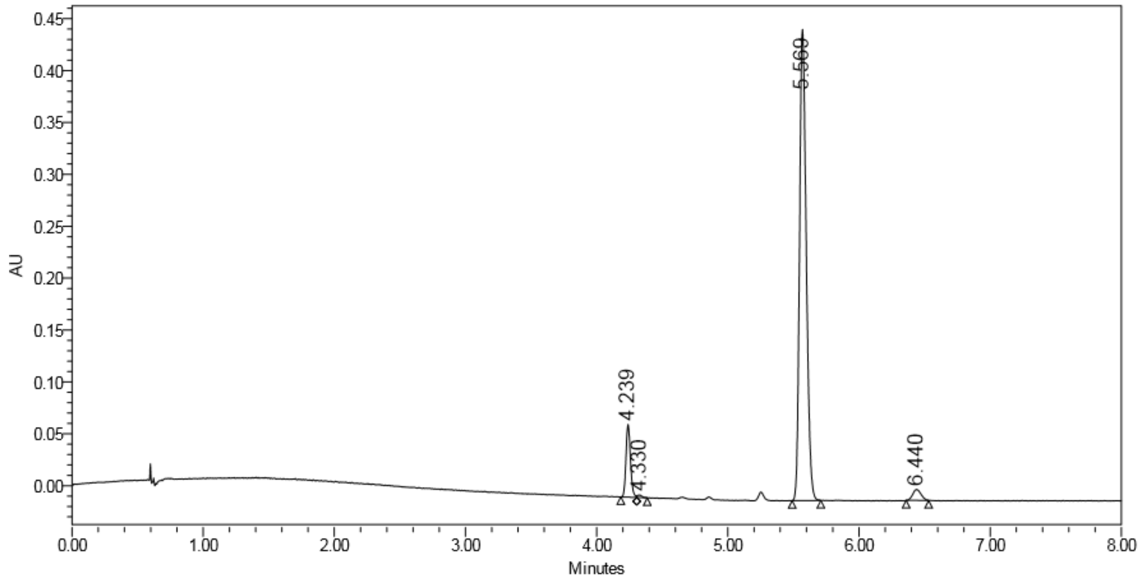

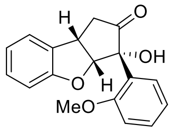

3n

	Retention Time (min)	% Area
1	4.239	9.05
2	4.330	0.33
3	5.569	88.07
4	6.440	2.54

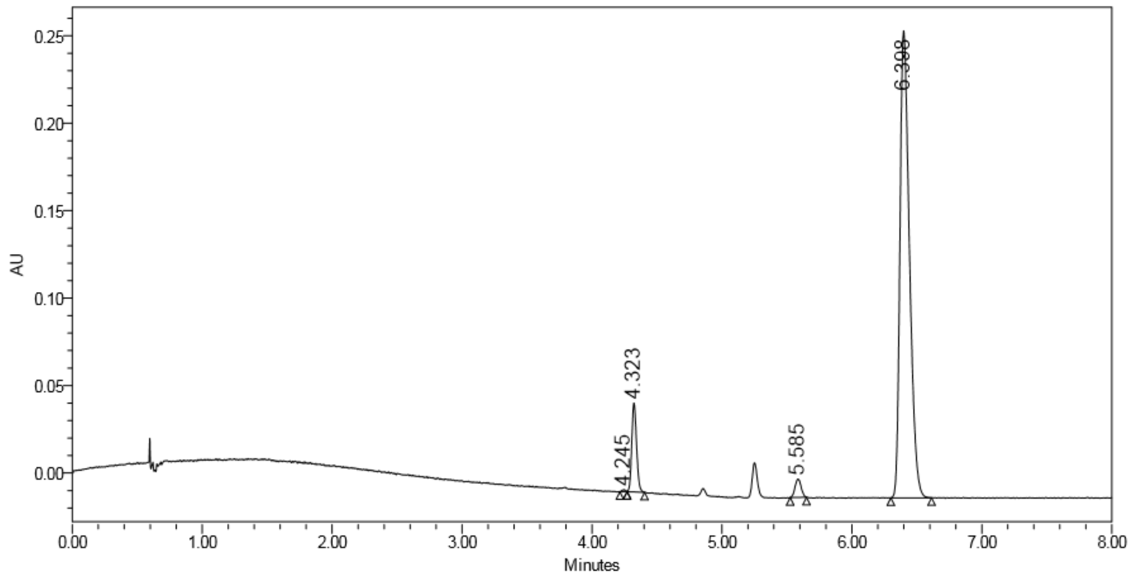

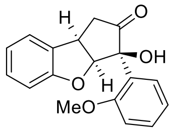

***ent*-3n**

	Retention Time (min)	% Area
1	4.245	0.15
2	4.323	8.56
3	5.585	2.27
4	6.398	89.01

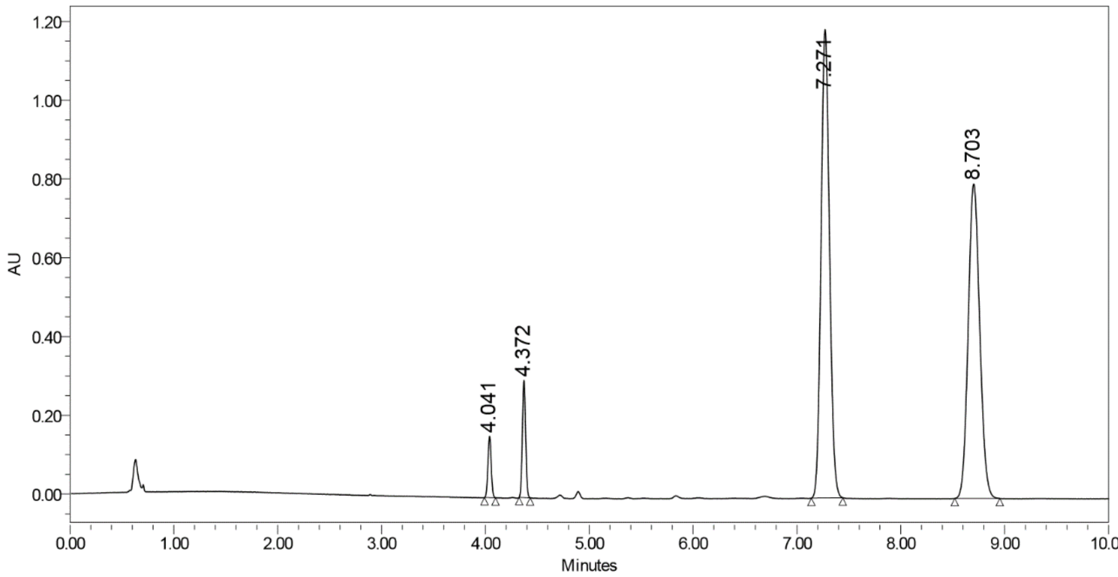

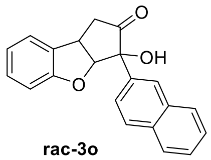

rac-3o

	Retention Time (min)	% Area
1	4.041	2.44
2	4.372	4.69
3	7.271	48.54
4	8.703	44.33

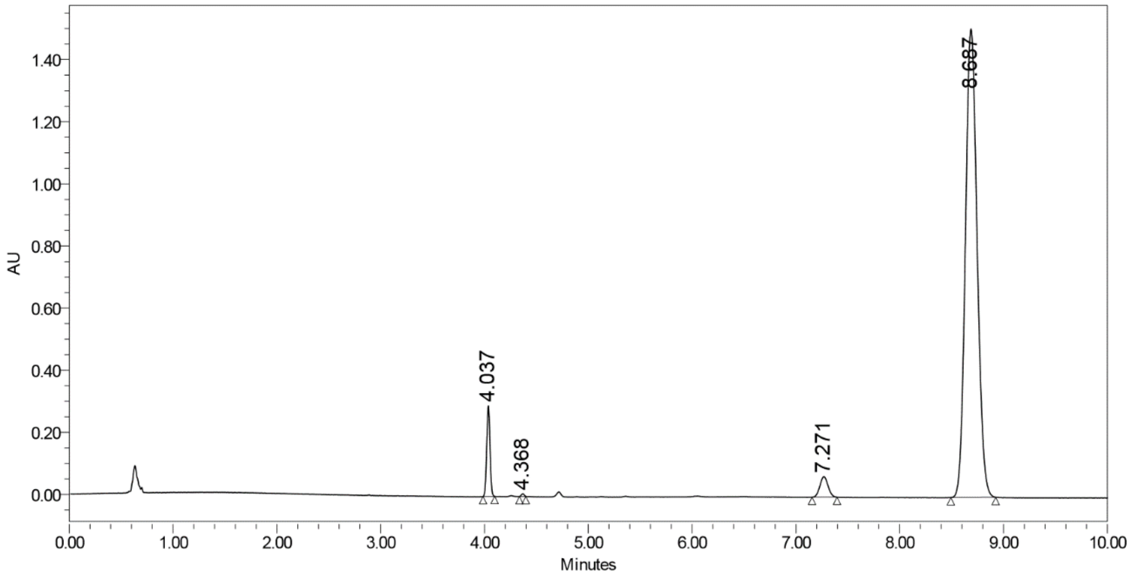

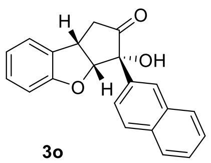

3o

	Retention Time (min)	% Area
1	4.037	5.00
2	4.368	0.13
3	7.271	2.83
4	8.687	92.04

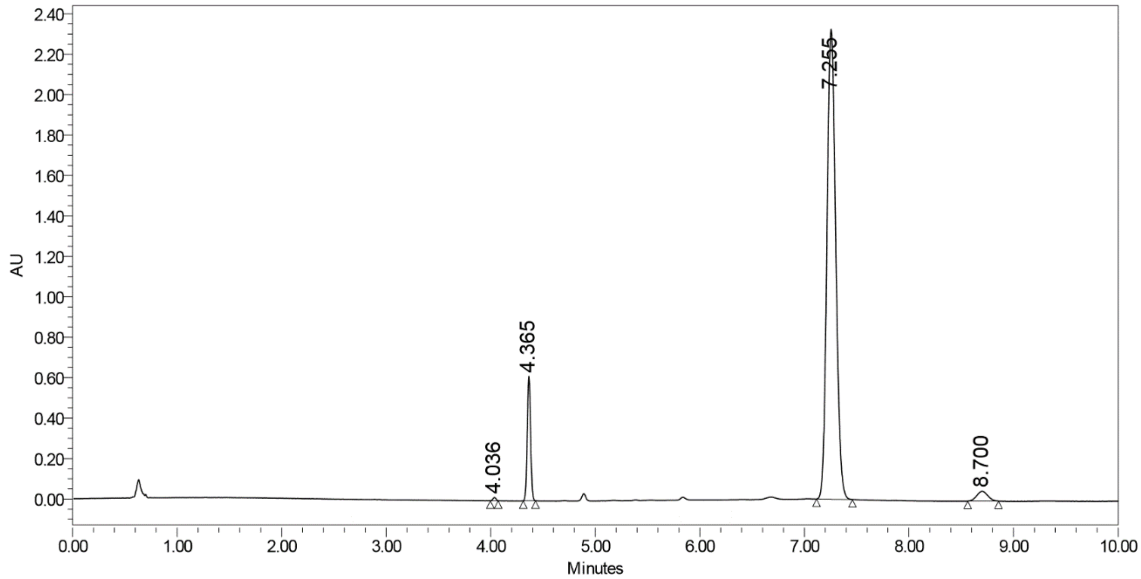

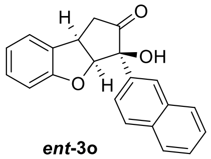

***ent*-3o**

	Retention Time (min)	% Area
1	4.036	0.22
2	4.365	8.89
3	7.255	88.58
4	8.700	2.31

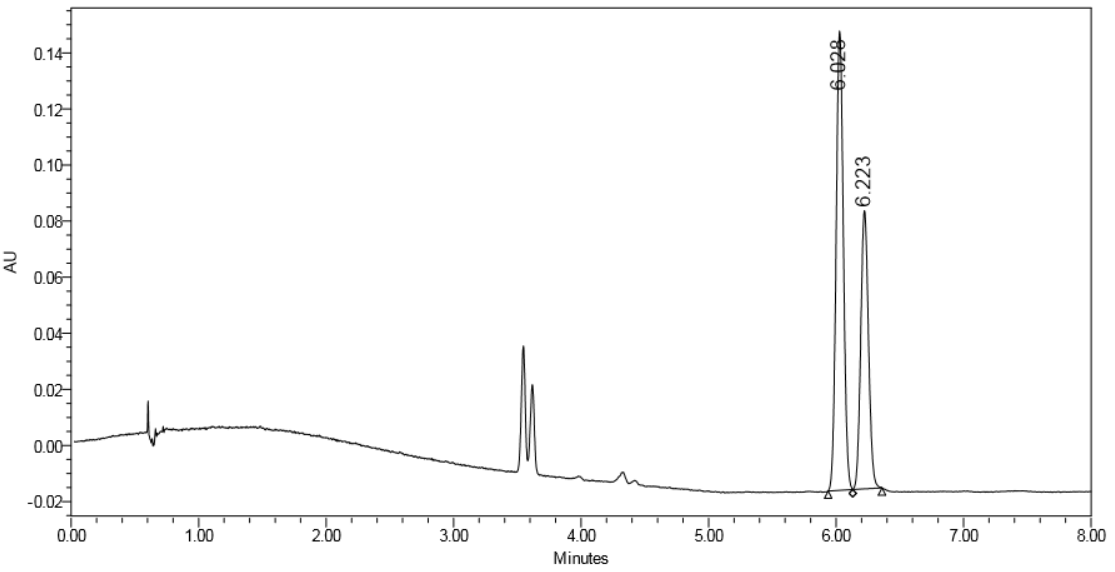

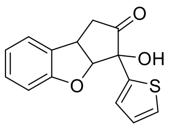

rac-3p

	Retention Time (min)	% Area
1	6.028	60.73
2	6.223	39.27

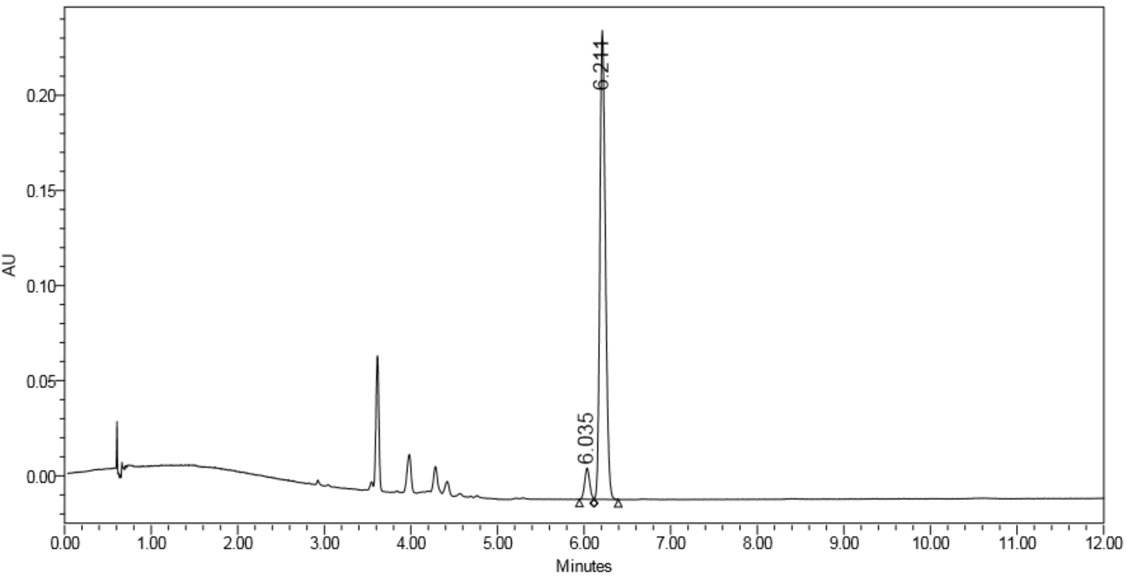

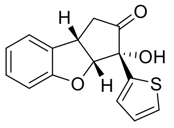

3p

	Retention Time (min)	% Area
1	6.035	5.64
2	6.211	94.36

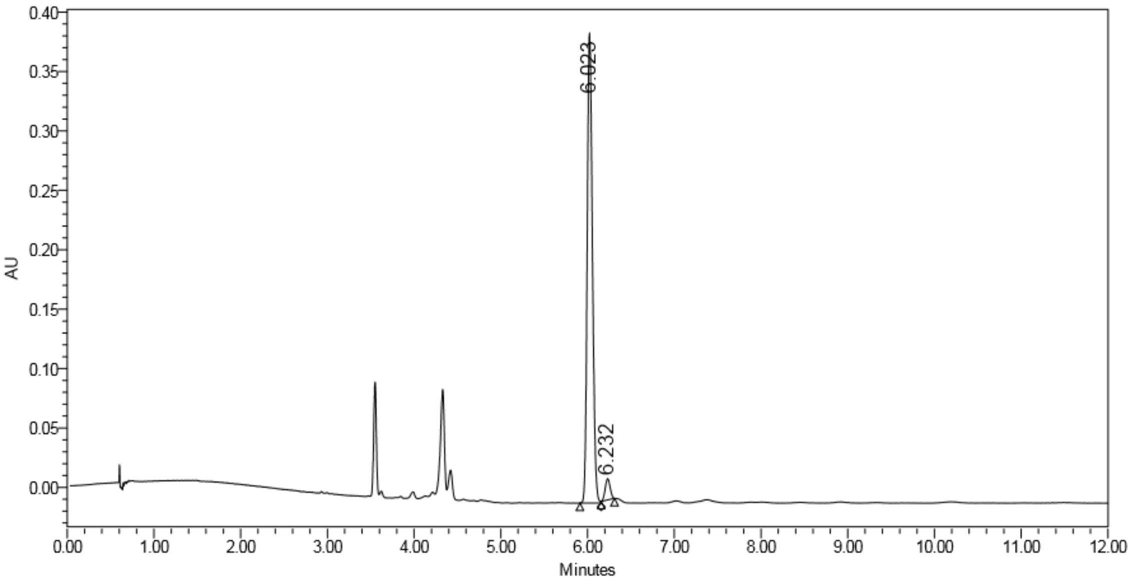

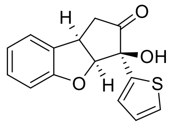

***ent*-3p**

	Retention Time (min)	% Area
1	6.023	95.92
2	6.232	4.08

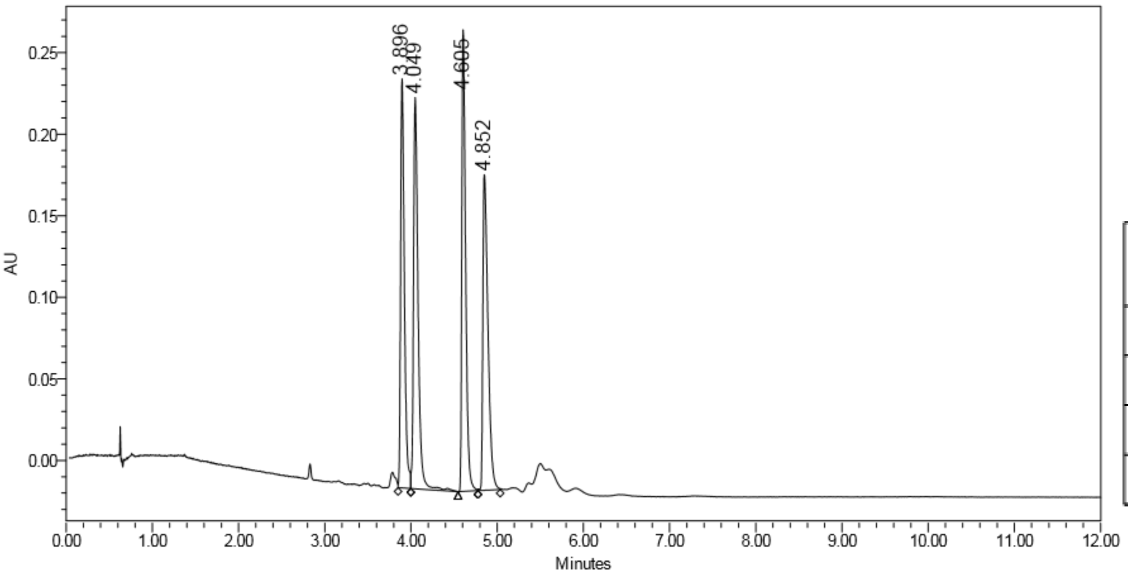

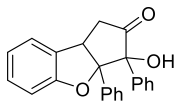

rac-3q

	Retention Time (min)	% Area
1	3.896	23.01
2	4.049	26.62
3	4.605	26.24
4	4.852	24.13

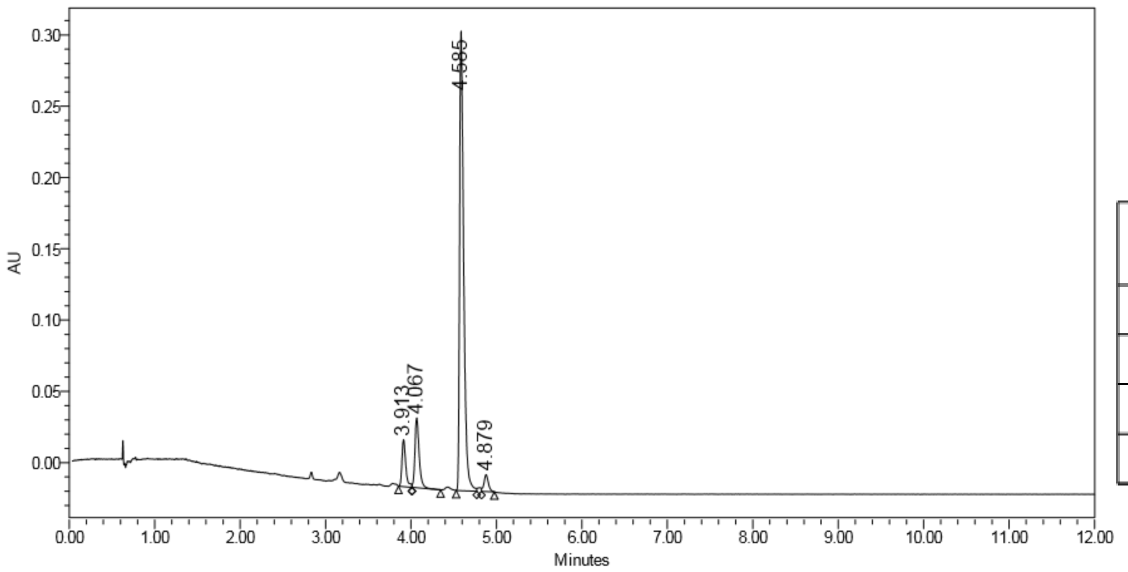

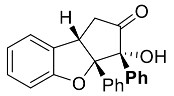

3q

	Retention Time (min)	% Area
1	3.913	7.38
2	4.067	12.23
3	4.585	77.36
4	4.879	3.03

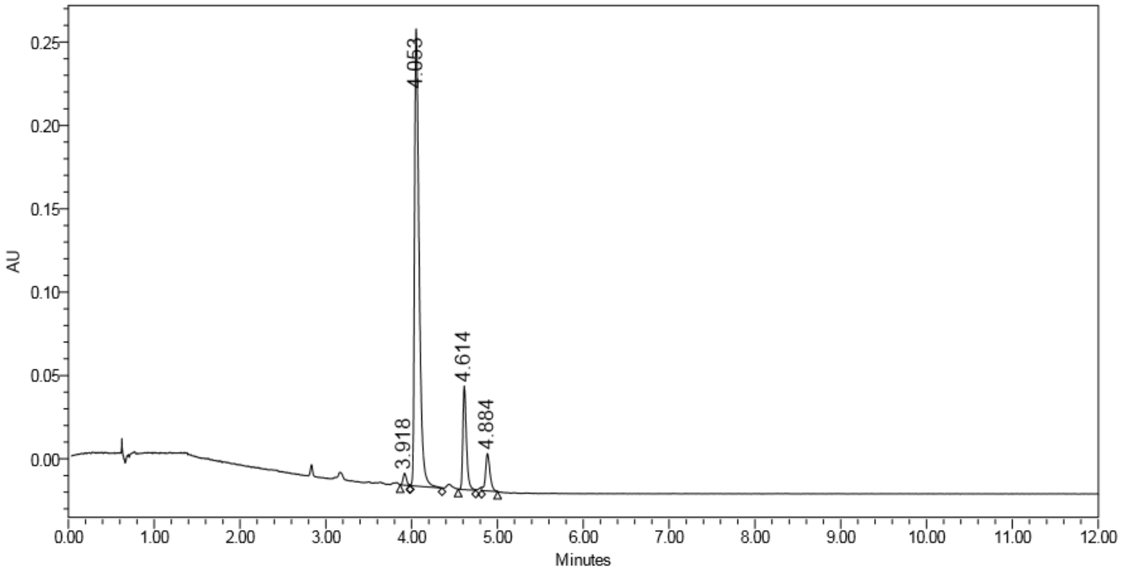

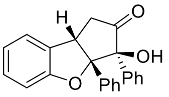

***epi*-3q**

	Retention Time (min)	% Area
1	3.918	1.51
2	4.053	78.54
3	4.614	13.76
4	4.884	6.19

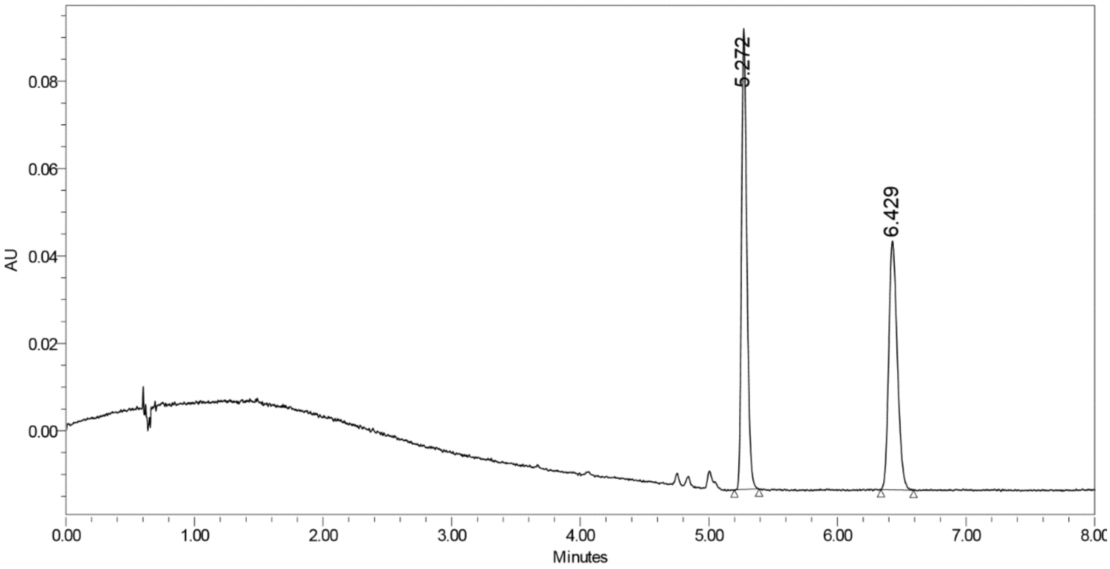

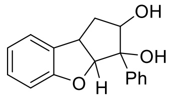

rac-6

	Retention Time (min)	% Area
1	5.272	53.45
2	6.429	46.55

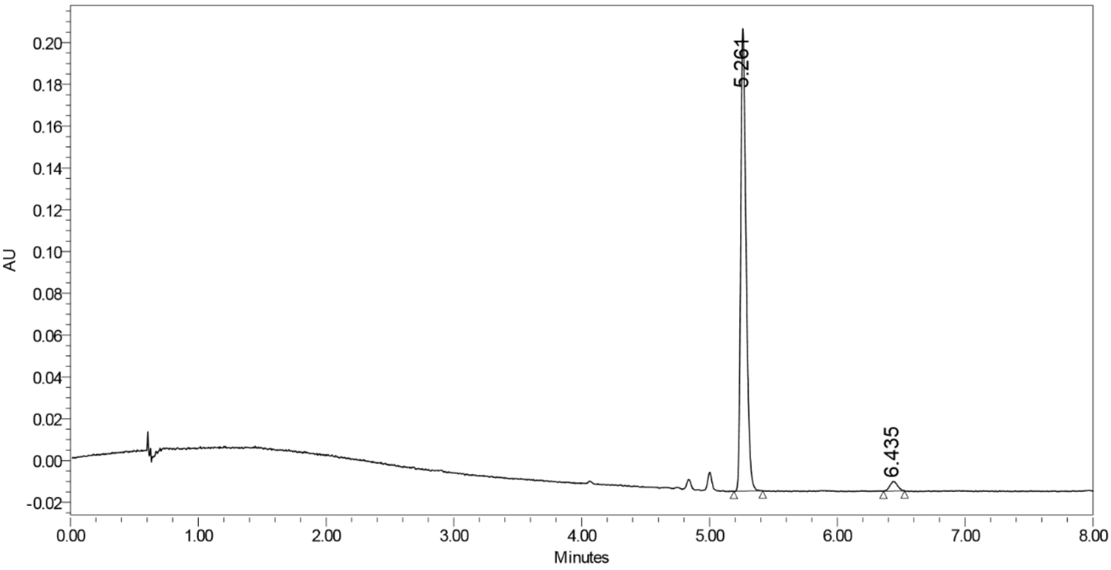

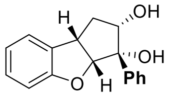

6

	Retention Time (min)	% Area
1	5.261	97.09
2	6.435	2.91

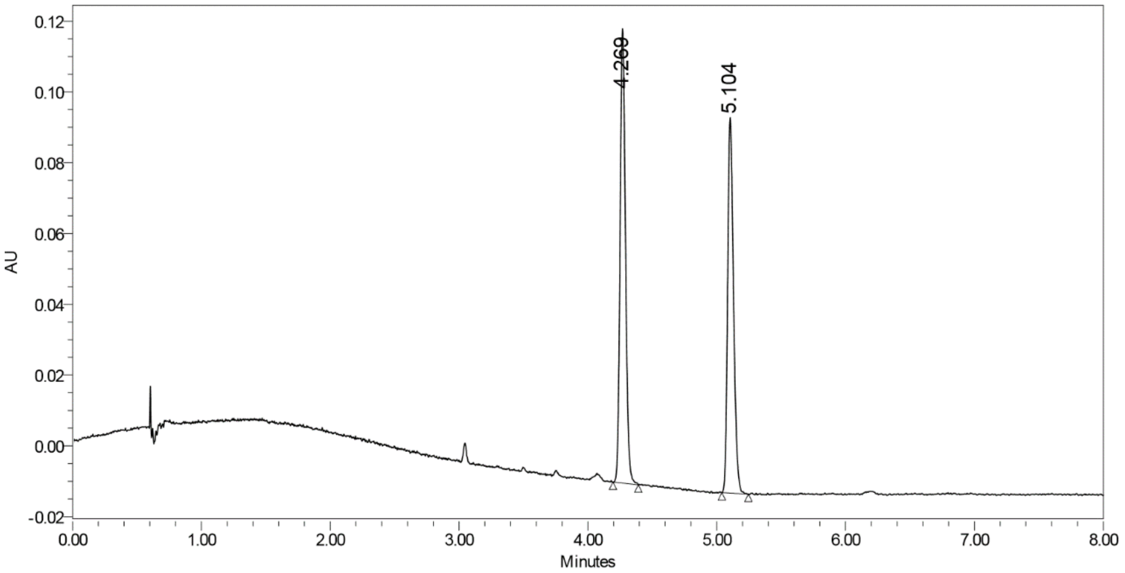

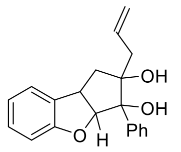

rac-7

	Retention Time (min)	% Area
1	4.269	52.69
2	5.104	47.31

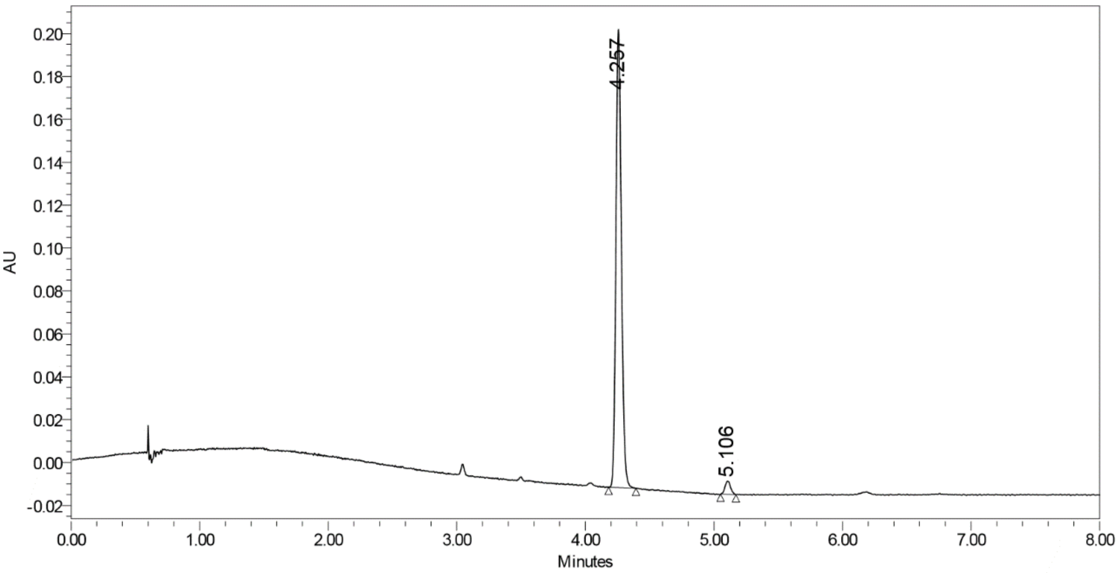

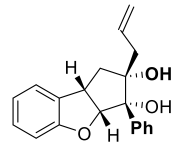

7

	Retention Time (min)	% Area
1	4.257	97.15
2	5.106	2.85

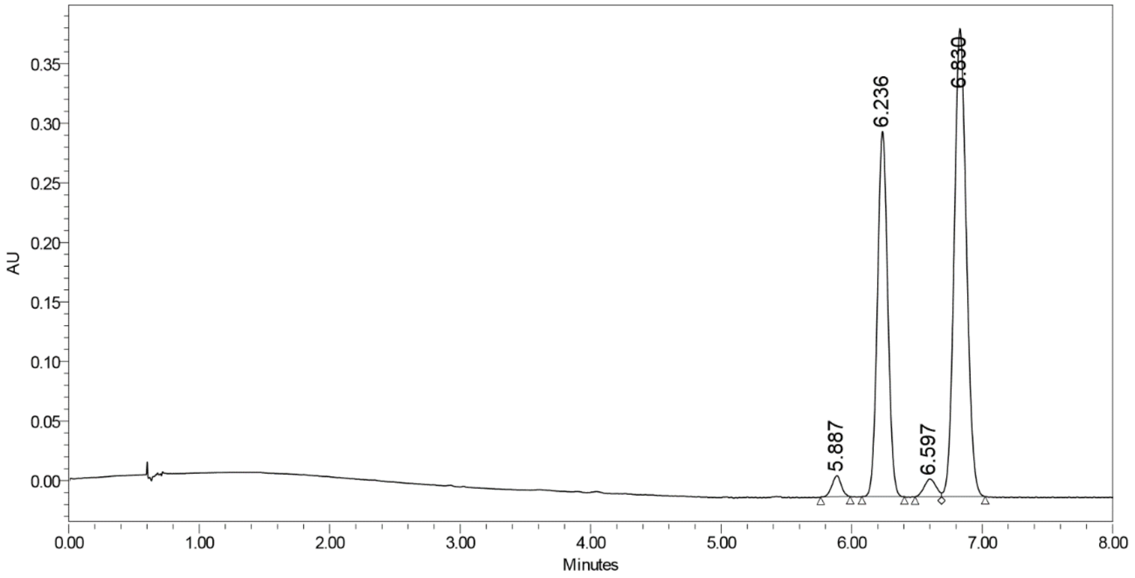

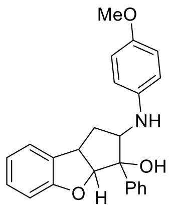

rac-8

	Retention Time (min)	% Area
1	5.887	1.99
2	6.236	38.02
3	6.597	2.14
4	6.830	57.85

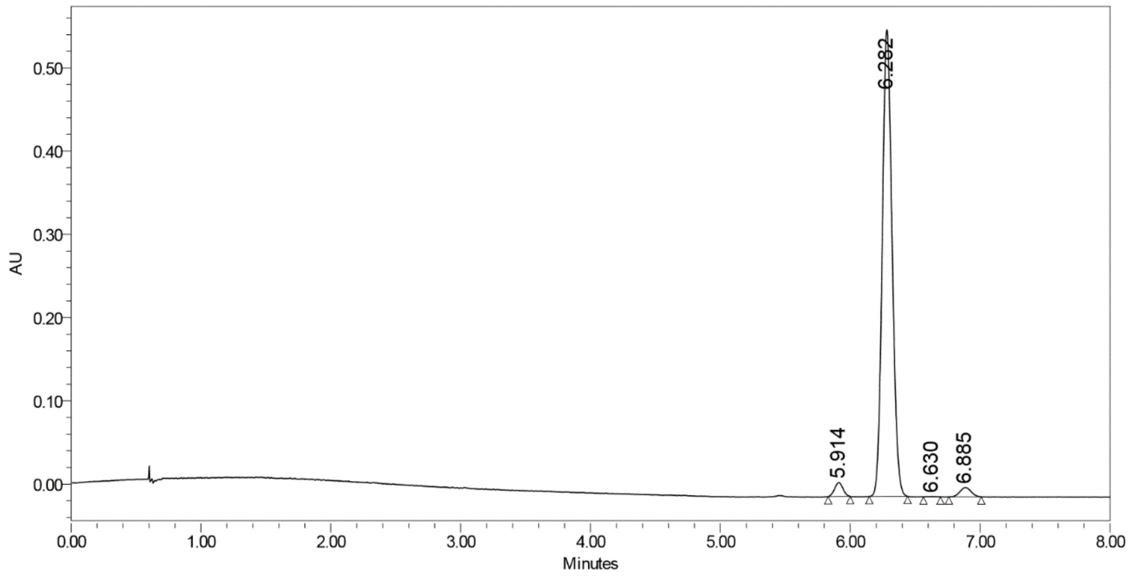

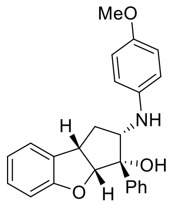

8

	Retention Time (min)	% Area
1	5.914	2.40
2	6.282	95.37
3	6.630	0.05
4	6.885	2.18

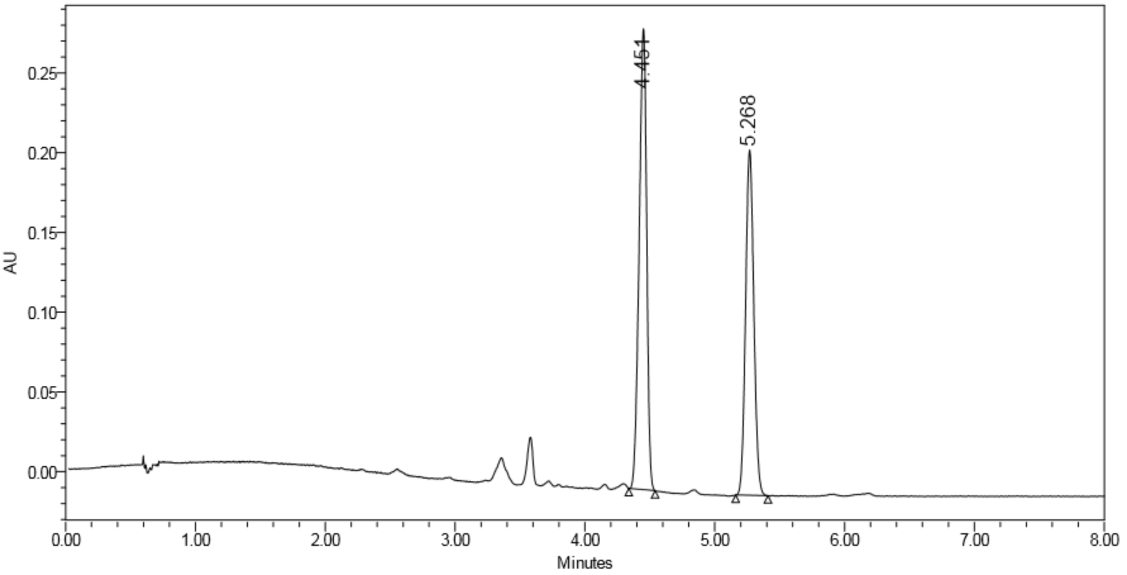

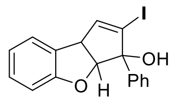

rac-9

	Retention Time (min)	% Area
1	4.451	54.94
2	5.268	45.06

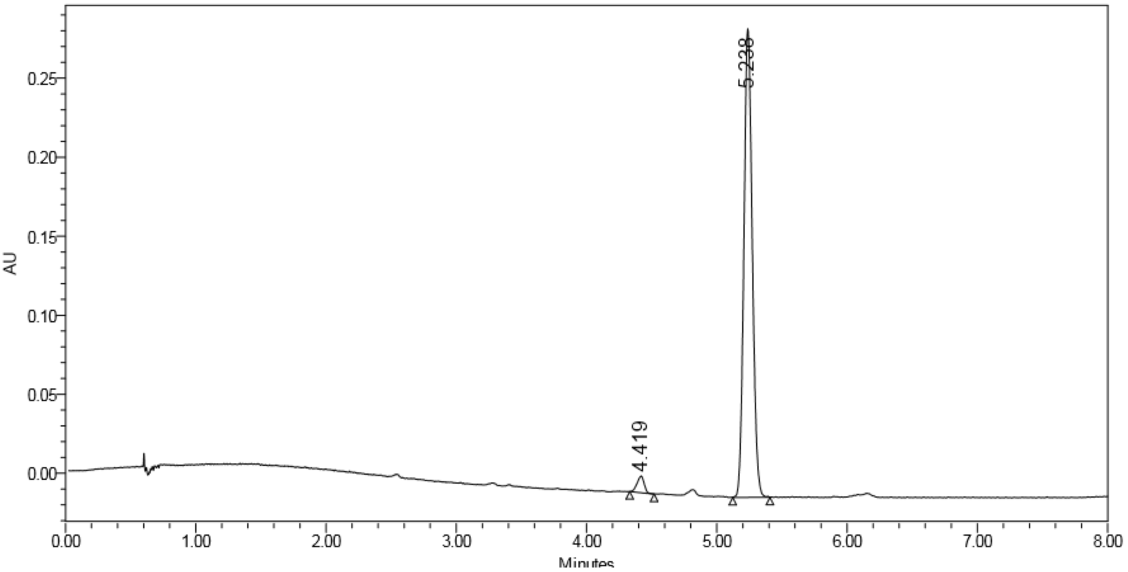

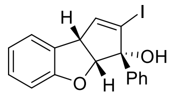

9

	Retention Time (min)	% Area
1	4.419	2.92
2	5.238	97.08

10. IR Spectra

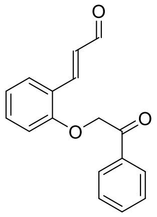

1a

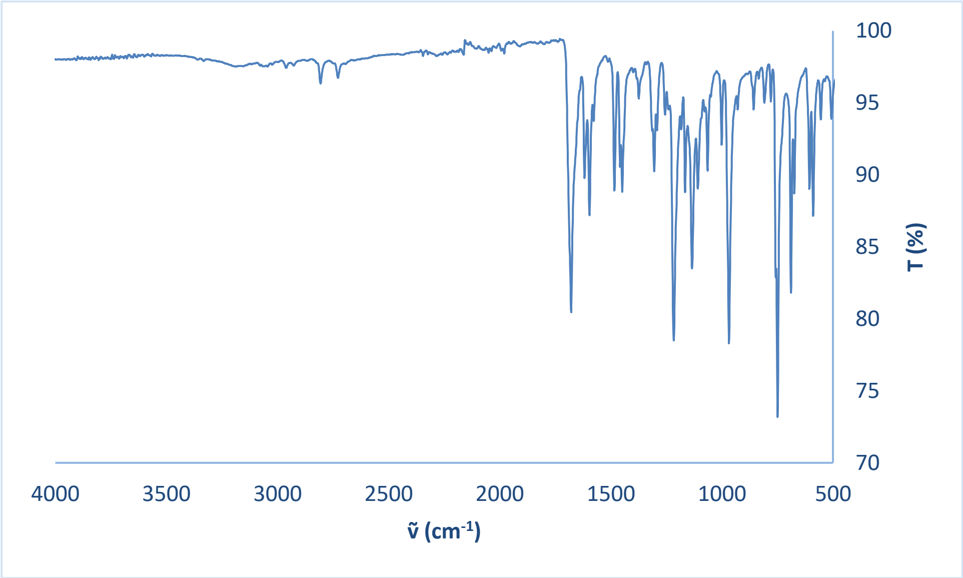

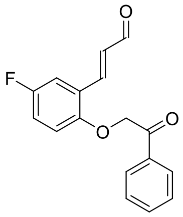

1b

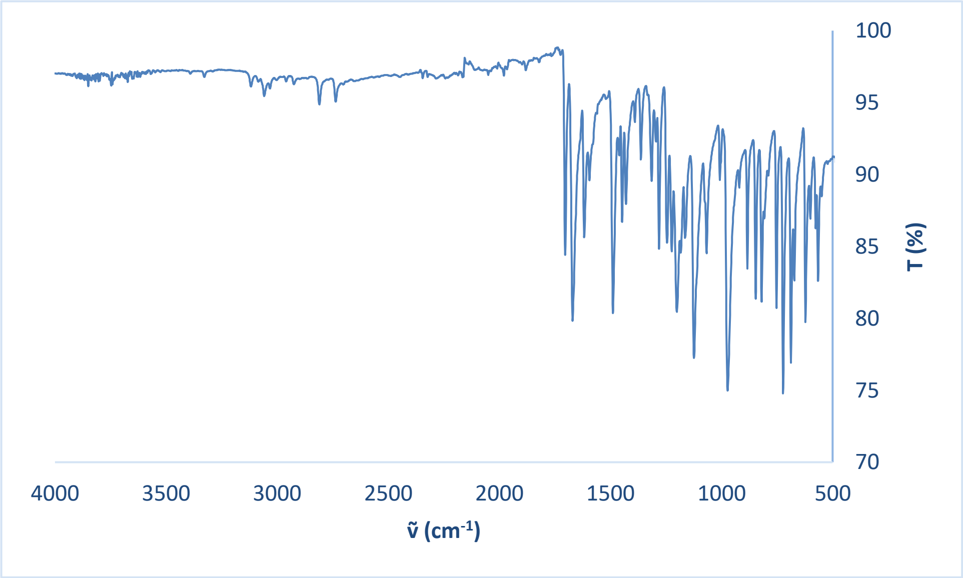

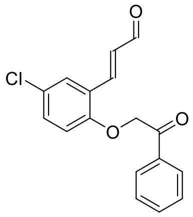

O
Cl
O
O

1c

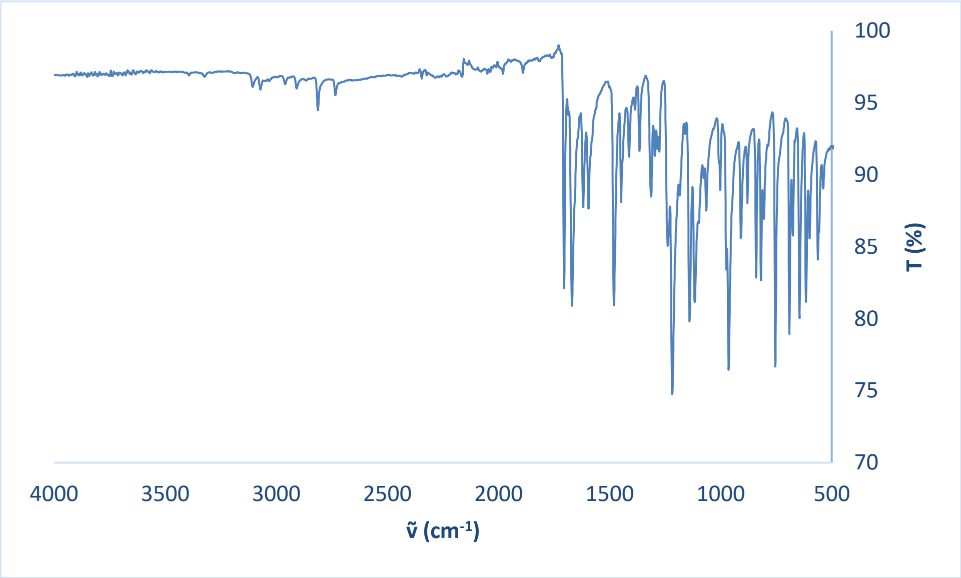

100
95
90
85
80
75
70
T (%)
4000
3500
3000
2500
2000
1500
1000
500
ṽ (cm-1)

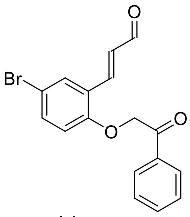

O
Br
O
O

1d

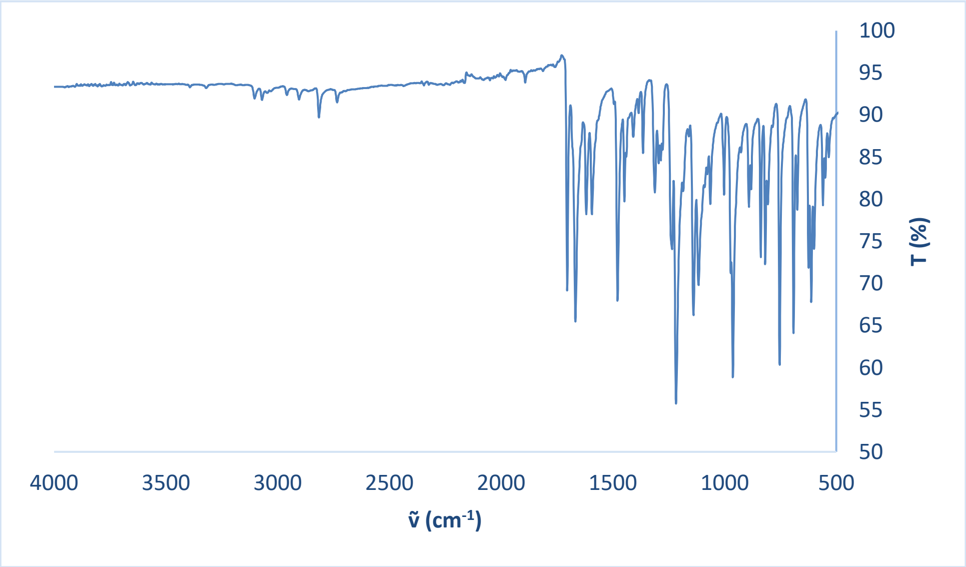

100
95
90
85
80
75
70
65
60
55
50
T (%)
4000
3500
3000
2500
2000
1500
1000
500
ṽ (cm-1)

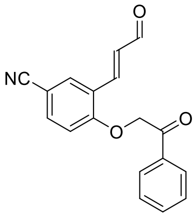
NC
O
O
O

1e

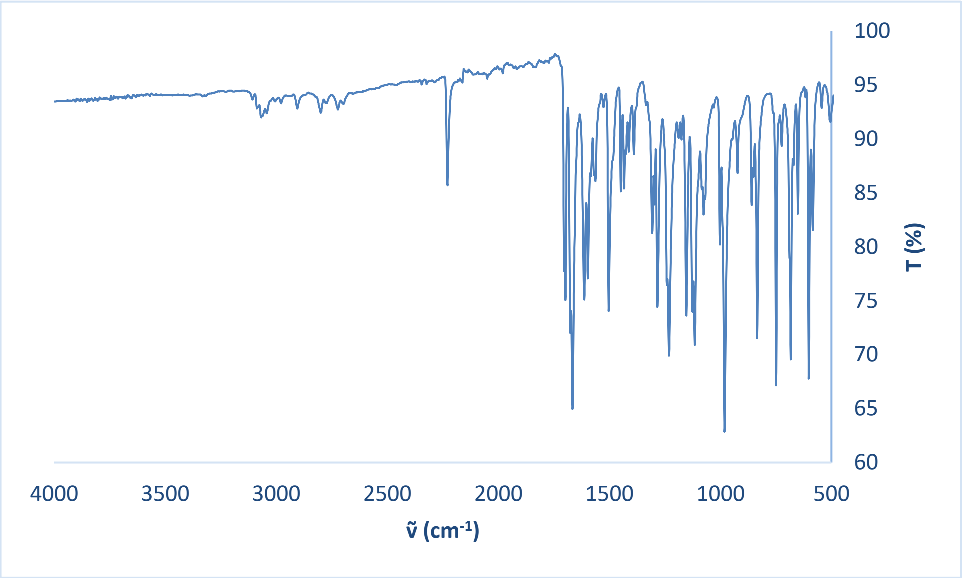
100
95
90
85
80
75
70
65
60
T (%)
4000
3500
3000
2500
2000
1500
1000
500
ṽ (cm-1)

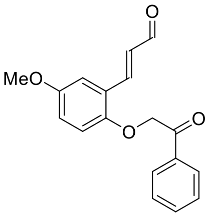
MeO
O
O
O

1f

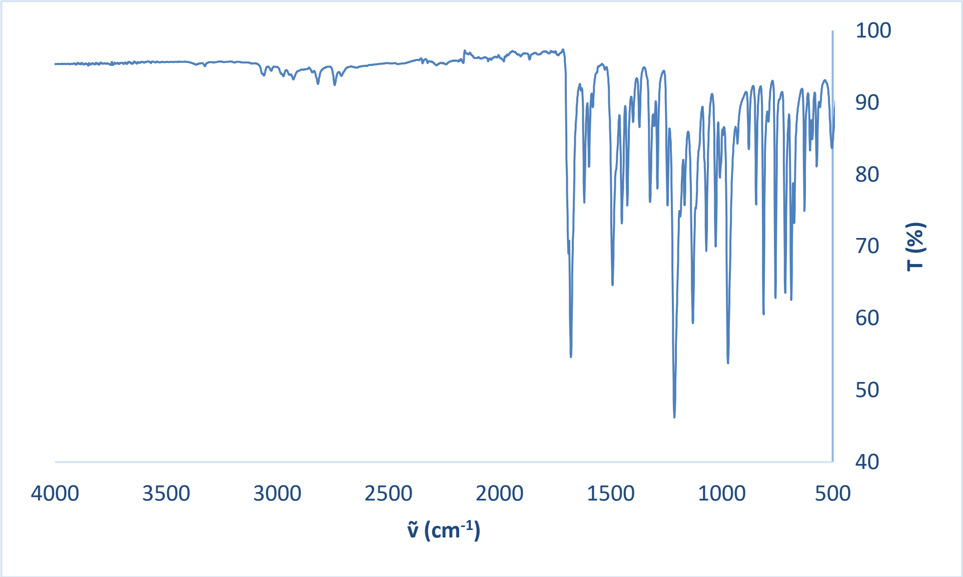
100
90
80
70
60
50
40
T (%)
4000
3500
3000
2500
2000
1500
1000
500
ṽ (cm-1)

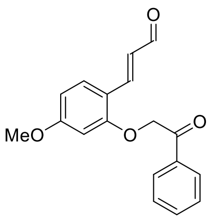
O
MeO
O
O

1g

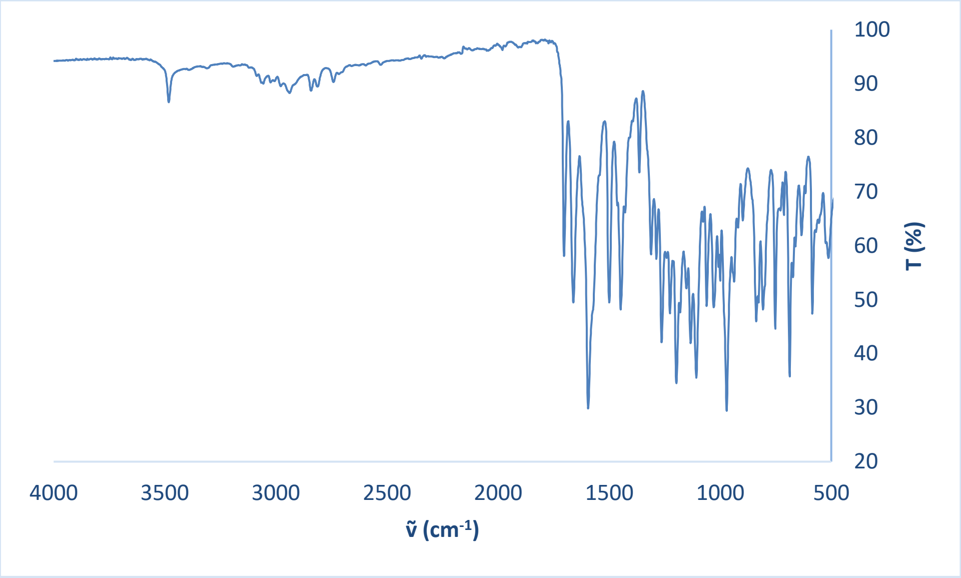
100
90
80
70
60
50
40
30
20
T (%)
4000
3500
3000
2500
2000
1500
1000
500
ṽ (cm-1)

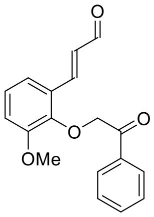
O
O
O
OMe

1h

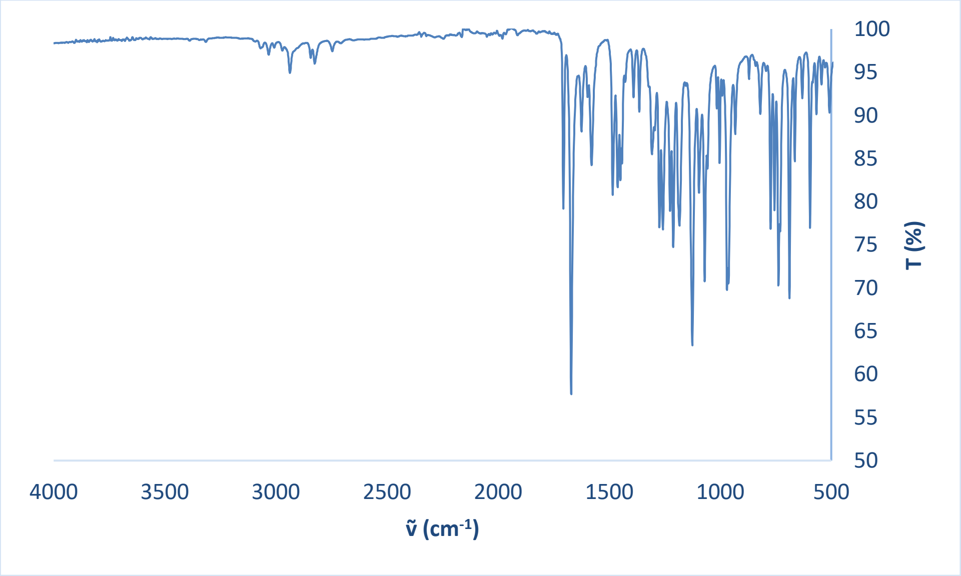
100
95
90
85
80
75
70
65
60
55
50
T (%)
4000
3500
3000
2500
2000
1500
1000
500
ṽ (cm-1)

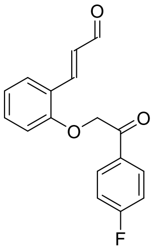
O
O
O
F

1i

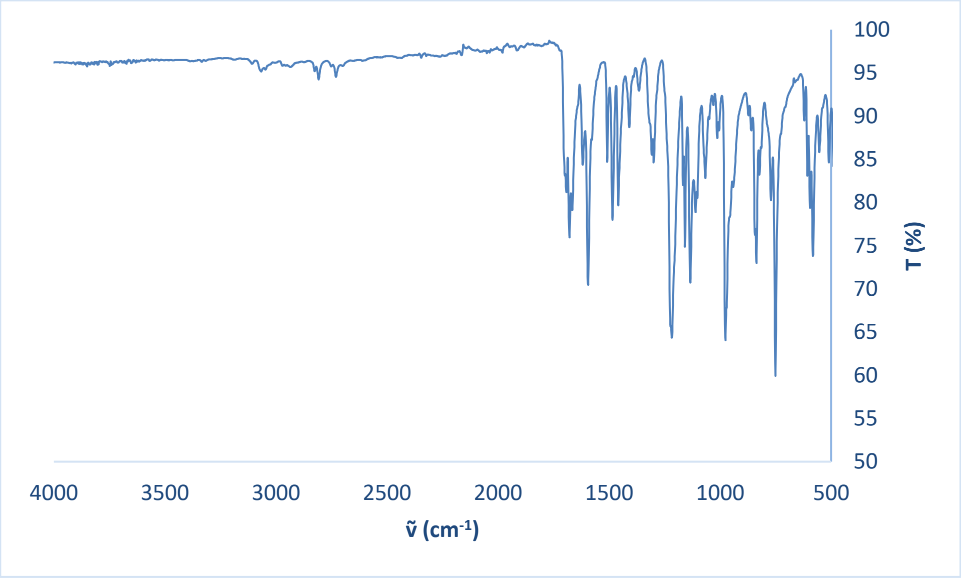
100
95
90
85
80
75
70
65
60
55
50
T (%)
4000
3500
3000
2500
2000
1500
1000
500
ṽ (cm-1)

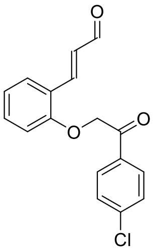
O
O
O
Cl

1j

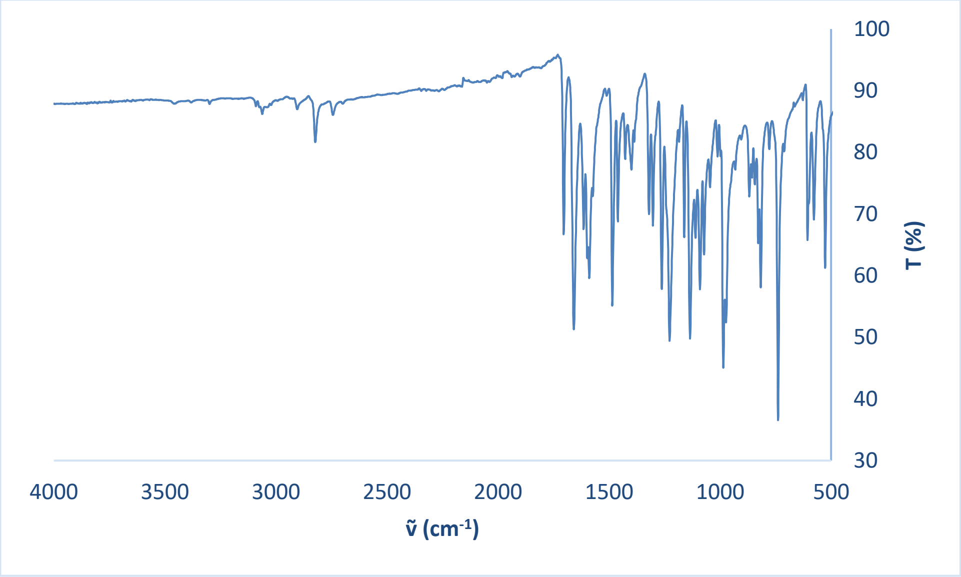
100
90
80
70
60
50
40
30
T (%)
4000
3500
3000
2500
2000
1500
1000
500
ṽ (cm-1)

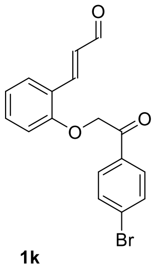
O
O
O
O
Br

1k

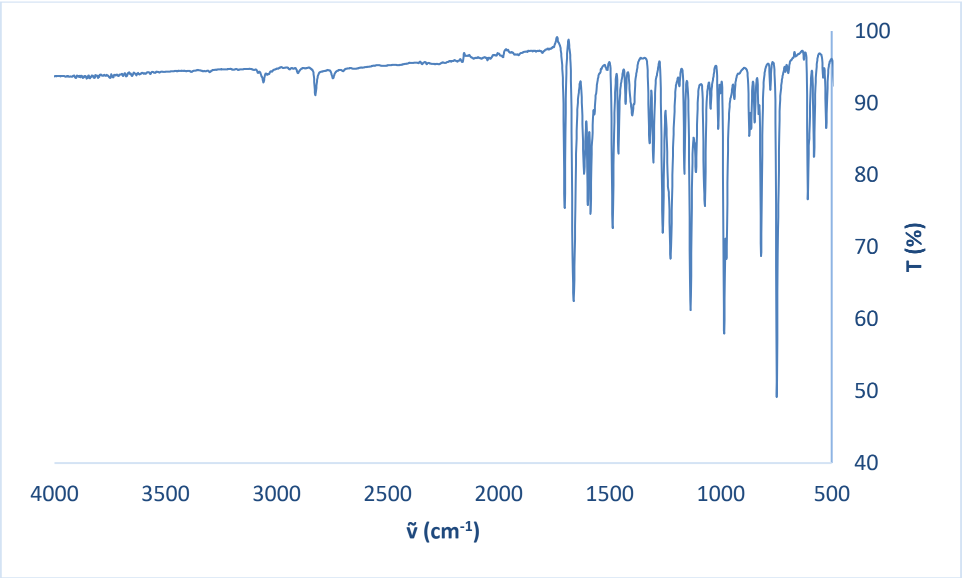
100
90
80
70
60
50
40
T (%)
4000
3500
3000
2500
2000
1500
1000
500
ṽ (cm-1)

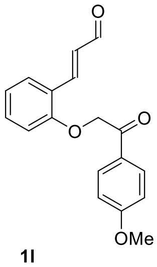
O
O
O
O
OMe

1l

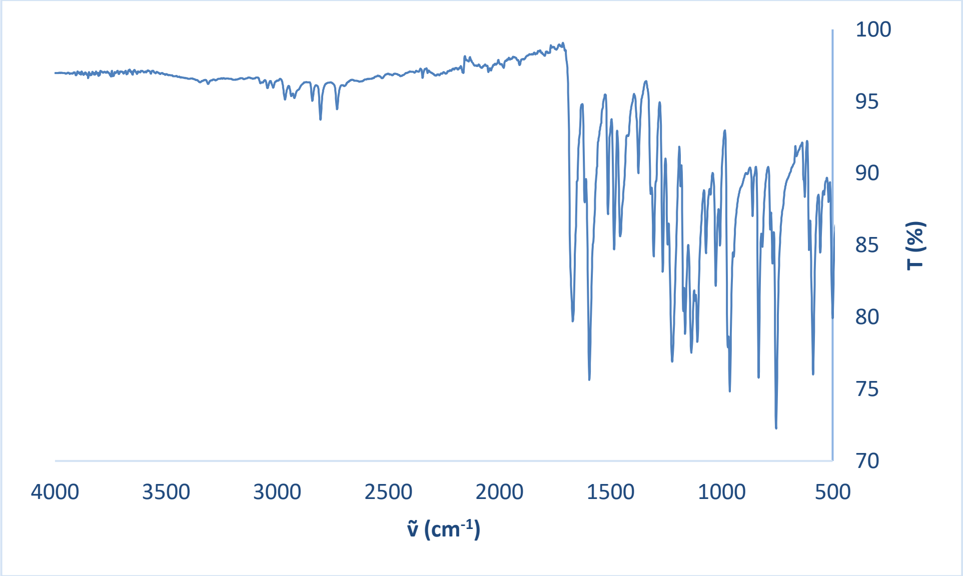
100
95
90
85
80
75
70
T (%)
4000
3500
3000
2500
2000
1500
1000
500
ṽ (cm-1)

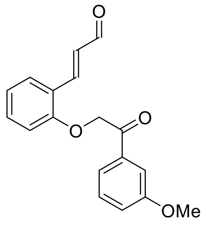
O
O
O
OMe

1m

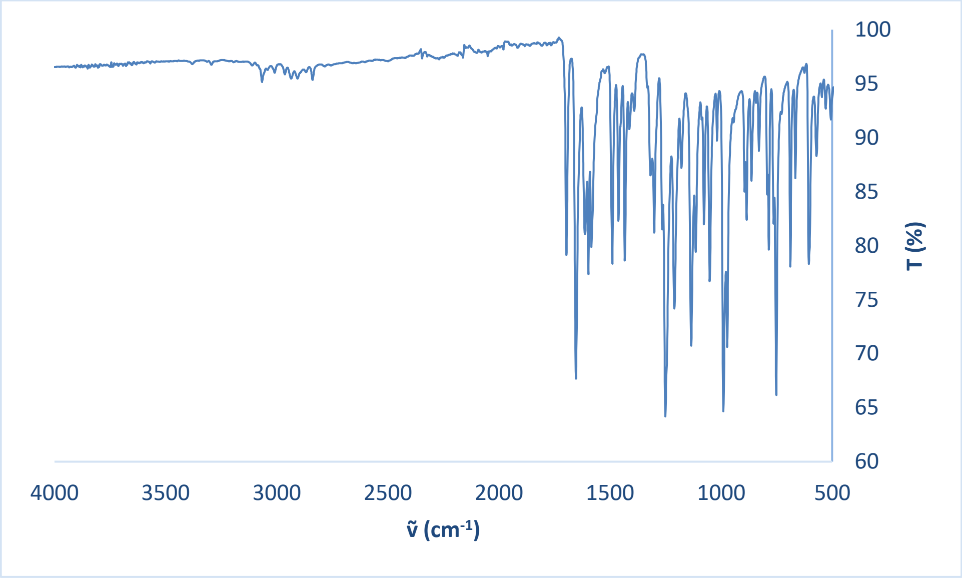
100
95
90
85
80
75
70
65
60
T (%)
4000
3500
3000
2500
2000
1500
1000
500
ṽ (cm-1)

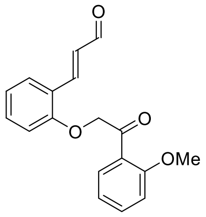
O
O
O
OMe

1n

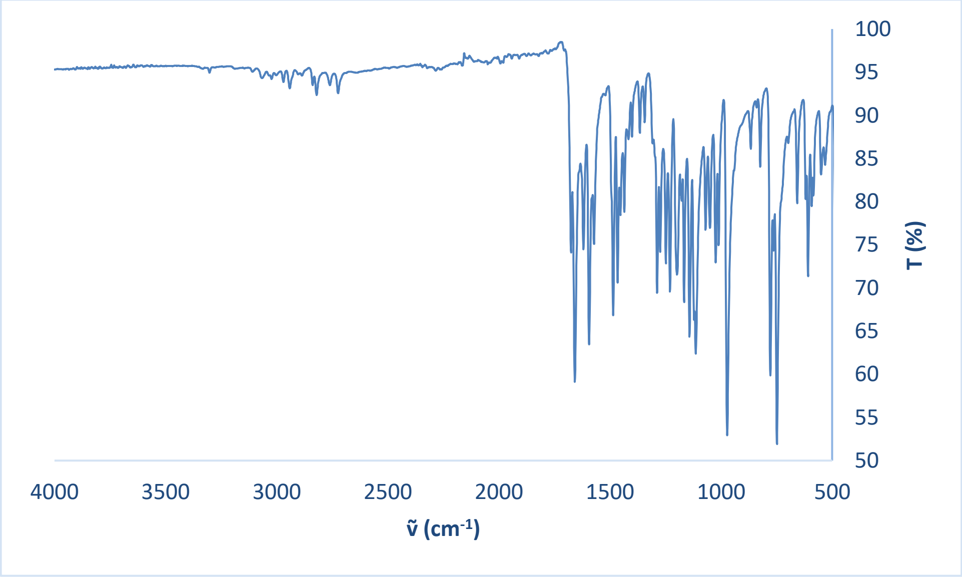
100
95
90
85
80
75
70
65
60
55
50
T (%)
4000
3500
3000
2500
2000
1500
1000
500
ṽ (cm-1)

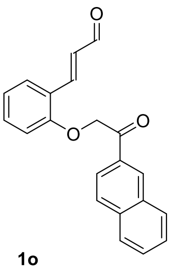

1o

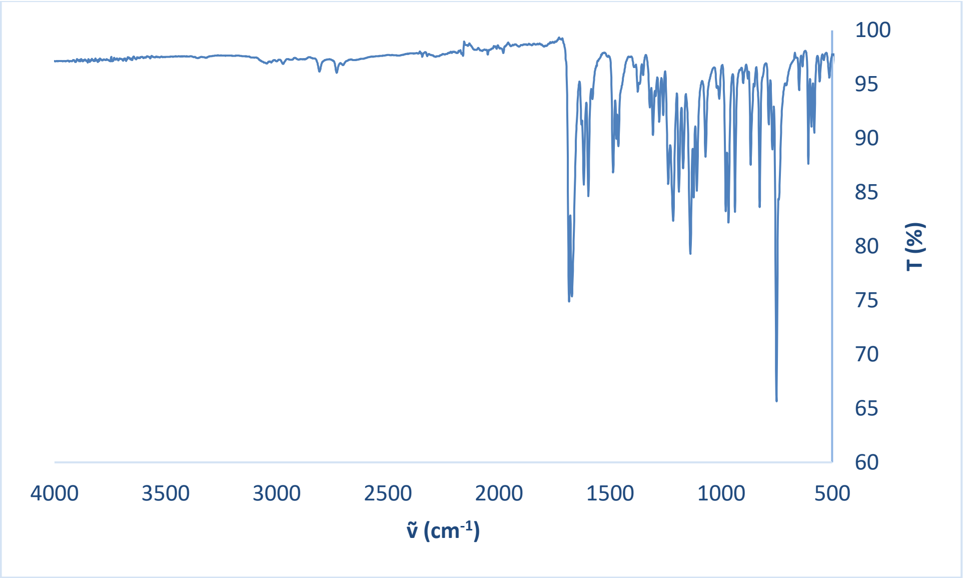
100
95
90
85
80
75
70
65
60
T (%)
4000
3500
3000
2500
2000
1500
1000
500
ṽ (cm-1)

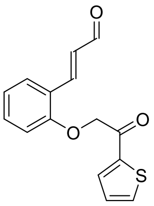

1p

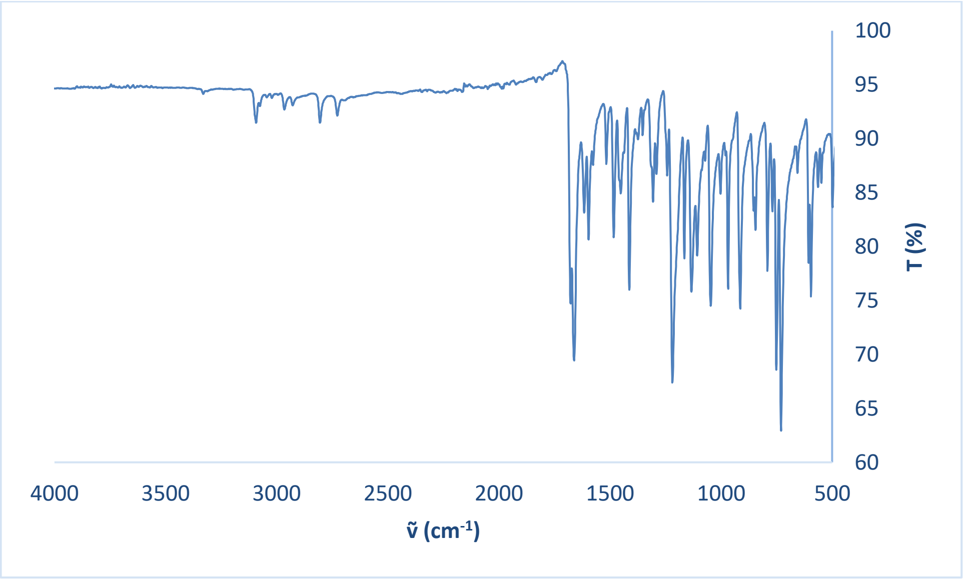
100
95
90
85
80
75
70
65
60
T (%)
4000
3500
3000
2500
2000
1500
1000
500
ṽ (cm-1)

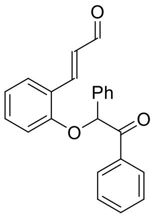

1q

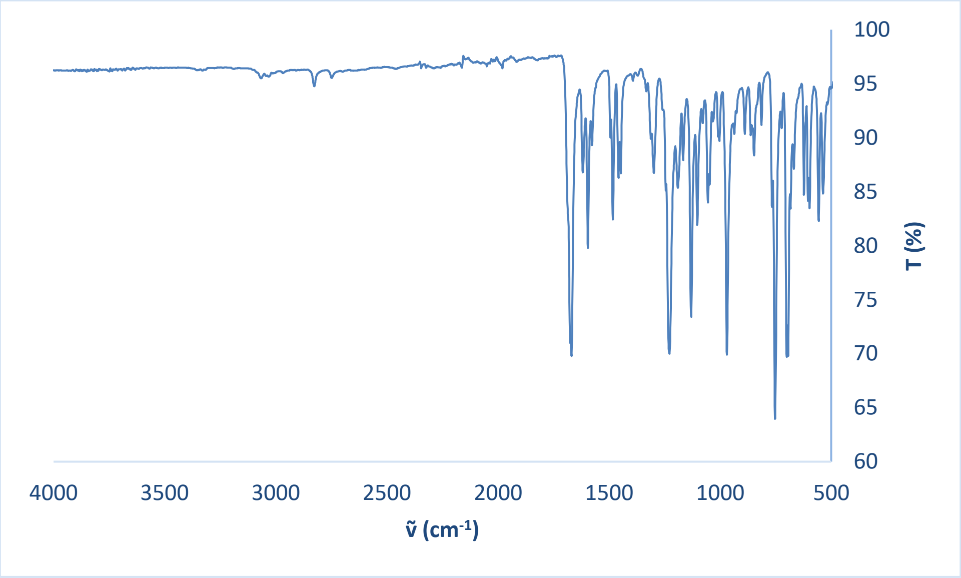
100
95
90
85
80
75
70
65
60
T (%)
4000
3500
3000
2500
2000
1500
1000
500
ṽ (cm-1)

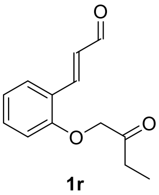

1r

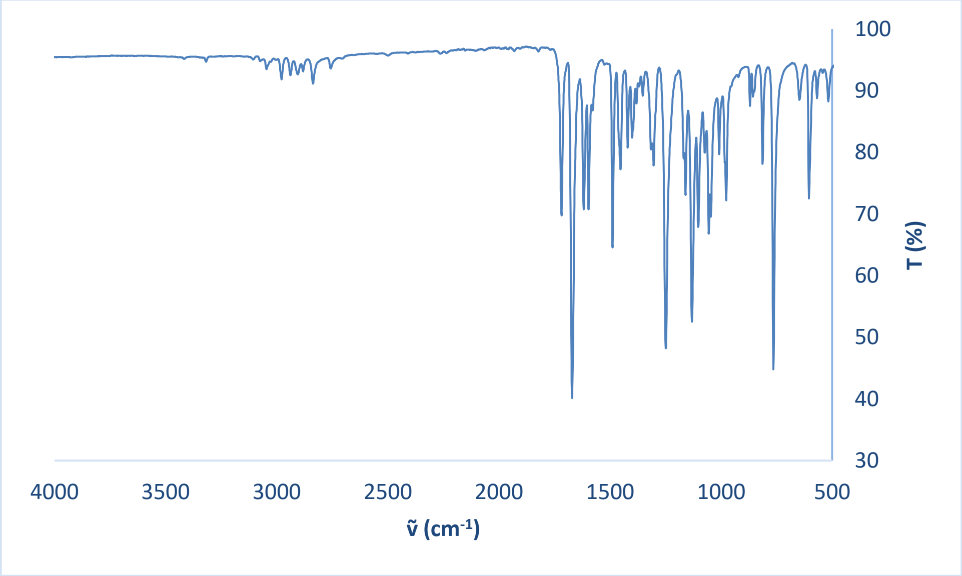
100
90
80
70
60
50
40
30
T (%)
4000
3500
3000
2500
2000
1500
1000
500
ṽ (cm-1)

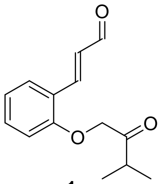

1s

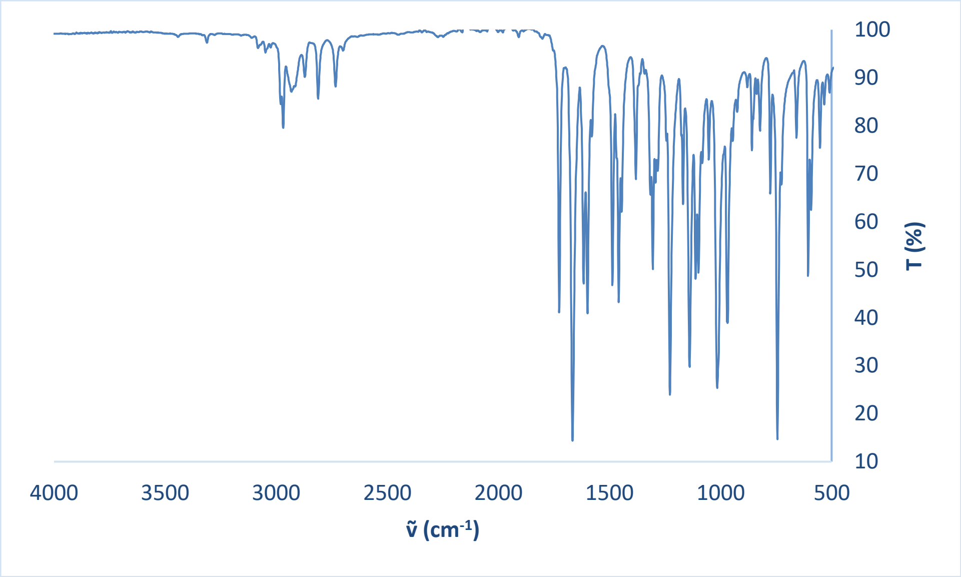

100
90
80
70
60
50
40
30
20
10
T (%)
4000
3500
3000
2500
2000
1500
1000
500
ṽ (cm-1)

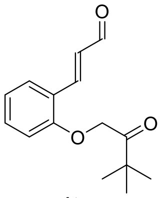

1t

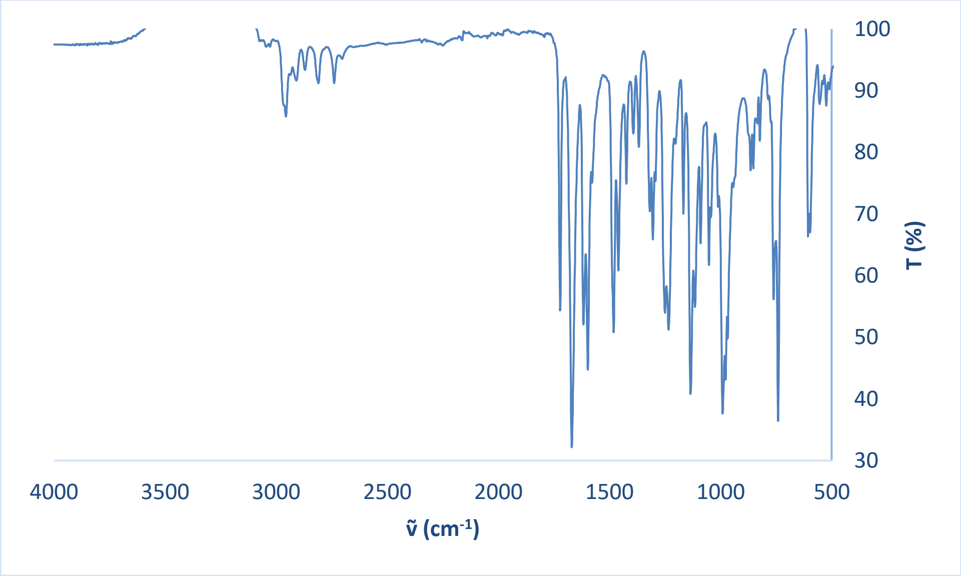

100
90
80
70
60
50
40
30
T (%)
4000
3500
3000
2500
2000
1500
1000
500
ṽ (cm-1)

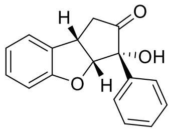
H
O
OH
O
H

3a

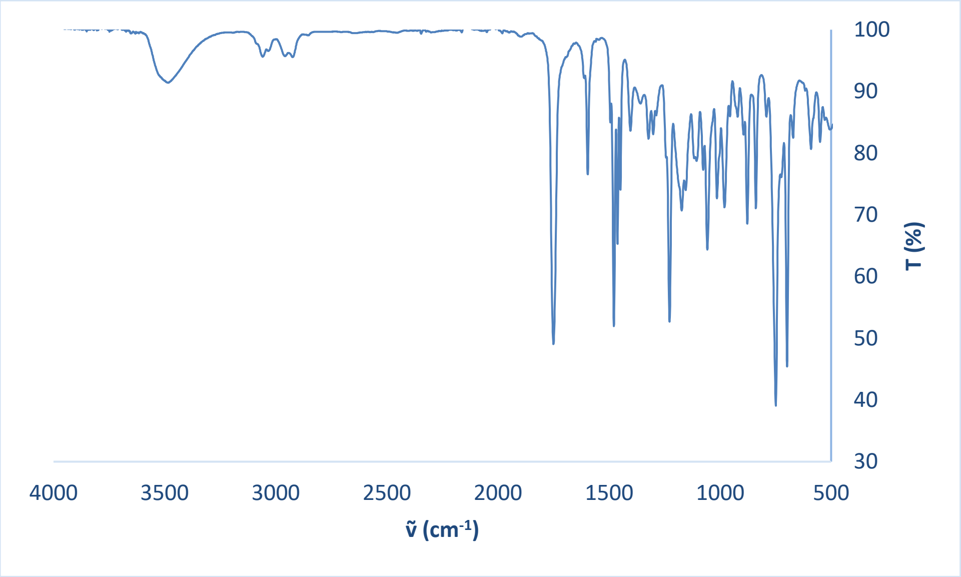
100
90
80
70
60
50
40
30
T (%)
4000
3500
3000
2500
2000
1500
1000
500
ṽ (cm-1)

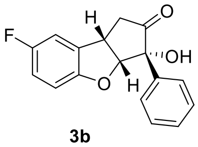
F
H
O
OH
O
H

3b

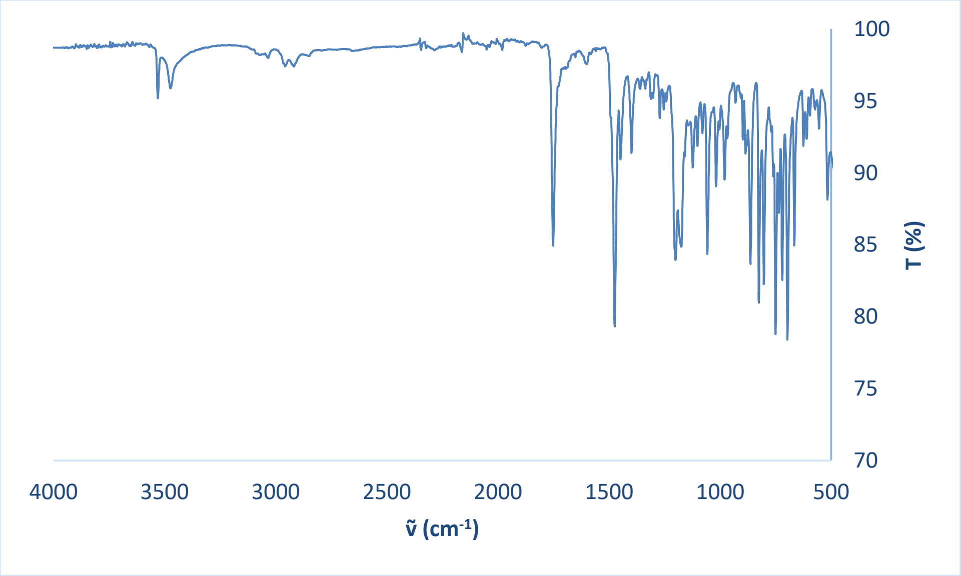
100
95
90
85
80
75
70
T (%)
4000
3500
3000
2500
2000
1500
1000
500
ṽ (cm-1)

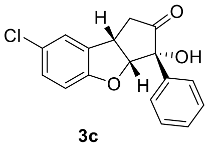
Cl
H
O
OH
O
H

3c

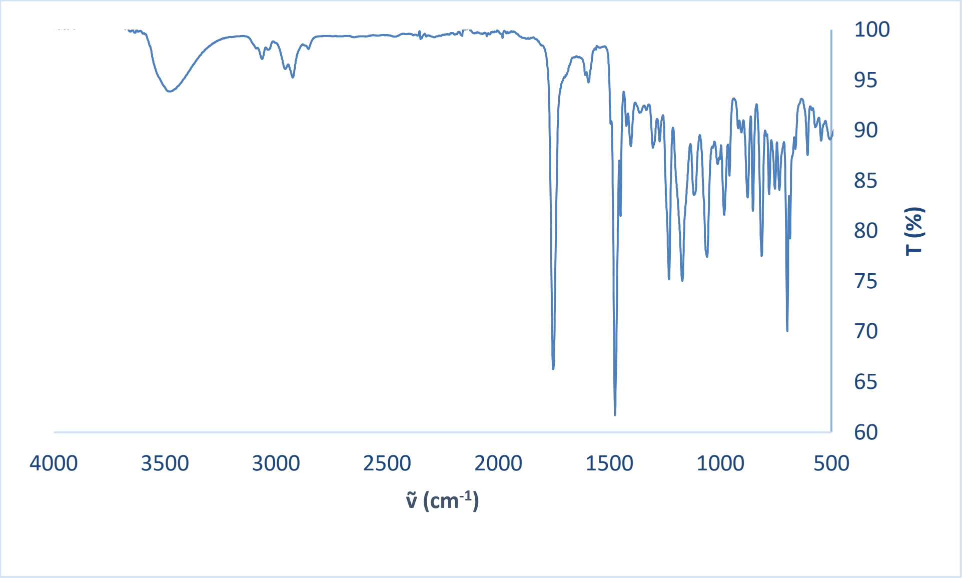
100
95
90
85
80
75
70
65
60
T (%)
4000
3500
3000
2500
2000
1500
1000
500
ṽ (cm⁻¹)

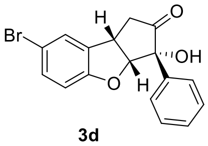
Br
H
O
OH
O
H

3d

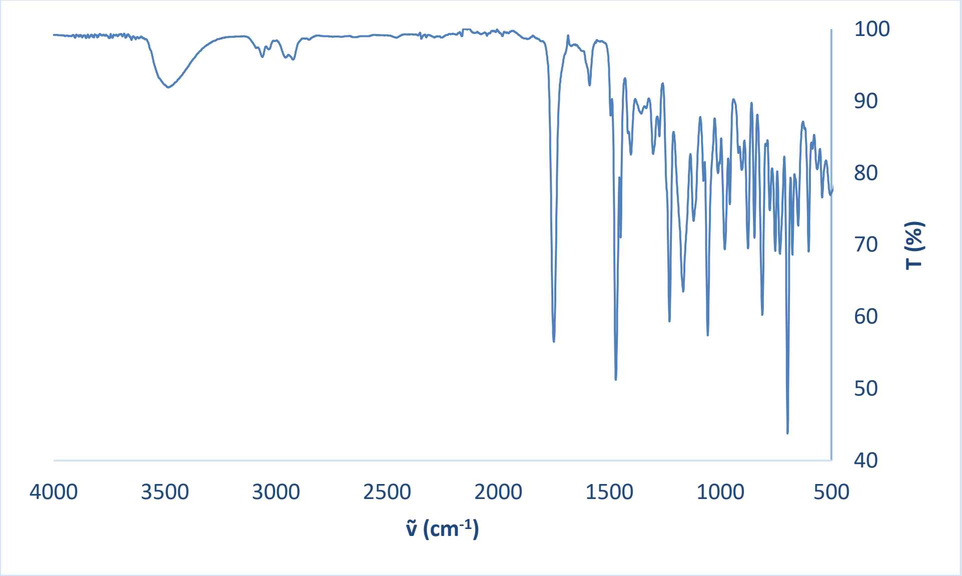
100
90
80
70
60
50
40
T (%)
4000
3500
3000
2500
2000
1500
1000
500
ṽ (cm⁻¹)

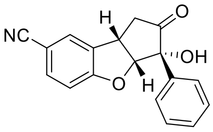
NC
H
O
OH
O
H

3e

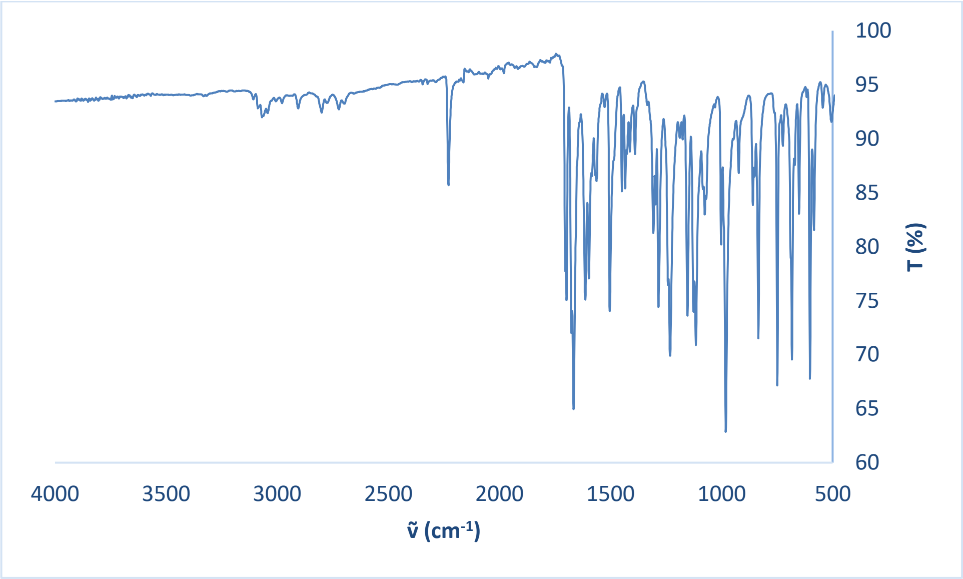
100
95
90
85
80
75
70
65
60
T (%)
4000
3500
3000
2500
2000
1500
1000
500
ṽ (cm-1)

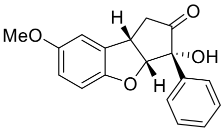
MeO
H
O
OH
O
H

3f

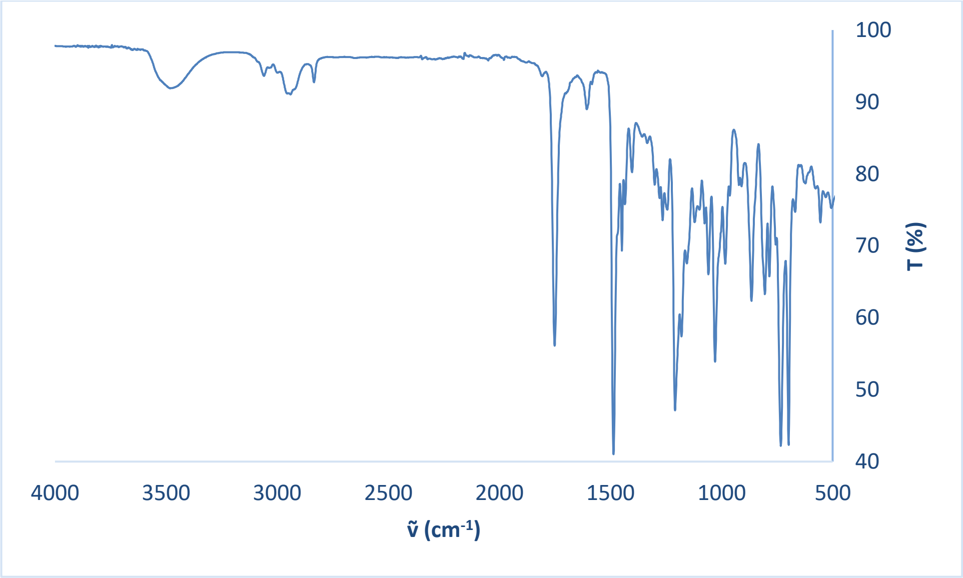
100
90
80
70
60
50
40
T (%)
4000
3500
3000
2500
2000
1500
1000
500
ṽ (cm-1)

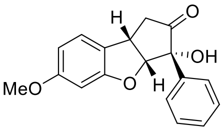
H
O
OH
MeO
O
H

3g

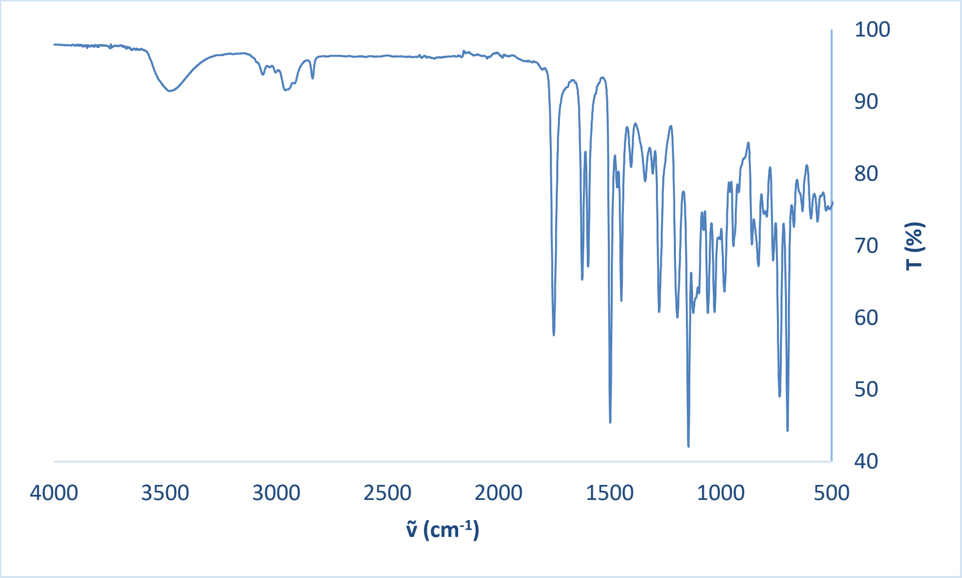
100
90
80
70
60
50
40
T (%)
4000
3500
3000
2500
2000
1500
1000
500
ṽ (cm-1)

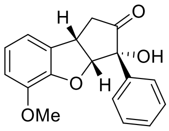
H
O
OH
O
H
OMe

3h

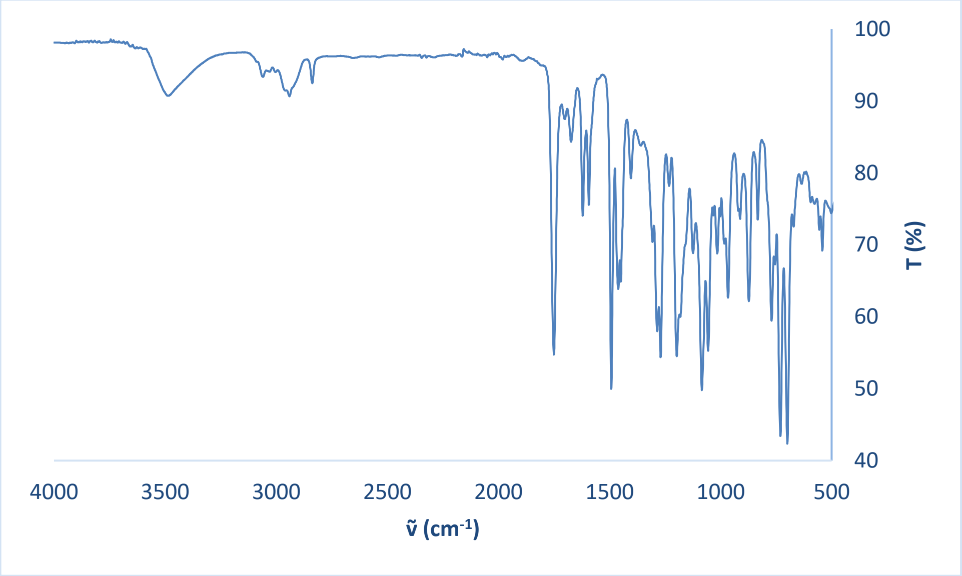
100
90
80
70
60
50
40
T (%)
4000
3500
3000
2500
2000
1500
1000
500
ṽ (cm-1)

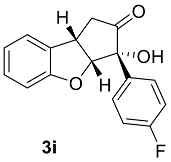

3i

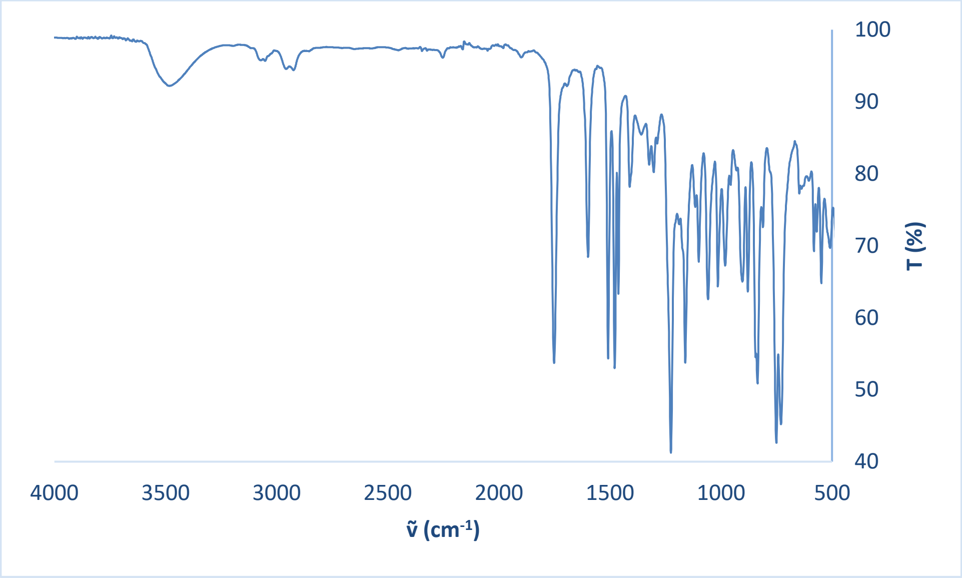
100
90
80
70
60
50
40
T (%)
4000
3500
3000
2500
2000
1500
1000
500
ṽ (cm-1)

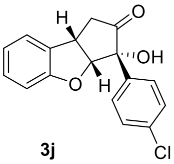

3j

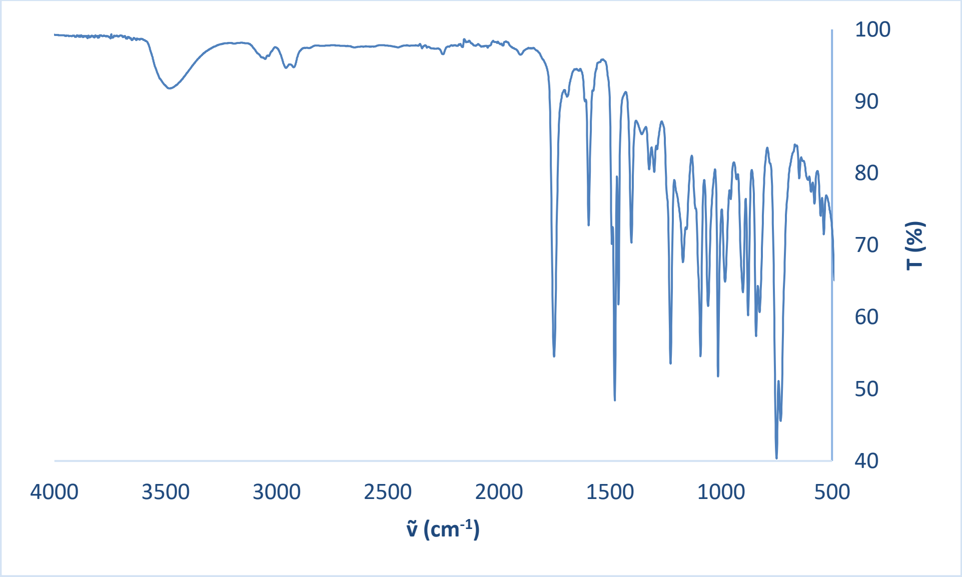
100
90
80
70
60
50
40
T (%)
4000
3500
3000
2500
2000
1500
1000
500
ṽ (cm-1)

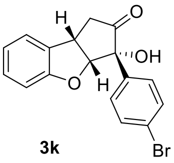

3k

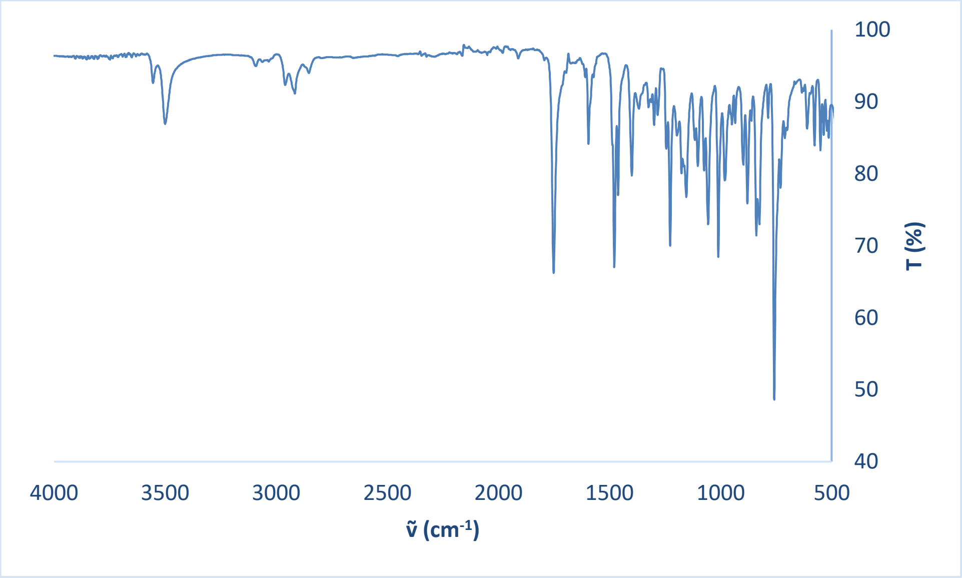
100
90
80
70
60
50
40
T (%)
4000
3500
3000
2500
2000
1500
1000
500
ṽ (cm-1)

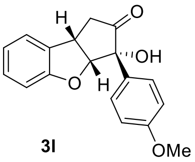

3l

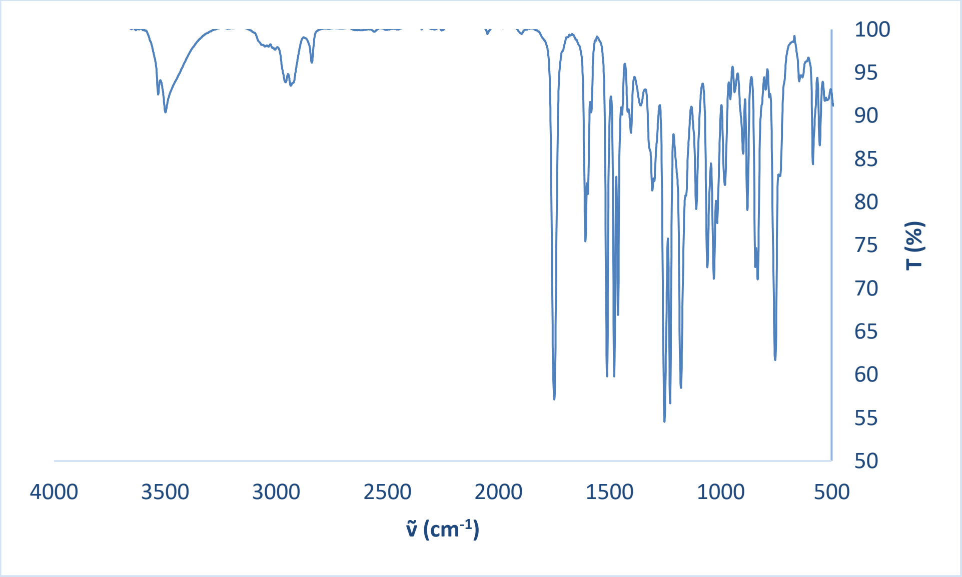
100
95
90
85
80
75
70
65
60
55
50
T (%)
4000
3500
3000
2500
2000
1500
1000
500
ṽ (cm-1)

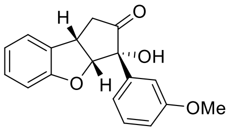
H
O
OH
O
H
OMe

3m

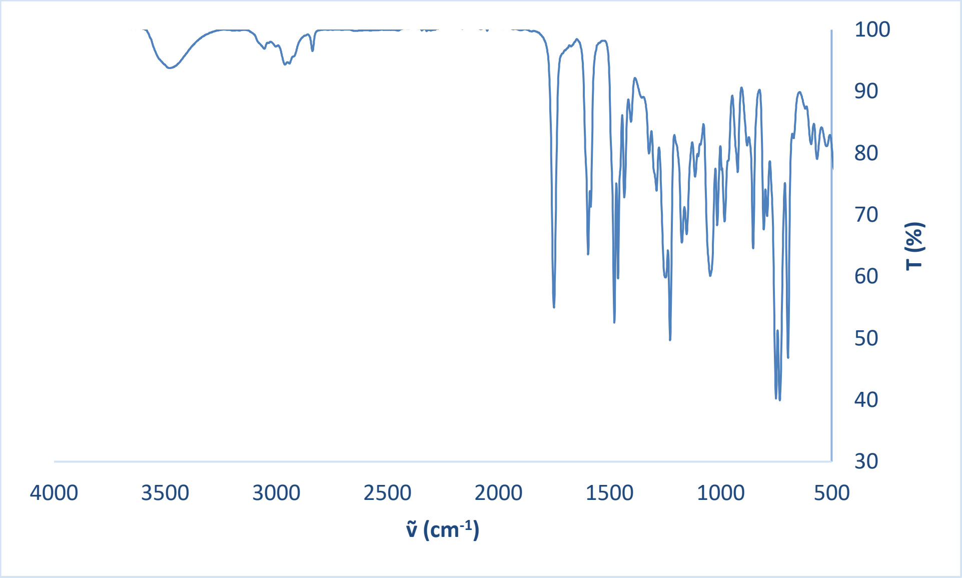
100
90
80
70
60
50
40
30
T (%)
4000
3500
3000
2500
2000
1500
1000
500
ṽ (cm-1)

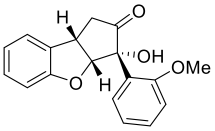
H
O
OH
OMe
O
H

3n

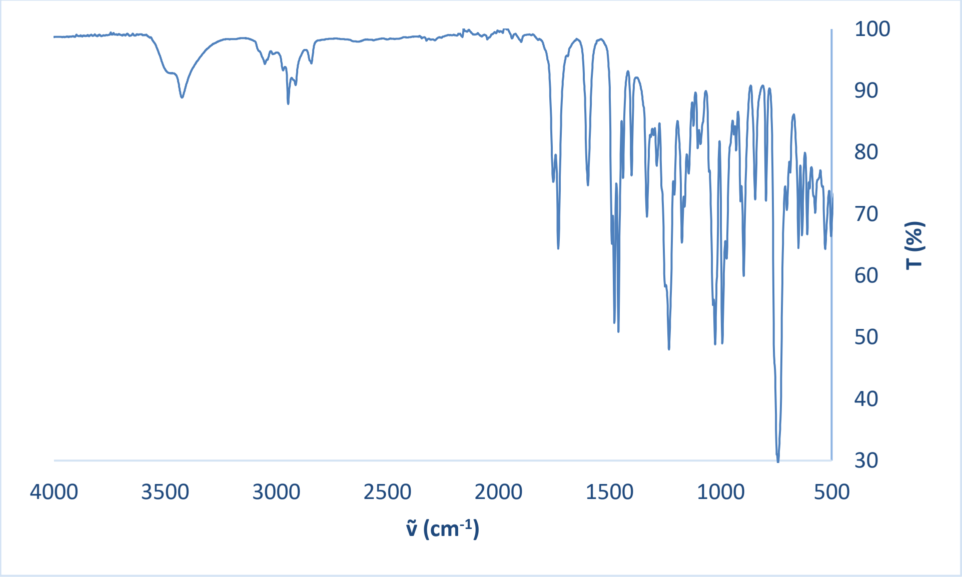
100
90
80
70
60
50
40
30
T (%)
4000
3500
3000
2500
2000
1500
1000
500
ṽ (cm-1)

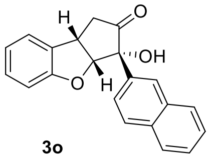
H
O
OH
O
H

3o

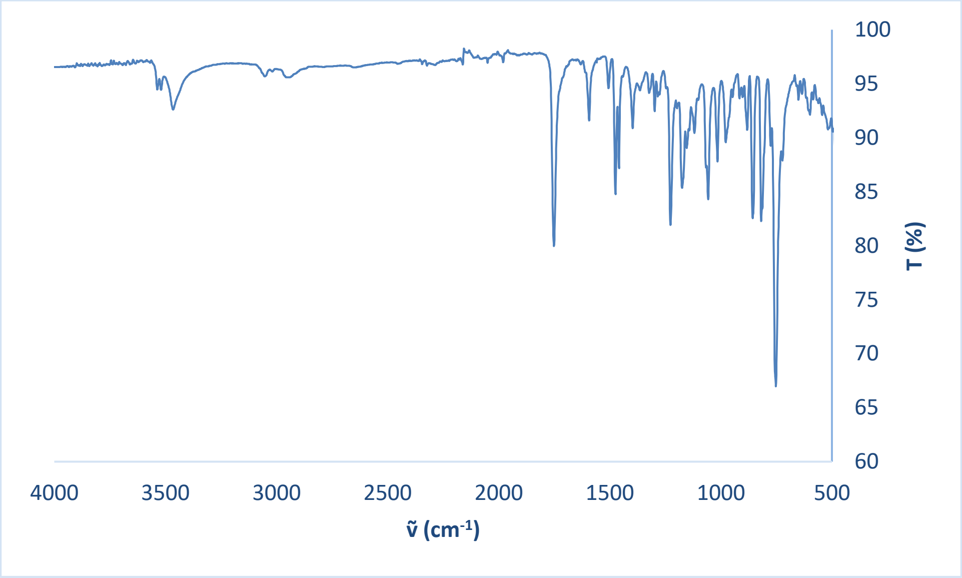
100
95
90
85
80
75
70
65
60
T (%)
4000
3500
3000
2500
2000
1500
1000
500
ṽ (cm-1)

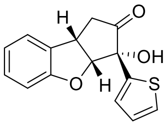
H
O
OH
O
H
S

3p

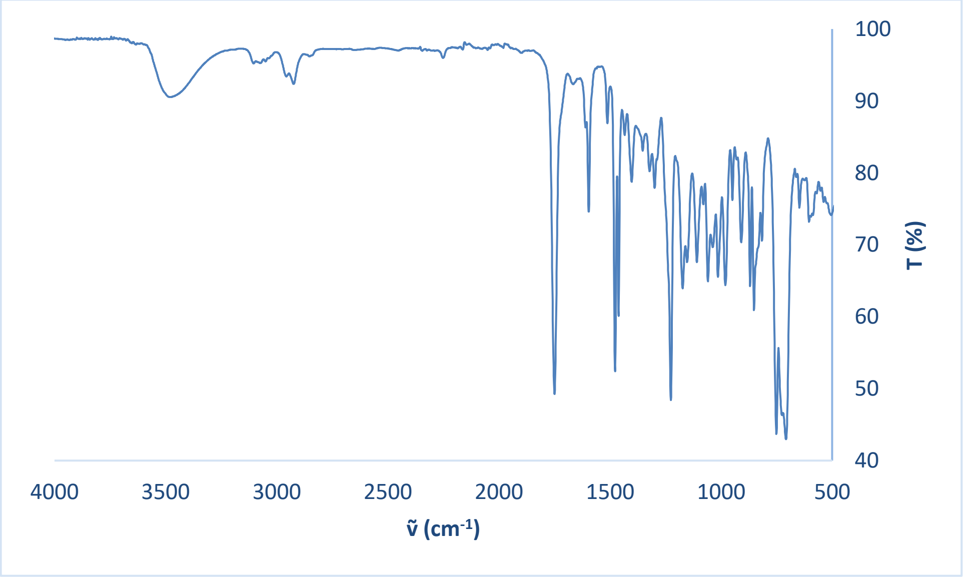
100
90
80
70
60
50
40
T (%)
4000
3500
3000
2500
2000
1500
1000
500
ṽ (cm-1)

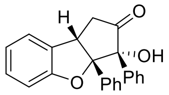
H
O
OH
O
Ph
Ph

3q

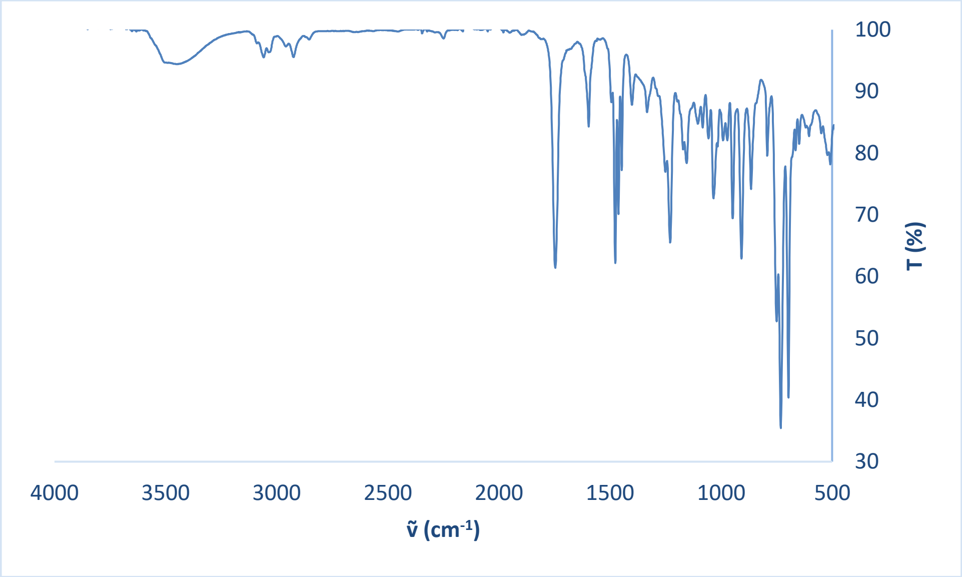
100
90
80
70
60
50
40
30
T (%)
4000
3500
3000
2500
2000
1500
1000
500
ṽ (cm-1)

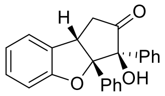
H
O
Ph
O
Ph
OH

***epi*-3q**

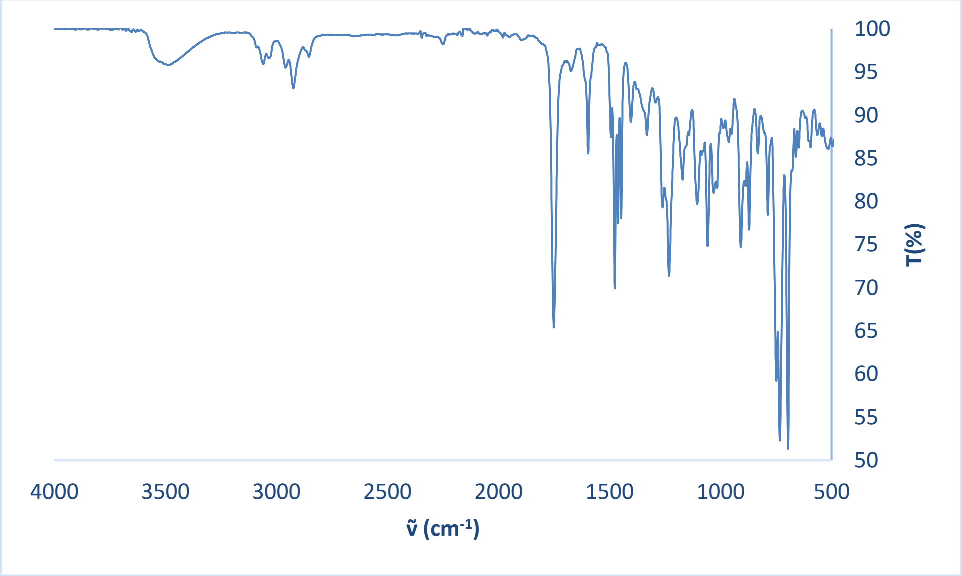
100
95
90
85
80
75
70
65
60
55
50
T(%)
4000
3500
3000
2500
2000
1500
1000
500
ṽ (cm-1)

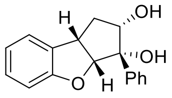
H
OH
OH
H
Ph
O

6

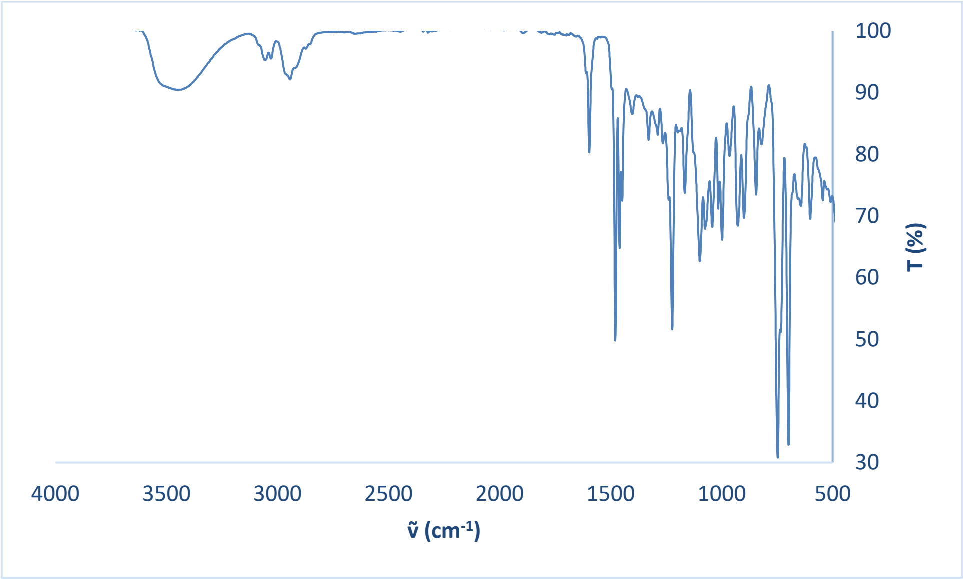
100
90
80
70
60
50
40
30
T (%)
4000
3500
3000
2500
2000
1500
1000
500
ṽ (cm-1)

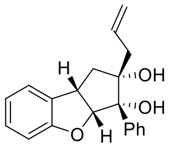
H
OH
OH
H
Ph
O

7

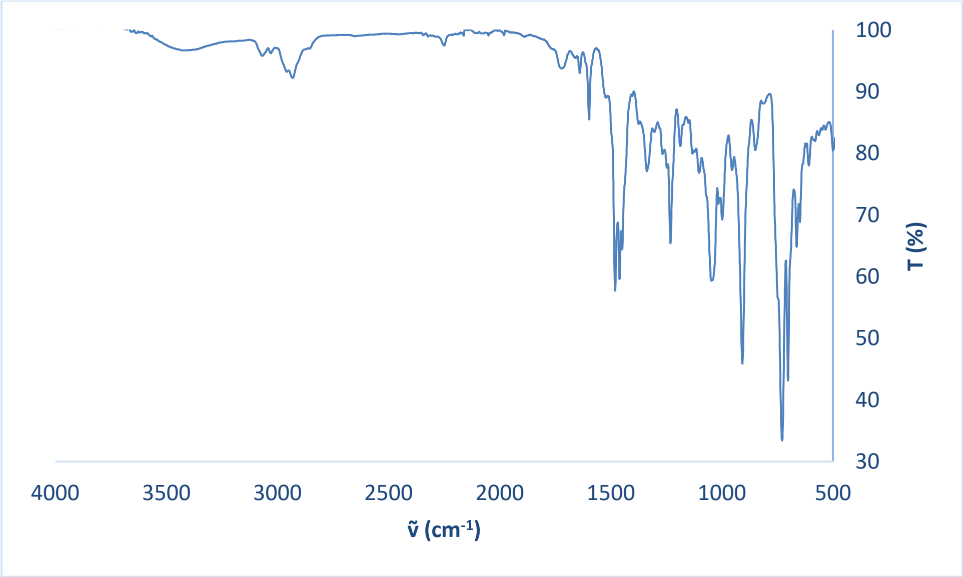
100
90
80
70
60
50
40
30
T (%)
4000
3500
3000
2500
2000
1500
1000
500
ṽ (cm-1)

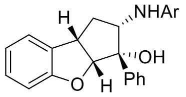

Ar = p-MeOC$_6$H$_5$
8

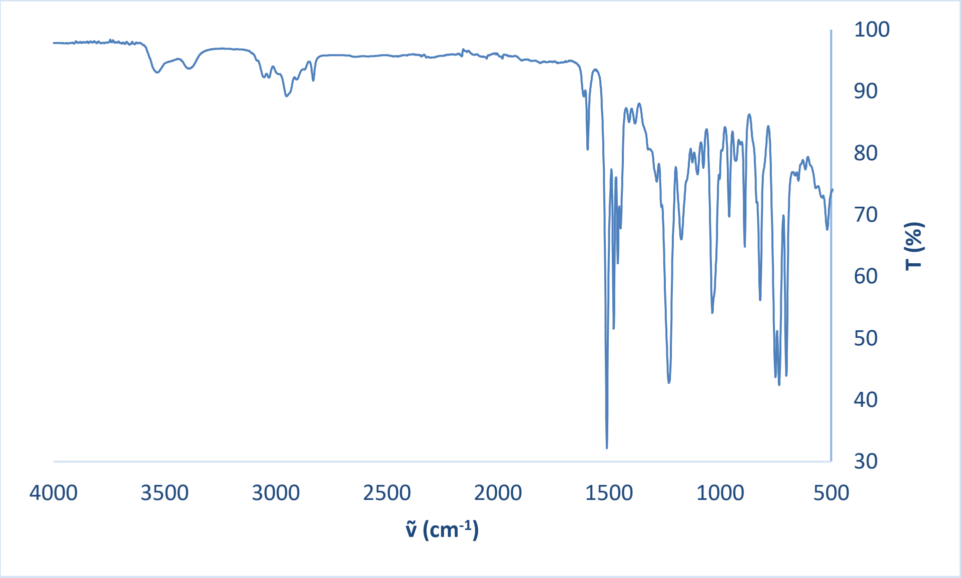

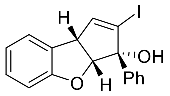

9

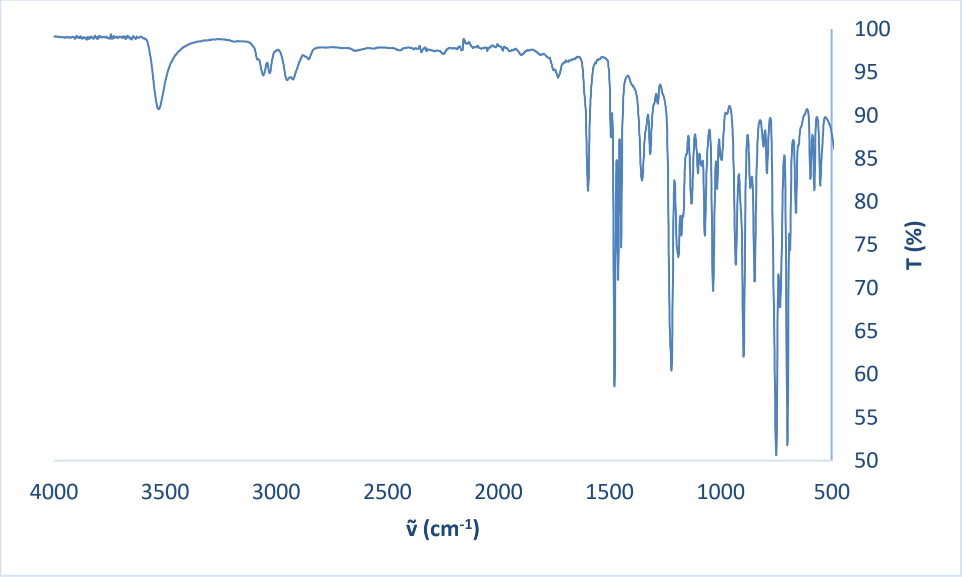

Supplement: Supplementary file 1 [file SC-008-C7SC03006A-s001.pdf]
